# Malic enzyme 1 senses L-lactate to determine tumor heterogeneity

**DOI:** 10.1038/s41392-026-02839-6

**Published:** 2026-09-11

**Authors:** Si-Yi Cao, Kewen Hu, Shijing Huang, Xiao-Lin Guan, Jian Wang, Yuan Shen, Hui Ming, Aihong Gu, Chao Wang, Xiao Shen, Qing Wu, Zhengjun Chen, Miao Yin, Wenyu Wen, Qun-Ying Lei

**Affiliations:** 1 Fudan University Shanghai Cancer Center & School of Basic Medical Sciences and Institutes of Biomedical Sciences; Cancer Institutes; Key Laboratory of Breast Cancer in Shanghai; Shanghai Key Laboratory of Radiation Oncology; Shanghai Key Laboratory of Medical Epigenetics, Shanghai Medical College, Fudan University, Shanghai, China; 2 Department of Oncology, Shanghai Medical College, Fudan University, Shanghai, China; 3 Shanghai Institute of Biochemistry and Cell Biology, Center for Excellence in Molecular Cell Science, Chinese Academy of Sciences, Shanghai, China; 4 Department of Neurosurgery, Huashan Hospital, Fudan University, Shanghai, China; 5 State Key Laboratory of Brain Function and Disorders and MOE Frontiers Center for Brain Science, Fudan University, Shanghai, China; 6 New Cornerstone Science Laboratory, School of Basic Medical Sciences, Fudan University, Shanghai, China

**Keywords:** Cell biology, Cancer

## Abstract

L-lactate is generally elevated in tumors and acts as a signaling molecule that promotes tumor progression. Here, we reveal that malic enzyme 1 (ME1) functions as a previously unrecognized sensor of L-lactate through direct binding at arginine 155 (R155), thereby potentiating malignancy. Mechanistically, L-lactate binding promotes the nuclear translocation of ME1, a process involving reduced acetylation at lysine 362 (K362) and facilitated by nuclear import of karyopherin-α 4 (KPNA4). Nuclear accumulation of ME1 enhances metastatic potential, which is correlated with increased interaction with hepatoma-derived growth factor (HDGF) and acquisition of an epithelial‒mesenchymal transition (EMT)-related phenotype. Under nutrient-deficient conditions, L-lactate promotes the assembly of a ME1-lactate dehydrogenase B (LDHB) complex, which enhances oxidative phosphorylation (OXPHOS) and increases ATP production, suggesting a metabolic adaptive mechanism that supports tumor cell survival. Notably, the ME1^R155A^ mutation, which disrupts L-lactate binding, abolishes the protumorigenic effect of the L-lactate-ME1 axis on tumor progression in vivo. In conclusion, our findings identify ME1 as a direct sensor of L-lactate and support a model in which lactate-mediated signaling and metabolic adaptation converge on ME1 to regulate tumor cell plasticity in a context-dependent manner under heterogeneous metabolic conditions. These insights advance our understanding of the spatiotemporal control of metabolic adaptation in cancer and reveal a potential therapeutic target.

## Introduction

Tumor heterogeneity poses a fundamental challenge to effective cancer therapy, particularly for prevalent solid malignancies such as those originating in the prostate, lung, and colon, which exhibit remarkable cellular diversity that undermines conventional treatments including chemotherapy, targeted therapy, and immunotherapy. Despite initial responses, recurrent disease almost invariably emerges, driven by subpopulations of cancer cells that survive therapeutic pressure. While morphological and genetic diversity have long been recognized as hallmarks of neoplastic disease, accumulating evidence reveals that metabolic heterogeneity constitutes an equally critical dimension of tumor complexity.^1,2^ Metabolic reprogramming has recently come to the forefront as a critical but underappreciated driver of tumor progression, which enables cancer cells to adapt dynamically to disparate microenvironmental conditions, thereby conferring competitive growth advantages and driving therapeutic resistance.^3^ A contemporary understanding has revealed that tumors are sophisticated, heterogeneous ecosystems characterized by pronounced spatial gradients in metabolite availability, including regional enrichment of glucose and glutamine, which are essential for bioenergetic and biosynthetic demands.^4^ The elevated consumption of these nutrients reflects their indispensable roles in ATP generation and biomass accumulation—processes requisite for sustained proliferation and metastatic dissemination. Such metabolic plasticity not only underlies phenotypic heterogeneity but also erects formidable barriers to therapeutic efficacy, as distinct metabolic dependencies and adaptive signaling circuits complicate the design of universal intervention strategies. Elucidating the mechanism underlying tumor metabolic heterogeneity thus represents a pressing priority with significant translational implications, particularly the identification of novel metabolic sensors that directly transduce metabolite fluctuations into pro-tumorigenic signaling cascades.

Among the manifestations of metabolic heterogeneity, the accumulation and spatially heterogeneous distribution of lactate are particularly important. Pioneering studies employing quantitative bioluminescence imaging first demonstrated that glucose and ATP exhibit marked regional variations within malignant tumors, establishing that the metabolic microenvironment is characterized by substantial spatial heterogeneity.^5^ As the terminal product of glycolytic metabolism, lactate achieves tissue concentrations averaging over 15 μmol/g, with localized peaks exceeding 40 μmol/g in specific tumor regions.^6^ This spatial variability has been systematically documented across diverse malignancies. In prostate cancer, sophisticated metabolic imaging modalities have provided direct visualization of lactate heterogeneity. By employing hyperpolarized [1-¹³C]-pyruvate magnetic resonance spectroscopic imaging (MRSI), the rapid conversion of hyperpolarized pyruvate to lactate in PC3 and DU145 tumor cell line xenografts was demonstrated; corresponding MRI revealed distinct lactate intensities across tumor subregions.^7^ Complementing these findings, ¹H MRSI was applied to map whole-tumor lactate distribution in Myc-CaP prostate tumors, documenting intratumoral lactate concentrations ranging from 3.3 to 20.8 mM (mean 7.9 mM).^8^ Similarly, lung cancer and colorectal cancer exhibit pronounced lactate heterogeneity with substantial clinical relevance.^9,10^ These observations establish that lactate is a critical determinant of tumor heterogeneity across solid malignancies. Transcending its traditional conceptualization as a metabolic waste product, lactate has emerged as a pivotal signaling molecule with pleiotropic biological functions. It actively remodels the tumor microenvironment through modulation of antitumor immunity, directly influences cell cycle dynamics via regulation of the anaphase-promoting complex,^11^ and drives neoplastic evolution through epigenetic modifications.^12^ Despite these advances, the repertoire of direct intracellular lactate sensors capable of transducing metabolic cues into specific signaling cascades remains remarkably limited, leaving critical gaps in our understanding of how neoplastic cells decode lactate abundance to execute adaptive programs. Given these multifaceted roles in cancer progression, the identification and characterization of lactate-sensing mechanisms constitute an essential frontier for understanding metabolic plasticity and may illuminate novel avenues for therapeutic intervention.

Malic enzyme 1 (ME1) occupies a central nexus in cellular metabolism, potentially bridging bioenergetic adaptation with signal transduction.^13^ This cytosolic enzyme catalyzes the oxidative decarboxylation of malate to pyruvate with the concomitant reduction of NADP⁺ to NADPH.^13^ Together with its mitochondrial isoforms ME2 and ME3, ME1 constitutes a critical source of NADPH—the essential electron donor for fatty acid and cholesterol biosynthesis, as well as cellular redox homeostasis. ME1 was originally identified as an insulin-responsive gene in hepatocytes and adipocytes and has been implicated in metabolic syndrome and diabetes pathogenesis.^13^ More recently, ME1 has been recognized as a pro-oncogenic factor across multiple epithelial malignancies that promotes cancer cell proliferation, antioxidant defense, and cellular senescence bypass.^14–16^ Notably, posttranslational modifications, particularly reversible acetylation, critically govern ME1 enzymatic activity and protein stability, suggesting that ME1 function may be dynamically tuned by metabolic microenvironmental cues.^17^

This study identifies ME1 as a direct L-lactate sensor that enables metabolic plasticity in cancer. In *Pten*-deficient prostate cancer, we demonstrated that accumulated L-lactate binds to ME1 directly, promoting its nuclear translocation to accelerate metastasis. Under nutrient deprivation, the assembly of the L-lactate-ME1-LDHB complex supports cell survival and metabolic adaptation. The ME1^R155A^ mutation abolishes lactate binding and abrogated these protumorigenic effects. We extend these findings to lung and colorectal cancer, establishing that the L-lactate-ME1 axis operates as a conserved metabolic sensing mechanism across diverse epithelial malignancies. Collectively, these results reveal that the L-lactate-ME1 axis is a critical mediator of tumor adaptation and a pancancer therapeutic target, offering a rationale for developing small-molecule disruptors of this interaction to overcome treatment resistance.

## Results

### L-lactate increases ME1 expression levels, and prostate-specific knockout of *Me1* suppresses tumor progression in *Pten*-deficient mice

**Fig. 1:**
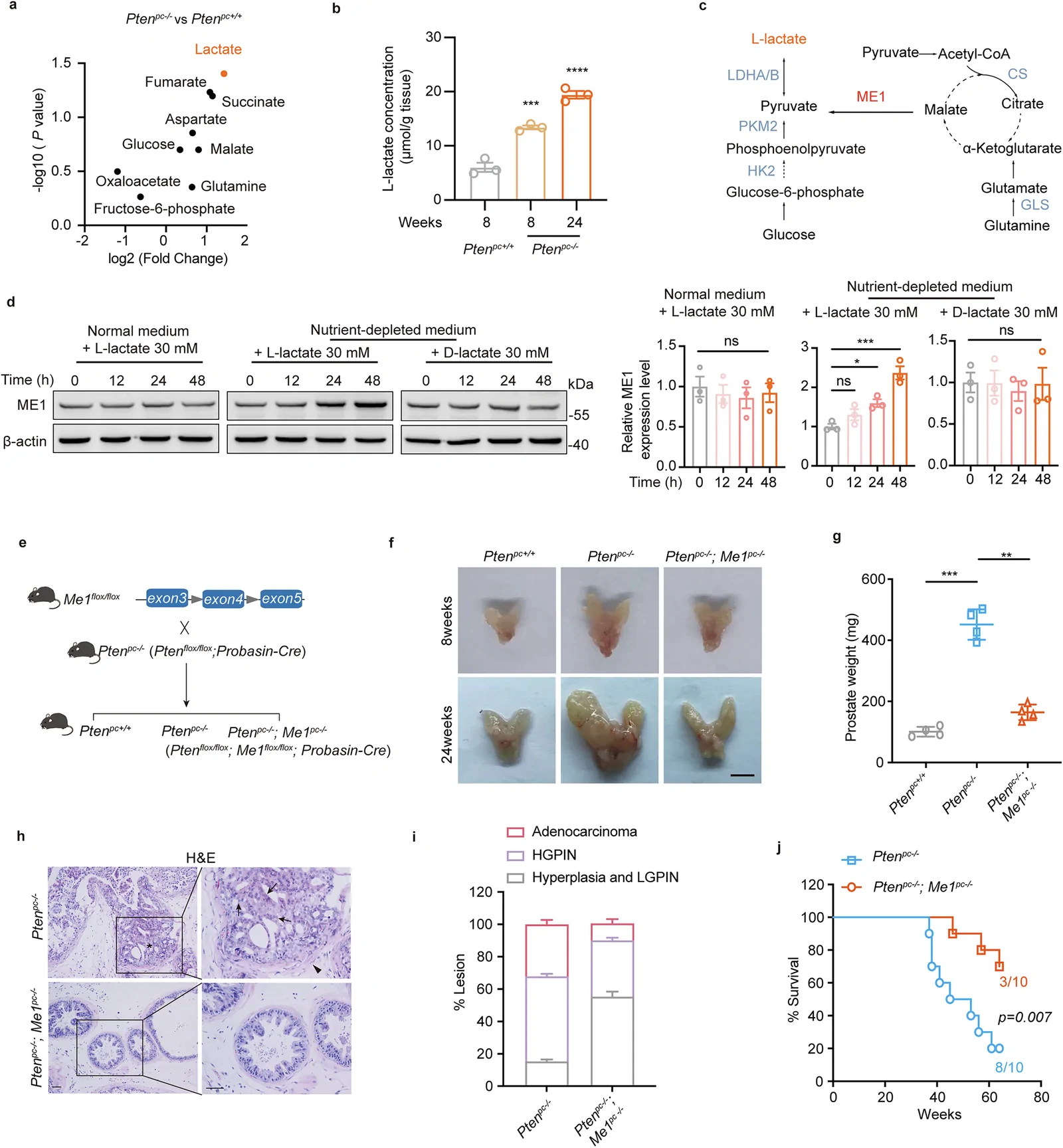
L-lactate increases ME1 expression level and prostate-specific knockout of *Me1* decreases tumorigenesis. **a** Lactate is accumulated in the prostate tissue from *Pten*^*pc-/-*^ mice. The scatter plot shows the relative level of metabolites in the prostate tissue from *Pten*^*pc+/+*^ and *Pten*^*pc-/-*^ mice analyzed by LC-MS experiment (*n* = 3 biologically independent animals, unpaired *t* test). **b** The concentration of L-lactate is increased up to about 20 μmol/g prostate tissue in 24-week-old *Pten*^*pc-/-*^ mice (*n* = 3, mean ± s.e.m., One-way ANOVA). **c** Schematic representation of L-lactate catabolism. The dashed arrow represents multiple reactions. HK2, hexokinase 2; PKM2, pyruvate kinase M2; LDHA/B, lactate dehydrogenase A/B; ME1, malic enzyme 1; CS, citrate synthase; GLS, glutaminase. **d** Increased ME1 expression level in DU145 cells under the nutrient-depleted medium with sodium L-lactate but not sodium D-lactate. 1 mM pyruvate, 25 mM glucose, and 4 mM glutamine in normal medium. 0.1 mM pyruvate, 2.5 mM glucose, and 0.4 mM glutamine in nutrient-depleted medium. Representative western blot (WB) images (left panel) and quantification (right panel) for ME1 expression level are shown (mean ± s.e.m., One-way ANOVA). **e** Generation of prostate tissue-specific *Me1*-knockout mice. **f**, **g** Prostate-specific deletion of *Me1* with *Pten* deficiency reduces the mouse prostate tumor. Representative images of prostates separated from 8-week and 24-week-old *Pten*^*pc+/+*^*, Pten*^*pc-/-*^, and *Pten*^*pc-/-*^*; Me1*^*pc-/-*^ mice (**f**). Quantification of prostate weight from *Pten*^*pc+/+*^, *Pten*^*pc-/-*^, and *Pten*^*pc-/-*^*; Me1*^*pc-/-*^ mice at 24 weeks of age (*n* = 4 biologically independent animals, mean ± s.d., Welch ANOVA tests) (**g**). Scale bar, 0.5 cm. **h** Representative H&E staining images of 24-week-old mice prostates. The typical cribriform epithelial proliferation (asterisk) with enlarged nuclei and scattered macronuclei (arrow) are shown in the *Pten*^*pc-/-*^ mice group with hypercellular stroma (arrowhead). Scale bar, 40 μm. **i** Quantification of histological grade by H&E staining. **j** Kaplan–Meier survival plots of *Pten*^*pc-/-*^ and *Pten*^*pc-/-*^*; Me1*^*pc-/-*^ mice (*n* = 10 biologically independent animals)

Metabolic reprogramming is a well-known hallmark of tumors. However, the essential functions of various metabolites in tumorigenesis remain largely unknown. A *Pten* null murine PCa model, in which *Pten* is deleted from the prostate epithelium, is a commonly adopted methodology associated with PCa research.^18^ We used this murine model of PCa through *Probasin-Cre*-mediated deletion of *Pten* (*Pten*^*flox/flox*^*; PB-cre4;* termed *Pten*^*pc-/-*^) to elucidate the role of critical metabolites in PCa development.^19^ Targeted metabolomic analyses of intermediate metabolites of the glucose/glutamine-lactate metabolic pathway in prostate tissue revealed that lactate levels were significantly greater in *Pten*^*pc-/-*^ mice than in *Pten* ^*pc+/+*^ mice (Fig. 1a). Lactate has two stereoisomeric forms, L-lactate and D-lactate. Normally, L-lactate is the most extensive form in mammalian cells, whereas D-lactate is present at minimum concentrations because of the degradation of methylglyoxal.^20^ The accumulation of L-lactate is a widely recognized indicator of the presence of tumors. We detected L-lactate concentrations of approximately 20 μmol/g in prostate tissues from 24-week-old *Pten*^*pc-/-*^ mice (Fig. 1b). To exclude the effect of the osmotic pressure on tumor cells upon L-lactate treatment, cell culture media were supplemented with low concentrations of sodium chloride (NaCl), and then, the corresponding concentrations of sodium L-lactate (a pH-neutral form of L-lactate) and/or NaCl were added to obtain the same equal amounts of Na^+^ in the final culture media as normal culture medium. The pH value of the customized culture medium was approximately 7.8 (Supplementary Fig. 1a), and the level of cellular lactate increased when sodium L-lactate was added (Supplementary Fig. 1b). Lactate can inhibit tumor cell line growth through the inhibition of glycolysis.^21^ Similarly, we also observed the inhibitory effect of L-lactate on the proliferation of both the PC3 and DU145 PCa cell lines in normal culture media supplemented with high concentrations of glucose and glutamine (Supplementary Fig. 1c). However, in nutrient-depleted medium with low levels of glucose and glutamine, the addition of sodium L-lactate increased the cellular lactate level (Supplementary Fig. 1d) and significantly reversed the inhibition of PC3 cell status (Supplementary Fig. 1e). To explore the exact role of L-lactate under different nutrient conditions, we detected the effects of L-lactate on the expression of the related enzymes that are responsible for L-lactate metabolism (Fig. 1c) in human PCa DU145 cells cultured in normal or nutrient-depleted medium formulated with low concentrations of pyruvate, glucose, and glutamine. The expression levels of multiple metabolic enzymes increased in response to nutrient-depleted medium supplemented with different concentrations of sodium L-lactate (Supplementary Fig. 1f–h). Among these enzymes, ME1 expression was significantly upregulated in an L-lactate dose-dependent manner and was higher than that of its cousins, ME2 and ME3 (Supplementary Fig. 1f–h). Moreover, L-lactate but not D-lactate induced ME1 expression in a time-dependent manner (Fig. 1d). In addition, the expression levels of LDHA and LDHB, the crucial enzymes involved in L-lactate utilization,^22^ were dramatically increased upon stimulation with sodium L-lactate (Supplementary Fig. 1f–h), indicating that ME1 and LDH may be indispensable for the function of L-lactate in PCa cells. In addition, L-lactate increased the ME1 expression level in A549 (lung) and HCT116 (colorectal) cancer cells cultured with nutrient-depleted medium (Supplementary Fig. 1i). Next, the clinical relevance of ME1 in human PCa was evaluated using a tumor tissue microarray (TMA) consisting of specimens from patients with PCa. The results revealed significantly elevated expression levels of ME1 in cancer tissues compared with their adjacent normal prostate epithelia (Supplementary Fig. 1j). Notably, data from the TCGA database indicated that the survival rate of cancer patients with high levels of *ME1* was significantly lower than that of patients with low levels of *ME1* (Supplementary Fig. 1k).

To investigate the crucial functional role of ME1 in PCa, we constructed a genetically modified Me1 mouse strain, identified as *Me1*^*flox/flox*^ mice, and crossed it with *Probasin-Cre4* mice to obtain prostate-specific *Me1* deletion mice (*Me1*^*pc-/-*^) (Supplementary Fig. 2a). There was no difference in prostate weight or histology between *Me1*^*pc-/-*^ and *Me1*^*pc+/+*^ mice (Supplementary Fig. 2b, c). These mice were subsequently crossbred with *Pten*^*pc-/-*^ mice to generate *Pten*^*flox/flox*^*; Me1*^*flox/flox*^*; PB-Cre4* (*Pten*^*pc-/-*^*; Me1*^*pc-/-*^) mice (Fig. 1e). The depletion efficiency of Me1 was verified by western blot analysis (Supplementary Fig. 2d). Compared with *Pten*^*pc-/-*^ mice, the average prostate weight of the 24-week-old *Pten*^*pc-/-*^; *Me1*^*pc-/-*^ mice decreased (Fig. 1f, g). Consistent with these findings, specific deletion of *Me1* in the prostate delayed the progression of prostatic intraepithelial neoplasia (PIN) lesions, leading to arrest at the stage of hyperplasia or LGPIN lesions, whereas HGPIN lesions significantly increased in *Pten*^*pc-/-*^ mice (Fig. 1h, i). Notably, the disruption of smooth muscle sheaths surrounding tumors was overtly alleviated in *Me1*-deleted *Pten*^*pc-/-*^ mice compared with that in *Pten*^*pc-/-*^ mice according to αSMA staining, indicating a decrease in the invasive capability of prostate epithelial cells (Supplementary Fig. 2e). The inhibitory effect of *Me1* deletion on prostate epithelial cell growth was confirmed by analysis of Ki-67 staining intensity, which was significantly reduced in *Me1* knockout tumors (Supplementary Fig. 2f). Furthermore, Kaplan–Meier survival analyses of the *Pten*^*pc-/-*^ and *Pten*^*pc-/-*^; *Me1*^*pc-/-*^ mice revealed that *Me1* deletion significantly extended the survival period; more than 70% of the *Pten*^*pc-/-*^*; Me1*^*pc-/-*^ mice survived, whereas 80% of the *Pten*^*pc-/-*^ mice died after 64-week (Fig. 1j). These results demonstrated the critical role of ME1 in the development of PCa in vivo. Collectively, the enrichment of L-lactate and ME1 in PCa suggests that L-lactate-regulated ME1 plays an important role in promoting PCa development.

### L-lactate is sensed by ME1 through direct binding

**Fig. 2:**
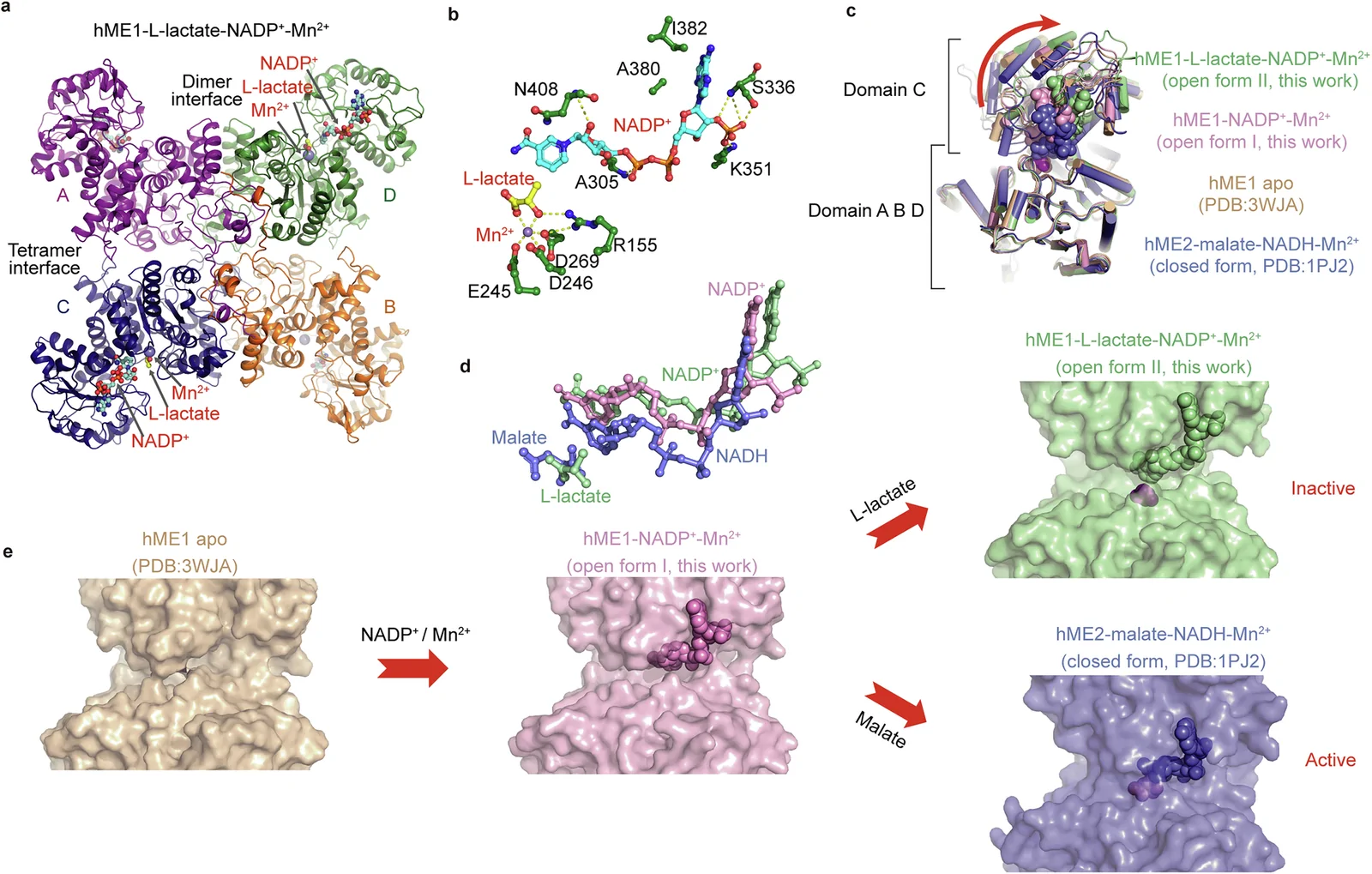
L-lactate directly binds to ME1. **a** Ribbon representation of the tetrameric hME1-L-lactate-NADP^+^-Mn^2+^ complex. Monomers A-D are colored in magenta, orange, blue and green, respectively. NADP^+^ (in cyan for carbon atoms) and L-lactate (in yellow for carbon atoms) are drawn in the ball-and-stick model, and Mn^2+^ is shown as a purple sphere. **b** The interaction details between ME1 and NADP^+^, Mn^2+^ (purple), and L-lactate at the active site region in monomer D. ME1, NADP^+^, and L-lactate are shown in green, cyan, and yellow for carbon atoms, respectively. Charge-charge and hydrogen-bonding interactions and coordination are highlighted by dashed lines in yellow. **c** Superimposition (with domains A, B, and D) of hME1 apo (PDB ID: 3WJA), hME1-NADP^+^-Mn^2+^ complex (pink), hME1-L-lactate- NADP^+^-Mn^2+^ complex (green, monomer D), and hME2-malate-NADH-Mn^2+^ complex (blue, PDB ID: 1PJ2). ME1 and ME2 are shown as ribbon representation and the small molecules as spheres. **d** Superimposition (with domains A, B, and D) of hME1 apo (PDB ID: 3WJA), hME1-NADP^+^-Mn^2+^ complex (pink), hME1-L-lactate-NADP^+^-Mn^2+^ complex (green, monomer D), and hME2-malate-NADH-Mn^2+^ complex (blue, PDB ID: 1PJ2). For clarify, ME1, ME2 and Mn^2+^ are omitted and the small molecules are drawn in the ball-and-stick model. **e** Combined surface (hME1 and hME2) and sphere (L-lactate, malate, NADH, and NADP^+^) diagrams of hME1 apo (wheat, PDB ID: 3WJA), hME1-NADP^+^-Mn^2+^ complex (pink), hME1-L-lactate-NADP^+^-Mn^2+^ complex (green, monomer D), and hME2-malate-NADH-Mn^2+^ complex (blue). L-lactate and malate are highlighted in deep purple. For clarify, Mn^2+^ is omitted in the figure

Increased L-lactate has different effects on tumor cells under different nutrient conditions and induces ME1 expression, exacerbating PCa progression. How L-lactate interacts with ME1 to perform diverse functions in tumor development remains poorly understood. We were curious whether ME1 might be a novel sensor of L-lactate by a direct physical interaction. Canonically, ME1 catalyzes the conversion of the citric acid cycle intermediate malate to pyruvate, which then consumes NADP^+^ to produce NADPH. We solved the crystal structures of human ME1 in complex with the cofactors NADP^+^ and Mn^2+^ at 2.31 Å resolution and L-lactate, NADP^+^ and Mn^2+^ at 2.03 Å resolution (Fig. 2a and Supplementary Fig. 3a, Table S1). In these structures, ME1 formed a canonical dimer-of-dimer architecture, similar to the reported structures of the ME1 and ME2 enzymes.^23–27^ Each monomer contains four structural domains (A, B, C, and D), which regulate the conformation and activity of the enzymes through rigid-body rotation.^23^ The catalytically active site was located in the cleft formed by domains B and C (Supplementary Fig. 3b), and the electron densities of L-lactate, NADP^+^ and Mn^2+^ were well defined (Supplementary Fig. 3c, d). NADP^+^ formed hydrogen bonds with Ala305, Ser336, Lys351, and Asn408. The nicotinamide ring was partially shielded by Asn408, whereas the adenine ring was positioned on the surface of the enzyme and was sandwiched between the side chains of Ala380, Ile382 and Ser336 (Fig. 2b and Supplementary Fig. 3e). L-lactate resided at the identical site of ME1 in monomers C and D (Supplementary Fig. 3d). Specifically, O1 of L-lactate formed a hydrogen bond with the side chain of Arg155 (Fig. 2b). Mn^2+^ played an essential role in stabilizing the interaction between L-lactate and ME1. O1 and O2 of L-lactate and Glu245, Asp246, and Asp269 of ME1 were ligands of the divalent cation (Fig. 2a and Supplementary Fig. 3f).^28^ Through isothermal titration calorimetry (ITC), we determined that the affinity of malate binding to ME1^WT^ was in the micromolar range (Kd = 2.41 ± 0.16 μM), whereas L-lactate bound to ME1^WT^ with millimolar affinity (Kd = 1.98 ± 0.64 mM; Supplementary Fig. 3g). According to the Human Metabolome Database (website: hmdb.ca/metabolites/HMDB000019), the cellular concentrations of malate and lactate are approximately 3 mM under normal conditions; however, under anoxic conditions, the concentrations of malate and lactate frequently decrease to approximately 0.48 mM, while the lactate concentration increases to 16 mM, yielding a molar ratio of approximately 3:100 (malate: lactate). Although malate has a greater binding affinity for ME1, the substantial accumulation of lactate under hypoxia may confer a competitive advantage for ME1 binding in cancer. In support of this hypothesis, a biotin-lactate pull-down assay confirmed the interaction between lactate and ME1 in various cancer cells (Supplementary Fig. 3h). Moreover, even at a 1:20 molar ratio (0.05 mM malate vs. 1 mM lactate), malate was largely unable to compete with lactate for ME1 binding (Supplementary Fig. 3i). Consistent with structural insights, mutation of the lactate-binding residue R155 on ME1 (ME1^R155A^) dramatically disrupted L-lactate binding (Supplementary Fig. 3g). Cellular thermal shift assays further demonstrated that L-lactate stabilized ME1^WT^, but not ME1^R155A^, in *ME1*-knockout PC3 cells (Supplementary Fig. 3j).

On the basis of previous structural studies of ME1 and ME2 in their apo and complexed forms,^23^ binding of NADP^+^ and Mn^2+^ switched the apo of ME1 to the open form I (Supplementary Fig. 4a, b). Substrate (malate) binding subsequently leads to closure of the active site (closed form) mediated by the movement of domain C toward domain B. Superimposition of the structures of ME1-L-lactate-NADP^+^-Mn^2+^, ME1-NADP^+^-Mn^2+^ (open form I), and human ME2-malate-NADH-Mn^2+^ (closed form, PDB ID: 1PJ2)^25^ complexes revealed that both L-lactate and malate occupied the same pocket in the enzymes, whereas NADP^+^ in the L-lactate-bound complex adopted a more stretched conformation (open form II) (Fig. 2c, d, Supplementary Fig. 4c). Gradual rigid-body movements of domain C were observed from the malate-NADH-Mn^2+^-bound form (ME2) to the L-lactate-NADP^+^-Mn^2+^-bound form (ME1), indicating a close-to-open transition of the enzyme (Fig. 2c, e).

The enzymatic activity of purified recombinant ME1 was inhibited by L-lactate in a dose-dependent manner (Supplementary Fig. 4d). Although L-lactate decreased M + 3 pyruvate levels in cells with normal or nutrient-depleted medium according to the results of a ^13^C_4_-malate tracing assay (Supplementary Fig. 4e, f), total cellular malate and pyruvate levels paradoxically increased upon L-lactate treatment (Supplementary Fig. 4g). These seemingly contradictory observations suggest that the cellular effects of L-lactate might involve mechanisms beyond direct ME1 enzymatic inhibition and that alternative mechanisms may be involved. The lactate-binding residue R155 appeared crucial for the canonical catalytic function of ME1, as compared with ME1^WT^, the purified recombinant ME1^R155A^ mutant exhibited inhibited in vitro enzymatic activity in terms of NADPH production (Supplementary Fig. 4h). To address the potential limitations of in vitro experiments, we performed a cellular ^13^C_4_ malate tracing assay. Notably, no significant differences in the amount of M + 3 pyruvate generation were observed between ME1^WT-^ and ME1^R155A^-expressing cells (Supplementary Fig. 4i), and the mutation likewise did not alter total cellular malate or pyruvate levels (Supplementary Fig. 4j). These findings indicate that metabolic flux rerouting or compensatory pathways may buffer the loss of ME1 catalytic function in intact cellular contexts. Collectively, these data demonstrate that, rather than modulating intrinsic enzymatic activity, ME1-R155 mediates lactate binding primarily for regulation of tumor cells function.

We observed the inhibitory effect of lactate on cell growth (Supplementary Fig. 1c). It has been reported that lactate can inhibit tumor cell growth by suppressing glycolysis.^21^ To explore whether the binding of L-lactate to ME1 influences tumor cell growth, we performed a seahorse analysis and reported that L-lactate inhibited the glycolytic capacity of ME1^WT^ cells, whereas R155A did not affect the level of glycolysis in the absence of lactate but did abrogate the inhibitory effect of lactate on glycolysis in PC3 cells (Supplementary Fig. 4k).

In summary, L-lactate competes with malate for binding to ME1, which is beneficial for sensing and relaying L-lactate signals via biological functions.

### L-lactate induces K362 deacetylation and enhances ME1 nuclear translocation

**Fig. 3:**
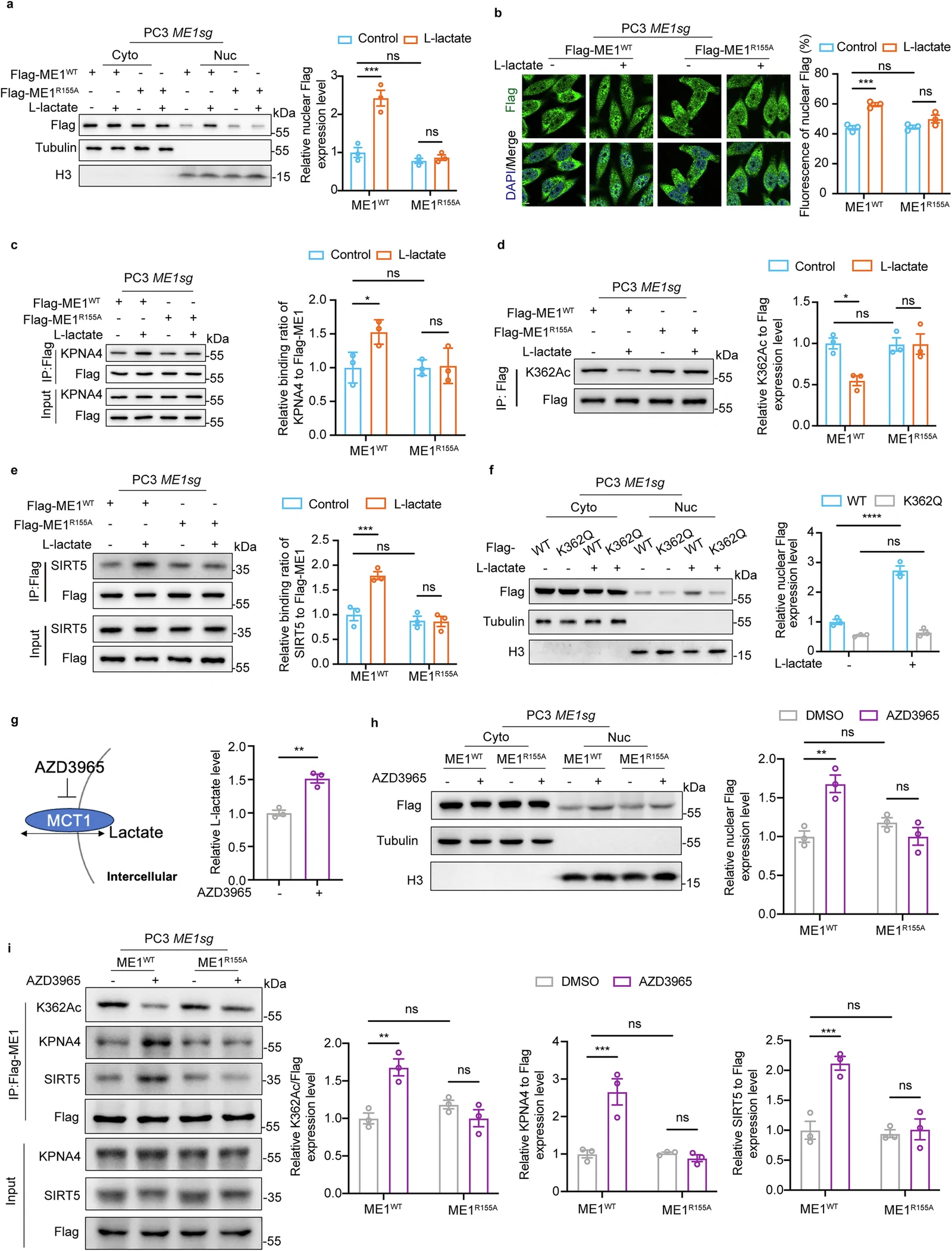
L-lactate binds to ME1 to promote ME1 nuclear translocation through inducing its K362 deacetylation. **a** ME1^R155A^ mutant blocks L-lactate-increased ME1 nuclear translocation in PC3 cells. Representative WB images (left panel) and quantification (right panel) are shown (mean ± s.e.m., Two-way ANOVA). **b** Re-expressing ME1^R155A^ mutant abolishes sodium L-lactate (10 mM)-promoted ME1 nuclear translocation in *ME1sg* PC3 cells. 10 mM NaCl treatment for the control group. Representative IF staining images (left panel) and quantification for the ratio of nuclear to total ME1 fluorescence (right panel) (mean ± s.e.m., Two-way ANOVA). Scale bar, 5 μm. **c** Sodium L-lactate (10 mM) (+) increases the interaction of ME1 with KPNA4 in *ME1sg* PC3 cells expressing ME1^WT^ but not ME1^R155A^. 10 mM NaCl treatment for the control group (-). Representative WB images (left panel) and quantification (right panel) are shown (mean ± s.e.m., Two-way ANOVA). **d** Sodium L-lactate (10 mM) decreases K362 acetylation level of ME1 through binding to ME1-R155 site. 10 mM NaCl treatment for the control group (-). Representative WB images (left panel) and quantification (right panel) are shown (mean ± s.e.m., Two-way ANOVA). **e** ME1^R155A^ mutant blocks L-lactate-promoted ME1 binding to SIRT5 in PC3 cells. Representative WB images (left panel) and quantification (right panel) are shown (mean ± s.e.m., Two-way ANOVA). **f** K362Q mutation inhibits ME1 nuclear translocation induced by 10 mM sodium L-lactate (+) as the control group (-) with 10 mM NaCl in *ME1sg* PC3 cells cultured with the normal medium (mean ± s.e.m., Two-way ANOVA). **g** monocarboxylate transporter 1 (MCT1) inhibitor AZD3965 (1 μM) increases cellular L-lactate level in PC3 cells (mean ± s.e.m., unpaired *t* test). **h** AZD3965 promotes ME1 nuclear translocation in *ME1sg* PC3 cells expressing ME1^WT^ but not ME1^R155A^. DMSO treatment for the control group (-). Representative WB images (left panel) and quantification (right panel) are shown (mean ± s.e.m., Two-way ANOVA). **i** AZD3965 decreases ME1 K362 acetylation through strengthening ME1 binding to SIRT5 and increases the interaction with KPNA4 in *ME1sg* PC3 cells expressing ME1^WT^ but not ME1^R155A^. DMSO treatment for the control group (-). Representative WB images (left panel) and quantification (right panel) are shown (mean ± s.e.m., Two-way ANOVA)

On the basis of the findings of direct binding of ME1 to L-lactate and L-lactate-inhibited PCa cell proliferation in normal medium with sufficient nutrients, we explored the exact effects of L-lactate-ME1 signaling under nutrient-replete conditions on PCa development. Intriguingly, IF analyses of PCa tissues from *Pten*^*pc-/-*^ mice revealed that cancer cells with enhanced nuclear distribution of Me1 displayed low expression levels of the cell proliferation marker Ki-67 (Supplementary Fig. 5a). In contrast, cancer cells with high expression levels of Ki-67 exhibited stronger IF staining intensity of Me1 in the cytosol (Supplementary Fig. 5a). These data suggested that ME1 translocation was linked to cell proliferation. ME1 is known to be located in the cytoplasm. However, we indeed observed nuclear accumulation of ME1 in response to sodium L-lactate treatment in both DU145 and PC3 cells (Supplementary Fig. 5b). Moreover, re-expressing ME1^R155A^ which blocks ME1 binding to L-lactate in *ME1sg* PC3 cells led to the abolishment of L-lactate-induced ME1 nuclear translocation (Fig. 3a, b). Specific soluble carrier proteins are indispensable for the transport of macromolecular cargos between the cytoplasmic and nuclear compartments. Analysis using tools online (accessed website: nls-mapper.iab.keio.ac.jp)^29,30^ shows that ME1 has a sequence containing a nuclear localization signal (NLS). In general, nucleus-localized proteins contain NLSs, which can be recognized by karyopherin α (KPNA) transporters to facilitate cytoplasmic-to-nuclear entry.^31^ To elucidate the mechanism of L-lactate-induced ME1 nuclear translocation, Flag-tagged KPNA1-6 was introduced into HEK293T cells, and only KPNA4 could bind to ME1, as shown by IP and western blot analysis (Supplementary Fig. 5c). Consistent with these findings, *KPNA4* knockout noticeably reduced the amount of nuclear ME1 (Supplementary Fig. 5d). Notably, L-lactate substantially increased the binding between ME1 and KPNA4, whereas ME1^R155A^ inhibited this effect of L-lactate (Fig. 3c).

Next, we investigated the mechanisms underlying L-lactate signaling-triggered nuclear translocation. In PC3 cells, the acetylation level of ME1 decreased in response to L-lactate (Supplementary Fig. 5e). Previous reports revealed that ME1 can be acetylated to promote tumorigenesis.^18^ On the basis of the online querying results (access website: phosphosite.org), we verified the potential acetylation sites at ME1 that were highly ranked. The acetylation level of ME1 in cells expressing various ME1^lysine (K)-to-arginine (R)^ mutants (mimicking the deacetylation of lysine) was detected. Introduction of the ME1^K362R^ mutant largely decreased the acetylation level of ME1 (Supplementary Fig. 5f). To decipher the function of K362 acetylation in ME1, we generated an antibody that specifically recognizes the acetylated K362 residue at ME1 (designated as the ‘K362Ac antibody’). A dot blotting assay was performed to characterize the specificity of the antibody. The results indicated that the K362Ac antibody preferentially detected the acetylated peptide (Supplementary Fig. 5g) and that L-lactate decreased the ME1 K362 acetylation level detected by the K362Ac antibody (Supplementary Fig. 5h). With the help of the K362Ac antibody, we found that L-lactate decreased the K362 acetylation level of ME1, which was abrogated by the introduction of ME1^R155A^ (Fig. 3d), suggesting that the L-lactate-controlled K362 acetylation level of ME1 is dependent on the interaction of L-lactate and ME1. The reversible protein acetylation reaction is catalyzed by deacetylases. Treatment with the histone deacetylase inhibitor trichostatin A (TSA) did not affect the K362 acetylation level of ME1 (Supplementary Fig. 5i). To identify the exact deacetylase of ME1 at K362, HA-tagged NAD^+^-dependent sirtuins (SIRTs), an important category of deacetylase, were individually overexpressed in HEK293T cells, and only SIRT5 effectively reduced the K362 acetylation level of ME1 (Supplementary Fig. 5j). Notably, L-lactate promoted a conspicuous interaction between ME1 and SIRT5, and this intensified interaction was restrained when the ME1 R155 residue was mutated (Fig. 3e). Lysine deacetylation of nonhistone proteins regulates diverse cellular processes.^32^ Thus, it was worthwhile to explore whether acetylation of ME1 at K362 could regulate its nuclear translocation. Notably, the K362Q mutation abolished the L-lactate-induced increase in ME1 nuclear translocation (Fig. 3f). In addition, the increase in nuclear ME1 in response to L-lactate could be negated by *SIRT5* knockdown in PC3 cells (Supplementary Fig. 5k). Moreover, L-lactate-induced ME1 K362 deacetylation promoted ME1 nuclear translocation in A549 and HCT116 cells (Supplementary Fig. 5l, m). To further explore the effect of L-lactate, we used the monocarboxylate transporter 1 (MCT1) inhibitor AZD3965 to increase the intercellular lactate level (Fig. 3g) and found that the effects of AZD3965 treatment were similar to those of L-lactate on ME1 K362 deacetylation and nuclear translocation in PC3 (Fig. 3h, i), A549, and HCT116 cells (Supplementary Fig. 5n, o). Collectively, these data reveal that L-lactate-induced K362 deacetylation contributes to ME1 nuclear translocation.

### L-lactate-promoted nuclear translocation potentiates tumor metastasis

**Fig. 4:**
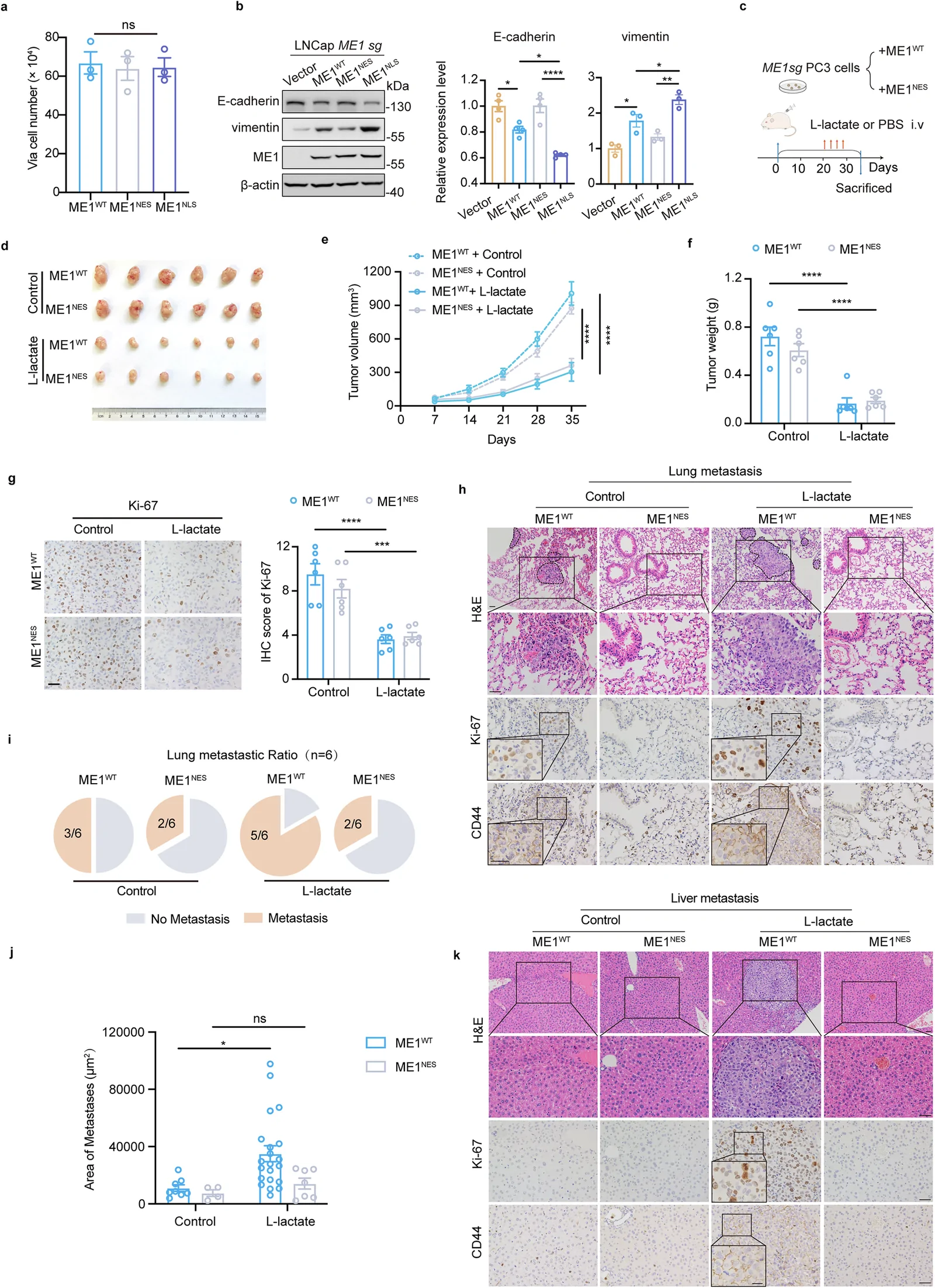
L-lactate- promoted ME1 nuclear translocation facilitates tumor metastasis. **a** Cell numbers of *ME1sg* PC3 cells expressing ME1^WT^, ME1^NES^, and ME1^NLS^ on Day 6 (mean ± s.e.m., One-way ANOVA). **b** ME1^NLS^ mutant increases epithelial-mesenchymal transition (EMT) in *ME1sg* PC3 cells compared to cells with ME1^NES^ mutant. Representative WB images (left panel) and quantification for EMT marker E-cadherin and vimentin expression level (right panel) are shown (mean ± s.e.m., One-way ANOVA). **c** Schematic of xenograft tumor model in NSG mice by injecting *ME1sg* PC3 cells expressing ME1^WT^ or ME1^NES^ subcutaneously with L-lactate treatment of 2 g/kg in tail vein or PBS as control. **d**, **e** Administration of sodium L-lactate (2 g/kg per day) by tail vein injection suppresses tumor growth in the *ME1sg* PC3 cells re-expressing ME1^WT^ or ME1^NES^ xenograft NSG mouse model. The mice from control group are treated with PBS (*n* = 6 mice for each group, mean ± s.e.m., Two-way ANOVA). **f** L-lactate inhibits tumor weight (mean ± s.e.m., Two-way ANOVA). **g** L-lactate decreases Ki-67 expression level according to IHC staining analysis. Representative images (left panel) and statistical analysis (right panel) are shown (*n* = 6, mean ± s.e.m., Two-way ANOVA). Scale bar, 40 μm. **h**–**j** L-lactate promotes lung metastases in ME1^WT^ group compared to ME1^NES^ group. Representative images of H&E and IHC staining of Ki-67 and CD44 (as a PC3 cell marker) for lung metastases (**h**) are shown. Statistical analyses for metastatic ratio (**i**) and area of metastatic foci (**j**) are shown (mean± s.e.m., Two-way ANOVA). The definite numbers of mice with metastasis in each group are shown in the bar graph (**i**). Each dot represents one metastatic foci (**j**). Scale bar, 40 μm. **k** Representative images of H&E staining of the case with liver metastasis in ME1^WT^ group administrated with sodium L-lactate and IHC staining of Ki-67 and CD44 are shown. Scale bar, 40 μm

On the basis of these findings, the effect of L-lactate-increased ME1 nuclear translocation on tumor cells was curious. There was no difference in cell growth among the ME1^WT^, NLS signal (cloned from simian virus 40)-fused wild-type ME1 (ME1^NLS^), and nuclear export signal (NES) (cloned from HIV REV protein)-fused wild-type ME1 (ME1^NES^) groups (Fig. 4a), suggesting that L-lactate-promoted ME1 nuclear translocation did not affect tumor cell proliferation. We subsequently showed that knocking out *ME1* downregulated the expression level of the zinc finger E-Box binding homeobox factor 2 (*ZEB2*) gene, which encodes an important transcription factor that drives EMT and is positively correlated with tumor aggressiveness,^33^ and upregulated the expression level of the epithelial marker *E-cadherin*, as determined by qPCR (Supplementary Fig. 6a). Western blot analyses further revealed a reduction in E-cadherin expression and an increase in vimentin protein expression in *ME1* knockout LNCap cells reexpressing ME^1NLS^ whereas overexpression of ME1^NES^ in *ME1*-deficient cells resulted in opposite effects on the expression levels of E-cadherin and vimentin (Fig. 4b). To explore how nuclear ME1 regulates ZEB2 expression levels and activates EMT, we performed anti-Flag IP combined with IP‒mass spectrometry (IP-MS) in *ME1-*knockout HEK293T cells with Flag-tagged ME1^WT^, ME1^NES^, and ME1^NLS^ (Supplementary Fig. 6b). We focused on transcription factors that potentially cooperate with nuclear ME1 to increase ZEB2 expression levels and identified HDGF as a new binding partner of nuclear ME1 (Supplementary Fig. 6b). HDGF was originally recognized as a secreted growth factor and has been reported to be involved in tumorigenesis through its participation in various biological processes, such as cell migration and EMT.^34,35^ The endogenous binding between ME1 and HDGF increased in response to L-lactate treatment (Supplementary Fig. 6c). TGF-β-Smad activity stimulates EMT in tumor cells, leading to tumor metastasis and chemotherapy resistance.^36^ Intriguingly, HDGF and L-lactate cooperatively activated Smad2/3 signaling in PC3 cells; however, this effect was abrogated upon *ME1* depletion (Supplementary Fig. 6d). Notably, nuclear ME1 promoted tumor cell migration and invasion in response to L-lactate treatment (Supplementary Fig. 6e); this effect was attributed to L-lactate binding at the R155 site (Supplementary Fig. 6f). To further determine whether L-lactate-induced nuclear translocation of ME1 regulates tumor metastasis, we constructed a *ME1sg* PC3 cell (expressing ME1^WT^ or ME1^NES^)-derived xenograft mouse model in which sodium L-lactate or PBS was administered through the tail vein and evaluated tumor growth and metastasis (Fig. 4c). As expected, compared with PBS, L-lactate suppressed xenograft tumor growth (Fig. 4d–f), as reflected by the results of the Ki-67 staining analysis of the xenograft tumor tissues (Fig. 4g). More importantly, both the lung metastatic ratio and the area were greater in the L-lactate group than in the PBS group; however, when ME1 nuclear translocation was inhibited (as indicated by the expression of ME1^NES^), the ability of L-lactate to promote xenograft tumor metastasis decreased (Fig. 4h–j). Notably, liver metastasis was observed in one mouse in the sodium L-lactate-treated ME1^WT^ group (Fig. 4k). Collectively, these findings show that L-lactate limits tumor growth and facilitates tumor metastasis by triggering ME1 nuclear translocation.

### L-lactate promotes cell survival by enhancing the interaction with LDHB under nutrient-deficient conditions

**Fig. 5:**
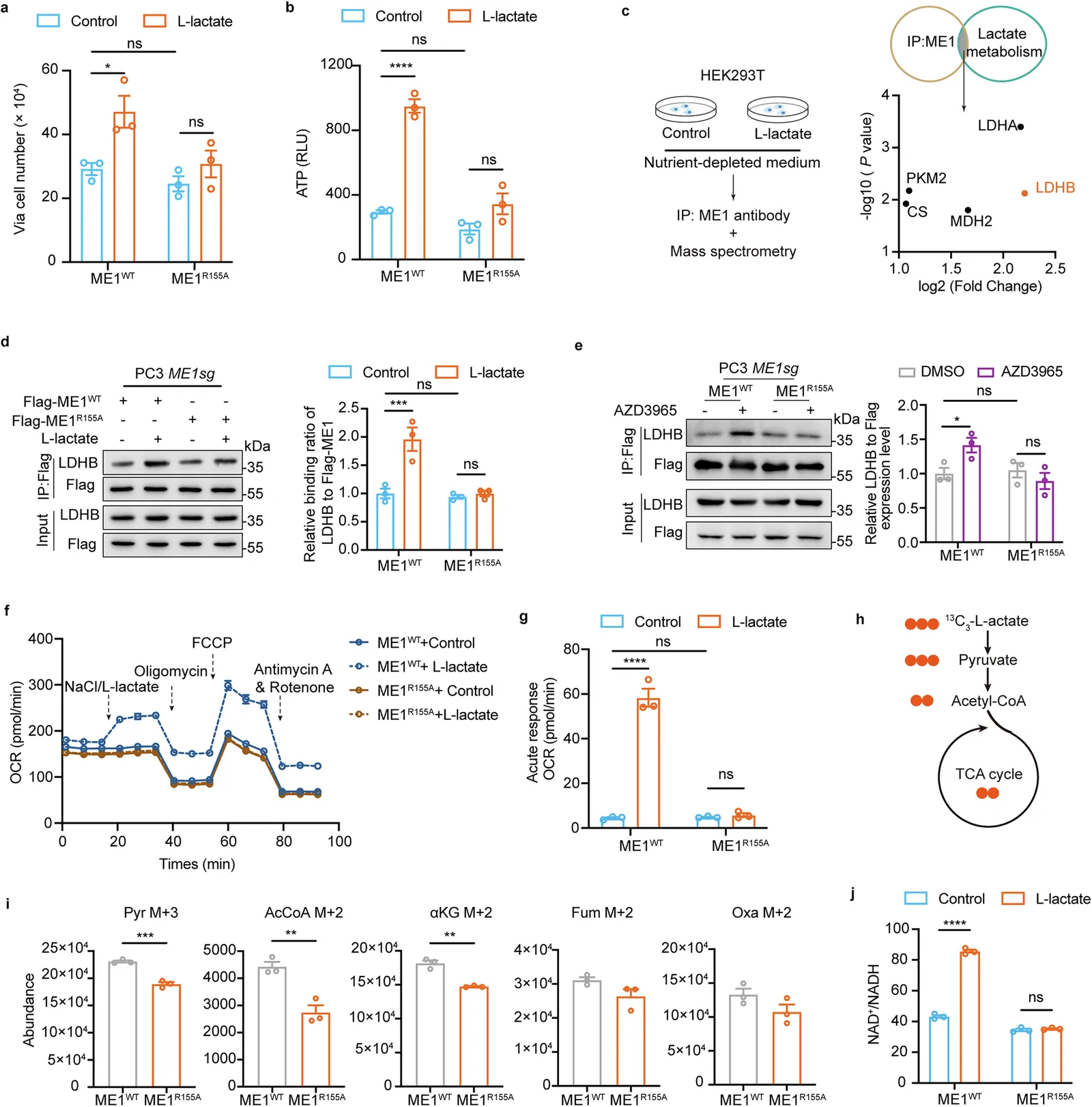
L-lactate binds to ME1 to strengthen the interaction with LDHB for cell survival under nutrients deficient condition. **a** L-lactate supports PC3 cell growth in the nutrient-depleted medium (2.5 mM glucose and 0.5 mM glutamine) dependent on binding to ME1-R155 site. Quantification for the cell numbers with 30 mM sodium L-lactate or NaCl as the control group are shown (mean ± s.e.m, Two-way ANOVA). **b** Sodium L-lactate (30 mM) increases the cellular ATP level to maintain the survival of *ME1sg* PC3 cells expressing ME1^WT^ but not ME1^R155A^ in the nutrient-depleted medium compared to the control group with NaCl (30 mM) (mean ± s.e.m., Two-way ANOVA). **c** IP-mass spectrometry shows the proteins associated with L-lactate metabolism according to fold change and *P* value in HEK293T cells treated with nutrient-depleted medium with 30 mM L-lactate or NaCl as the control group (*n* = 3, unpaired *t* test). **d** Sodium L-lactate promotes ME1 binding to LDHB in *ME1sg* PC3 cells expressing ME1^WT^ but not ME1^R155A^ in the nutrient-depleted medium compared to the control group with NaCl (30 mM). Representative WB images (left panel) and quantification (right panel) are shown (mean ± s.e.m., Two-way ANOVA). **e** AZD3965 treatment strengthens ME1 binding to LDHB in *ME1sg* PC3 cells expressing ME1^WT^ but not ME1^R155A^. DMSO treatment for the control group (-). Representative WB images (left panel) and quantification (right panel) are shown (mean ± s.e.m., Two-way ANOVA). **f**, **g** Seahorse analysis shows mitochondrial function of *ME1sg* PC3 cells expressing ME1^WT^ or ME1^R155A^ with acute L-lactate or NaCl injection (mean ± s.e.m., Two-way ANOVA). **h** A diagram for incorporation of ^13^C derived from lactate into downstream metabolites of TCA cycle in the first run. **i** Abundance of ^13^C-labeled pyruvate (Pyr), acetyl-CoA (AcCOA), α-ketoglutarate (α-KG), fumurate (Fum), and oxaloacetate (Oxa) (mean ± s.e.m., unpaired *t* test). **j** The NAD^+^/NADH ratio of ME1^WT^ or ME1^R155A^ cells with or without L-lactate treatment under nutrient-deficient condition (mean ± s.e.m., Two-way ANOVA)

In nutrient-limited medium, L-lactate supported tumor survival (Supplementary Fig. 1e) and increased ME1 expression levels (Fig. 1d); however, whether the L-lactate-ME1 complex functioned in maintaining tumor cell status under such conditions was unclear. Unlike in the ME1^WT^ group, L-lactate treatment did not increase the survival of *ME1sg* PC3, A549 or HCT116 cells expressing ME1^R155A^ (Fig. 5a and Supplementary Fig. 7a). Similarly, the survival of ME1^WT^ cells, but not that of ME1^R155A^ cells, was sustained by AZD3965 treatment (Supplementary Fig. 7b). Moreover, the ME1^R155A^ mutation decreased the L-lactate-induced increase in cellular ATP levels (Fig. 5b). Observations of the ME1-mediated effects of L-lactate on cell states in response to different nutritional stresses prompted us to define the underlying mechanism. LDHA and LDHB are indispensable for L-lactate metabolism and were both upregulated under nutrient-poor conditions (Supplementary Fig. 1f–h). To determine whether these proteins are involved in the ability of L-lactate-ME1 to regulate function, the proteins binding to ME1 in HEK293T cells were screened in nutrient-depleted medium with or without L-lactate using IP-MS experiments. Enzymes related to L-lactate metabolism were recorded on the basis of the fold change and *P* value. The results revealed that both LDHA and LDHB were potential binding partners of ME1 (Fig. 5c). Indeed, L-lactate only increased ME1 binding to LDHB but not LDHA in various cancer cells cultured with nutrient-depleted medium (Supplementary Fig. 7c, d); however, owing to disrupted binding to ME1 in ME1^R155A^ cells, L-lactate could not strengthen this interaction between ME1 and LDHB (Fig. 5d and Supplementary Fig. 7e). Similarly, AZD3965 promoted the binding of ME1^WT^, but not ME1^R155A^, to LDHB in PC3, A549, and HCT116 cells (Fig. 5e and Supplementary Fig. 7f).

Next, we investigated how the intensified interaction of ME1 with LDHB affects L-lactate-regulated cell survival in the absence of nutrients. Enzymatic activity analysis in vitro revealed that the presence of ME1 upregulated LDHB enzymatic activity (Supplementary Fig. 7g). Furthermore, the L-lactate-enhanced oxygen consumption rate (OCR) under nutrient-deficient conditions was dependent on both ME1 and LDHB according to the results of the Seahorse analysis (Supplementary Fig. 7h). In agreement with these findings, under nutrient-deficient conditions, L-lactate did not increase OCR or ATP production impaired by silencing *ME1* (Supplementary Fig. 7h, i), and ME1 restoration improved cell states upon L-lactate treatment but not in *LDHB* knockdown cells (Supplementary Fig. 7h, i). These data indicate that LDHB is the key enzyme involved in the L-lactate-ME1 metabolic signaling axis when tumor cells are exposed to a nutrient-deficient microenvironment.

More importantly, the R155A mutation blocked the ability of L-lactate to increase mitochondrial function (Fig. 5f, g). Isotope tracing revealed that ^13^C_3_ of L-lactate directly promoted the metabolism of the TCA cycle in ME1^WT^ cells (Fig. 5h, i); consequently, the NAD^+^/NADH ratio was elevated in ME1^WT^ cells treated with L-latacte, which is critical for ATP production (Fig. 5j). Interestingly, given that the ME1-R155A mutation blocks L-lactate binding, this stimulatory effect of L-lactate on mitochondrial function was abolished (Fig. 5h–j).

### ME1^R155A^ mutation obstructs the favorable effects of L-lactate on tumor progression in vivo

**Fig. 6:**
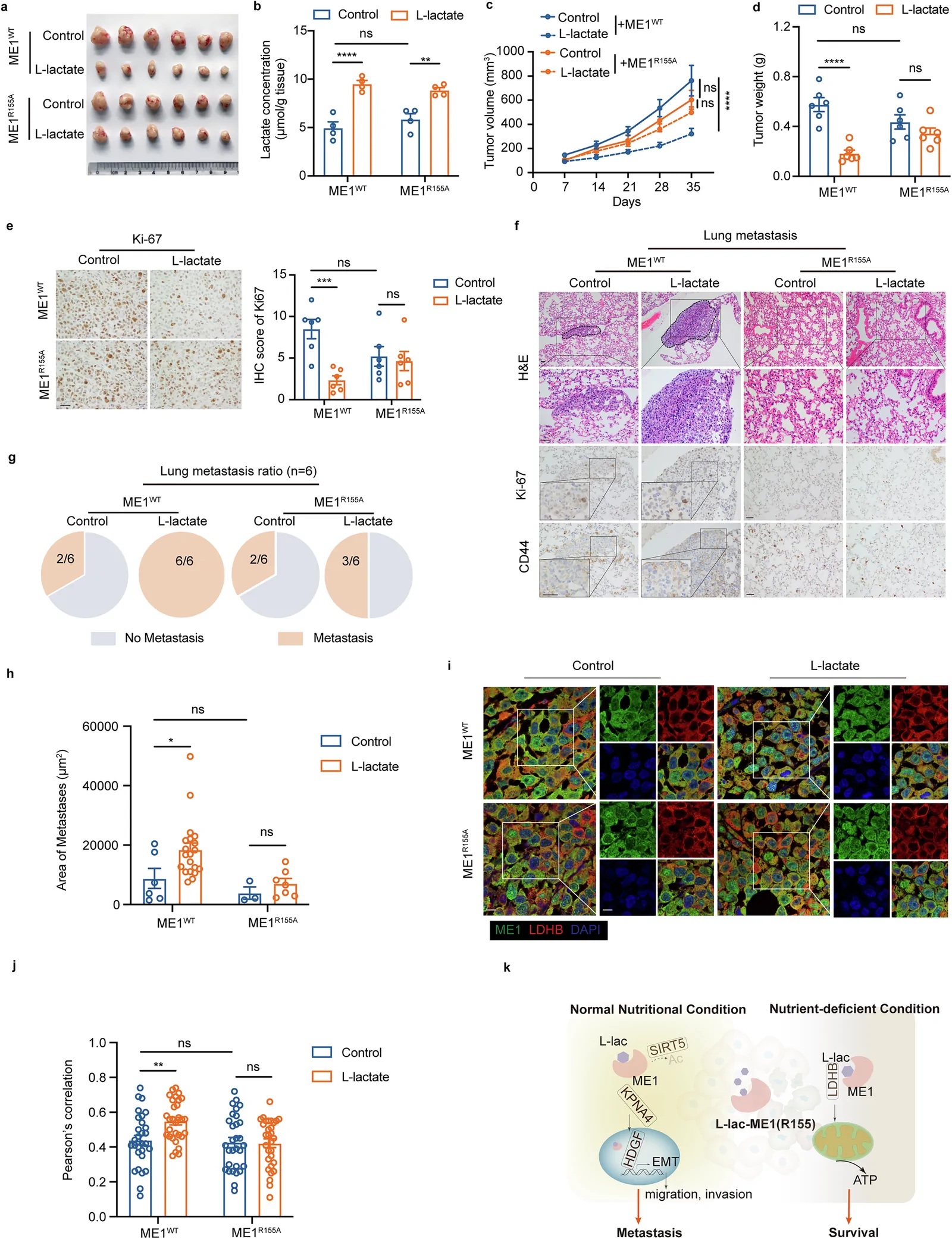
ME1^R155A^ mutation abolishes L-lactate- induced tumor metastasis and -intensified interaction of ME1 with LDHB. **a** Administration of L-lactate by tail vein injection (2 g/kg per day) suppresses tumor growth in the *ME1sg* PC3 cells expressing ME1^WT^ or ME1^R155A^ xenograft NSG mouse model compared to the control group with PBS (*n* = 6 mice for each group). **b** Lactate concentration in ME1^WT^ or ME1^R155A^ xenograft tumor tissue (mean ± s.e.m., Two-way ANOVA). **c** ME1^WT^ or ME1^R155A^ xenograft tumor growth with L-lactate treatment or PBS (mean ± s.e.m., Two-way ANOVA). **d** L-lactate inhibits the xenograft tumor weight in the ME1^WT^ group but not ME1^R155A^ group (mean ± s.e.m., Two-way ANOVA). **e** L-lactate decreases Ki-67 expression level in the ME1^WT^ group but not ME1^R155A^ group according to IHC staining analysis. Representative images (left panel) and statistical analysis (right panel) are shown (*n* = 6, mean ± s.e.m., Two-way ANOVA). Scale bar, 40 μm. **f**–**h** L-lactate significantly promotes lung metastases in the ME1^WT^ group compared to ME1^R155A^ group. Representative images of H&E and IHC staining of Ki-67 and CD44 (as a PC3 cell marker) for lung metastases (**f**) are shown. Statistical analyses for metastatic ratio (**g**) and area of metastatic foci (**h**) are shown (mean ± s.e.m., Two-way ANOVA). The definite numbers of mice with metastasis in each group are shown in the bar graph (**g**). Each dot represents one metastatic foci (**h**). Scale bar, 40 μm. **i**, **j** R155A mutation abolishes L-lactate-promoted colocalization of ME1 with LDHB. Representative images for ME1 and LDHB staining are shown (**i**). Quantification for Pearson’s correlation of ME1 with LDHB (**j**) are shown. Five independent views for each sample are calculated (*n* = 6, mean ± s.e.m., Two-way ANOVA). Scale bar, 10 μm. **k** Schematic of Lactate-ME1 axis signaling in the tumor progress

L-lactate sensed by ME1 increases ME1 nuclear translocation and intensifies the interaction between ME1 and LDHB to support tumor cell survival under different nutritional conditions, demonstrating that the direct binding of L-lactate to the ME1 R155 residue is fundamentally important for the diverse effects of L-lactate-ME1 axis signaling. Thus, we constructed a xenograft *ME1sg* PC3 cell (reexpressing ME1^WT^ or ME1^R155A^)-derived NSG tumor model to verify the influence of L-lactate-ME1 signaling on tumor progression in vivo (Fig. 6a). While intravenous administration of sodium L-lactate increased the lactate concentration in tumor tissue, it suppressed tumor growth and tumor weight compared with those in the ME1^WT^ group, and these inhibitory effects were negated in the ME1^R155A^ group (Fig. 6b–d). Consistently, IHC analyses of Ki-67 staining revealed that L-lactate-mediated inhibition of Ki-67 expression was suppressed by the introduction of the ME1^R155A^ mutation (Fig. 6e). Importantly, L-lactate significantly promoted lung tumor metastasis in the ME1^WT^ group (Fig. 6f–h). Compared with the ME1^WT^ group, the ME1^R155A^ mutant group had lower L-lactate-strengthened tumor metastasis in terms of both the ratio and the area of tumor lung metastasis (Fig. 6f–h). In addition, the colocalization of ME1 with LDHB was increased in the ME1WT group but not in the ME1R155A group under L-lactate treatment (Fig. 6i, j).

To further verify the effect of L-lactate-ME1 on tumor progression, we used the MCT1 inhibitor AZD3965 to increase intracellular lactate levels in a xenograft *ME1sg* PC3 or A549 cell (reexpressing ME1^WT^ or ME1^R155A^)-derived NSG tumor model (Supplementary Figs. 8 and 9). When the xenograft tumors grew to approximately 100 μm^3^, the mice from each group (ME1^WT^ or ME1^R155A^) were randomly divided into two groups and orally administered AZD3965 or vehicle. While AZD3965 increased lactate levels in tumor tissue, it inhibited ME1^WT^ tumor growth but had no effect on the ME1^R155A^ group (Supplementary Figs. 8a–d and 9a–d). Consistent with these findings, the results of the IHC analysis of Ki-67 revealed that AZD3965 inhibited Ki-67 expression in the ME1^WT^ group, but this effect was blocked in the ME1^R155A^ mutant group (Supplementary Figs. 8e and 9e). Intriguingly, although AZD3965 had no effect on the ratio of metastasis (3/6 for each group), it significantly increased the lesion area of metastatic tumors (Supplementary Figs. 8f and 9f) and the colocalization of ME1 with LDHB in tumor tissues from ME1^WT^ mice but not in those from ME1^R155A^ mice (Supplementary Figs. 8g and 9g).

In summary, our in vitro evidence revealed that the diverse L-lactate-promoted functions of PCa cells are dependent on the binding of L-lactate to the ME1 R155 residue and that the L-lactate-ME1 axis plays a pivotal role in the metabolic adaptation of PCa cells to various nutritional microenvironments (Fig. 6k).

## Discussion

The metabolic landscape of solid tumors is characterized by profound spatial and temporal heterogeneity, wherein cancer cells residing in distinct microenvironmental niches or progressing through different stages of tumorigenesis exhibit distinct metabolic phenotypes. This metabolic plasticity represents a critical determinant of tumor behavior and therapeutic resistance. In this study, we identify ME1 as a direct intracellular sensor of L-lactate that functions as a molecular switch to dictate alternative cellular fates contingent upon nutritional availability. Rather than operating through a linear cascade, lactate-bound ME1 engages in mutually exclusive functional programs that enable tumor cells to either disseminate when nutrients are abundant or persist under metabolic stress.

The physiological relevance of the lactate-ME1 axis is supported by extensive evidence demonstrating that lactate levels are highly heterogeneous within tumor tissues. Various technologies have revealed that intratumor lactate levels are highly variable. Mane et al. mapped the spatially heterogeneous distribution of whole-tumor lactate concentrations using the ¹H MRSI and demonstrated that intratumor lactate concentrations can range from 0 to >40 μmol/g.^6^ Specifically, in prostate cancer, the levels of intratumor lactate ranged from 3.3 to 20.8 mM, with whole-tumor lactate concentrations calculated to be approximately 7.9 mM.^8^ These studies provide a foundation for spatially distinct lactate-ME1 signaling within tumor microenvironments. In this study, we employed an in vitro concentration gradient ranging from 0 to 40 mM to recapitulate this physiological heterogeneity, which was designed on the basis of clinical evidence encompassing normal physiological serum lactate in healthy individuals (1.5–3 mM)^6,37^ and substantially elevated levels observed within tumor tissues (10–50 mM).^6^ Although the direct spatial correlation between lactate levels and ME1 function in vivo remains to be fully characterized, the concentration ranges employed in our experiments mirror the documented heterogeneity of lactate distribution in clinical tumors, supporting the physiological relevance of lactate-ME1 axis function in tumor development.

Under conditions of nutritional sufficiency, L-lactate binding to ME1 initiates a signaling axis that culminates in enhanced metastatic capability. We revealed that lactate sensing triggers SIRT5-mediated deacetylation of ME1 at lysine 362, a posttranslational modification that is linked to nuclear translocation. This deacetylation event may license ME1 for KPNA4-dependent nuclear import, potentially repositioning the enzyme from its canonical cytosolic location to the nuclear compartment. Although ME1 predominantly resides in the cytosol under steady-state conditions, the physiological significance of minor nuclear pools of metabolic enzymes is well established in the contemporary literature. This “moonlighting” paradigm illustrates that subcellular compartmentalization determines functional switching, wherein even a small fraction of nuclear translocation could exert disproportionate regulatory effects distinct from canonical metabolic roles.^38^ For instance, K433 acetylation of PKM2 controls the switch of PKM2 from a cytoplasmic metabolite kinase to a nuclear protein kinase^,^^39^ and it is translocated to the nucleus upon EGFR/ERK signaling to act as a transcriptional coactivator for HIF-1α and β-catenin to regulate glycolytic gene expression.^40^ In addition, PGK1 is translocated to the mitochondria or nucleus upon hypoxia or oncogenic signaling to function as a protein kinase independent of its glycolytic role,^41^ and the nuclear localization of FBP1 enables it to serve as a histone H3 phosphatase to suppresse gene transcription regardless of its cytosolic abundance.^42^ Consistent with this paradigm, our study revealed that nuclear ME1 has noncanonical functions distinct from its cytosolic metabolic role in NADPH production. The nuclear translocation of ME1 likely occurs in specific physiological contexts, such as in cells with limited proliferative capacity or those experiencing metabolic stress, where discrete nuclear signaling is required to coordinate transcriptional reprogramming toward metastasis. Within the nucleus, ME1 engages with HDGF, potentially forming a regulatory complex that may contribute to the transcriptional programming of the EMT. This molecular circuitry provides a mechanistic framework for how nutritional signals are transduced into pro-invasive cellular states when the microenvironment supports biosynthetic demands, independent of the bulk cytosolic enzyme pool. Additionally, our findings revealed that both pharmacologic accumulation of lactate (via AZD3965-mediated inhibition of lactate efflux) and blockade of lactate production (via 2-DG) (Data S1 in the Supplementary Material) modulate cell migration in an ME1-dependent manner. This mechanistic specificity has important therapeutic implications, positioning the lactate–ME1 axis as a promising target for antimetastatic intervention.

The lactate-mediated reduction in primary tumor burden coupled with enhanced metastatic dissemination may reflect several nonmutually exclusive mechanisms. Metastatic dissemination often precedes clinically detectable primary tumor growth or occurs early during treatment.^43^ If lactate-induced ME1 nuclear translocation follows disseminated tumor cell (DTC) seeding, primary regression may simply unmask preexisting micrometastases. Alternatively, primary tumors may systemically suppress distant DTCs by circulating inhibitors or immune factors; their shrinkage could release dormant metastases from inhibitory control.^44^ Additionally, metabolic stress may select for EMT/cancer stem cell clones with attenuated primary proliferation but heightened metastatic capacity.^45,46^ These scenarios decouple primary burden from metastatic potential, highlighting the need for temporally staged strategies that simultaneously address primary regression and metastatic dormancy. Lineage-tracing and temporal genetic models will be needed to determine when lactate-ME1 signaling critically influences metastatic fate.

In stark contrast, when tumor cells encounter nutrient scarcity, lactate-activated ME1 orchestrates an alternative survival program. Under these restrictive conditions, ME1 recruits LDHB to form a functional complex that enhances mitochondrial respiratory capacity. This metabolic rewiring enables cells to bypass glycolytic constraints and sustain ATP production through oxidative phosphorylation, thereby preserving viability during periods of metabolic crisis. Notably, this represents a distinct functional outcome of the same initial stimulus—lactate binding to ME1—yet channels the metabolic enzyme toward cytoprotection rather than nuclear signaling. While classical lactate oxidation occurs through MCT1-mediated import and the mitochondrial lactate oxidation complex,^47,48^ our findings suggest that ME1 acts as a context-dependent alternative or complementary node, although the quantitative contribution of the ME1–LDHB complex relative to canonical pathways requires further investigation. Lactate-derived lactyl-CoA can induce epigenetic modifications that upregulate OXPHOS gene expression.^12^ Residual lactate responsiveness in ME1-R155A-mutant cells indicates the presence of parallel ME1-independent bioenergetic pathways.

The bifurcated functional outcomes described above necessitate careful consideration of the mechanistic relationship between the enzymatic activity of ME1 and its signaling functions. We acknowledge that the R155A mutation employed in our studies does not achieve complete genetic separation of lactate binding from catalytic activity, as Arg155 contributes to both substrate coordination and catalysis. While an ideal “catalytically dead but binding-competent” mutant would provide cleaner experimental resolution, such constructs are theoretically ideal but experimentally challenging for ME1. The ME1 active site utilizes overlapping residues for substrate coordination and catalysis; lactate binding likely induces conformational changes similar to coupling of catalytic substrate, and complete catalytic inactivation may perturb these structural transitions. Furthermore, even if successfully generated, catalytically dead ME1 might trigger metabolic stress responses that confound the interpretation of signaling mechanisms. Nevertheless, two lines of evidence support the interpretation that R155 mediates signaling primarily through lactate binding rather than catalytic activity: (i) stable isotope tracing demonstrates that R155A does not impair cellular malate-to-pyruvate flux, indicating that the mutation signaling defect is not secondary to metabolic disturbance; and (ii) pyruvate supplementation fails to recapitulate lactate-induced signaling (data not shown), excluding the catalytic product as the signaling mediator. These findings align with the emerging paradigm of metabolic enzyme “moonlighting,” where catalytic and regulatory functions can involve overlapping structural elements yet operate through distinct mechanisms.

Collectively, ME1 functions as a metabolic rheostat that interprets lactate to direct mutually exclusive programs: prometastatic nuclear signaling under nutrient sufficiency conditions versus cytoprotective mitochondrial adaptation under scarcity conditions. This binary, state-dependent switch enables dynamic resource allocation between dissemination and survival without obligatory sequential progression. Our data establish L-lactate as a spatiotemporal coordinator of tumor malignancy through ME1 hijacking, conferring metabolic flexibility-invasive expansion when nutrients permit, dormancy and persistence when they dwindle. Therefore, therapeutic targeting of lactate sensing must account for the microenvironmental nutrient context to avoid inadvertently selecting for a survival-oriented phenotype. Elucidating these context-dependent bifurcations may reveal vulnerabilities in the metabolic plasticity driving tumor progression and therapeutic resistance.

## Materials and methods

### Cell lines and cell culture

DU145, 22RV1, PC3, A549, and HCT116 cell lines were acquired from the ATCC. DU145, A549, and HCT116 cells were cultured in Dulbecco’s modified Eagle’s medium (DMEM; Invitrogen, Thermo Fisher Scientific), and 22RV1 and PC3 cells were cultured in 1640 medium (Invitrogen, Thermo Fisher Scientific) supplemented with 10% fetal bovine serum (Gibco, Thermo Fisher Scientific), penicillin, and streptomycin at 37 °C. In nutrient-depleted medium, the amounts of glucose, glutamine, and pyruvate were reduced to the concentrations indicated in the Fig. legends. Upon L-lactate treatment, to exclude the effect of osmotic pressure, cell culture media were supplemented with different concentrations of NaCl, and then, the corresponding concentrations of sodium L-lactate (a pH-neutral form of L-lactate) and/or NaCl were added to obtain equal amounts of Na^+^ in the final culture media, which were the same as those in normal DMEM or RPMI 1640 culture medium. The pH value of the customized culture medium was approximately 7.8.

### Stable cell pool generation and transient transfection

Retroviral or lentiviral expression systems were used to generate stable cell lines with overexpression, knockout, or knockdown. To generate stable *ME1* knockout cell pools, sgRNAs targeting *ME1* were constructed, and the oligos were subsequently cloned and inserted into the pX459 vector. After transient transfection using polyethylenimine (PEI), single-cell clones were selected and verified by western blot analysis. To generate *ME1* knockout and rescue stable cell pools, Flag-tagged human wild-type (WT), R155A, K362Q, or K362R ME1 was subsequently cloned and inserted into the pQCHIX vector (ME1^WT^, ME1^R155A^, ME1^K362Q^, and ME1^K362R^). Flag-tagged ME1^NLS^ was cloned by insertion of a classical NLS signal (identified through simian virus 40) fused with ME1. Flag-tagged ME1^NES^ was cloned by insertion of a classical nuclear export signal (NES) (identified in the HIV REV protein) fused with ME1. The sgRNA targeting *KPNA4* was inserted into the lentiCRISPRv2 vector to generate stable *KPNA4* knockout cells. shRNA targeting *LDHB* was inserted into a pLKO shRNA vector to generate stable *LDHB* knockdown cells. In brief, virus particles were produced in HEK293T cells using PEI transfection. After filtration with a Millex-HV sterile 0.45 μm filter (Merck Millipore) and titration, viruses were added to cells in the presence of polybrene (7.5 μg/ml). The medium was replaced 24–48 h after infection, after which the cells were selected with puromycin (2 μg/ml) or hygromycin B (200 μg/ml) for 1–2 weeks. Protein overexpression or knockdown efficiency was confirmed by western blot.

The sequences mentioned above are listed as follows: *ME1*-sg1: TATAGAGCCAATTTACCCAC; *ME1*-sg2: ACACCTGATTGTGATGGCCT; NLS: CCTAAGAAGAAGAGGAAGGTT; NES: CTTCAGCTACCACCGCTTGAGAGACTTACTCTT; sg*KPNA4*-1: GGTTCCTCTGCTCAGCCACC; sg*KPNA4*-2*:* GCTGTGGGCAACATTGTTAC; sg*SIRT5*-1: 5’-CACCGGCTGGGAAATCAATCGACTT-3; and sg*SIRT5*-1: 5’-GGCGCTCCGGACCTGAGCCA-3’.

*shLDHB*-1: AAGATTGTAGTGGTAACTGCA; *shLDHB*-2: GCAGCTGACTTTGTCTTCT.

### Cell lysis, coimmunoprecipitation (Co-IP), nuclear isolation, biotin-lactate pull-down and western blot

Cells were lysed in 0.3% ice-cold NP-40 buffer (50 mM Tris-HCl (pH 7.4), 150 mM NaCl and 0.3% NP-40) containing protease inhibitors (1 mM phenylmethylsulfonyl fluoride, 1 μg/ml aprotinin, 1 μg/ml leupeptin, 1 μg/ml pepstatin, 1 mM Na_3_VO_4_, 1 mM NaF, 30 μM TSA and 15 μM NAM). For Co-IP of endogenous proteins, whole-cell lysates were incubated with the corresponding antibody and protein G-Agarose beads (Roche, #11243233001) overnight at 4 °C. The immunoprecipitates were subsequently washed three times with lysis buffer, resuspended in loading buffer, boiled at 95 °C for 5 min and analyzed by western blot according to standard protocols. To isolate the nuclei, a commercial nuclear and cytoplasmic protein extraction kit (Beyotime, Cat# P0028) was used. In accordance with the manufacturer’s instructions, the cells were washed with PBS in 10 cm dishes and scraped in 500 μL of cytosolic lysis buffer A supplemented with PMSF per dish. After being collected in tubes, the cells were quickly vortexed for 10 s and dissociated on ice for 15 min. After 5 s of vortexing and centrifugation at 14,000 × *g* and 4 °C, the supernatant was preserved as cytoplasmic protein, and the nuclei were collected as the pellet at the bottom. After three washes, the pellets from each dish were incubated in 50 μL of lysis buffer containing PMSF for 1 h, after which they were repeatedly frozen and thawed in liquid nitrogen three times. The nuclear protein was obtained after centrifugation. Both cytosolic and nuclear proteins were boiled in loading buffer for western blot analysis. For the biotin-lactate pull-down assay, streptavidin beads (CST, Cat #3419) were incubated with biotin or biotin-lactate in PBS for 1 h at room temperature. After being washed with PBS, the beads were incubated with cell lysates overnight at 4 °C with rotation. On the second day, the beads were washed and boiled for analysis by SDS‒PAGE. The blots were exposed and documented using an LAS 4000 or Amersham Imager 600. The western blotting results were quantified using ImageJ software.

### Antibodies

Primary antibodies against ME1 (rabbit, Proteintech, 16619-1-AP), ME2 (rabbit, Abcam, ab248127), ME3 (rabbit, Abcam, ab232359), LDHA (rabbit, Proteintech, 19987-1-AP), LDHB (rabbit, Proteintech, 2867), PKM2 (rabbit, Cell Signaling Technology, 4053), CS (rabbit, Cell Signaling Technology, 14309), GLS (rabbit, Proteintech, 12855-1-AP), KPNA4 (rabbit, Proteintech, 12463-1-AP), SIRT5 (rabbit, Proteintech, 15122-1-AP), Flag (mouse, Proteintech, 20543-1-AP), Panacetylation (mouse, PTM-Biolab, PTM-101), pSmad 2/3 (rabbit, Cell Signaling Technology, 8828), HDGF (rabbit, Abcam, ab128921), E-cadherin (rabbit, Cell Signaling Technology, 3195), Vimentin (rabbit, Cell Signaling Technology, 5741), and β-actin (mouse, Proteintech, 66009-1-Ig) were used for western blotting. Secondary antibodies against rabbit (Jackson ImmunoRes, 211-032-171) and mouse (Thermo Fisher, 31430) proteins were used for western blotting. Antibodies specific to ME1 K362Ac were prepared commercially at Shanghai Genomic, Inc., and the synthesized acetylated peptide (FAHEHEEM**K(Ac)**NLEAI) was used as an antigen to immunize rabbits. Primary antibodies against SMAα (rabbit, Abcam, ab5694), Ki67 (rabbit, Abcam, ab15580), and CD44 (rat, Thermo Fisher, 14-0441-82) were used for immunohistochemistry.

### Quantitative real-time PCR

Total RNA was isolated from cells by TRIzol and reverse transcribed to cDNA (Takara), followed by amplification by quantitative real-time PCR (qPCR) with gene-specific primers and SYBR Premix Ex-Taq (Takara). PCRs were performed in triplicate, and relative mRNA levels were calculated by the comparative CT method using *Actb* as a control.

The sequences of primers used for qPCR were as follows: qPCR-*ME1*-F, 5′-ACGAATTCATGGAGGCAGTT-3′; qPCR-*ME1*-R, 5′-GGAGACGAAATGCATTCACA-3′; qPCR-*ME2*-F, 5′-ATGTTGTCCCGGTTAAGAGTAGT-3′; qPCR-*ME2*-R, 5′-ACCAAGCATTTGTCGTTCTTGT-3′; qPCR-*ME3*-F, 5′-TGAAGAAGCGCGGATACGATG-3′; qPCR-*ME3*-R, 5′-GAAAGCAGGGCGGGATTAGG-3′; qPCR-*LDHA*-F, 5′-GGAGATCCATCATCTCTCCC-3′; qPCR-*LDHA*-R, 5′-GGCCTGTGCCATCAGTATCT-3′; qPCR-*LDHB*-F: 5′-CCTCAGATCGTCAAGTACAGTCC-3′; qPCR-*LDHB*-R: 5′-ATCACGCGGTGTTTGGGTAAT-3′; and qPCR-*PKM2*-F, 5′-GTCTGAATGAAGGCAGTCCC-3′; and qPCR-*PKM2*-R, 5′-TCCGGATCTCTTCGTCTTTG-3′; and qPCR-*HK2*-F, 5′-AGCCCTTTCTCCATCTCCTT-3′; qPCR-*HK2*-R, 5′-AACCATGACCAAGTGCAGAA-3′; qPCR-*GLS*-F, 5′-GCAAATCTTCGAAGTGCAGAC-3′; qPCR-*GLS*-R, 5′-AAAAACTTGATCCTCGAAGAGA-3′; qPCR-*CS*-F, 5′-TGCTTCCTCCACGAATTTGAAA-3′; qPCR-*CS*-R, 5′-CCACCATACATCATGTCCACAG-3′; qPCR-*ACTB*-F, 5′-GCACAGAGCCTCGCCTT-3′; qPCR-*ACTB*-R, 5′-GTTGTCGACGACGAGCG-3′.

### Liquid chromatography‒mass spectrometry metabolite measurement

To extract metabolites from tissue samples, tissue samples were first weighed (approximately 20 mg each sample) and ground using a porcelain mortar. The resulting powder was resuspended in 800 ml of ice-cold 80% methanol and 20% ddH_2_O. The samples were vigorously vortexed and placed in liquid nitrogen for 10 min to freeze and then thawed on ice for 10 min. The freeze‒thaw cycle was repeated twice. The samples were subsequently centrifuged at 13,000 r.p.m for 15 min to pellet the cell debris, lipids and proteins. The supernatant was evaporated, and the resulting metabolites were resuspended for MS analysis. The metabolites were normalized on the basis of tissue weight.

### Isotope tracing with ¹³C₄-malate

Cells were cultured overnight in RPMI 1640 (12.5 mM glucose, 2 mM glutamine) or nutrient-depleted RPMI 1640 (2.5 mM glucose, 0.4 mM glutamine). Prior to the experiments, the cells were washed with PBS and then incubated with relatively fresh medium containing 0.5 mM ¹³C₄-malate (Cambridge Isotope Laboratories, Inc., CLM-8065). Trace labeling was performed for 2 h. For the lactate supplementation experiments, sodium L-lactate was added to the medium simultaneously with the initiation of ¹³C₄-malate labeling. Metabolites were extracted using 80% methanol at −80 °C and analyzed by LC‒MS/MS. The amount of M + 3 pyruvate was used to evaluate the conversion of M + 4 malate under different conditions.

### Isotope tracing with ¹³C_3_-lactate

Cells were cultured overnight in nutrient-depleted RPMI 1640 (2.5 mM glucose, 0.4 mM glutamine). Prior to the experiments, the cells were washed with PBS and then incubated with relatively fresh medium containing 10 mM ¹³C_3_-lactate (Cambridge Isotope Laboratories, Inc., CLM-1579). Trace labeling was performed for 4 h. Metabolites were extracted using 80% methanol at −80 °C and analyzed by LC‒MS/MS. The amount of M + 2 metabolites in the TCA cycle was used to evaluate the conversion of M + 3 lactate into the TCA cycle.

### Recombinant ME1 expression and purification

Human ME1 (UniProt ID: P48163.1) was cloned and inserted into a modified pET32a or pET28a vector, with the obtained protein containing a Trx-His_6_ or His_6_ tag followed by a Prescission or thrombin protease-cutting site in its N-terminus, respectively.^49^ Human LDHB (UniProt ID: P07195) was cloned and inserted into a pET28a vector with the resulting protein containing a His_6_ tag in its N-terminus. Recombinant proteins were expressed in *Escherichia coli* BL21 (DE3) host cells (New England Biolabs) in LB medium at 16 °C for 16 h and then harvested and purified using a Ni^2+^-NTA agarose affinity column followed by size-exclusion chromatography (HiLoad 26/600 Superdex 75/200 pg columns on an AKTA FPLC system; GE Healthcare) with buffer A containing 30 mM Tris-HCl (pH 7.4), 500 mM NaCl and 2 mM DTT.

### In vitro enzyme activity assay

For the ME1 enzymatic activity assay, the purified proteins were dissolved in reaction buffer supplemented with 30 mM Tris-HCl (pH 7.4), 300 mM NaCl, 0.5–2 mM L-malic acid, 0.3 mM NADP^+^, and 5.0 mM manganese chloride (MnCl_2_). The reactions were started immediately after the addition of purified ME1 proteins, followed by shaking and measurement of the increase in fluorescence (Ex. 350 nm, Em. 470 nm) for NADPH generation using a Fluostar Optima (Synergy™ HTX Multi-Mode Microplate Reader). For the LDHB activity assay, purified proteins were dissolved in reaction buffer containing 30 mM Tris-HCl (pH 7.4), 300 mM NaCl, 3 mM L-lactate, 0.3 mM NAD^+^, and 5.0 mM manganese chloride (MnCl_2_) with or without the addition of purified ME1 proteins, after which the increase in fluorescence was measured by shaking (Ex. 350 nm, Em. 470 nm) for NADH generation. For each set of experiments, a background control was run without L-malate or L-lactate as the substrate. Enzyme activity was calculated by subtracting the value of the blank control. The obtained curves of absorbance versus time were normalized to the protein content using a BCA protein kit (Bio-Rad).

### Protein preparation for crystallization

For crystallization, the N-terminal Trx-tagged fragments of recombinant ME1 were cleaved by digesting the fusion proteins with Prescission protease (Sigma, GE27-0843-01) at 4 °C, after which the fragments were purified on a Ni^2+^-NTA agarose affinity column and then stored in 30 mM Tris-HCl (pH 7.4) with 2 mM DTT.

### Protein crystallization and X-ray data collection

Freshly purified human ME1 was concentrated to 6 mg/ml in buffer containing 30 mM Tris-HCl (pH 7.4), 2.5 mM NADP^+^, 5 mM MnCl_2_, 2 mM DTT, and with or without 40 mM L-lactate. Crystals of the ME1-L-lactate-NADP^+^-Mn^2+^ complex were grown by the hanging-drop vapor diffusion method at 16 °C in a reservoir solution containing 100 mM imidazole (pH 8.0) and 12% (w/v) polyethyleneglycol 8000 (PEG8K), whereas crystals of the ME1-NADP^+^-Mn^2+^ complex were grown in a reservoir solution containing 100 mM HEPEs (pH 7.5), 10% (w/v) polyethyleneglycol 3350 and 150 mM L-proline. The crystals were then soaked in crystallization solution containing 25% glycerol for cryoprotection. The diffraction data of the ME1-L-lactate-NADP^+^-Mn^2+^ crystals were collected at the Shanghai Synchrotron Radiation Facility (SSRF) beamline BL18U1 in China at a wavelength of 0.9792 Å and then processed and scaled using HKL3000. The diffraction data of the ME1-NADP^+^-Mn^2+^ crystals were collected at the SSRF beamline BL17U1 in China at a wavelength of 0.9792 Å and then processed and scaled using XDS. The phase problem of ME1 structures was solved by molecular replacement using the program Phaser MR with the human c-NADP-ME (ME1) structure (PDB ID: 3WJA [http://www1.rcsb.org/structure/3WJA]) as the search model against the respective datasets. The initial model was further rebuilt, adjusted manually with COOT and refined by the program Refmac5. The statistics of the data collection and the final refinement statistics are summarized in Table S1. The PDB accession codes for ME1-NADP^+^-Mn^2+^ and ME1-L-lactate-NADP^+^-Mn^2+^ are 23JW and 23JV, respectively. All the structures shown in Figures were prepared in PyMol (http://www.pymol.org).

### Isothermal titration calorimetry (ITC) assay

ITC measurements were performed on a MicroCal PEAQ-ITC (Malvern) at 25 °C. ME1^WT^ or ME1^R155A^ protein samples were in buffer containing 30 mM Tris-HCl (pH 7.4), 0.3 mM NADPH, 5 mM MnCl_2_ and 300 mM NaCl. Titrations were carried out by individually injecting 40 μL aliquots of either 1 mM malate or 20 mM L-lactate into a 100 μM ME1^WT^ protein sample or 40 μL aliquots of 40 mM L-lactate into a 100 μM ME1^R155A^ protein sample at time intervals of 2.5 min. The titration data were analyzed using MicroCal PEAQ-ITC Analysis Software and fitted by the one-site binding model.

### Cell proliferation and cell viability

For the cell proliferation assay, cells were plated in triplicate in 12-well plates at 4000–20,000 cells per well. The culture medium was supplemented with 10% normal FBS (Gibco, 10099-141C) and NaCl in the presence or absence of sodium L-lactate (110 mM NaCl at the final concentration). The cell numbers were calculated by a BECKMAN COULTER Vi-cell XR. To determine cell viability, cells were seeded at 2 × 10^6^ cells per well in 6-well plates, and cell viability was calculated by a BECKMAN COULTER Vi-cell XR.

### Cell migration and invasion assays

PC3 cells were prestarved in serum-free RPMI 1640 medium for 24 h. Then, the cells were plated in the upper chamber of Transwells (Corning, Cat. No. 3422) or Matrigel-coated invasion inserts (Corning, Cat. No. 3554480) in serum-free RPMI 1640 medium with or without 10 mM sodium L-lactate. RPMI 1640 medium (600 μL) containing 10% FBS with or without 10 mM sodium L-lactate was added to the lower chambers. After 48 h, the cells on the upper membrane were scraped, and the membrane was fixed and stained with 0.5% crystal violet in methanol solution.

### ATP production measurement

To measure intracellular ATP production, the cells were subjected to an ATP assay kit (Beyotime, S0026). After being seeded into 6-well plates for 48 h, the PC3 cells were treated with nutrient-depleted medium supplemented with low glucose and low glutamine according to the manufacturer’s instructions.

### Seahorse assay

ECAR and OCR were determined using an XF96 Extracellular Flux Analyzer (Seahorse Bioscience) as previously described.^50^ In brief, 8 × 10^3^ cells were seeded into 96-well plates and incubated overnight. In accordance with the manufacturer’s instructions, after normal medium or nutrient-depleted medium with or without sodium L-lactate was added, a Glycolysis Stress Kit or a Cell Mito Stress Test Kit was used to measure cellular mitochondrial flux. Oligomycin, FCCP and rotenone were added to each well at final concentrations of 1.5 μM, 1 μM, and 1 μM, respectively. For the acute injection assay, after treatment with nutrient-depleted medium, a Cell Mito Stress Test Kit was used to measure cellular mitochondrial flux. Sodium L-lactate/NaCl, oligomycin, FCCP and rotenone were added to each well at final concentrations of 30 mM, 1.5 μM, 1 μM, and 1 μM, respectively. All the data were normalized to the cell number.

### Animal experiments

The procedures were approved by the ethics committee of the Department of Laboratory Animals, Fudan University. *Me1*^*flox/flox*^ mice on the C57BL/6 background were generated through separate insertion of the loxp site (loxp: ATAACTTCGTATAGCATACATTATACGAAGTTAT) upstream and downstream of exon 4 (sequence: AGGCCTCTTTATTAGTATCCATGACAAAGGGCACATTGCTTCAGTTCTTAATGCATGGCCAGAGGATGTCGTCAAG, 76 bp) of the Me1 gene (Gene ID: 17436) by BRL Medicine Inc. A modified prostate-specific promoter, Probasin, was used to drive postnatal expression of Cre recombinase (termed PB-Cre4),^19^ which was crossbred with *Pten*^flox/flox^ to generate *Pten*^*flox/flox*^; PB-cre4 (*Pten*^*pc-/-*^) mice. *Pten*^*pc-/-*^; *Me1*^*pc-/-*^ mice were generated by crossing *Me1*^*flox/flox*^ *mice* with *Pten*^*pc-/-*^ mice.

For the xenograft mouse model, 3 × 10^6^ cells were mixed with RPMI 1640 medium and Matrigel and subcutaneously injected into NSG mice. These mice were administered 2 g/kg sodium L-lactate via the tail vein daily or 100 mg/kg AZD3965 by oral gavage. After the tumors were harvested, tumor volume and weight were calculated.

### Human tissue specimens

A tissue microarray (TMAs) containing paired tumor and adjacent normal tissue sections was constructed by Shanghai Outdo Biotech Co., Ltd. (HProA150CS01). This study was approved by the Ethics Committee of Shanghai Outdo Biotech Co., Ltd (approve number: # SHYJS-CP-1907004). All the experiments were performed in accordance with all the relevant ethical regulations. All participants in this study signed informed consent forms. No participant compensation was offered. Pathology diagnosis information was directly provided by Shanghai Outdo Biotech Co., Ltd. and is shown in Table S2.

### Histochemistry, immunohistochemistry, tissue microarray staining and image analysis

The tissues were dissected and fixed in 4% paraformaldehyde (PFA) for 24 h, followed by paraffin embedding. Sections (4 μm) were processed for hematoxylin and eosin staining using standard methods. Immunohistochemical staining was performed as previously described.^50^ In brief, 4-μm-thick sections were baked overnight and deparaffinized using a series of xylene, ethanol, and water solutions. Antigen retrieval was performed by incubating the slides in 0.01 M citrate buffer (pH 6.0) for 15 min. Endogenous peroxidase activity was inactivated in a solution containing 3% hydrogen peroxide (H_2_O_2_) in methanol. After being washed with nanopure water and TBS-20, the slides were immersed in 5% goat serum for blocking, followed by incubation with primary antibodies at 4 °C overnight. After the sections were incubated with secondary antibody, they were detected using a diaminobenzidine kit according to the manufacturer’s instructions. Histochemistry and immunohistochemistry images were recorded using an Olympus microscope. ImageJ software was used for image analyses.

### Immunofluorescence and microscopy

Prostate tissue from *Pten*^*pc-/*-^ mice and xenograft tumors from NSG mice were dissected and fixed in 4% paraformaldehyde (PFA) for 24 h, followed by paraffin embedding. The sections were baked, deparaffinized, and subjected to antigen retrieval according to previously described procedures. After blocking, the slides were incubated with primary antibody overnight. The following day, secondary antibody incubation was performed at room temperature for 2 h. DAPI in PBS was added prior to imaging. For cellular nuclear translocation, cells were cultured in glass-bottom dishes with L-lactate treatment for 24 h and then fixed with 4% paraformaldehyde, followed by permeabilization in 0.1% Triton-x-X100 for 10 min. After blocking in 10% BSA for 30 min, the primary antibodies were incubated overnight at 4 °C. The next day, the cells were incubated with secondary antibodies for 1 h, and nuclear staining with DAPI was performed for 15 min. All the confocal microscopy images were collected with an Olympus FV3000 and a Leica SP5, followed by analysis with ImageJ software.

### Statistics and reproducibility

The statistical procedures are indicated in the figure legends and were performed using GraphPad Prism 8. *P* values < 0.05 were considered significant (*p < 0.05, **p < 0.01, ***p < 0.001, ****p < 0.0001, and ns, not significant). Each experiment was repeated independently with similar results.

## Supplementary information

Supplementary material

Data S3

Data S1

Data S2

Data S2

## Data Availability

The mass spectrometry proteomics data have been deposited to the ProteomeXchange Consortium via the PRIDE partner repository with the dataset identifier PXD077606. The coordinates and structural factors for the ME1-NADP^+^-Mn^2+^ complex have been deposited in the Protein Data Bank (http://www.rcsb.org/) under the accession number 23JW. The coordinates and structural factors for the ME1-L-lactate-NADP^+^-Mn^2+^ complex have been deposited in the PDB under accession number 23JV.
