# Supplementary material for: Malic enzyme 1 senses L-lactate to determine tumor heterogeneity
Source: Signal Transduct Target Ther. 2026 Sep 11;11:386. doi: 10.1038/s41392-026-02839-6 (PMC13569829; doi:10.1038/s41392-026-02839-6)

## Fig 1d

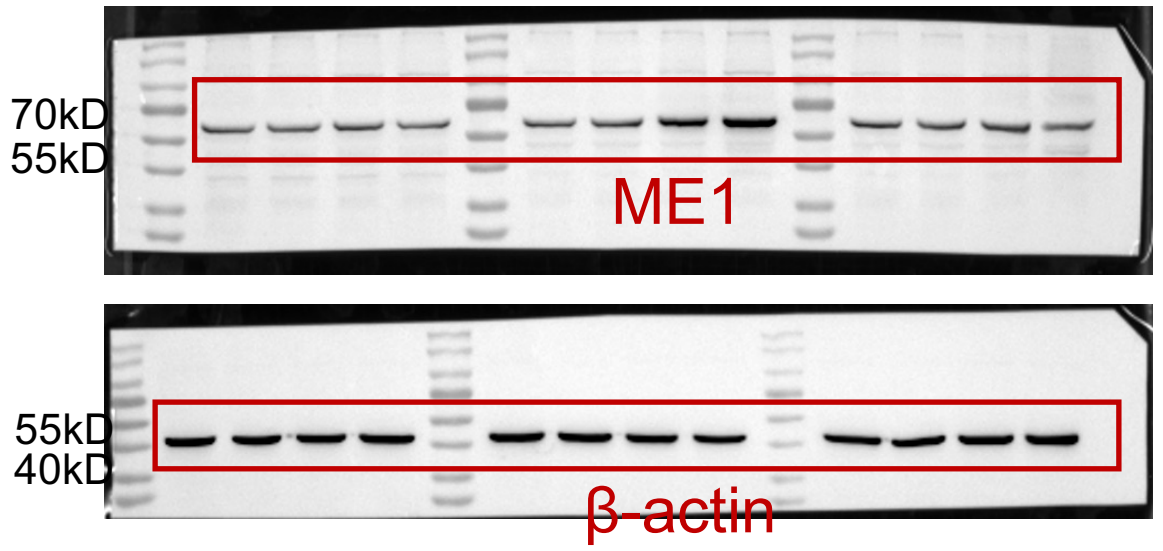


## Fig 3a

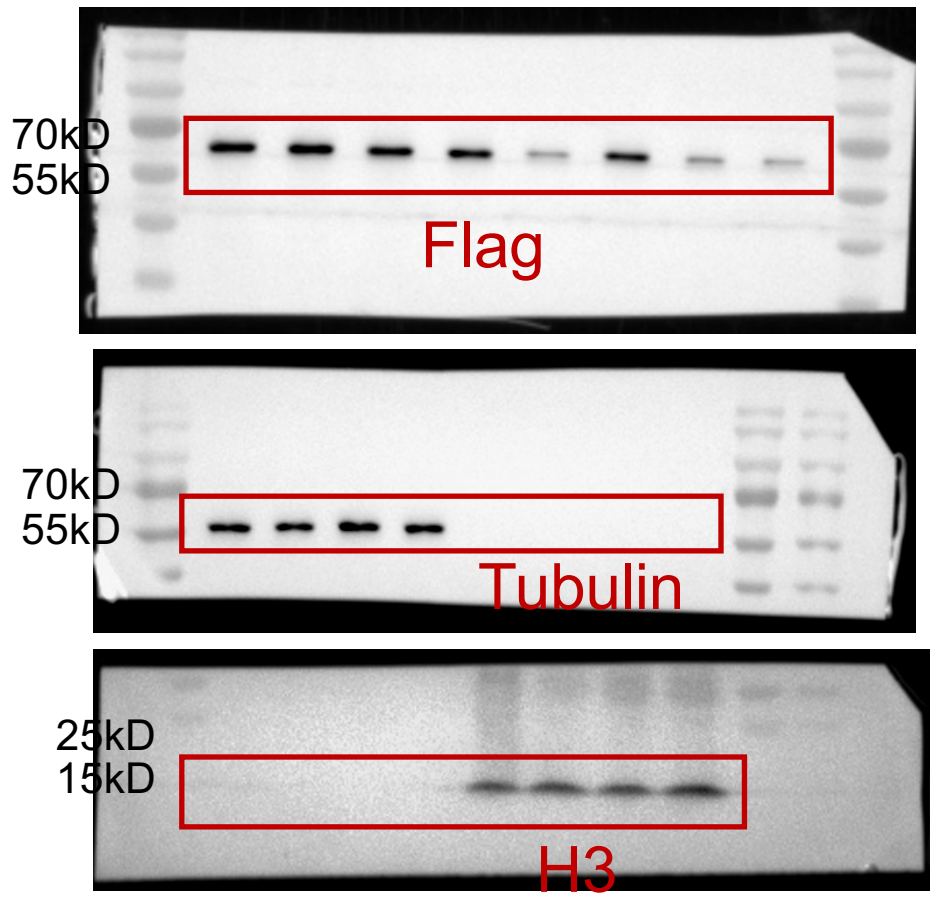


## Fig 3c

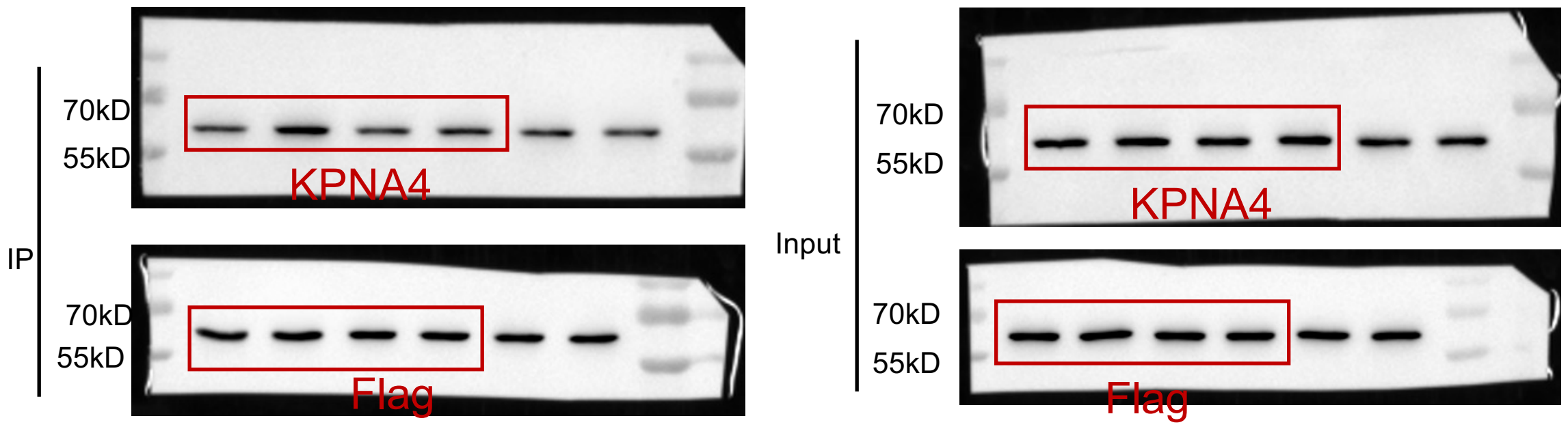


## Fig 3d

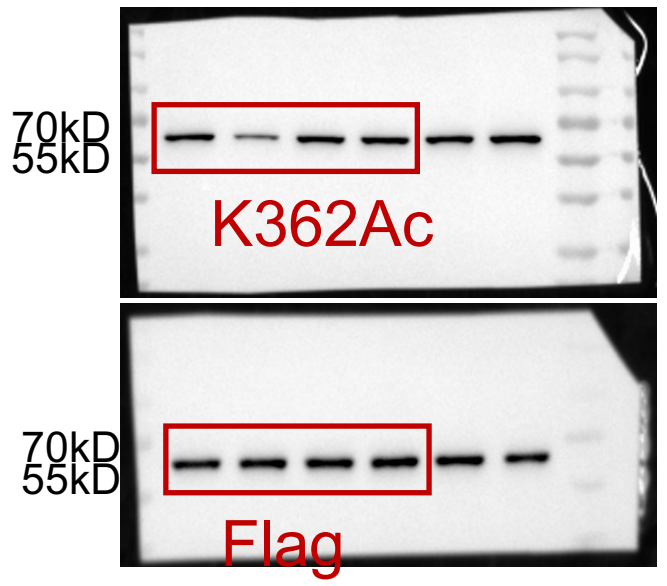

# Fig 3e

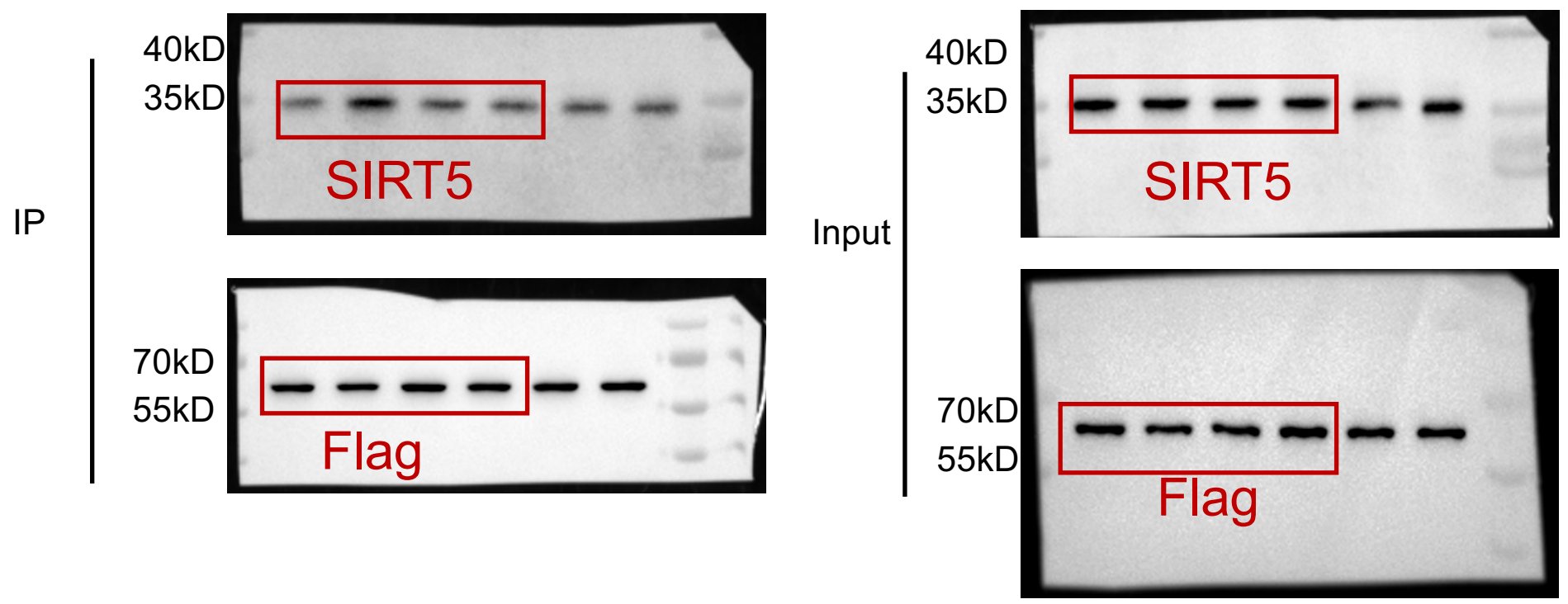


# Fig 3f

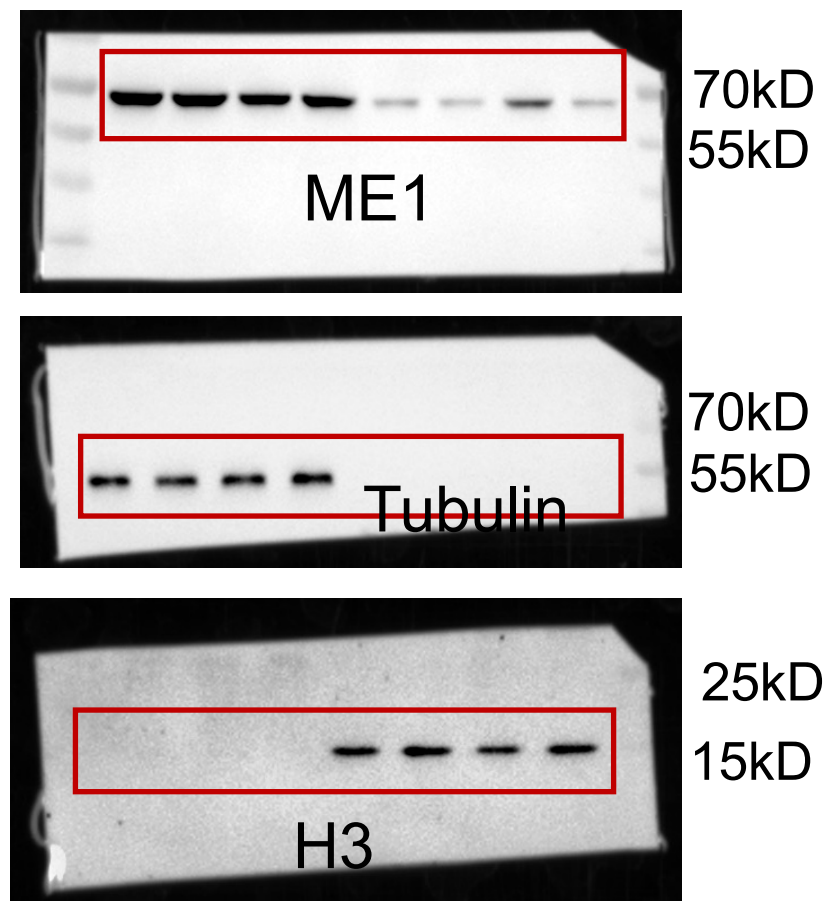


# Fig 3h

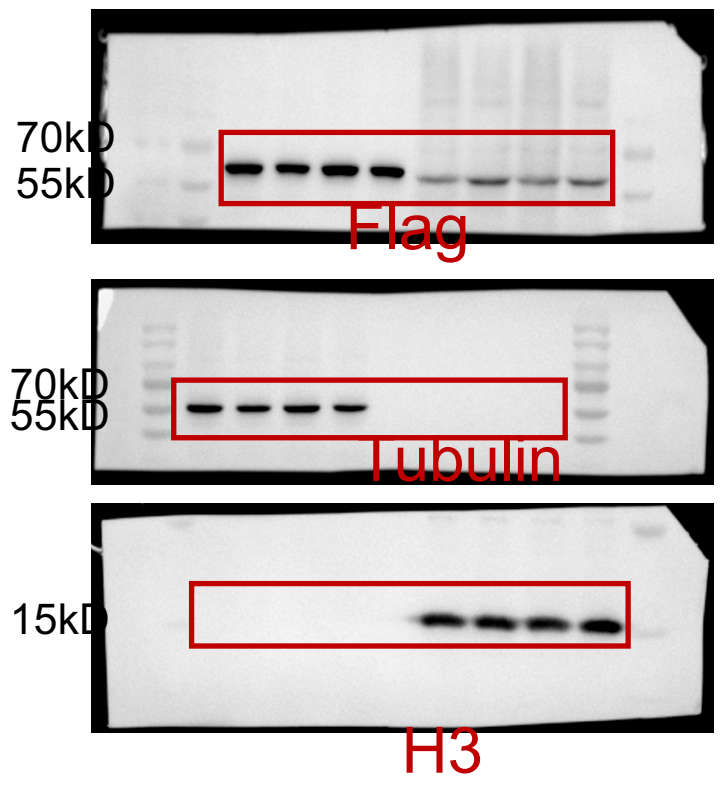


# Fig 3i

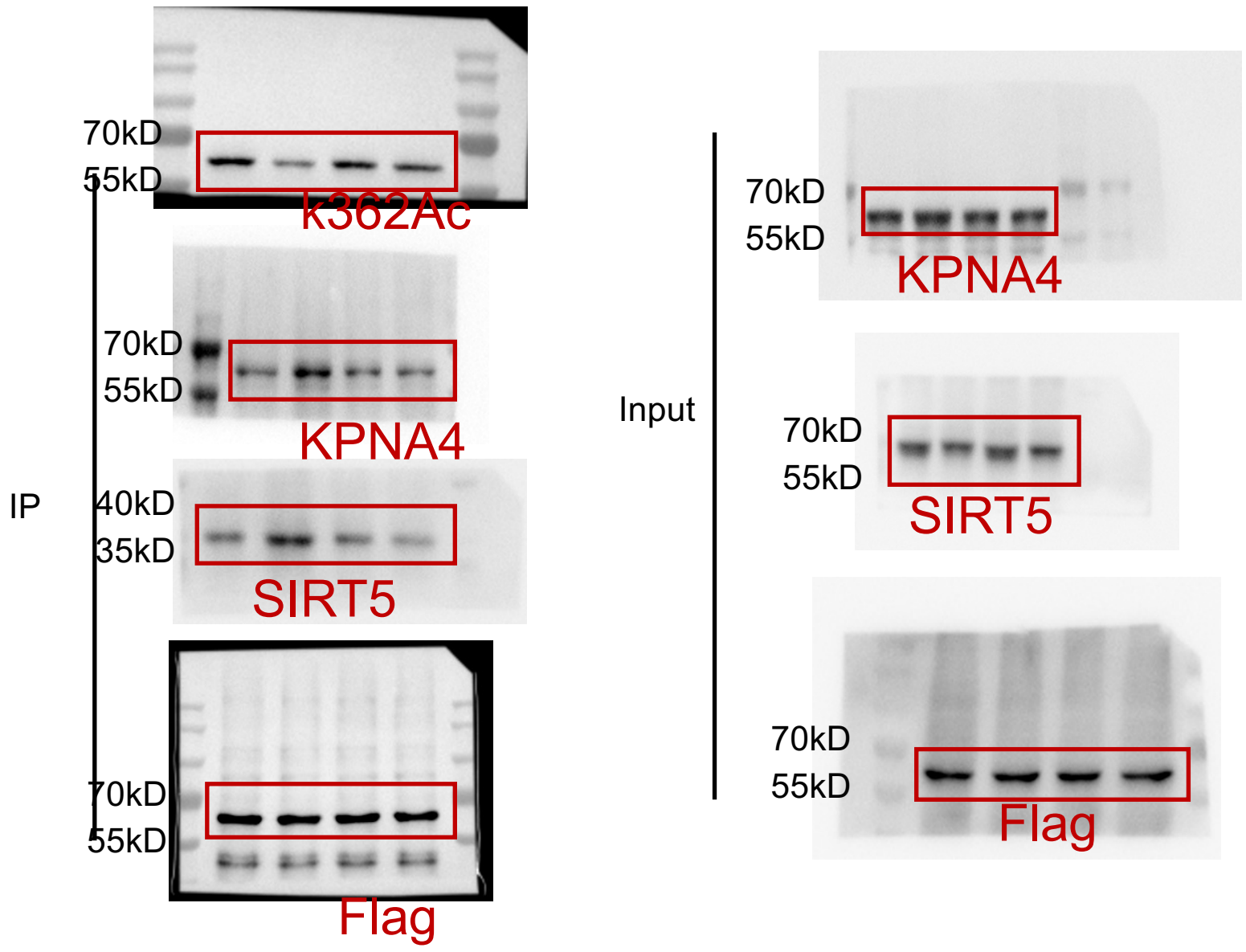

## Fig 4b

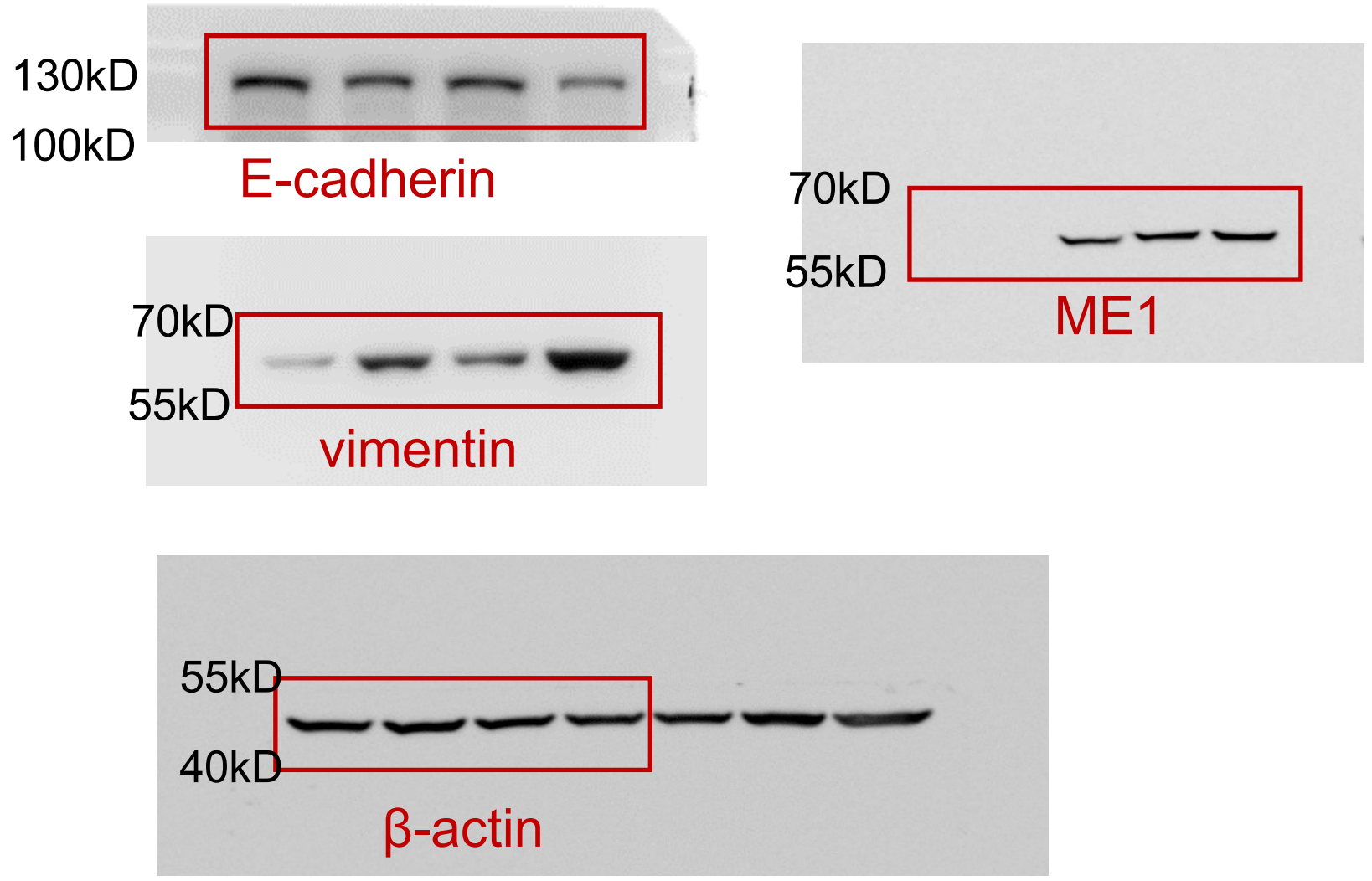


## Fig 5d

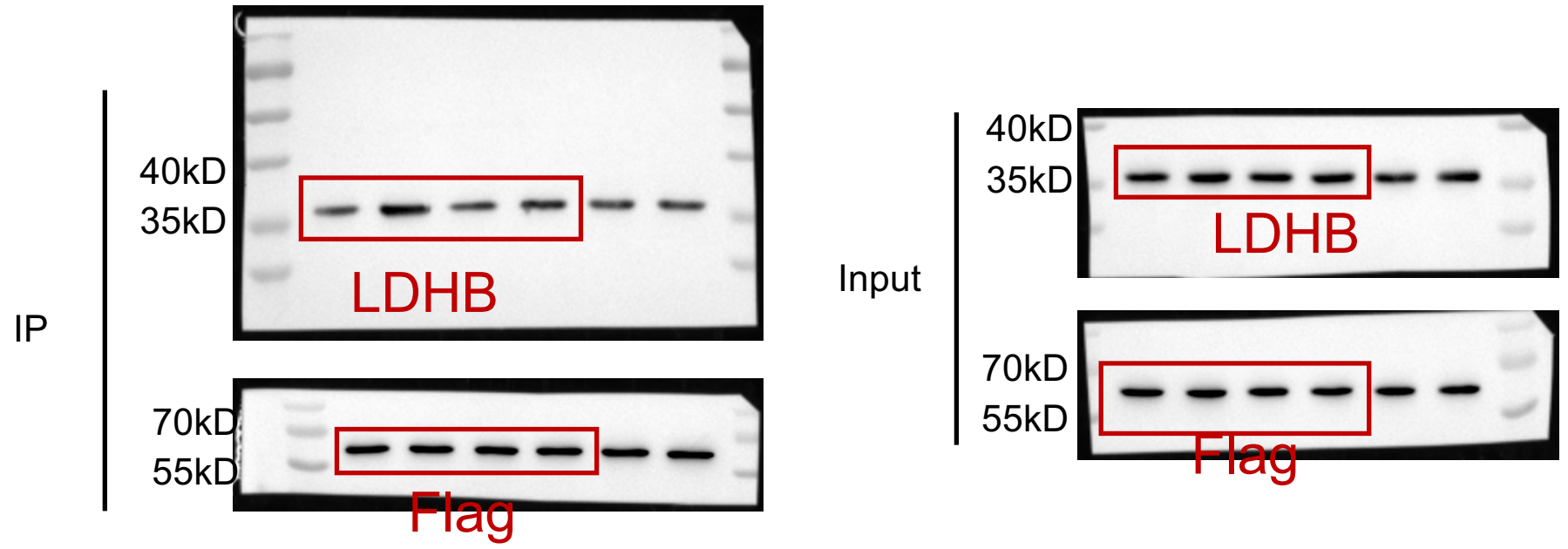


## Fig 5e

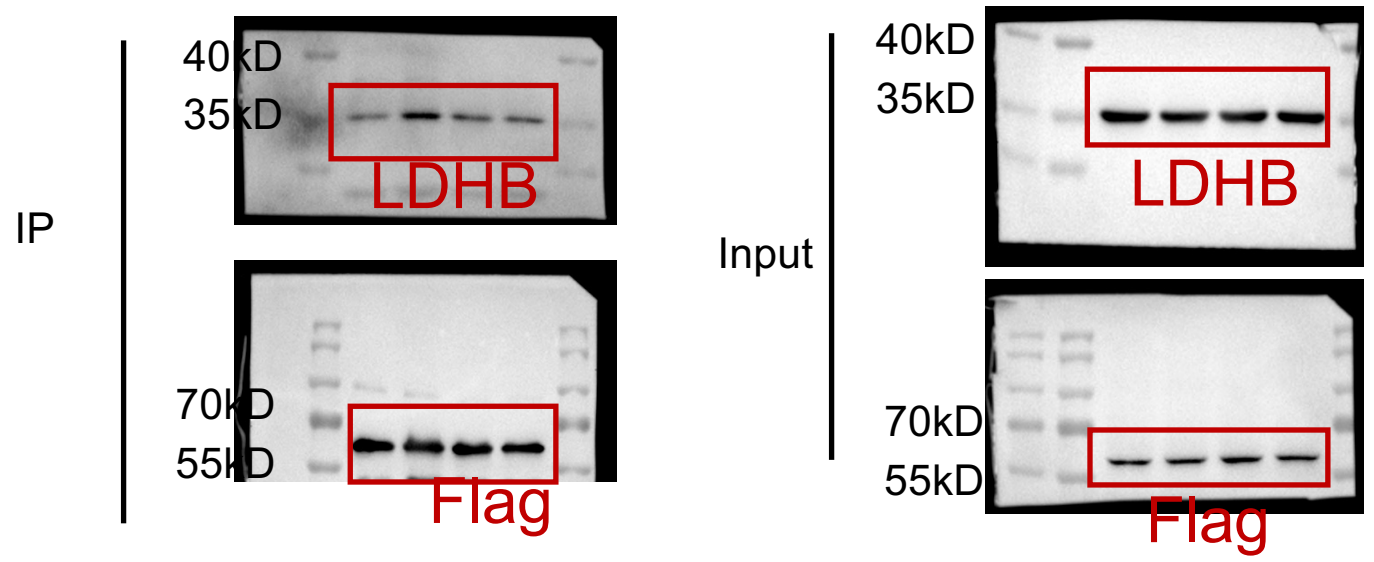

## Supplementary Fig 1f

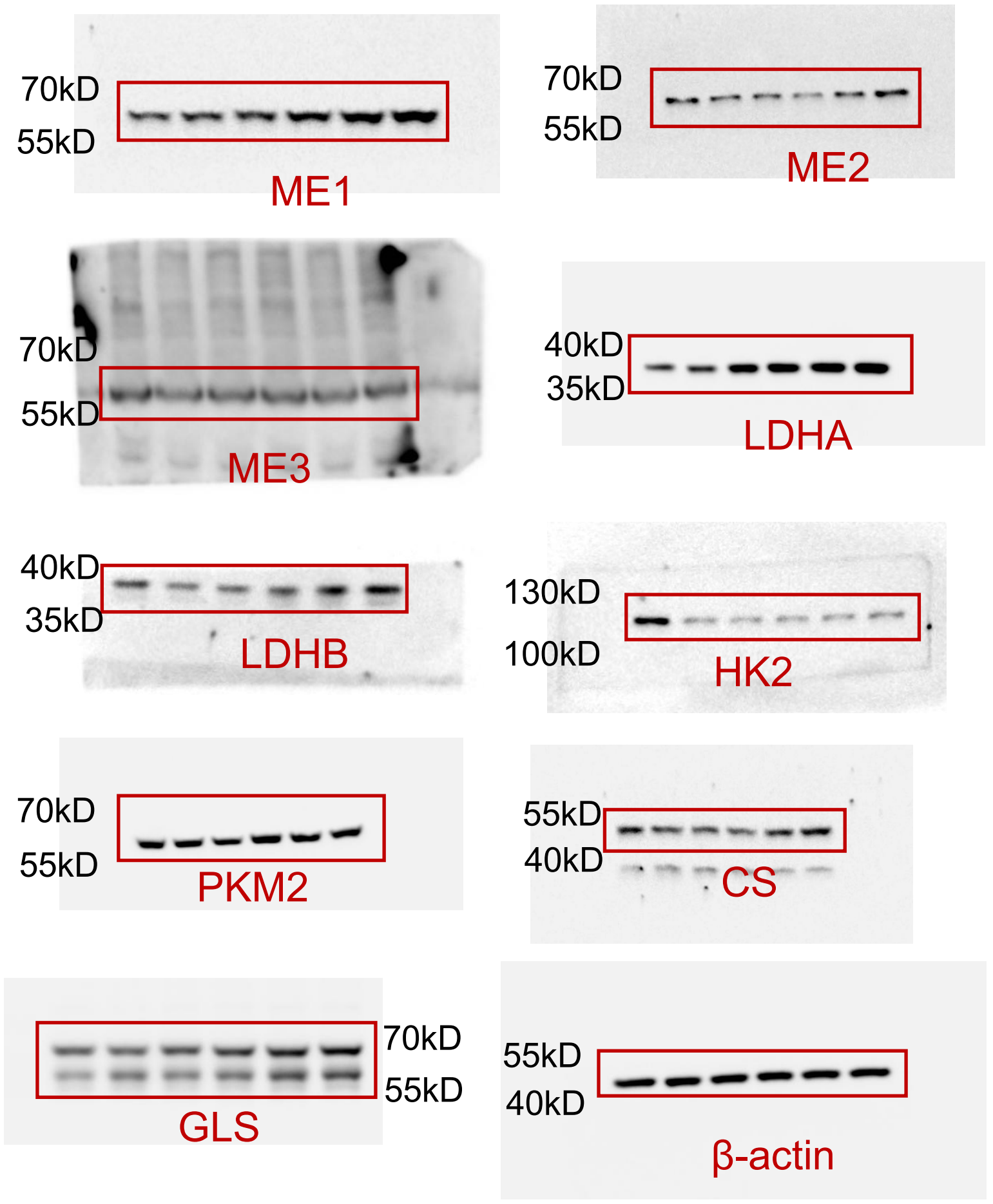


## Supplementary Fig 1i

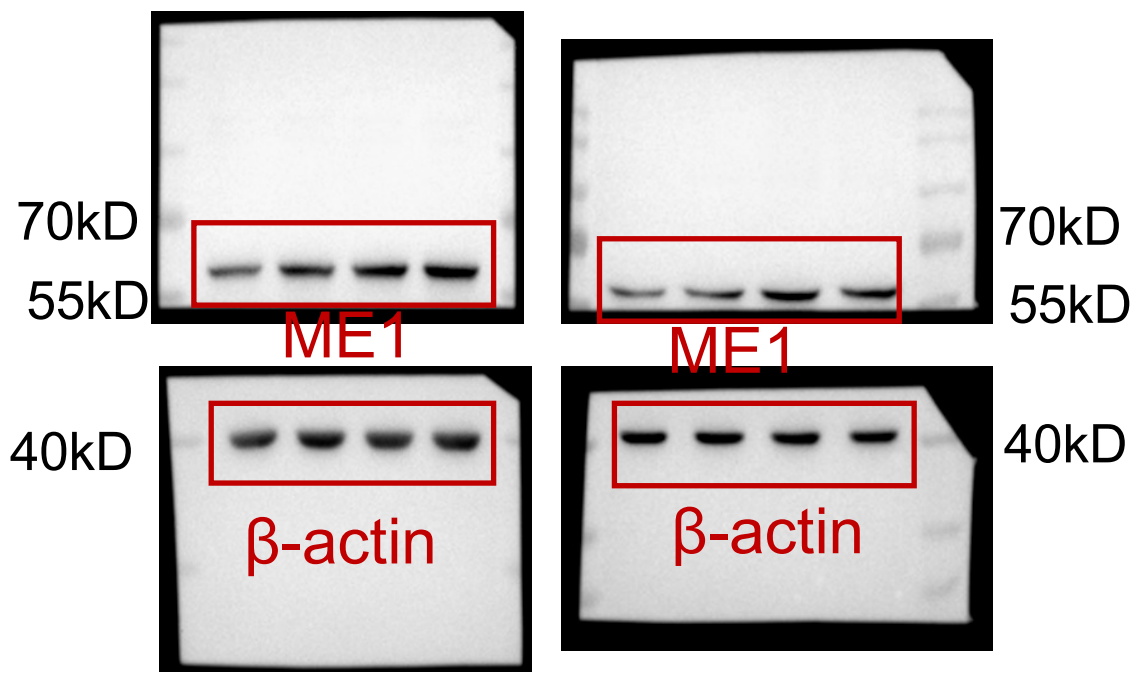


## Supplementary Fig 2d

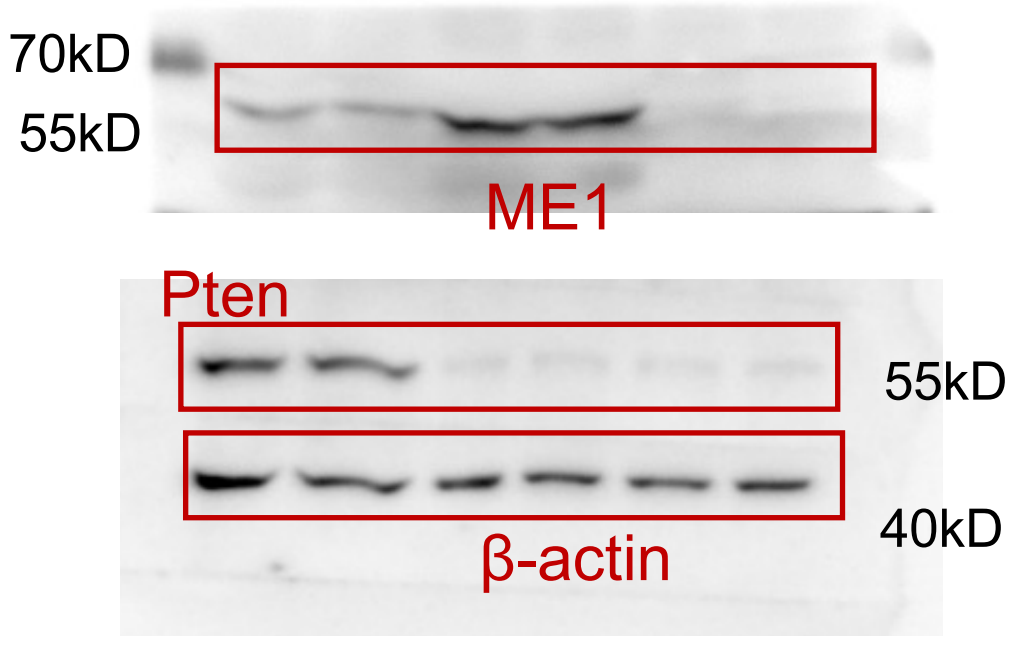

## Supplementary Fig 3h

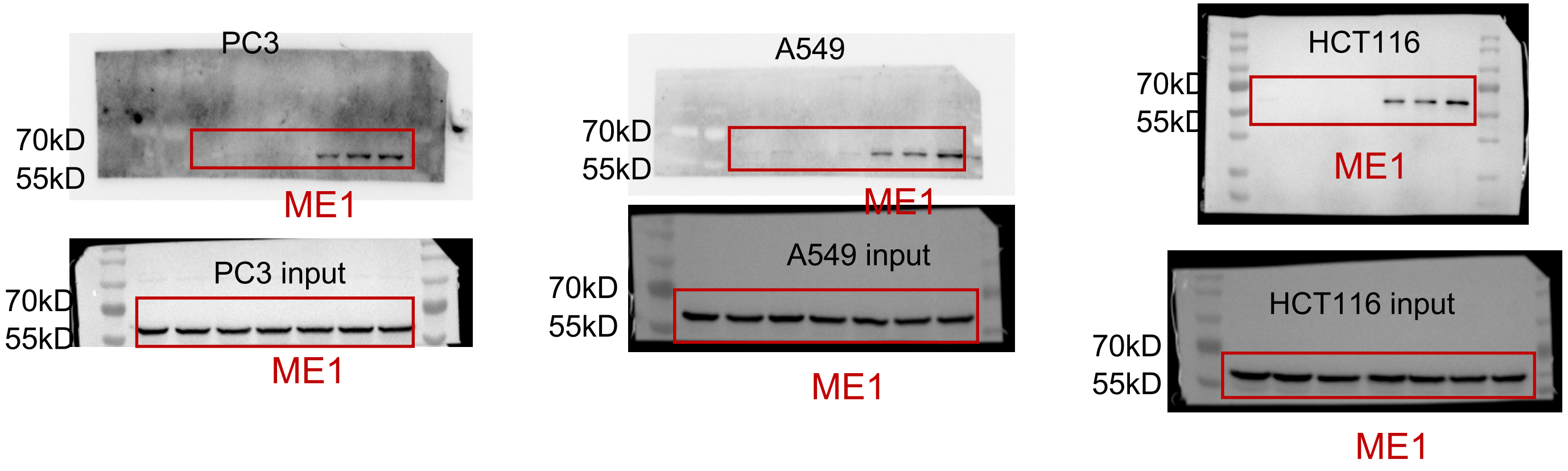


## Supplementary Fig 3i

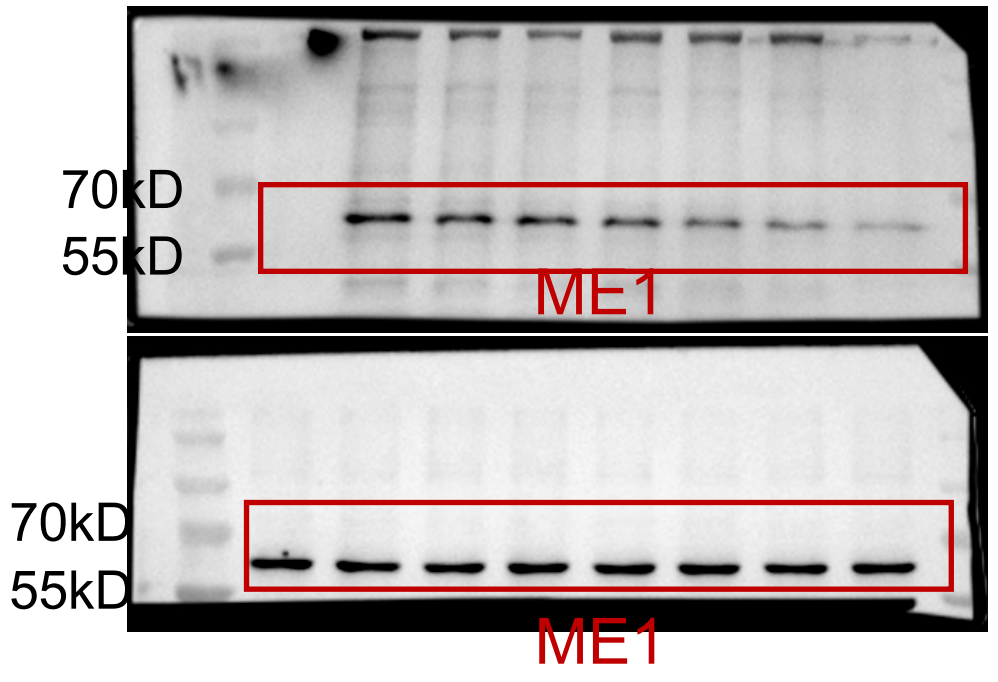


## Supplementary Fig 3j

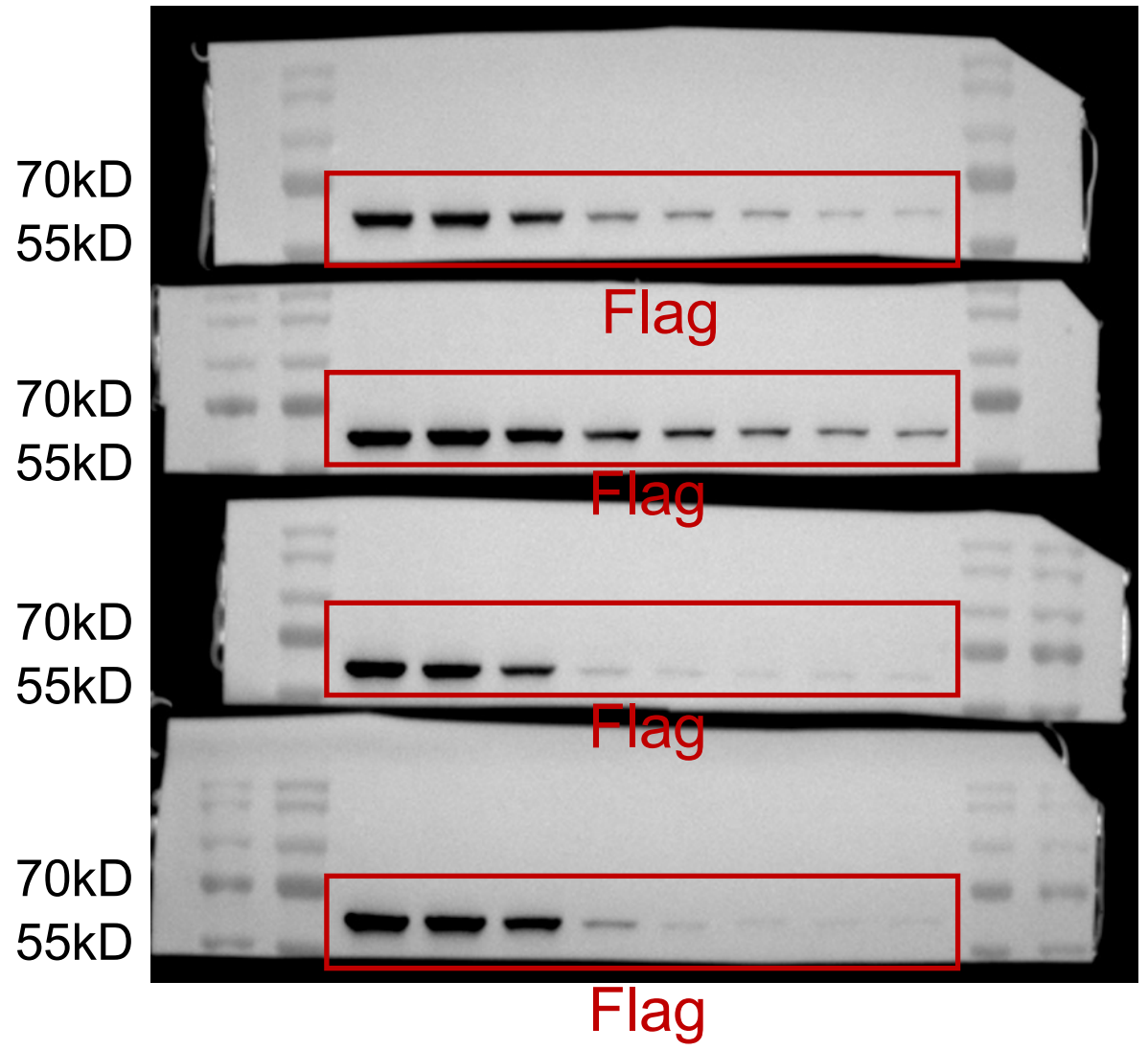

## Supplementary Fig 5b

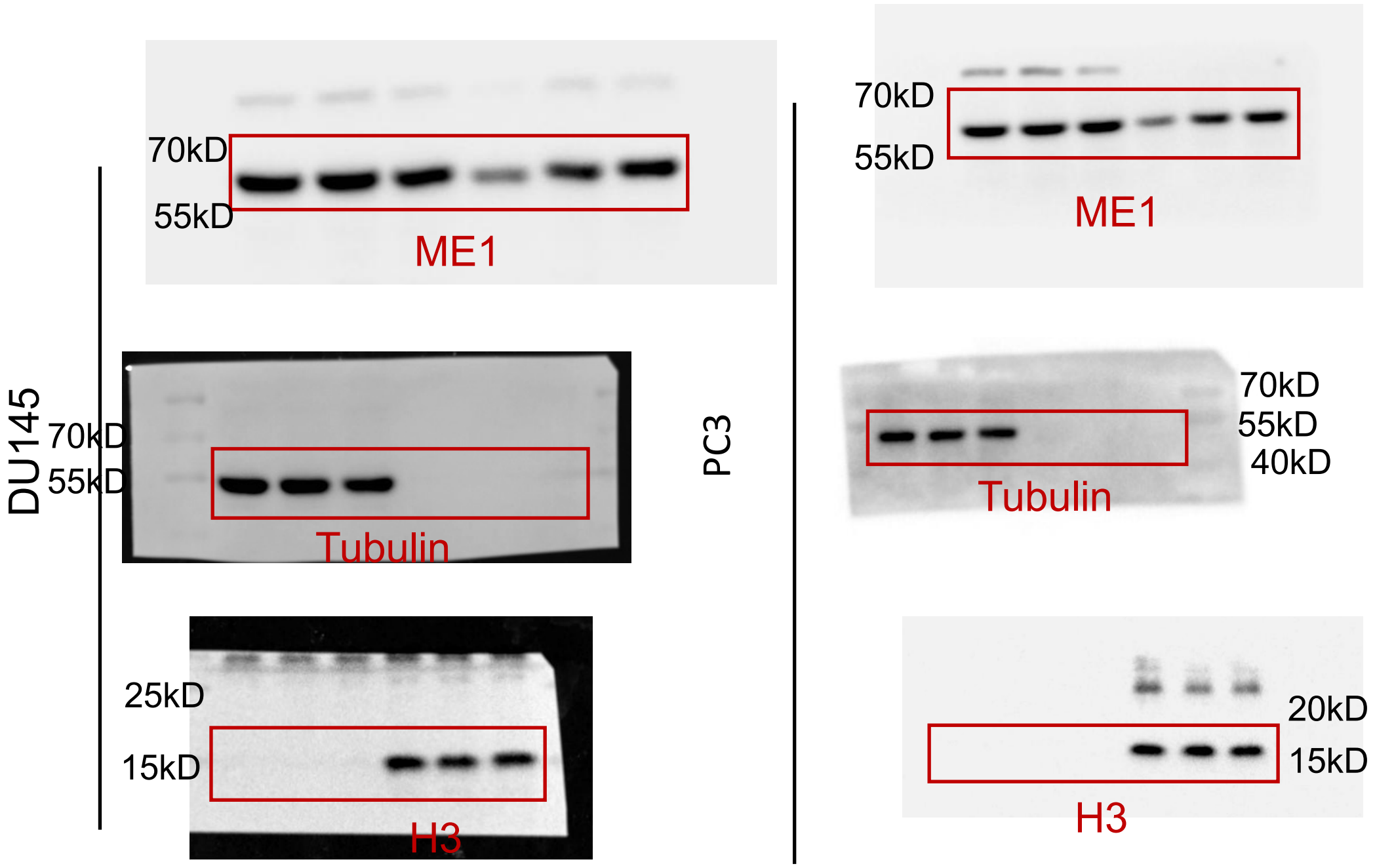


## Supplementary Fig 5c

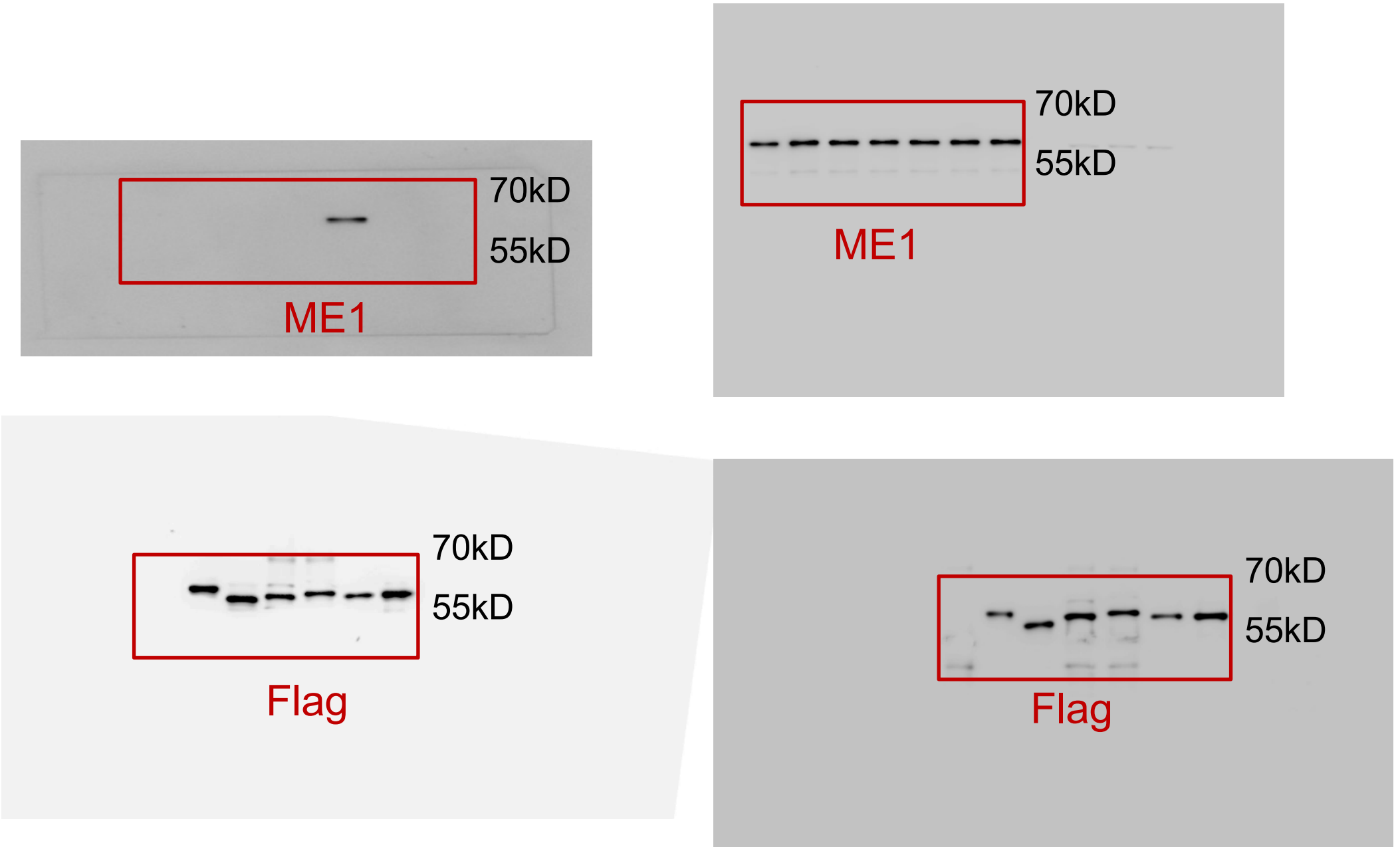

## Supplementary Fig 5d

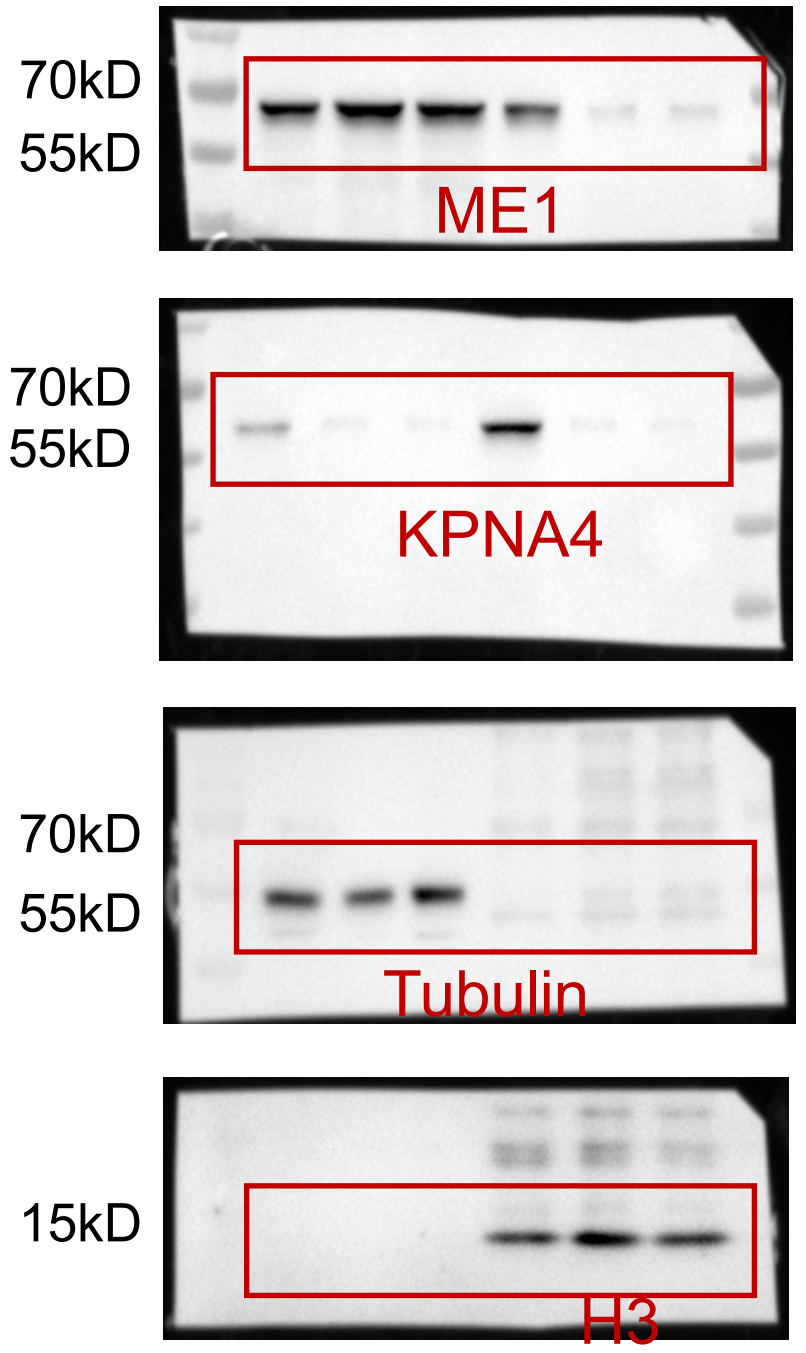


## Supplementary Fig 5e

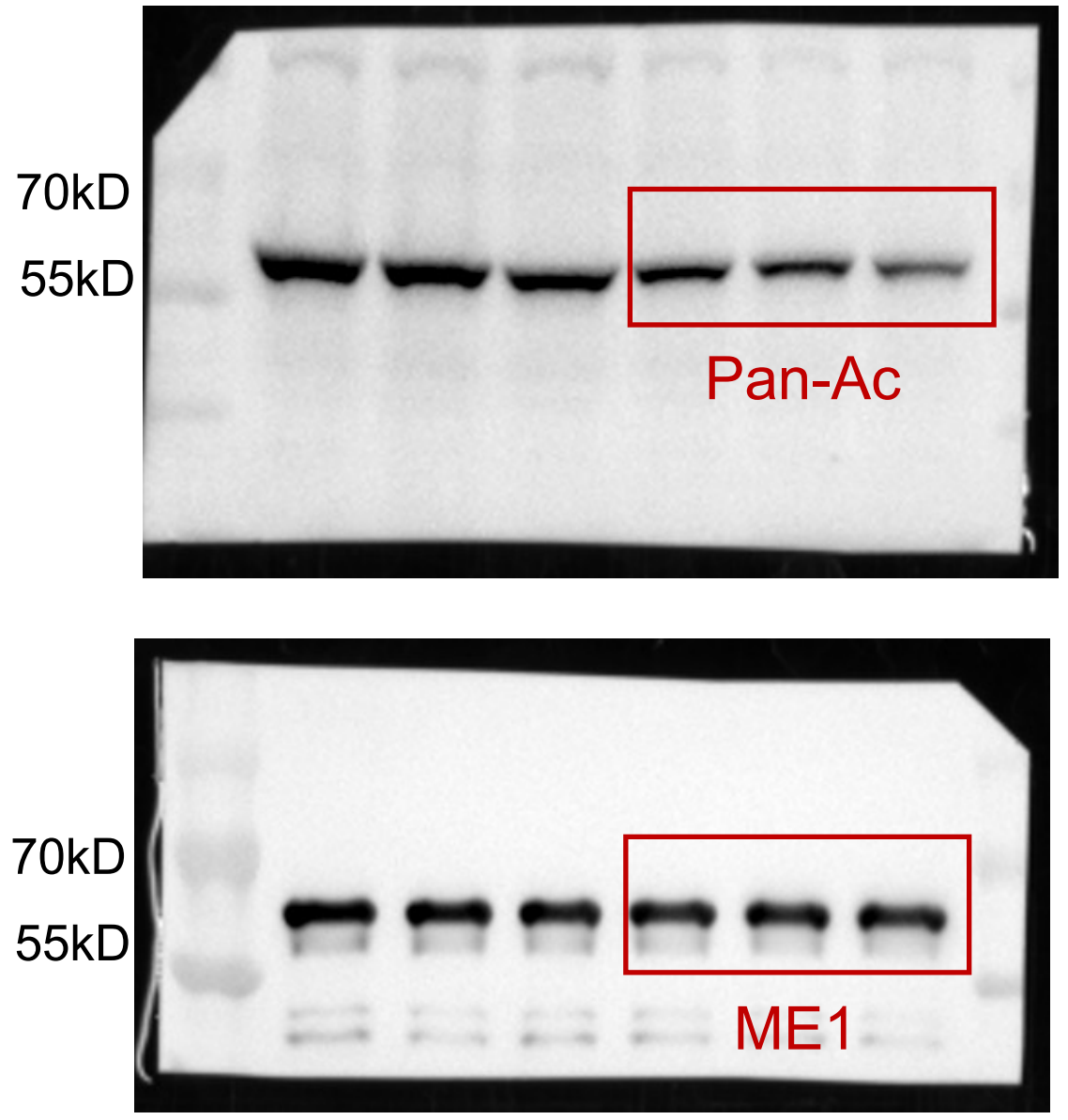


## Supplementary Fig 5f

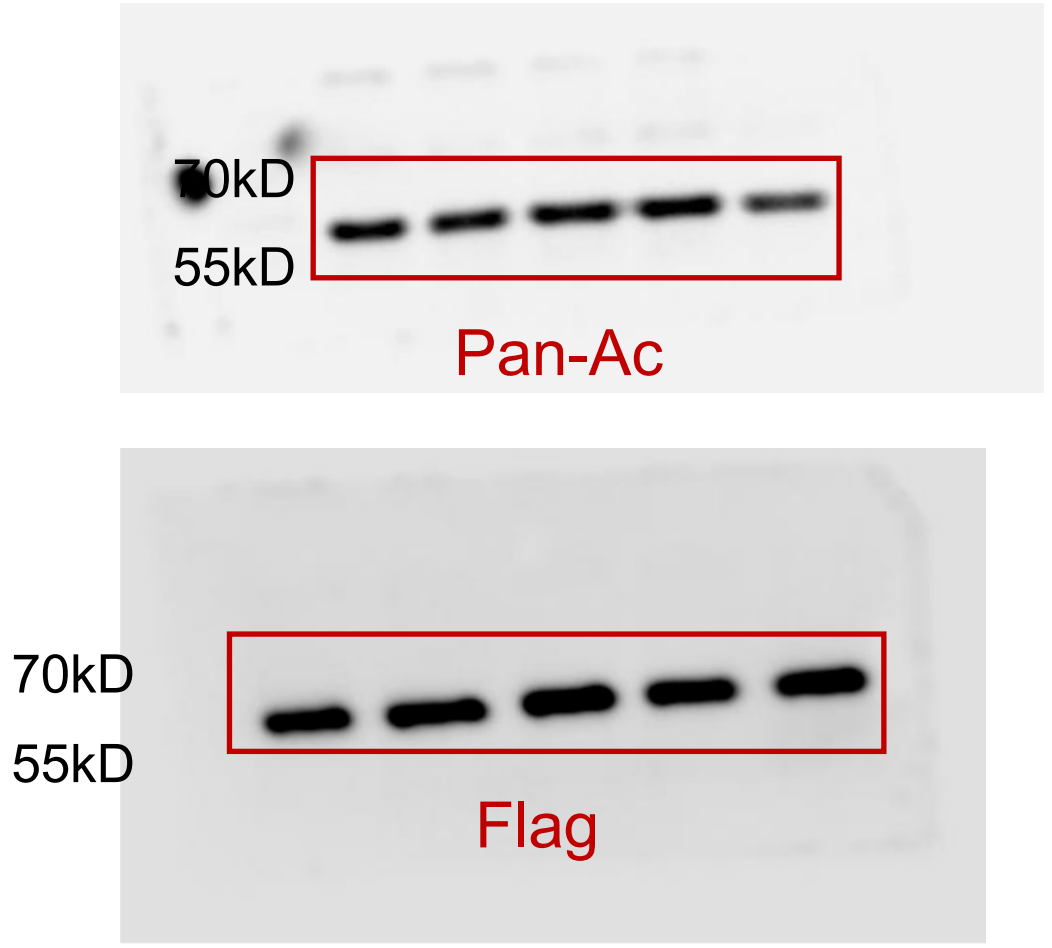

## Supplementary Fig 5g

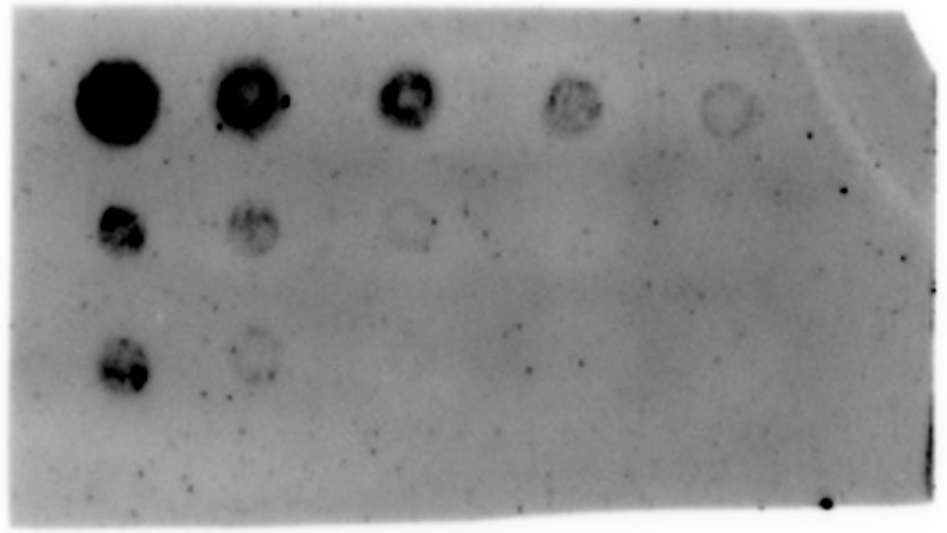

## Supplementary Fig 5h

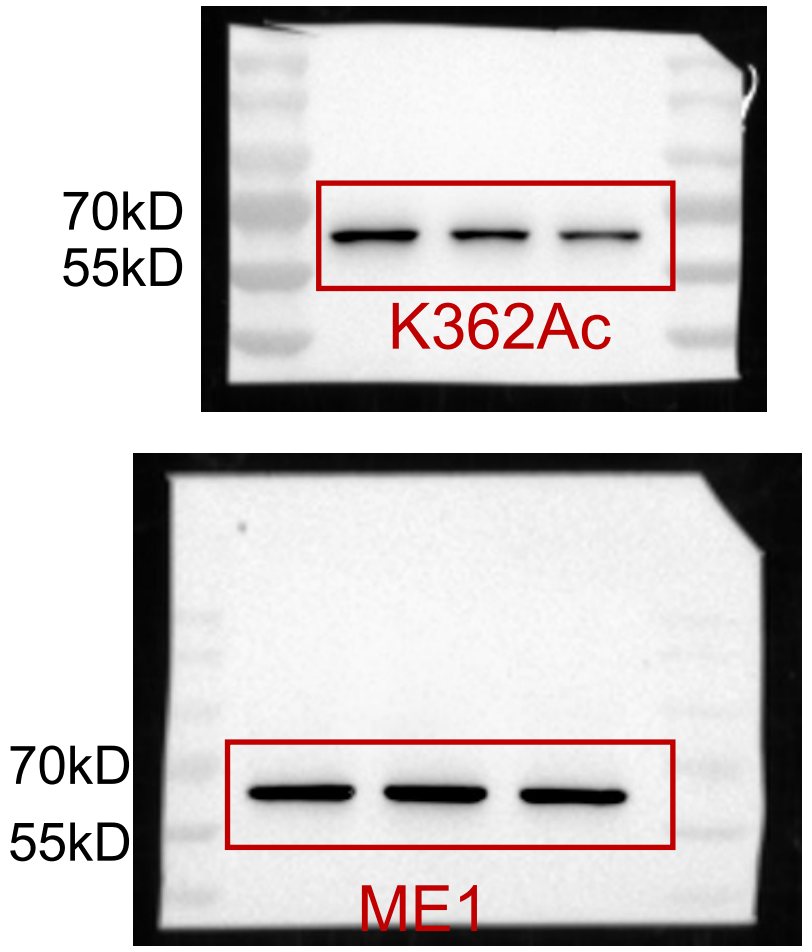


## Supplementary Fig 5i

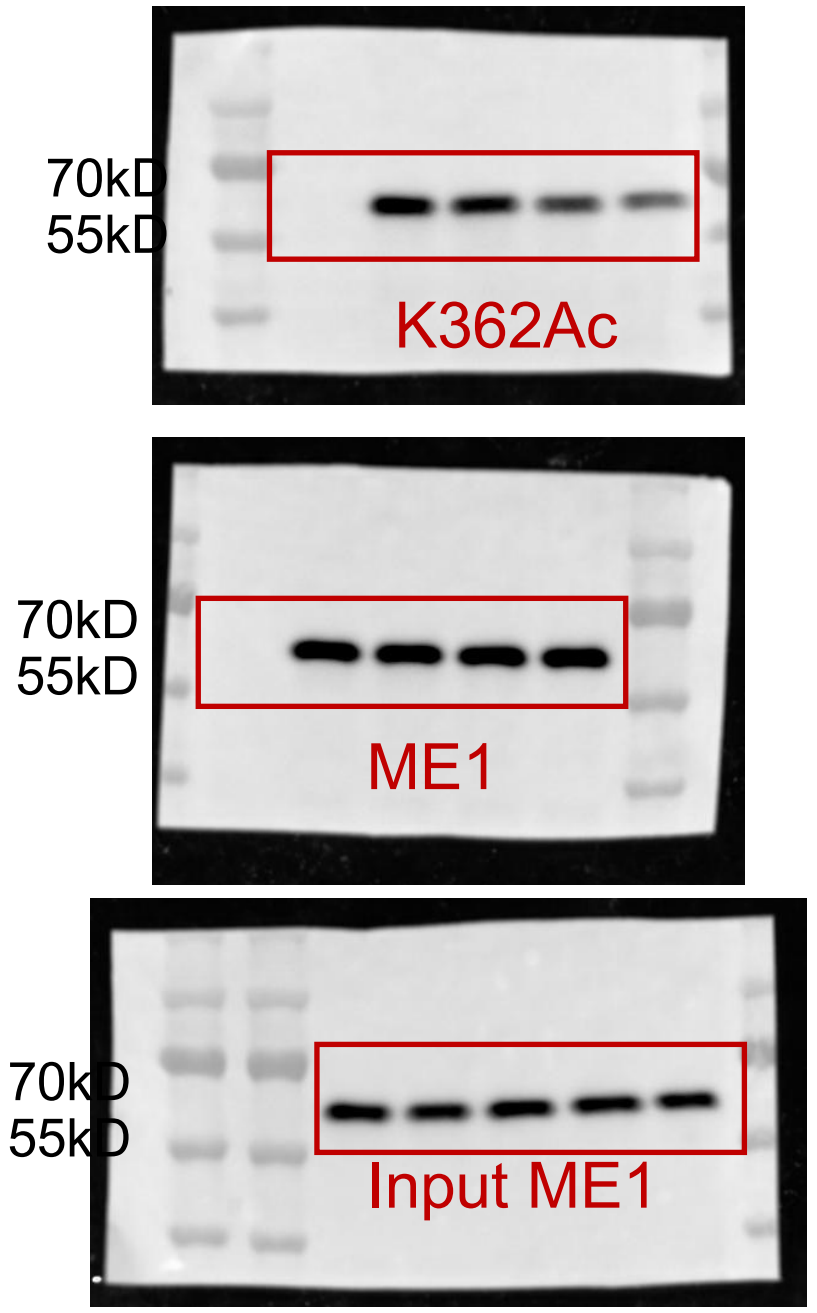

## Supplementary Fig 5j

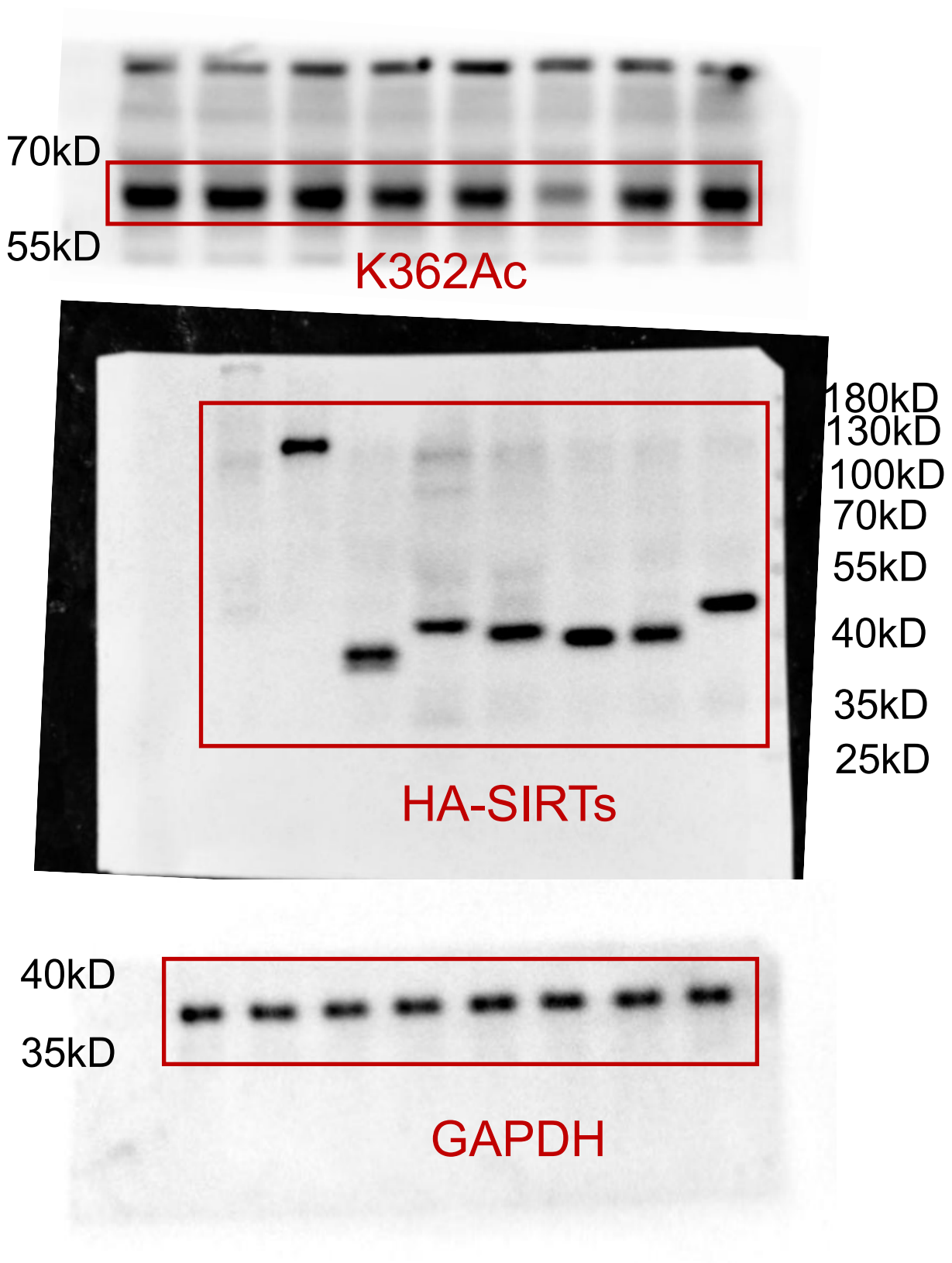


## Supplementary Fig 5k

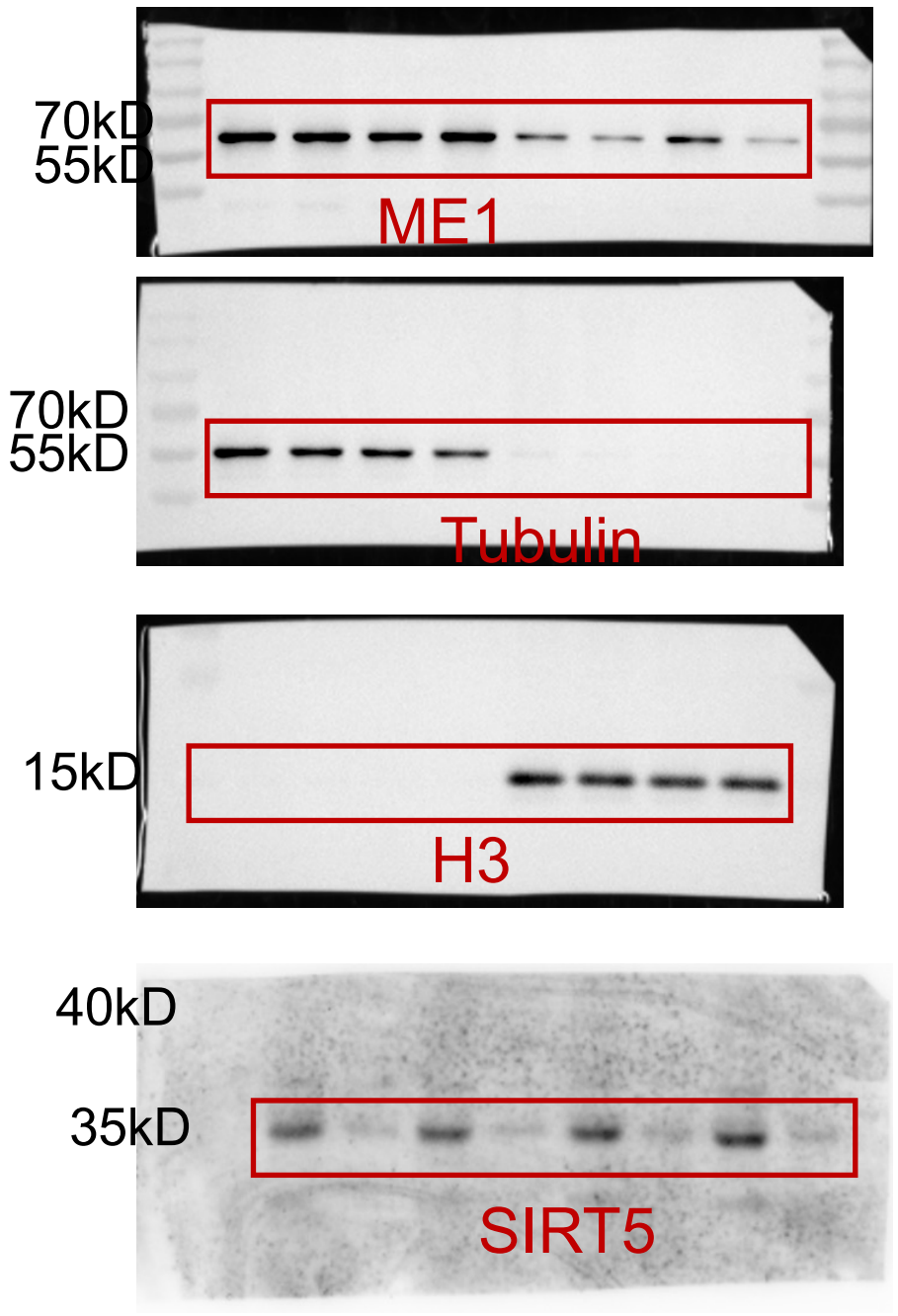

## Supplementary Fig 5I

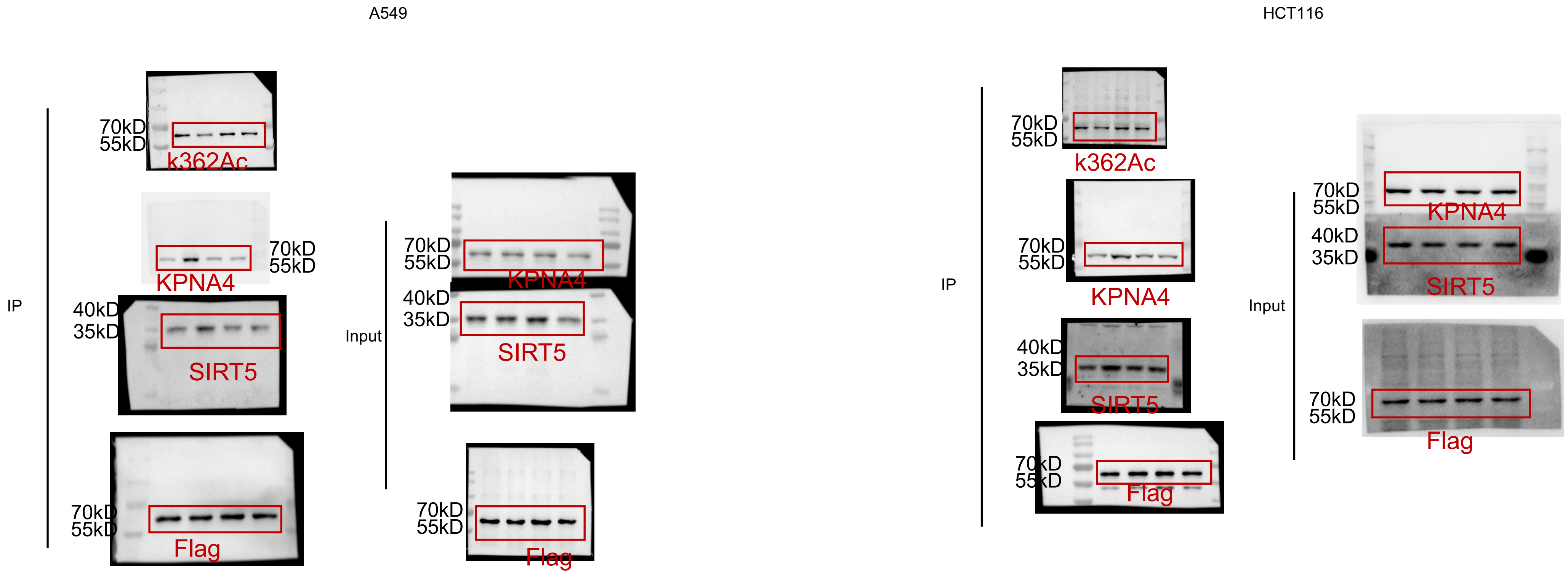

# Supplementary Fig 5M

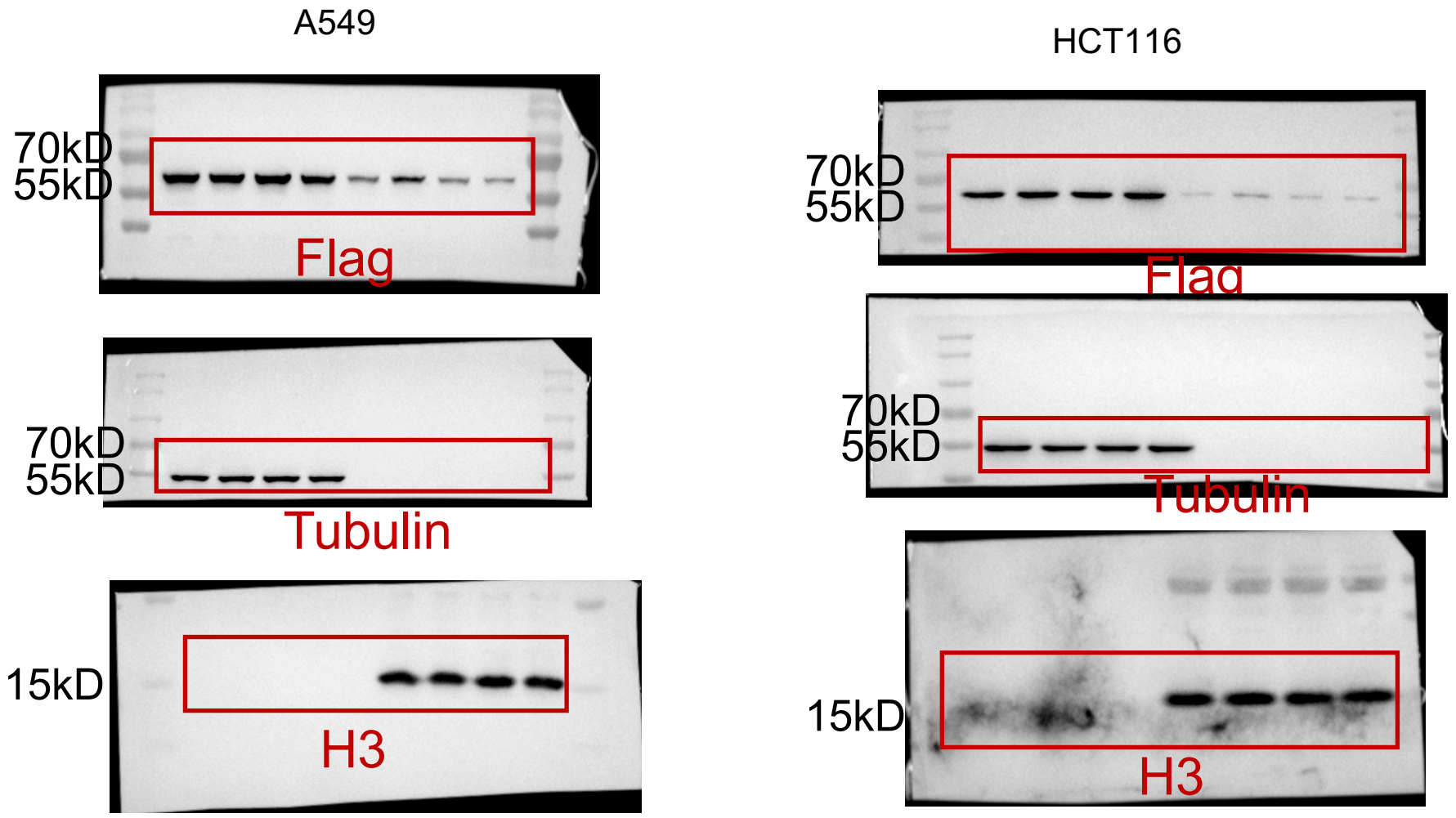

# Supplementary Fig 5n

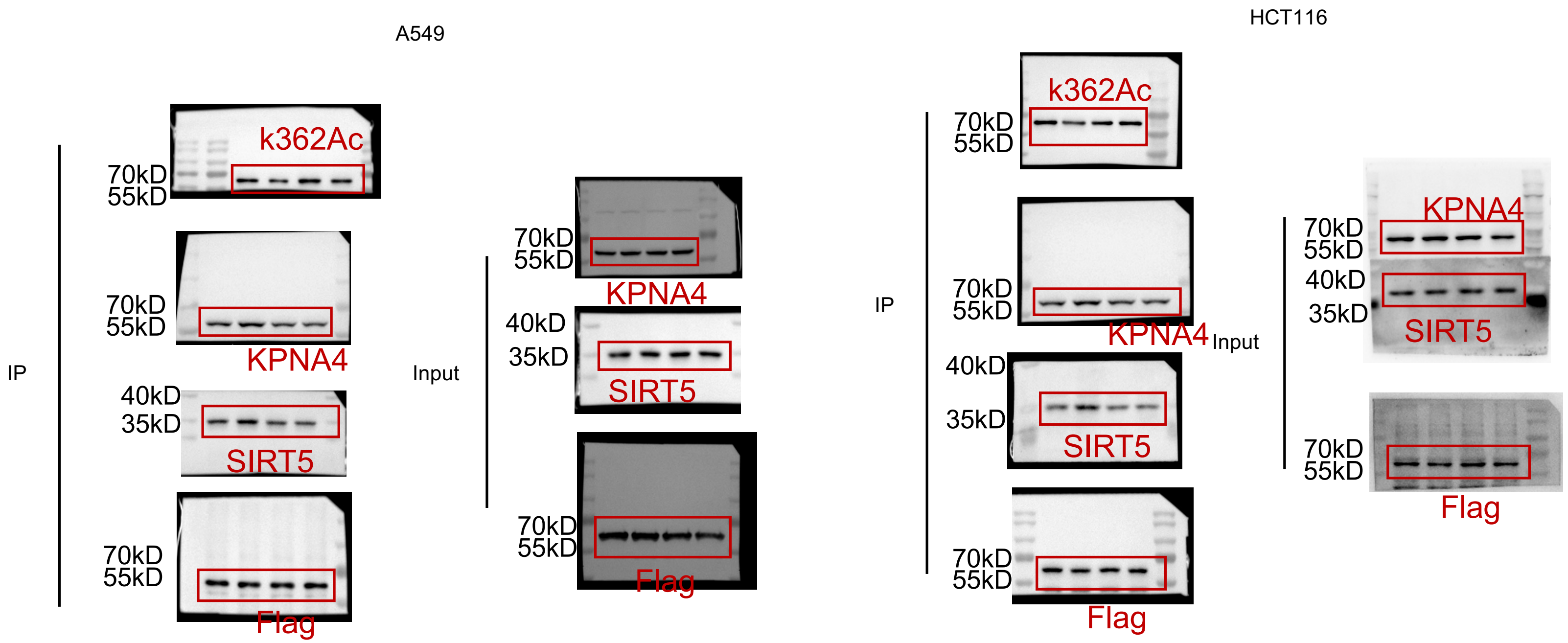

# Supplementary Fig 5o

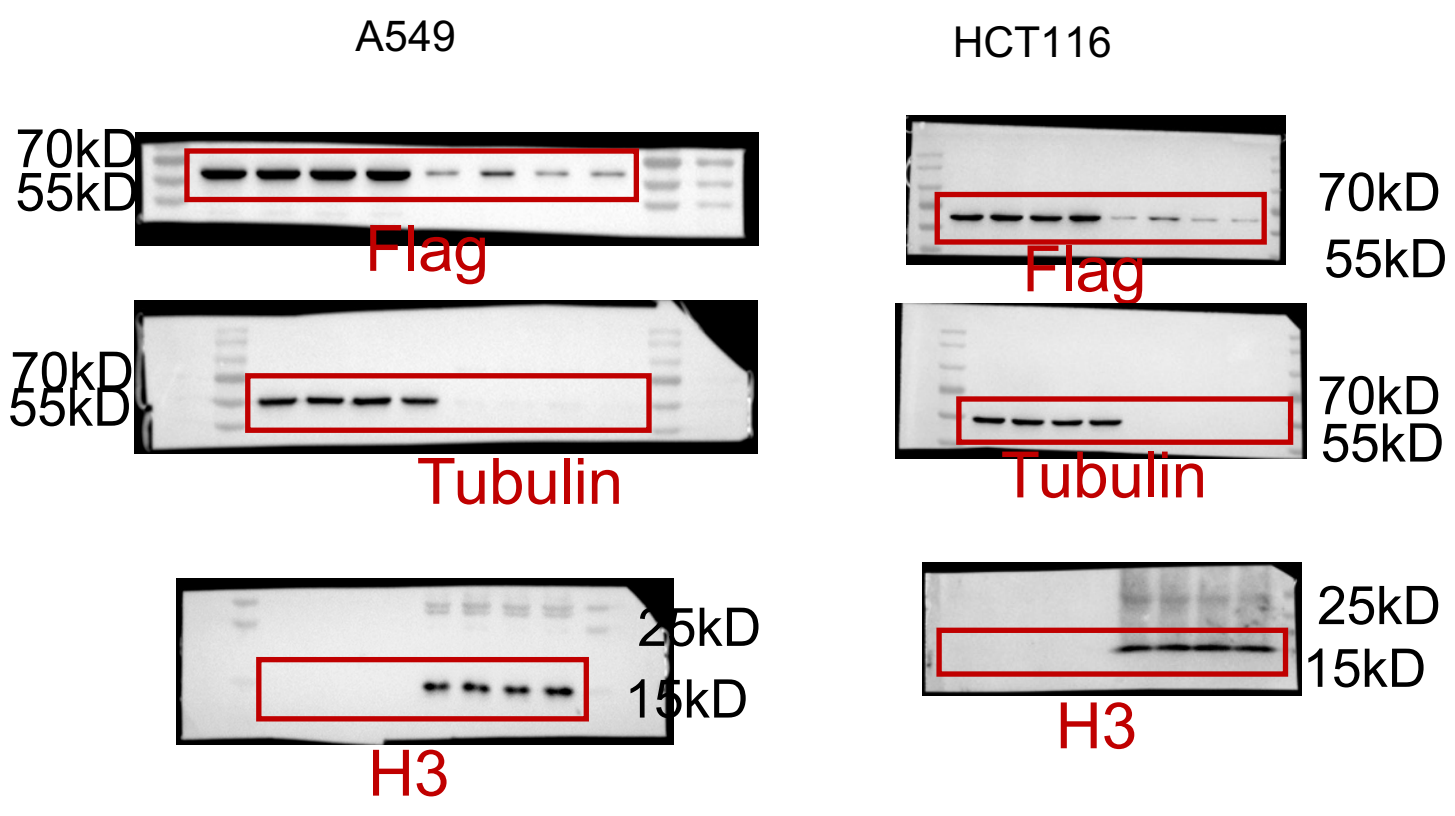

## Supplementary Fig 6c

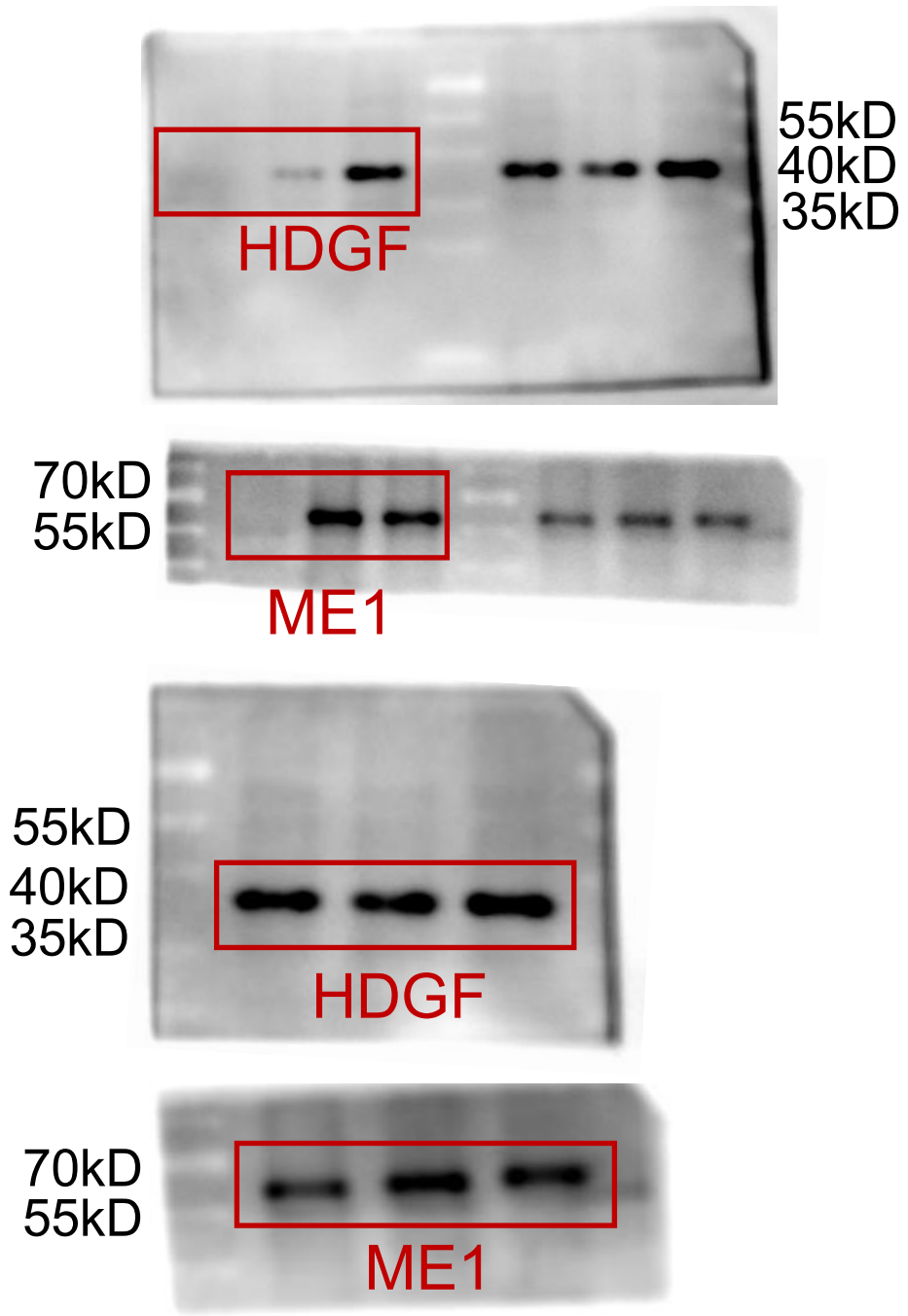


## Supplementary Fig 6d

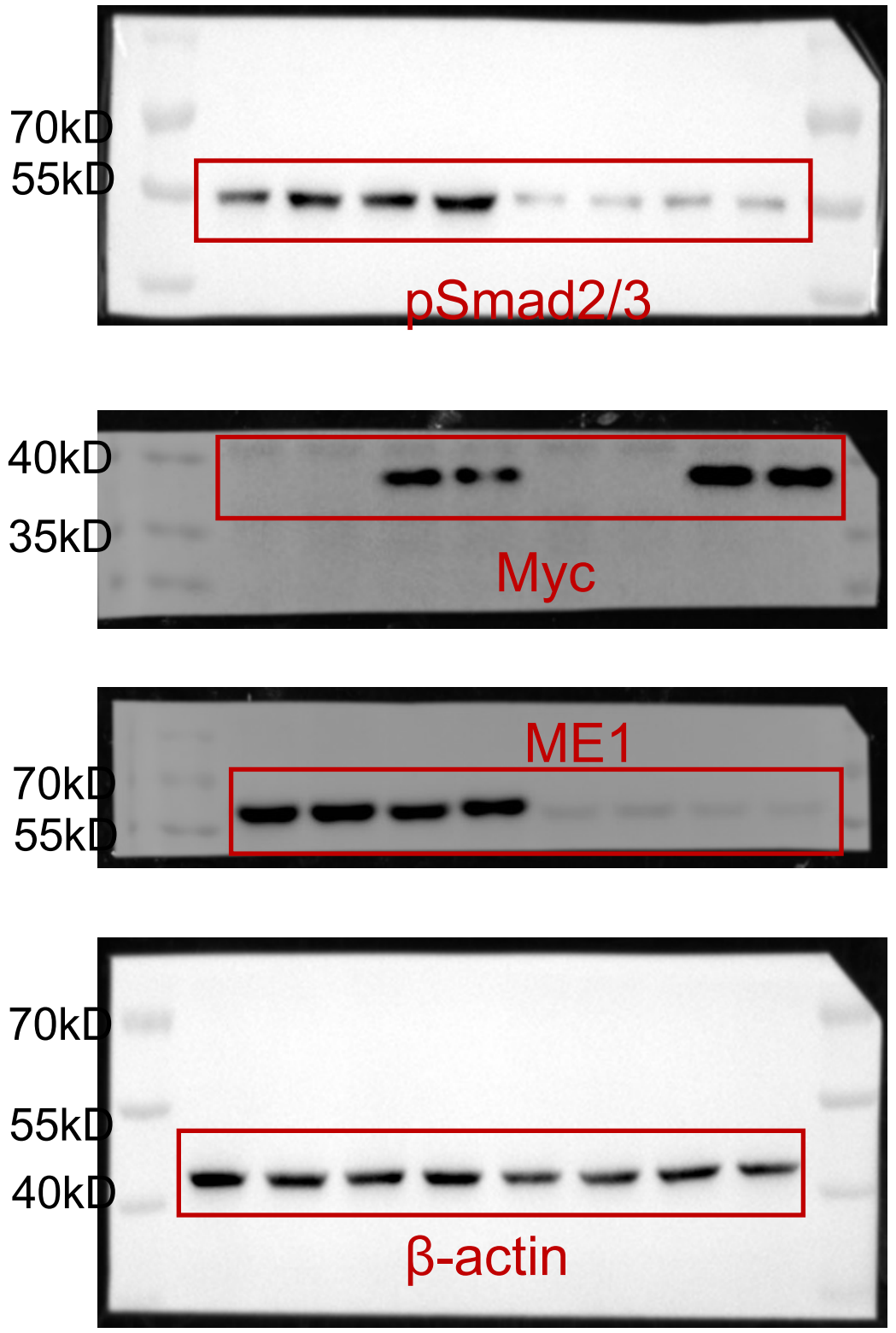

# Supplementary Fig. 7c

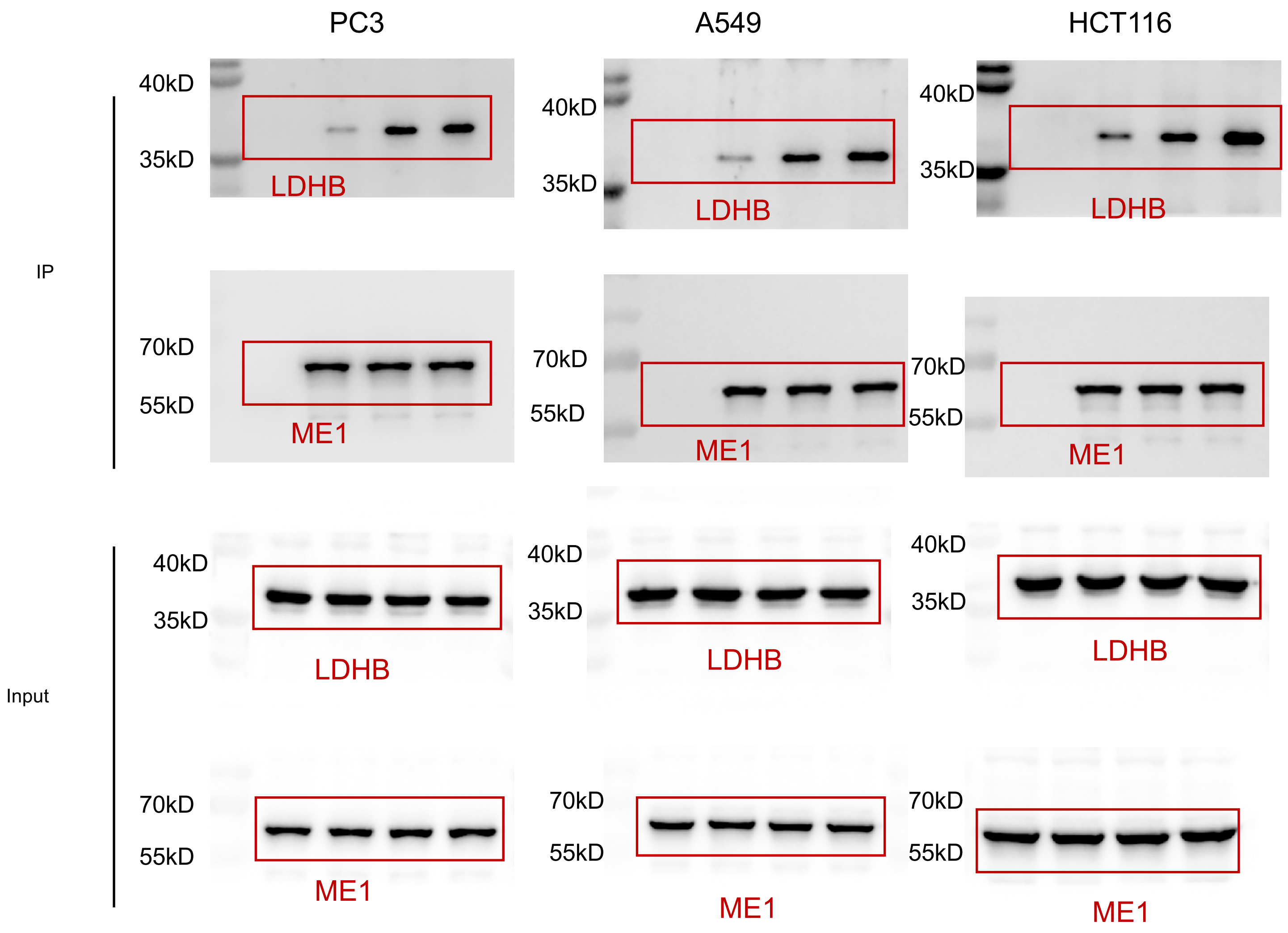

## Supplementary Fig 7d

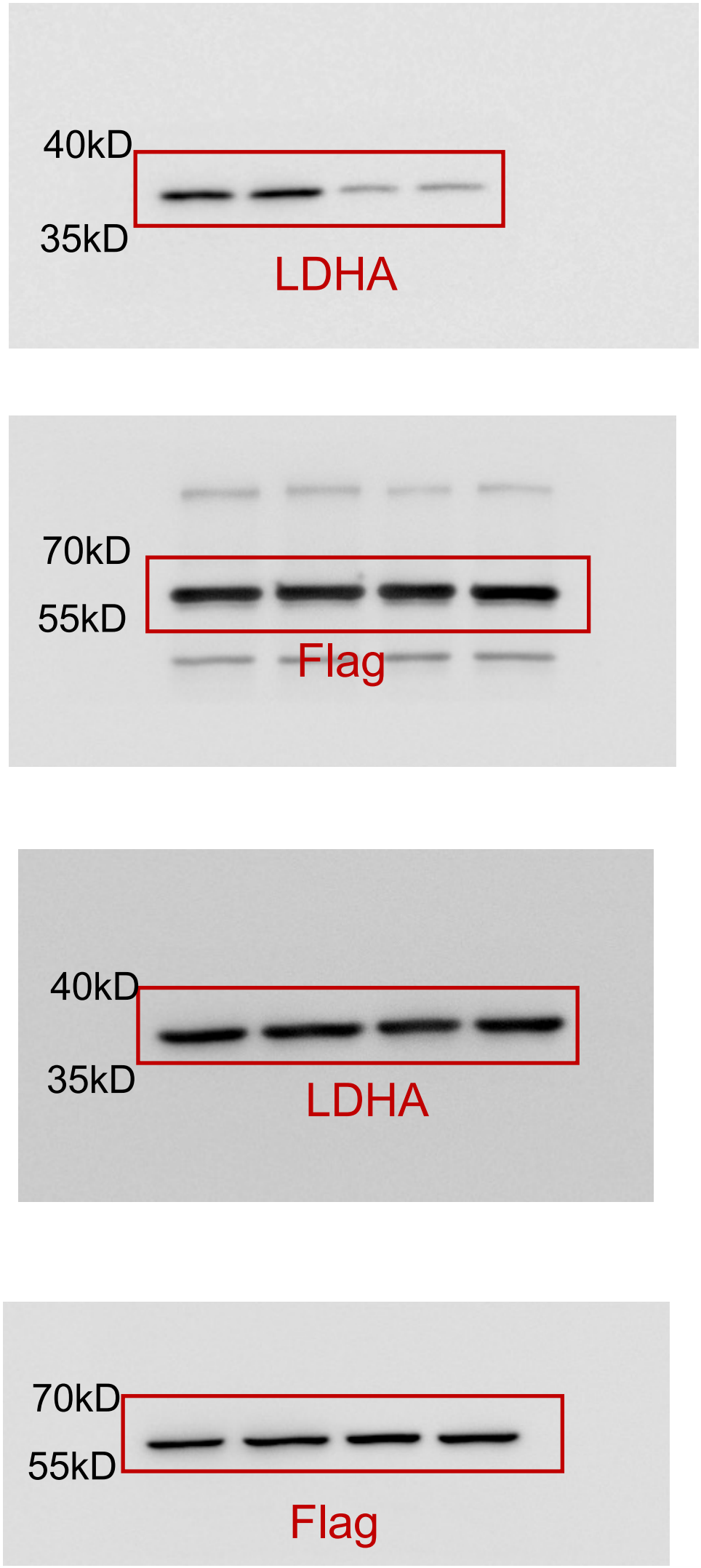


## Supplementary Fig 7e

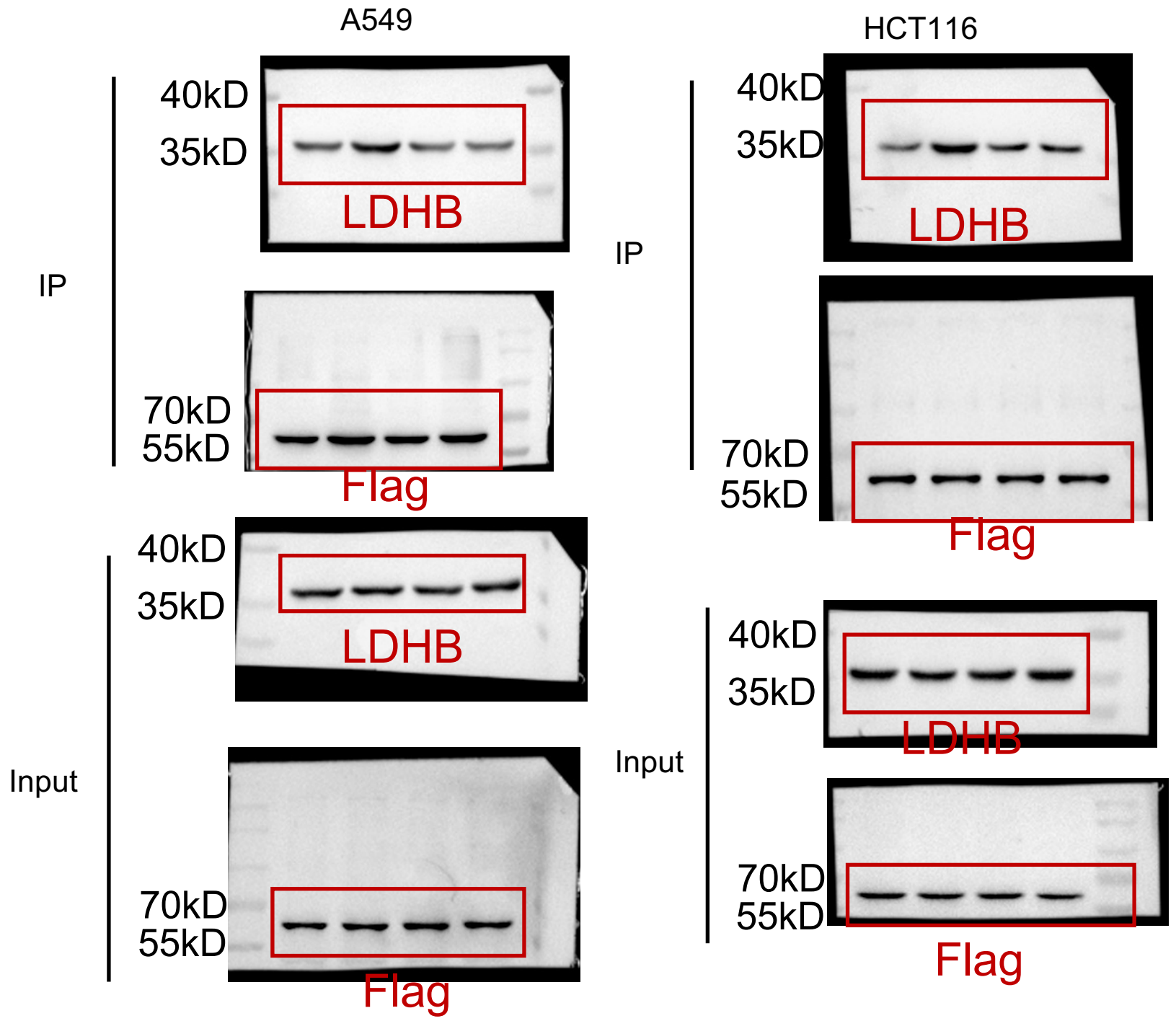

## Supplementary Fig 7f

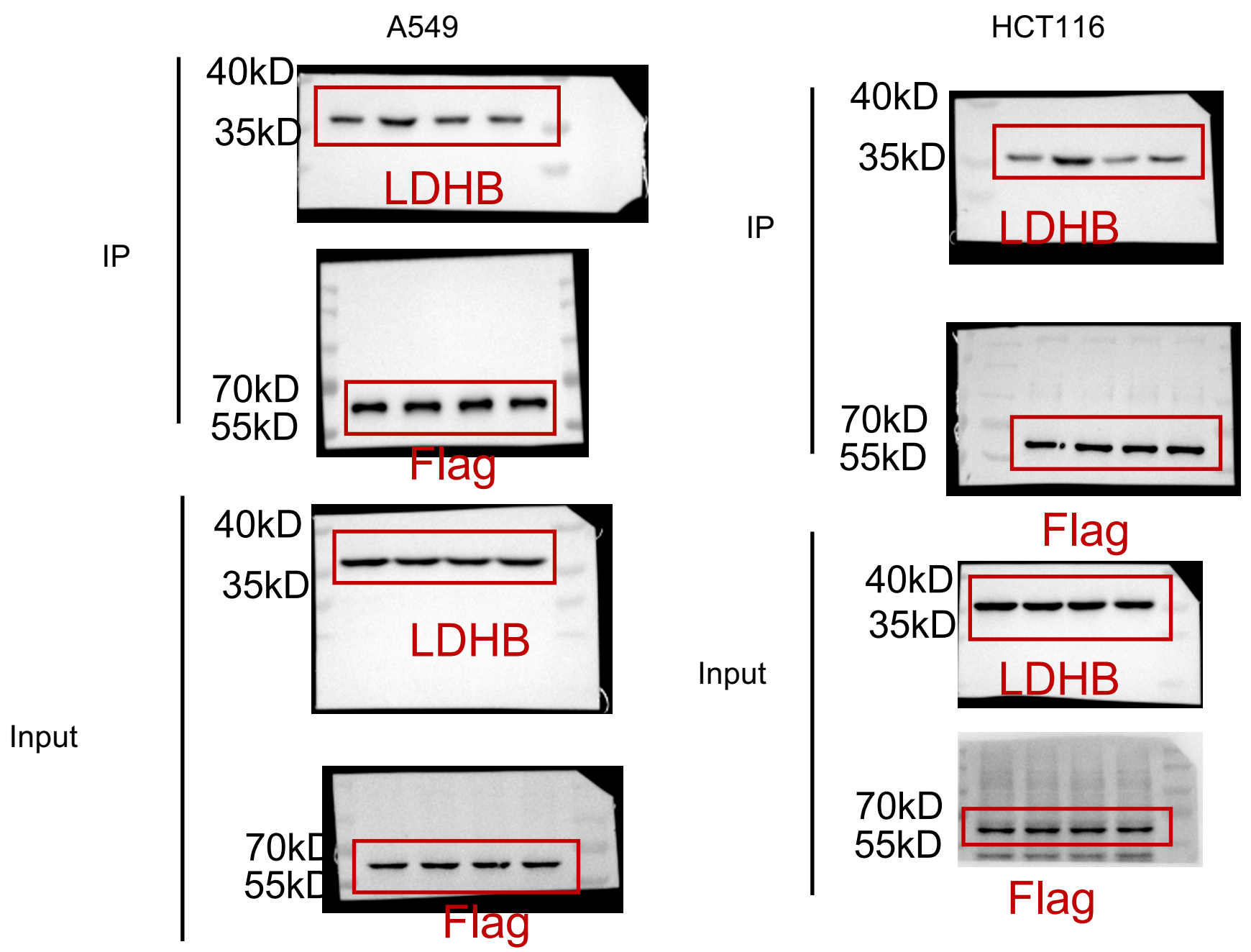


## Supplementary Fig 7g

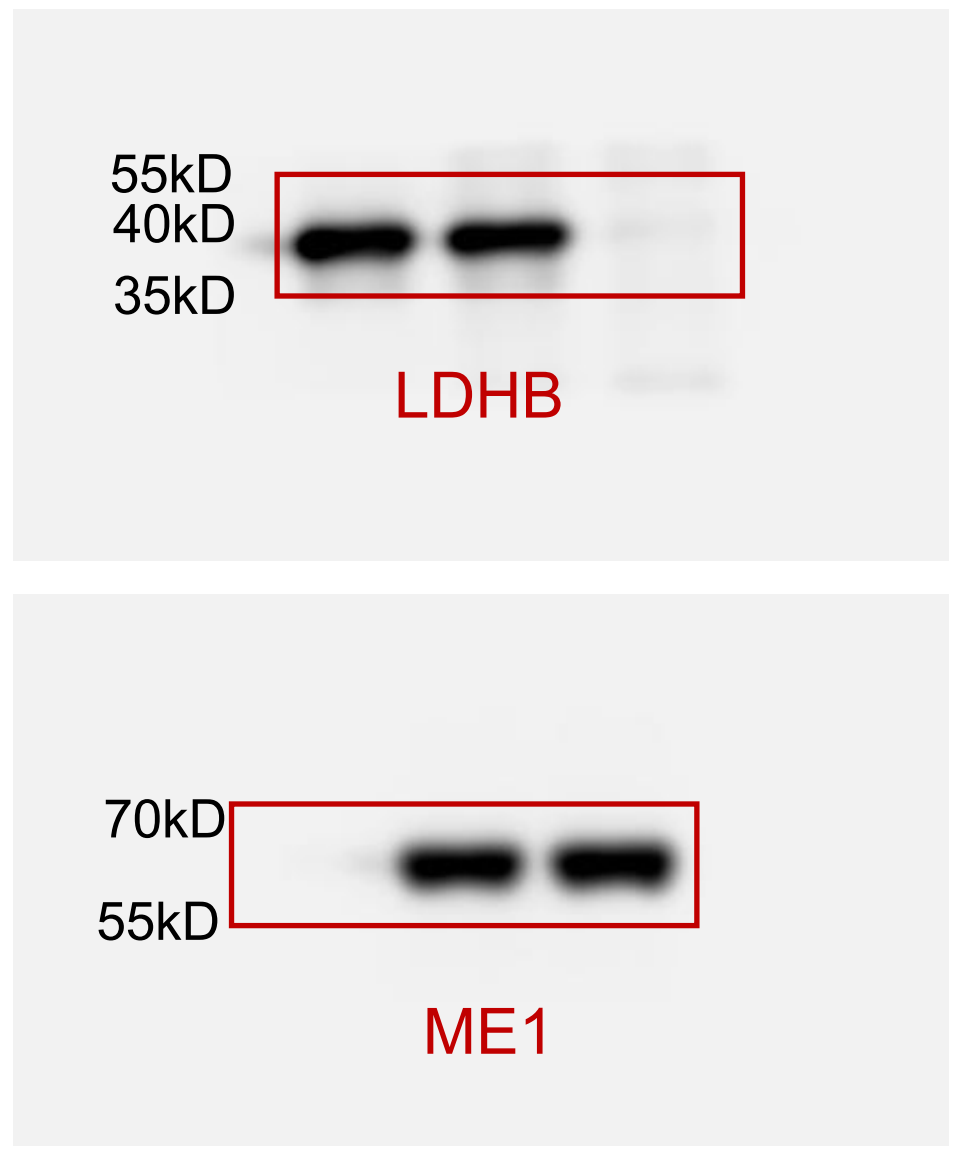


## Supplementary Fig 7i

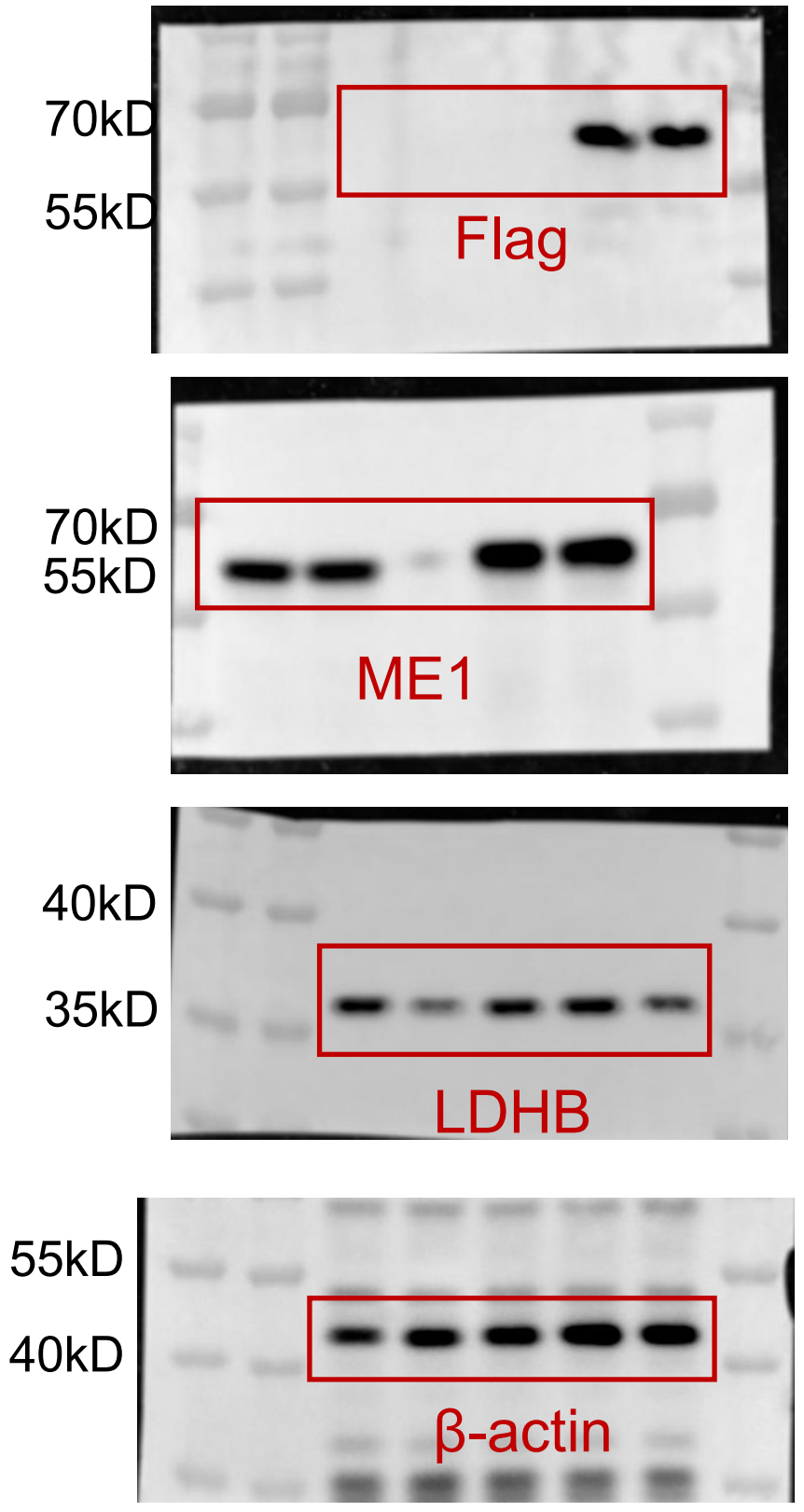

Supplement: Supplementary file 1 [file 41392_2026_2839_MOESM2_ESM.pdf]
