# Supplementary material for: Malic enzyme 1 senses L-lactate to determine tumor heterogeneity
Source: Signal Transduct Target Ther. 2026 Sep 11;11:386. doi: 10.1038/s41392-026-02839-6 (PMC13569829; doi:10.1038/s41392-026-02839-6)
Supplement: Supplementary file 2 [file 41392_2026_2839_MOESM4_ESM.pdf]

Full wwPDB X-ray Structure Validation Report (i)

Feb 13, 2026 – 10:56 PM JST

PDB ID	:	23JW / pdb_000023jw
Title	:	Crystal structure of human cytosolic NADP(+)-dependent malic enzyme in a ternary complex with NADP+ and Mn2+
Deposited on	:	2026-02-09
Resolution	:	2.31 Å(reported)

This wwPDB validation report is for manuscript review

This is a Full wwPDB X-ray Structure Validation Report.

This report is produced by the wwPDB biocuration pipeline after annotation of the structure.

We welcome your comments at *validation@mail.wwpdb.org*
A user guide is available at
https://www.wwpdb.org/validation/2017/XrayValidationReportHelp
with specific help available everywhere you see the (i) symbol.

The types of validation reports are described at
http://www.wwpdb.org/validation/2017/FAQs#types.

For Manuscript Review

The following versions of software and data (see references (i)) were used in the production of this report:

MolProbity	:	4-5-2 with Phenix2.0
Mogul	:	1.8.5 (274361), CSD as541be (2020)
Xtriage (Phenix)	:	2.0
EDS	:	3.0
buster-report	:	1.1.7 (2018)
Percentile statistics	:	20231227.v01 (using entries in the PDB archive December 27th 2023)
CCP4	:	9.0.010 (Gargrove)
Density-Fitness	:	1.0.12
Ideal geometry (proteins)	:	Engh & Huber (2001)

1 Overall quality at a glance (i)

The following experimental techniques were used to determine the structure:
X-RAY DIFFRACTION

The reported resolution of this entry is 2.31 Å.

Percentile scores (ranging between 0-100) for global validation metrics of the entry are shown in the following graphic. The table shows the number of entries on which the scores are based.

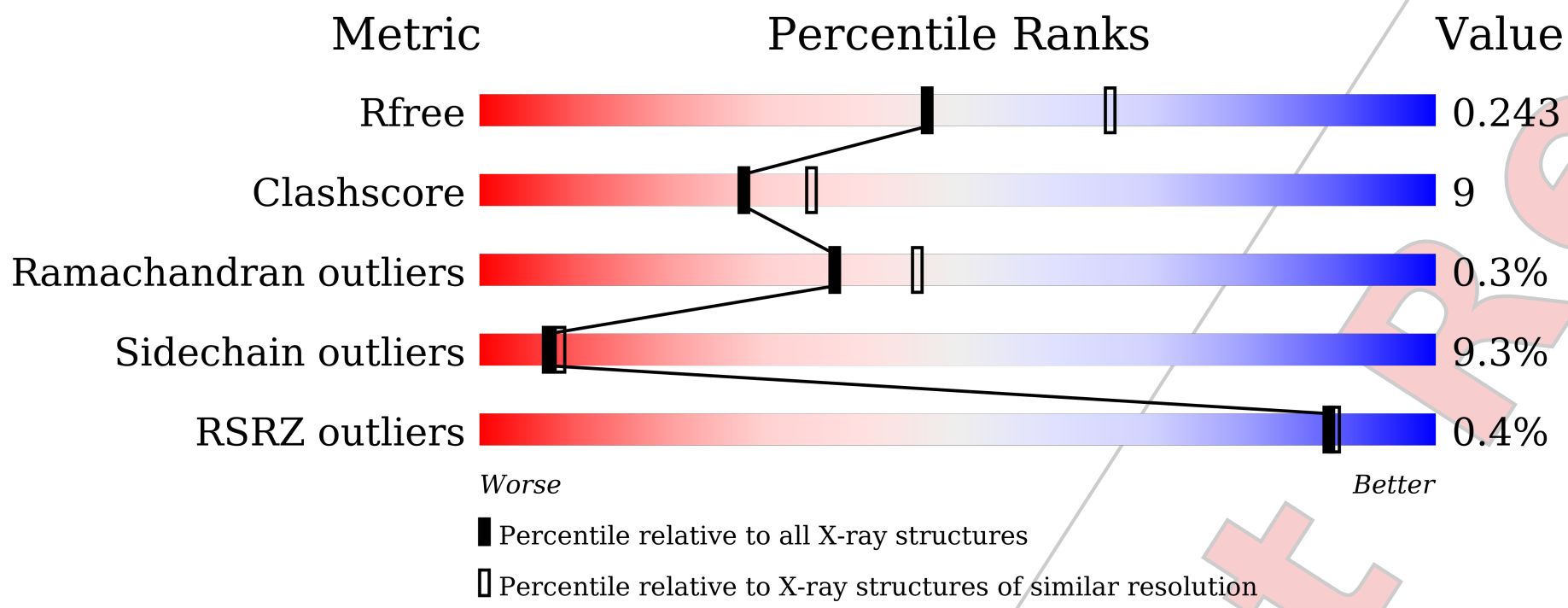

Metric	Whole archive (#Entries)	Similar resolution (#Entries, resolution range(Å))
R_{free}	164625	7250 (2.34-2.30)
Clashscore	180529	8063 (2.34-2.30)
Ramachandran outliers	177936	7993 (2.34-2.30)
Sidechain outliers	177891	7993 (2.34-2.30)
RSRZ outliers	164620	7250 (2.34-2.30)

The table below summarises the geometric issues observed across the polymeric chains and their fit to the electron density. The red, orange, yellow and green segments of the lower bar indicate the fraction of residues that contain outliers for >=3, 2, 1 and 0 types of geometric quality criteria respectively. A grey segment represents the fraction of residues that are not modelled. The numeric value for each fraction is indicated below the corresponding segment, with a dot representing fractions <=5% The upper red bar (where present) indicates the fraction of residues that have poor fit to the electron density. The numeric value is given above the bar.

Mol	Chain	Length	Quality of chain
1	A	572	83% 14% ••
1	B	572	79% 16% • •
1	C	572	% 77% 16% 5% •
1	D	572	% 73% 21% • •

Ideal geometry (DNA, RNA) : Parkinson et al. (1996)
Validation Pipeline (wwPDB-VP) : 2.48

For Manuscript Review

2 Entry composition (i)

There are 4 unique types of molecules in this entry. The entry contains 18216 atoms, of which 0 are hydrogens and 0 are deuteriums.

In the tables below, the ZeroOcc column contains the number of atoms modelled with zero occupancy, the AltConf column contains the number of residues with at least one atom in alternate conformation and the Trace column contains the number of residues modelled with at most 2 atoms.

- Molecule 1 is a protein called NADP-dependent malic enzyme.

Mol	Chain	Residues	Atoms					ZeroOcc	AltConf	Trace
1	A	566	Total 4465	C 2842	N 767	O 836	S 20	0	0	0
1	C	562	Total 4433	C 2823	N 760	O 830	S 20	0	0	0
1	B	562	Total 4433	C 2823	N 760	O 830	S 20	0	0	0
1	D	561	Total 4424	C 2817	N 758	O 829	S 20	0	0	0

- Molecule 2 is NADP NICOTINAMIDE-ADENINE-DINUCLEOTIDE PHOSPHATE (CCD ID: NAP) (formula: $C_{21}H_{28}N_7O_{17}P_3$) (labeled as "Ligand of Interest" by depositor).

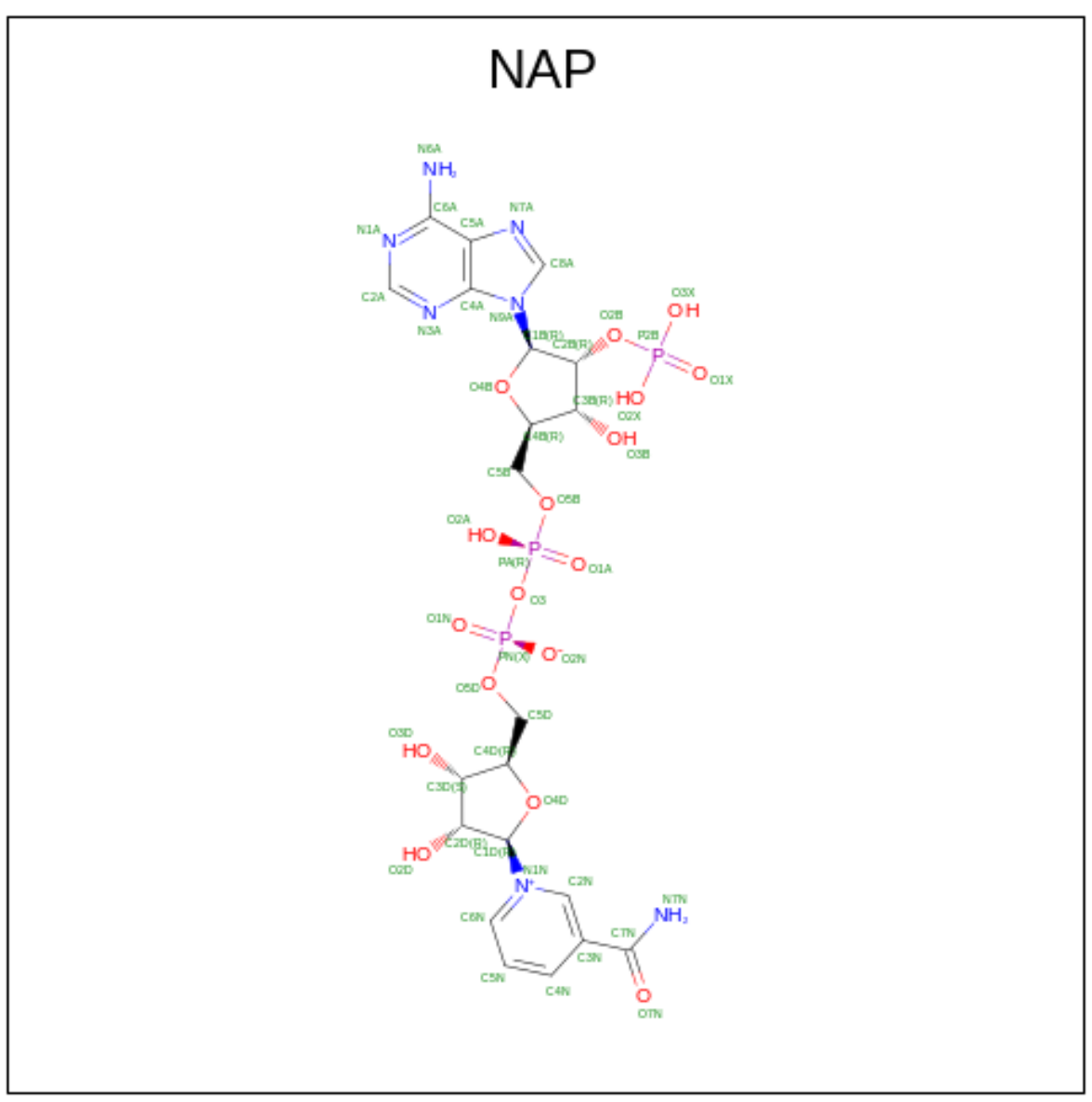

Mol	Chain	Residues	Atoms					ZeroOcc	AltConf
2	A	1	Total 48	C 21	N 7	O 17	P 3	0	0

Continued on next page...

For Manuscript Review

Continued from previous page...

Mol	Chain	Residues	Atoms	ZeroOcc	AltConf
2	C	1	Total C N O P<br>48 21 7 17 3	0	0
2	B	1	Total C N O P<br>48 21 7 17 3	0	0
2	D	1	Total C N O P<br>48 21 7 17 3	0	0

- Molecule 3 is MANGANESE (II) ION (CCD ID: MN) (formula: Mn) (labeled as "Ligand of Interest" by depositor).

Mol	Chain	Residues	Atoms	ZeroOcc	AltConf
3	A	1	Total Mn<br>1 1	0	0
3	C	1	Total Mn<br>1 1	0	0
3	B	1	Total Mn<br>1 1	0	0
3	D	1	Total Mn<br>1 1	0	0

- Molecule 4 is water.

Mol	Chain	Residues	Atoms	ZeroOcc	AltConf
4	A	110	Total O<br>110 110	0	0
4	C	51	Total O<br>51 51	0	0
4	B	45	Total O<br>45 45	0	0
4	D	59	Total O<br>59 59	0	0

For Manuscript Review

3 Residue-property plots (i)

These plots are drawn for all protein, RNA, DNA and oligosaccharide chains in the entry. The first graphic for a chain summarises the proportions of the various outlier classes displayed in the second graphic. The second graphic shows the sequence view annotated by issues in geometry and electron density. Residues are color-coded according to the number of geometric quality criteria for which they contain at least one outlier: green = 0, yellow = 1, orange = 2 and red = 3 or more. A red dot above a residue indicates a poor fit to the electron density (RSRZ > 2). Stretches of 2 or more consecutive residues without any outlier are shown as a green connector. Residues present in the sample, but not in the model, are shown in grey.

• Molecule 1: NADP-dependent malic enzyme

Chain A:

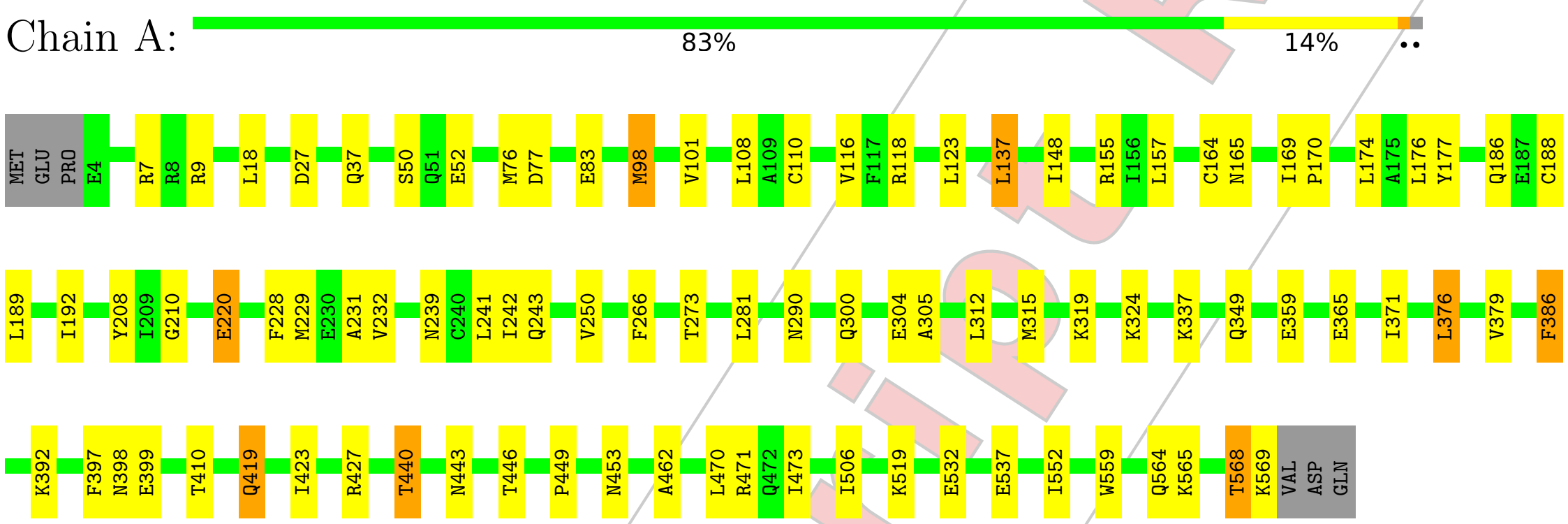

• Molecule 1: NADP-dependent malic enzyme

Chain C:

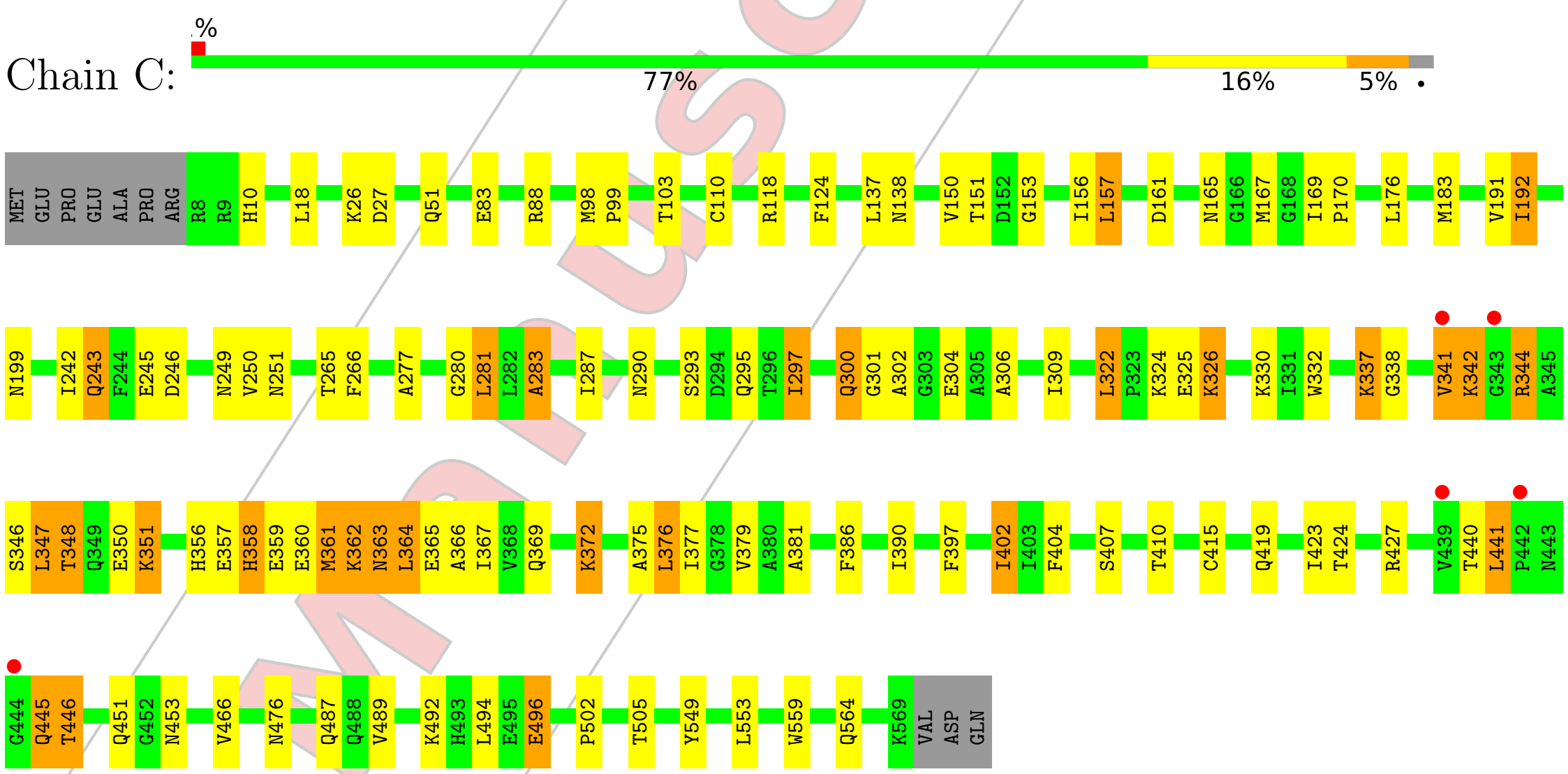

• Molecule 1: NADP-dependent malic enzyme

Chain B:

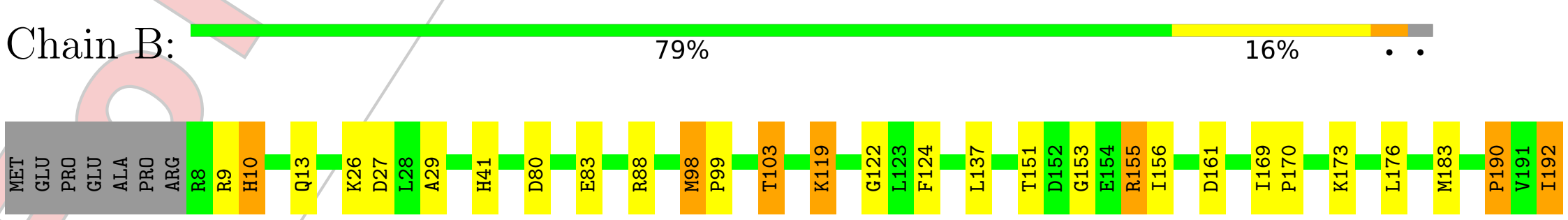

For Manuscript Review

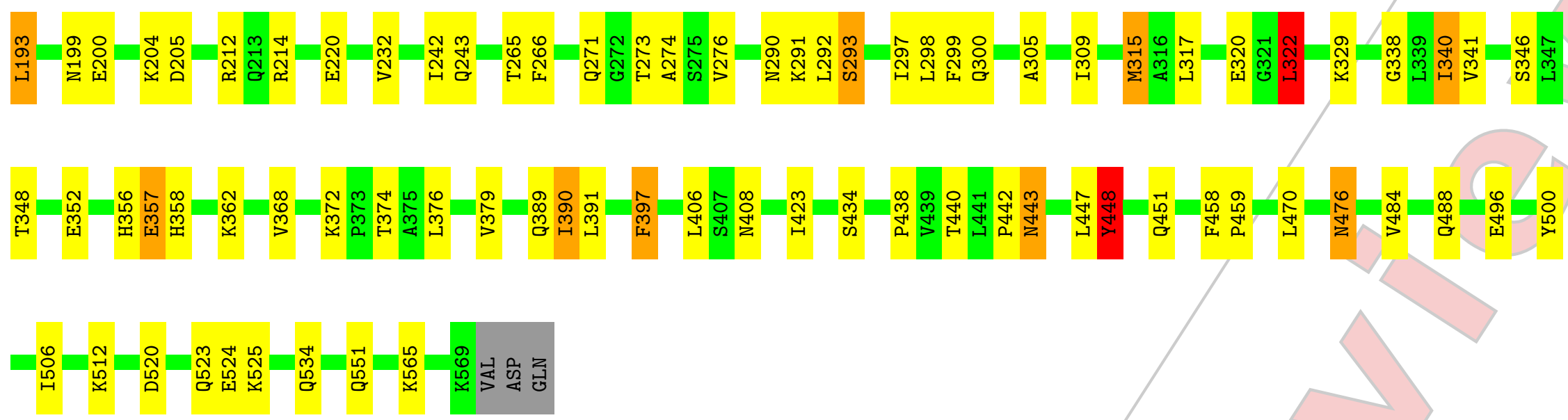

• Molecule 1: NADP-dependent malic enzyme

Chain D:

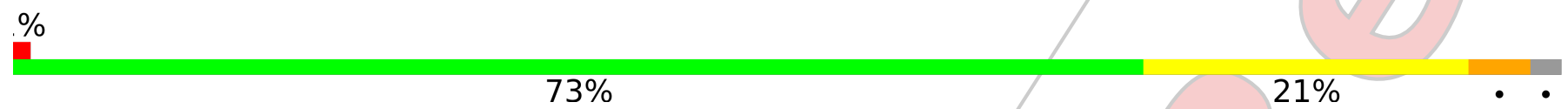

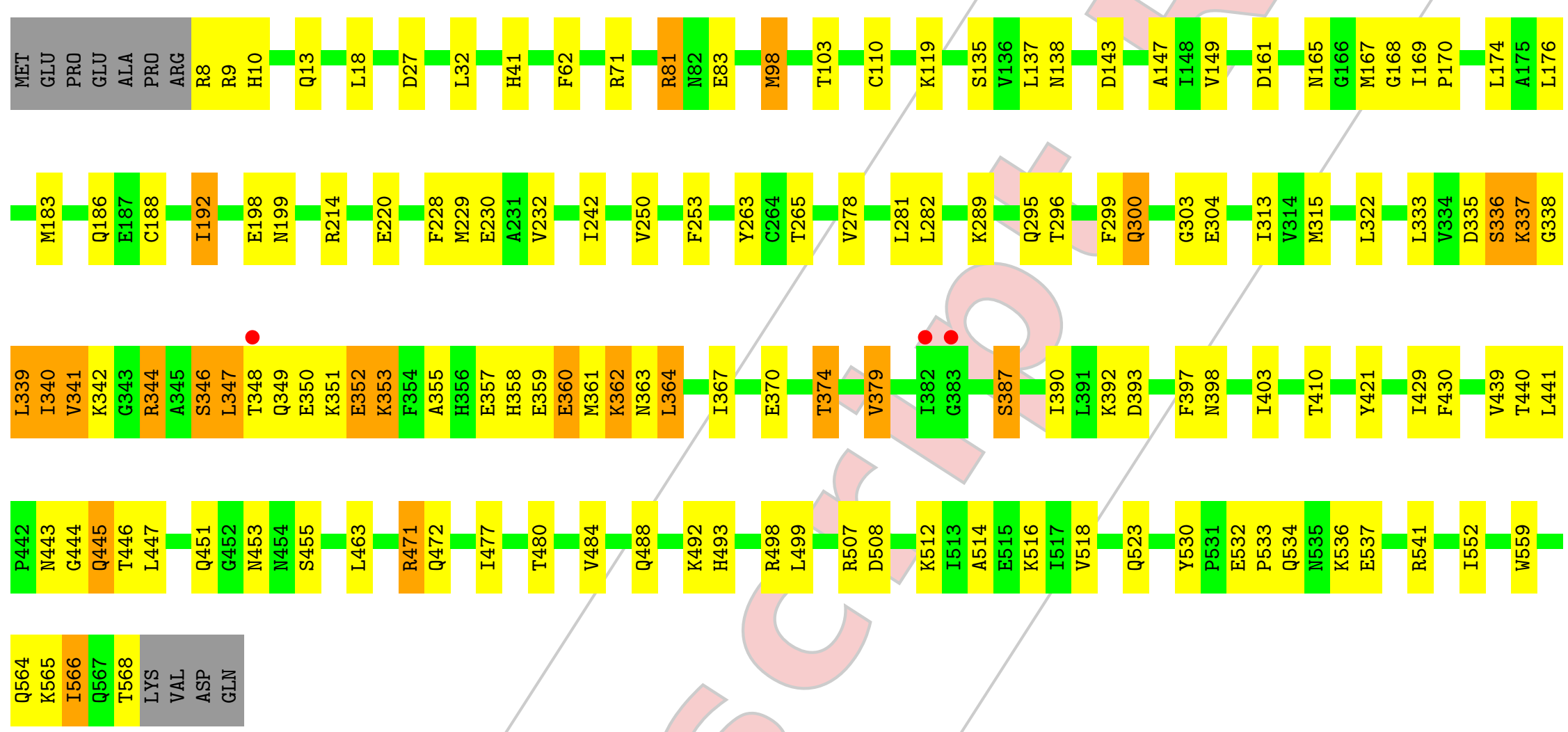

For Manuscript Review

4 Data and refinement statistics (i)

Property	Value	Source
Space group	P 1 21 1	Depositor
Cell constants<br>a, b, c, α, β, γ	70.71Å 181.88Å 108.80Å<br>90.00° 105.33° 90.00°	Depositor
Resolution (Å)	27.02 – 2.31<br>27.02 – 2.31	Depositor<br>EDS
% Data completeness<br>(in resolution range)	99.9 (27.02-2.31)<br>99.9 (27.02-2.31)	Depositor<br>EDS
R_{merge}	0.06	Depositor
R_{sym}	(Not available)	Depositor
$< I/\sigma(I) >$ [1]	2.08 (at 2.31Å)	Xtriage
Refinement program	REFMAC 5.8.0258	Depositor
R, R_{free}	0.175 , 0.239<br>0.183 , 0.243	Depositor<br>DCC
R_{free} test set	5877 reflections (5.08%)	wwPDB-VP
Wilson B-factor (Å^2)	67.2	Xtriage
Anisotropy	0.014	Xtriage
Bulk solvent k_{sol}(e/Å^3), B_{sol}(Å^2)	0.34 , 49.2	EDS
L-test for twinning[2]	$< \|L\| > = 0.50$, $< L^2 > = 0.33$	Xtriage
Estimated twinning fraction	No twinning to report.	Xtriage
F_o,F_c correlation	0.97	EDS
Total number of atoms	18216	wwPDB-VP
Average B, all atoms (Å^2)	80.0	wwPDB-VP

Xtriage's analysis on translational NCS is as follows: *The largest off-origin peak in the Patterson function is 3.38% of the height of the origin peak. No significant pseudotranslation is detected.*

[1] Intensities estimated from amplitudes.

[2] Theoretical values of $< |L| >$, $< L^2 >$ for acentric reflections are 0.5, 0.333 respectively for untwinned datasets, and 0.375, 0.2 for perfectly twinned datasets.

For Manuscript Review

5 Model quality (i)

5.1 Standard geometry (i)

Bond lengths and bond angles in the following residue types are not validated in this section: MN, NAP

The Z score for a bond length (or angle) is the number of standard deviations the observed value is removed from the expected value. A bond length (or angle) with $|Z| > 5$ is considered an outlier worth inspection. RMSZ is the root-mean-square of all Z scores of the bond lengths (or angles).

Mol	Chain	Bond lengths		Bond angles	
		RMSZ	#|Z| >5	RMSZ	#|Z| >5
1	A	1.11	2/4552 (0.0%)	1.48	13/6162 (0.2%)
1	B	1.13	5/4519 (0.1%)	1.55	17/6117 (0.3%)
1	C	1.06	0/4519	1.46	8/6117 (0.1%)
1	D	1.09	2/4510 (0.0%)	1.44	7/6106 (0.1%)
All	All	1.10	9/18100 (0.0%)	1.48	45/24502 (0.2%)

All (9) bond length outliers are listed below:

Mol	Chain	Res	Type	Atoms	Z	Observed(Å)	Ideal(Å)
1	B	448	TYR	C-N	13.55	1.50	1.33
1	A	532	GLU	C-N	12.90	1.50	1.33
1	B	322	LEU	C-N	12.86	1.50	1.33
1	B	103	THR	C-N	8.02	1.49	1.34
1	B	41	HIS	CE1-NE2	6.04	1.38	1.32
1	D	41	HIS	CE1-NE2	5.90	1.38	1.32
1	B	190	PRO	C-O	-5.32	1.18	1.23
1	A	449	PRO	C-O	-5.19	1.17	1.23
1	D	168	GLY	C-O	5.03	1.30	1.23

All (45) bond angle outliers are listed below:

Mol	Chain	Res	Type	Atoms	Z	Observed(^{o})	Ideal(^{o})
1	B	322	LEU	CA-C-N	16.36	136.58	119.89
1	B	322	LEU	C-N-CA	16.36	136.58	119.89
1	B	448	TYR	CA-C-N	14.30	135.30	120.14
1	B	448	TYR	C-N-CA	14.30	135.30	120.14
1	A	532	GLU	CA-C-N	14.25	134.25	119.85
1	A	532	GLU	C-N-CA	14.25	134.25	119.85
1	C	27	ASP	CA-CB-CG	8.77	121.37	112.60
1	B	27	ASP	CA-CB-CG	8.06	120.66	112.60

Continued on next page...

For Manuscript Review

Continued from previous page...

Mol	Chain	Res	Type	Atoms	Z	Observed(°)	Ideal(°)
1	B	27	ASP	CB-CA-C	-7.22	108.26	116.63
1	A	27	ASP	CB-CA-C	-6.97	108.53	116.54
1	C	301	GLY	CA-C-O	-6.88	117.75	122.22
1	C	496	GLU	N-CA-C	-6.48	103.65	113.89
1	A	398	ASN	CA-C-N	6.46	133.88	121.54
1	A	398	ASN	C-N-CA	6.46	133.88	121.54
1	D	81	ARG	CB-CA-C	6.26	120.17	109.65
1	D	27	ASP	CA-CB-CG	6.25	118.85	112.60
1	A	397	PHE	CB-CA-C	-6.23	98.81	109.65
1	B	10	HIS	CA-C-O	-6.14	114.72	122.63
1	B	88	ARG	N-CA-C	-5.90	104.93	111.36
1	A	83	GLU	CB-CG-CD	5.80	122.46	112.60
1	C	283	ALA	CA-C-N	5.79	128.04	120.28
1	C	283	ALA	C-N-CA	5.79	128.04	120.28
1	B	83	GLU	CB-CG-CD	5.78	122.43	112.60
1	D	103	THR	CA-CB-OG1	-5.73	101.00	109.60
1	B	299	PHE	CB-CA-C	-5.69	100.86	110.19
1	B	292	LEU	CA-C-N	5.68	128.16	120.38
1	B	292	LEU	C-N-CA	5.68	128.16	120.38
1	A	77	ASP	CA-CB-CG	5.62	118.22	112.60
1	A	231	ALA	CA-C-N	5.62	127.75	120.56
1	A	231	ALA	C-N-CA	5.62	127.75	120.56
1	D	530	TYR	CB-CA-C	5.57	117.26	108.84
1	A	27	ASP	CA-CB-CG	5.44	118.04	112.60
1	C	466	VAL	CA-C-O	-5.42	115.42	121.17
1	D	10	HIS	CA-C-O	-5.37	116.19	122.37
1	B	214	ARG	CA-C-O	-5.29	115.57	121.81
1	B	551	GLN	CB-CA-C	5.28	118.46	109.80
1	B	338	GLY	CA-C-N	5.14	128.15	120.90
1	B	338	GLY	C-N-CA	5.14	128.15	120.90
1	B	119	LYS	CB-CA-C	5.11	116.03	110.15
1	A	386	PHE	CA-CB-CG	5.10	118.90	113.80
1	A	440	THR	CB-CA-C	5.06	118.39	110.19
1	C	138	ASN	CA-CB-CG	5.05	117.65	112.60
1	D	336	SER	N-CA-C	-5.04	106.39	112.54
1	D	13	GLN	CB-CA-C	5.03	118.06	109.72
1	C	83	GLU	CB-CG-CD	5.00	121.11	112.60

There are no chirality outliers.

There are no planarity outliers.

For Manuscript Review

5.2 Too-close contacts (i)

In the following table, the Non-H and H(model) columns list the number of non-hydrogen atoms and hydrogen atoms in the chain respectively. The H(added) column lists the number of hydrogen atoms added and optimized by MolProbity. The Clashes column lists the number of clashes within the asymmetric unit, whereas Symm-Clashes lists symmetry-related clashes.

Mol	Chain	Non-H	H(model)	H(added)	Clashes	Symm-Clashes
1	A	4465	0	4493	51	0
1	B	4433	0	4462	59	0
1	C	4433	0	4462	108	0
1	D	4424	0	4449	103	0
2	A	48	0	25	6	0
2	B	48	0	25	2	0
2	C	48	0	25	3	0
2	D	48	0	25	5	0
3	A	1	0	0	0	0
3	B	1	0	0	0	0
3	C	1	0	0	0	0
3	D	1	0	0	0	0
4	A	110	0	0	3	0
4	B	45	0	0	0	0
4	C	51	0	0	0	0
4	D	59	0	0	1	0
All	All	18216	0	17966	319	0

The all-atom clashscore is defined as the number of clashes found per 1000 atoms (including hydrogen atoms). The all-atom clashscore for this structure is 9.

All (319) close contacts within the same asymmetric unit are listed below, sorted by their clash magnitude.

Atom-1	Atom-2	Interatomic distance (Å)	Clash overlap (Å)
1:C:348:THR:HG22	1:C:351:LYS:CB	1.74	1.16
1:C:348:THR:CG2	1:C:351:LYS:HB2	1.77	1.14
1:C:348:THR:CG2	1:C:351:LYS:H	1.67	1.06
1:D:339:LEU:H	1:D:339:LEU:HD22	1.28	0.99
1:C:363:ASN:HD21	1:C:366:ALA:H	1.09	0.99
1:A:379:VAL:HG22	2:A:601:NAP:H51N	1.44	0.98
1:C:441:LEU:H	1:C:441:LEU:HD12	1.29	0.96
1:D:300:GLN:HE22	2:D:601:NAP:H2A	1.31	0.96
1:B:80:ASP:OD2	1:B:119:LYS:HE3	1.65	0.95
1:C:348:THR:HG22	1:C:351:LYS:HB2	0.97	0.94
1:C:441:LEU:HD12	1:C:445:GLN:O	1.69	0.93

Continued on next page...

For Manuscript Review

Continued from previous page...

Atom-1	Atom-2	Interatomic distance (Å)	Clash overlap (Å)
1:D:362:LYS:H	1:D:362:LYS:HD3	1.34	0.92
1:C:348:THR:HG22	1:C:351:LYS:N	1.84	0.91
1:A:9:ARG:HH22	1:A:186:GLN:NE2	1.69	0.90
1:C:348:THR:CG2	1:C:351:LYS:N	2.35	0.89
1:D:441:LEU:HD12	1:D:445:GLN:HB3	1.54	0.89
1:C:348:THR:HG22	1:C:351:LYS:H	1.31	0.87
1:B:322:LEU:HD12	1:B:322:LEU:H	1.38	0.86
1:C:440:THR:HG22	1:C:446:THR:HG23	1.57	0.86
1:D:300:GLN:HE22	2:D:601:NAP:C2A	1.90	0.84
1:C:363:ASN:ND2	1:C:366:ALA:H	1.74	0.84
1:B:151:THR:HG23	1:B:192:ILE:HD13	1.59	0.84
1:D:440:THR:HG22	1:D:446:THR:HG22	1.58	0.84
1:D:362:LYS:HD3	1:D:362:LYS:N	1.94	0.82
1:D:443:ASN:O	1:D:443:ASN:ND2	2.13	0.81
1:A:9:ARG:HH22	1:A:186:GLN:HE22	1.28	0.81
1:C:361:MET:HE2	1:C:361:MET:O	1.81	0.80
1:C:338:GLY:HA3	1:C:361:MET:HG2	1.63	0.80
1:B:447:LEU:CD2	1:B:496:GLU:O	2.30	0.79
1:A:228:PHE:CD2	1:A:229:MET:HE2	2.19	0.78
1:C:348:THR:HG23	1:C:351:LYS:H	1.46	0.78
1:B:447:LEU:HD23	1:B:496:GLU:O	1.82	0.78
1:C:441:LEU:CD1	1:C:445:GLN:HG2	2.14	0.78
1:C:441:LEU:CD1	1:C:445:GLN:O	2.31	0.77
1:B:322:LEU:HD12	1:B:322:LEU:N	1.99	0.77
1:D:228:PHE:CD2	1:D:229:MET:HE2	2.19	0.77
1:C:348:THR:HG22	1:C:351:LYS:CA	2.15	0.77
1:C:322:LEU:HD12	1:C:322:LEU:H	1.51	0.75
1:B:317:LEU:O	1:B:322:LEU:CD1	2.35	0.75
1:C:322:LEU:HD12	1:C:322:LEU:N	2.03	0.73
1:C:372:LYS:CG	1:C:397:PHE:CD1	2.72	0.73
1:C:441:LEU:HD12	1:C:441:LEU:N	2.03	0.73
1:A:559:TRP:H	1:A:564:GLN:NE2	1.87	0.72
1:C:440:THR:HG22	1:C:446:THR:CG2	2.18	0.72
1:A:305:ALA:HB3	1:A:379:VAL:HG21	1.70	0.72
1:A:379:VAL:CG2	2:A:601:NAP:H51N	2.20	0.72
1:D:339:LEU:N	1:D:339:LEU:HD13	2.04	0.72
1:C:365:GLU:HG2	1:C:390:ILE:HG12	1.70	0.72
1:A:305:ALA:CB	1:A:379:VAL:HG21	2.20	0.71
1:D:340:ILE:N	1:D:340:ILE:HD13	2.04	0.71
1:C:372:LYS:HG3	1:C:397:PHE:CD1	2.25	0.71
1:B:151:THR:CG2	1:B:192:ILE:HD13	2.21	0.71

Continued on next page...

For Manuscript Review

Continued from previous page...

Atom-1	Atom-2	Interatomic distance (Å)	Clash overlap (Å)
1:D:228:PHE:CD2	1:D:229:MET:CE	2.75	0.69
1:C:440:THR:CG2	1:C:446:THR:HG23	2.21	0.69
1:A:9:ARG:NH2	1:A:186:GLN:HE22	1.90	0.69
1:A:440:THR:HG22	1:A:446:THR:OG1	1.93	0.68
1:B:80:ASP:OD2	1:B:119:LYS:CE	2.40	0.68
1:D:339:LEU:HD22	1:D:339:LEU:N	2.03	0.68
1:D:348:THR:O	1:D:352:GLU:HG3	1.93	0.68
1:C:363:ASN:HD21	1:C:366:ALA:N	1.87	0.68
1:C:361:MET:HE2	1:C:361:MET:C	2.19	0.68
1:A:273:THR:HG23	2:A:601:NAP:H4N	1.76	0.67
1:C:441:LEU:HD11	1:C:445:GLN:HG2	1.75	0.67
1:D:110:CYS:O	1:D:165:ASN:HB3	1.94	0.67
1:D:337:LYS:HE2	1:D:346:SER:OG	1.95	0.67
1:C:424:THR:O	1:C:427:ARG:HG3	1.95	0.66
1:B:155:ARG:NH2	2:B:601:NAP:O1N	2.28	0.66
1:B:357:GLU:O	1:B:357:GLU:HG3	1.97	0.65
1:C:300:GLN:HE21	1:C:300:GLN:HA	1.60	0.65
1:B:340:ILE:N	1:B:340:ILE:HD13	2.11	0.65
1:B:317:LEU:O	1:B:322:LEU:HD11	1.97	0.64
1:C:372:LYS:HG3	1:C:397:PHE:CE1	2.33	0.64
1:A:155:ARG:NH2	2:A:601:NAP:O1N	2.31	0.64
1:C:153:GLY:HA2	1:C:156:ILE:HD11	1.80	0.64
1:D:341:VAL:HG21	1:D:358:HIS:O	1.98	0.63
1:C:364:LEU:HA	1:C:367:ILE:HG13	1.82	0.62
1:D:9:ARG:HH22	1:D:186:GLN:HE22	1.47	0.62
1:C:441:LEU:HD13	1:C:445:GLN:HG2	1.80	0.61
1:C:376:LEU:HD13	1:C:386:PHE:CZ	2.36	0.61
1:A:148:ILE:HG12	1:A:189:LEU:HB3	1.83	0.61
1:D:9:ARG:HH12	1:D:186:GLN:NE2	1.97	0.61
1:B:438:PRO:CG	1:B:448:TYR:HD2	2.14	0.61
1:C:360:GLU:OE1	1:C:367:ILE:HA	2.01	0.60
1:B:293:SER:HB2	1:B:320:GLU:OE2	2.02	0.60
1:D:421:TYR:HD2	1:D:439:VAL:HG21	1.66	0.60
1:A:228:PHE:CD2	1:A:229:MET:CE	2.86	0.59
1:C:441:LEU:H	1:C:441:LEU:CD1	2.09	0.59
1:C:337:LYS:O	1:C:344:ARG:NH1	2.35	0.59
1:D:296:THR:H	1:D:374:THR:CG2	2.16	0.59
1:D:228:PHE:HD2	1:D:229:MET:CE	2.14	0.59
1:C:342:LYS:N	1:C:356:HIS:O	2.24	0.59
1:C:375:ALA:HB2	1:C:402:ILE:HD12	1.85	0.59
1:C:322:LEU:H	1:C:322:LEU:CD1	2.14	0.59

Continued on next page...

For Manuscript Review

Continued from previous page...

Atom-1	Atom-2	Interatomic distance (Å)	Clash overlap (Å)
1:A:116:VAL:O	1:A:118:ARG:HD3	2.04	0.58
1:B:317:LEU:O	1:B:322:LEU:HD12	2.03	0.58
1:C:243:GLN:HG3	1:C:266:PHE:CZ	2.39	0.58
1:C:337:LYS:HE2	2:C:601:NAP:O2X	2.04	0.57
1:C:492:LYS:O	1:C:496:GLU:HG3	2.04	0.57
1:D:169:ILE:HB	1:D:170:PRO:HD3	1.85	0.57
1:D:229:MET:HE1	1:D:242:ILE:HG21	1.85	0.57
1:B:124:PHE:CD2	1:B:192:ILE:HG21	2.39	0.57
1:B:438:PRO:HG3	1:B:448:TYR:HB3	1.85	0.57
1:D:338:GLY:H	1:D:344:ARG:NH1	2.02	0.57
1:D:300:GLN:NE2	2:D:601:NAP:H2A	2.10	0.57
1:C:338:GLY:CA	1:C:361:MET:HG2	2.33	0.56
1:B:329:LYS:HA	1:B:356:HIS:CE1	2.40	0.56
1:D:347:LEU:HD23	1:D:352:GLU:HA	1.87	0.56
1:D:361:MET:HE2	1:D:363:ASN:HB2	1.85	0.56
1:D:167:MET:HE2	1:D:192:ILE:HG22	1.87	0.56
1:D:379:VAL:O	1:D:379:VAL:HG13	2.05	0.56
1:D:363:ASN:O	1:D:367:ILE:HG12	2.06	0.56
1:B:169:ILE:HB	1:B:170:PRO:HD3	1.86	0.56
1:D:295:GLN:HA	1:D:374:THR:HG21	1.86	0.56
1:D:421:TYR:CE1	1:D:447:LEU:HD13	2.40	0.56
1:B:372:LYS:HE3	1:B:397:PHE:CD1	2.41	0.56
1:D:362:LYS:O	1:D:362:LYS:HG2	2.05	0.56
1:D:333:LEU:HB2	1:D:340:ILE:HG12	1.87	0.56
1:D:18:LEU:C	1:D:18:LEU:HD23	2.31	0.55
1:D:484:VAL:O	1:D:488:GLN:HG2	2.06	0.55
1:B:438:PRO:HB3	1:B:448:TYR:CD2	2.41	0.55
1:A:228:PHE:CE2	1:A:229:MET:HE2	2.41	0.55
1:C:423:ILE:O	1:C:423:ILE:HG22	2.06	0.55
1:D:250:VAL:HG12	4:D:752:HOH:O	2.06	0.55
1:A:273:THR:HG23	2:A:601:NAP:C4N	2.37	0.55
1:D:387:SER:H	1:D:390:ILE:HD13	1.72	0.55
1:D:339:LEU:HD11	1:D:361:MET:O	2.07	0.55
1:C:341:VAL:HG21	1:C:358:HIS:O	2.07	0.54
1:A:559:TRP:H	1:A:564:GLN:HE21	1.55	0.54
1:A:305:ALA:HB3	1:A:379:VAL:CG2	2.35	0.54
1:D:566:ILE:HD13	1:D:566:ILE:N	2.23	0.53
1:D:299:PHE:HB2	1:D:333:LEU:HD23	1.90	0.53
1:B:406:LEU:O	2:B:601:NAP:H2N	2.09	0.53
1:B:298:LEU:HD23	1:B:376:LEU:CD1	2.39	0.53
1:B:520:ASP:O	1:B:524:GLU:HG2	2.09	0.53

Continued on next page...

For Manuscript Review

Continued from previous page...

Atom-1	Atom-2	Interatomic distance (Å)	Clash overlap (Å)
1:C:151:THR:HG23	1:C:192:ILE:HD12	1.91	0.52
1:C:297:ILE:HD13	1:C:297:ILE:N	2.23	0.52
1:A:228:PHE:CE2	1:A:229:MET:CE	2.93	0.52
1:D:228:PHE:CE2	1:D:229:MET:CE	2.93	0.52
1:C:559:TRP:H	1:C:564:GLN:NE2	2.07	0.51
1:D:9:ARG:HD2	1:D:552:ILE:HG22	1.92	0.51
1:C:361:MET:C	1:C:361:MET:SD	2.94	0.51
1:B:10:HIS:CG	1:D:138:ASN:HB3	2.45	0.51
1:B:151:THR:HG23	1:B:192:ILE:CD1	2.35	0.51
1:A:419:GLN:O	1:A:423:ILE:HD13	2.11	0.51
1:D:339:LEU:H	1:D:339:LEU:CD2	2.06	0.51
1:C:381:ALA:HA	1:C:407:SER:HB3	1.92	0.51
1:A:243:GLN:HG3	1:A:266:PHE:CZ	2.46	0.51
1:C:348:THR:CB	1:C:351:LYS:HB2	2.38	0.51
1:C:281:LEU:HD23	1:C:404:PHE:CE2	2.46	0.51
1:A:98:MET:HE1	1:A:453:ASN:HD21	1.76	0.50
1:C:110:CYS:O	1:C:165:ASN:HB3	2.12	0.50
1:D:62:PHE:CZ	1:D:71:ARG:HD3	2.46	0.50
1:C:18:LEU:C	1:C:18:LEU:HD23	2.36	0.50
1:D:336:SER:OG	2:D:601:NAP:O1X	2.25	0.50
1:D:445:GLN:OE1	1:D:445:GLN:HA	2.10	0.50
1:A:376:LEU:HD13	1:A:386:PHE:CZ	2.47	0.50
1:B:315:MET:HE1	1:B:476:ASN:HD22	1.74	0.50
1:A:239:ASN:HB2	4:A:766:HOH:O	2.12	0.50
1:C:98:MET:HE3	1:C:453:ASN:ND2	2.27	0.50
1:D:263:TYR:O	1:D:265:THR:HG22	2.12	0.50
1:C:51:GLN:HG2	1:C:88:ARG:HG2	1.94	0.50
1:D:337:LYS:HE2	1:D:346:SER:CB	2.42	0.50
1:C:151:THR:CG2	1:C:192:ILE:HD12	2.42	0.49
1:C:157:LEU:HG	1:C:157:LEU:O	2.11	0.49
1:C:361:MET:SD	1:C:362:LYS:N	2.86	0.49
1:B:276:VAL:O	1:B:500:TYR:OH	2.23	0.49
1:B:484:VAL:O	1:B:488:GLN:HG2	2.12	0.49
1:C:300:GLN:HE21	1:C:300:GLN:CA	2.25	0.49
1:D:183:MET:HE1	1:D:463:LEU:HA	1.93	0.49
1:C:502:PRO:O	1:C:505:THR:OG1	2.29	0.49
1:B:122:GLY:CA	1:B:190:PRO:HG2	2.43	0.49
1:C:386:PHE:HB2	1:C:415:CYS:HB3	1.94	0.49
1:B:205:ASP:O	1:B:212:ARG:NH1	2.45	0.49
1:B:122:GLY:HA2	1:B:190:PRO:HG2	1.94	0.48
1:C:250:VAL:HG13	1:C:251:ASN:N	2.27	0.48

Continued on next page...

For Manuscript Review

Continued from previous page...

Atom-1	Atom-2	Interatomic distance (Å)	Clash overlap (Å)
1:D:453:ASN:OD1	1:D:455:SER:OG	2.26	0.48
1:B:341:VAL:CG1	1:B:358:HIS:HB3	2.42	0.48
1:C:277:ALA:CB	1:C:309:ILE:HG12	2.44	0.48
1:B:447:LEU:CD2	1:B:496:GLU:C	2.86	0.48
1:D:303:GLY:HA3	2:D:601:NAP:O5B	2.13	0.48
1:D:440:THR:HA	1:D:446:THR:HA	1.95	0.48
1:A:228:PHE:HD2	1:A:229:MET:HE2	1.75	0.48
1:B:458:PHE:N	1:B:459:PRO:CD	2.76	0.48
1:A:379:VAL:HG23	2:A:601:NAP:O4D	2.13	0.48
1:C:281:LEU:CD2	1:C:404:PHE:CE2	2.97	0.48
1:B:99:PRO:HA	1:B:103:THR:O	2.14	0.48
1:B:341:VAL:HG12	1:B:358:HIS:HB3	1.95	0.48
1:C:98:MET:HE3	1:C:453:ASN:HD21	1.79	0.48
1:A:169:ILE:HB	1:A:170:PRO:HD3	1.96	0.48
1:B:448:TYR:N	1:B:448:TYR:CD1	2.81	0.48
1:A:241:LEU:CD1	1:A:473:ILE:HD11	2.44	0.48
1:C:290:ASN:ND2	1:C:295:GLN:HE22	2.12	0.47
1:D:348:THR:O	1:D:352:GLU:CG	2.62	0.47
1:D:351:LYS:C	1:D:353:LYS:H	2.21	0.47
1:C:332:TRP:CH2	1:C:356:HIS:HB2	2.49	0.47
1:B:322:LEU:N	1:B:322:LEU:CD1	2.72	0.47
1:C:363:ASN:ND2	1:C:363:ASN:C	2.71	0.47
1:D:296:THR:H	1:D:374:THR:HG23	1.79	0.47
1:A:9:ARG:O	1:A:9:ARG:HG3	2.15	0.47
1:A:76:MET:HE3	1:A:101:VAL:HG12	1.96	0.47
1:A:50:SER:HB2	1:A:52:GLU:OE2	2.15	0.47
1:A:110:CYS:O	1:A:165:ASN:HB3	2.15	0.47
1:C:151:THR:HB	1:C:245:GLU:O	2.14	0.47
1:C:150:VAL:HA	1:C:191:VAL:O	2.15	0.47
1:C:361:MET:C	1:C:361:MET:CE	2.88	0.47
1:D:421:TYR:CD2	1:D:439:VAL:HG21	2.47	0.47
1:D:514:ALA:O	1:D:518:VAL:HG23	2.16	0.47
1:C:344:ARG:HE	1:C:344:ARG:HB3	1.44	0.46
1:D:564:GLN:HG2	1:D:564:GLN:O	2.15	0.46
1:A:228:PHE:HD2	1:A:229:MET:CE	2.28	0.46
1:C:300:GLN:CA	1:C:300:GLN:NE2	2.78	0.46
1:C:300:GLN:HE22	2:C:601:NAP:C2A	2.29	0.46
1:C:348:THR:HG23	1:C:350:GLU:N	2.30	0.46
1:C:423:ILE:O	1:C:423:ILE:CG2	2.63	0.46
1:B:322:LEU:HD13	1:B:322:LEU:O	2.15	0.46
1:B:447:LEU:HD21	1:B:496:GLU:C	2.41	0.46

Continued on next page...

For Manuscript Review

Continued from previous page...

Atom-1	Atom-2	Interatomic distance (Å)	Clash overlap (Å)
1:D:361:MET:HB2	1:D:362:LYS:HD3	1.96	0.46
1:C:372:LYS:HG2	1:C:397:PHE:CD1	2.47	0.46
1:D:340:ILE:N	1:D:340:ILE:CD1	2.73	0.46
1:D:337:LYS:CE	1:D:346:SER:OG	2.63	0.46
1:C:347:LEU:HD12	1:C:347:LEU:HA	1.69	0.46
1:D:337:LYS:HD3	1:D:346:SER:CB	2.46	0.46
1:D:353:LYS:HE3	1:D:353:LYS:HB3	1.80	0.46
1:D:403:ILE:O	1:D:430:PHE:HA	2.16	0.46
1:D:429:ILE:CG2	1:D:499:LEU:HD21	2.46	0.46
1:C:167:MET:O	1:C:170:PRO:HD2	2.16	0.46
1:D:340:ILE:O	1:D:355:ALA:HA	2.16	0.45
1:A:379:VAL:HG13	1:A:379:VAL:O	2.16	0.45
1:B:379:VAL:O	1:B:379:VAL:HG23	2.16	0.45
1:D:300:GLN:HE21	1:D:300:GLN:HA	1.82	0.45
1:A:229:MET:HE1	1:A:242:ILE:HG21	1.97	0.45
1:C:302:ALA:HA	1:C:306:ALA:HB3	1.99	0.45
1:A:174:LEU:HD13	1:A:188:CYS:HB3	1.99	0.45
1:D:440:THR:O	1:D:440:THR:OG1	2.35	0.45
1:D:98:MET:HE1	1:D:453:ASN:HD21	1.80	0.45
1:D:480:THR:CG2	1:D:516:LYS:HE3	2.46	0.45
1:B:26:LYS:HB3	1:B:29:ALA:HB3	1.99	0.45
1:C:376:LEU:HD13	1:C:386:PHE:CE1	2.51	0.44
1:B:161:ASP:O	1:B:199:ASN:HB2	2.17	0.44
1:D:228:PHE:HD2	1:D:229:MET:HE3	1.82	0.44
1:D:278:VAL:O	1:D:282:LEU:HG	2.16	0.44
1:B:153:GLY:HA2	1:B:156:ILE:HD11	1.99	0.44
1:D:358:HIS:NE2	1:D:360:GLU:HG2	2.32	0.44
1:D:361:MET:HE3	1:D:362:LYS:HZ3	1.82	0.44
1:C:326:LYS:HD3	1:C:326:LYS:HA	1.53	0.44
1:C:124:PHE:CD2	1:C:192:ILE:CG2	3.01	0.44
1:C:344:ARG:HB2	1:C:347:LEU:HD22	2.00	0.44
1:D:167:MET:HE2	1:D:192:ILE:CG2	2.48	0.44
1:C:124:PHE:CD2	1:C:192:ILE:HG21	2.53	0.44
1:C:26:LYS:HG2	1:C:549:TYR:CD1	2.52	0.44
1:C:300:GLN:HE22	2:C:601:NAP:H2A	1.83	0.44
1:D:347:LEU:HD21	1:D:355:ALA:HB2	1.99	0.44
1:A:98:MET:HE2	1:A:98:MET:HB3	1.82	0.43
1:C:564:GLN:NE2	1:D:32:LEU:HD23	2.33	0.43
1:B:442:PRO:HG2	1:B:443:ASN:ND2	2.32	0.43
1:B:298:LEU:HD23	1:B:376:LEU:HD13	1.99	0.43
1:C:10:HIS:HD2	1:C:553:LEU:O	1.99	0.43

Continued on next page...

For Manuscript Review

Continued from previous page...

Atom-1	Atom-2	Interatomic distance (Å)	Clash overlap (Å)
1:A:305:ALA:HB1	1:A:379:VAL:HG21	1.98	0.43
1:C:363:ASN:C	1:C:363:ASN:HD22	2.26	0.43
1:C:376:LEU:HD23	1:C:376:LEU:HA	1.75	0.43
1:D:351:LYS:C	1:D:353:LYS:N	2.76	0.43
1:A:9:ARG:CG	1:A:552:ILE:HG22	2.49	0.43
1:B:192:ILE:O	1:B:192:ILE:HG23	2.19	0.43
1:A:174:LEU:HD23	1:A:174:LEU:HA	1.91	0.43
1:C:242:ILE:O	1:C:265:THR:HA	2.19	0.43
1:C:287:ILE:HG13	1:C:494:LEU:HD22	2.01	0.43
1:D:339:LEU:HD21	1:D:360:GLU:HB3	2.01	0.43
1:C:249:ASN:ND2	1:C:304:GLU:HG3	2.34	0.42
1:D:339:LEU:N	1:D:339:LEU:CD1	2.73	0.42
1:D:533:PRO:HG2	1:D:536:LYS:HD2	2.00	0.42
1:A:177:TYR:HE1	1:A:462:ALA:HB2	1.83	0.42
1:C:161:ASP:O	1:C:199:ASN:HB2	2.19	0.42
1:D:393:ASP:HB3	1:D:397:PHE:CZ	2.55	0.42
1:A:471:ARG:NH2	4:A:709:HOH:O	2.51	0.42
1:D:228:PHE:CE2	1:D:229:MET:HE2	2.52	0.42
1:A:9:ARG:O	1:A:552:ILE:HA	2.19	0.42
1:A:568:THR:O	1:A:569:LYS:C	2.62	0.42
1:B:192:ILE:HD12	1:B:193:LEU:N	2.35	0.42
1:A:123:LEU:CD2	1:A:137:LEU:HD12	2.49	0.42
1:C:156:ILE:HD13	1:C:246:ASP:HB3	2.01	0.42
1:B:242:ILE:O	1:B:265:THR:HA	2.19	0.42
1:C:99:PRO:HA	1:C:103:THR:O	2.20	0.42
1:C:151:THR:CB	1:C:245:GLU:O	2.67	0.42
1:D:228:PHE:HD2	1:D:229:MET:HE2	1.73	0.42
1:C:169:ILE:HB	1:C:170:PRO:HD3	2.02	0.41
1:C:364:LEU:HA	1:C:367:ILE:CG1	2.50	0.41
1:B:98:MET:N	1:B:99:PRO:HD2	2.35	0.41
1:C:379:VAL:HG23	1:C:379:VAL:O	2.20	0.41
1:D:364:LEU:HD23	1:D:364:LEU:HA	1.91	0.41
1:D:471:ARG:HH11	1:D:471:ARG:CG	2.33	0.41
1:A:208:TYR:CZ	1:A:210:GLY:HA3	2.55	0.41
1:C:280:GLY:O	1:C:283:ALA:HB3	2.21	0.41
1:B:305:ALA:O	1:B:309:ILE:HG13	2.19	0.41
1:D:149:VAL:HG23	1:D:174:LEU:HD21	2.02	0.41
1:D:198:GLU:OE2	1:D:214:ARG:HD3	2.20	0.41
1:D:559:TRP:H	1:D:564:GLN:HE21	1.68	0.41
1:B:368:VAL:HG21	1:B:390:ILE:HG23	2.03	0.41
1:A:312:LEU:HD12	1:A:312:LEU:HA	1.93	0.41

Continued on next page...

For Manuscript Review

Continued from previous page...

Atom-1	Atom-2	Interatomic distance (Å)	Clash overlap (Å)
1:D:337:LYS:HD3	1:D:346:SER:HB2	2.03	0.41
1:C:377:ILE:HA	1:C:404:PHE:HB2	2.02	0.41
1:D:339:LEU:N	1:D:339:LEU:CD2	2.72	0.41
1:D:348:THR:HG23	1:D:351:LYS:HD2	2.02	0.41
1:D:358:HIS:NE2	1:D:370:GLU:HG2	2.36	0.41
1:A:18:LEU:C	1:A:18:LEU:HD23	2.46	0.41
1:B:273:THR:O	1:B:274:ALA:C	2.62	0.41
1:C:290:ASN:ND2	1:C:295:GLN:NE2	2.69	0.41
1:B:391:LEU:HB2	1:B:423:ILE:HG21	2.03	0.41
1:D:161:ASP:O	1:D:199:ASN:HB2	2.21	0.41
1:D:493:HIS:HB3	1:D:498:ARG:HB2	2.02	0.41
1:A:376:LEU:HD13	1:A:386:PHE:CE1	2.56	0.40
1:B:293:SER:CB	1:B:320:GLU:OE2	2.69	0.40
1:D:147:ALA:O	1:D:188:CYS:HA	2.22	0.40
1:D:9:ARG:HH12	1:D:186:GLN:HE22	1.65	0.40
1:D:174:LEU:HD23	1:D:174:LEU:HA	1.96	0.40
1:B:266:PHE:HB2	1:B:271:GLN:OE1	2.22	0.40
1:A:220:GLU:HB2	4:A:701:HOH:O	2.21	0.40
1:B:408:ASN:OD1	1:B:434:SER:N	2.53	0.40
1:D:335:ASP:HB3	1:D:337:LYS:H	1.86	0.40
1:D:350:GLU:OE1	1:D:350:GLU:N	2.55	0.40

There are no symmetry-related clashes.

5.3 Torsion angles (i)

5.3.1 Protein backbone (i)

In the following table, the Percentiles column shows the percent Ramachandran outliers of the chain as a percentile score with respect to all X-ray entries followed by that with respect to entries of similar resolution.

The Analysed column shows the number of residues for which the backbone conformation was analysed, and the total number of residues.

Mol	Chain	Analysed	Favoured	Allowed	Outliers	Percentiles	
1	A	564/572 (99%)	542 (96%)	21 (4%)	1 (0%)	44	54
1	B	560/572 (98%)	538 (96%)	22 (4%)	0	100	100
1	C	560/572 (98%)	525 (94%)	33 (6%)	2 (0%)	30	37
1	D	559/572 (98%)	527 (94%)	28 (5%)	4 (1%)	19	23

Continued on next page...

For Manuscript Review

Continued from previous page...

Mol	Chain	Analysed	Favoured	Allowed	Outliers	Percentiles	
All	All	2243/2288 (98%)	2132 (95%)	104 (5%)	7 (0%)	37	46

All (7) Ramachandran outliers are listed below:

Mol	Chain	Res	Type
1	C	362	LYS
1	A	399	GLU
1	D	444	GLY
1	C	157	LEU
1	D	346	SER
1	D	379	VAL
1	D	337	LYS

5.3.2 Protein sidechains (i)

In the following table, the Percentiles column shows the percent sidechain outliers of the chain as a percentile score with respect to all X-ray entries followed by that with respect to entries of similar resolution.

The Analysed column shows the number of residues for which the sidechain conformation was analysed, and the total number of residues.

Mol	Chain	Analysed	Rotameric	Outliers	Percentiles	
1	A	484/490 (99%)	448 (93%)	36 (7%)	11	14
1	B	481/490 (98%)	437 (91%)	44 (9%)	7	9
1	C	481/490 (98%)	439 (91%)	42 (9%)	8	10
1	D	480/490 (98%)	423 (88%)	57 (12%)	4	4
All	All	1926/1960 (98%)	1747 (91%)	179 (9%)	7	8

All (179) residues with a non-rotameric sidechain are listed below:

Mol	Chain	Res	Type
1	A	7	ARG
1	A	37	GLN
1	A	98	MET
1	A	108	LEU
1	A	137	LEU
1	A	157	LEU
1	A	164	CYS
1	A	176	LEU

Continued on next page...

For Manuscript Review

Continued from previous page...

Mol	Chain	Res	Type
1	A	192	ILE
1	A	220	GLU
1	A	232	VAL
1	A	250	VAL
1	A	281	LEU
1	A	290	ASN
1	A	300	GLN
1	A	304	GLU
1	A	315	MET
1	A	319	LYS
1	A	324	LYS
1	A	337	LYS
1	A	349	GLN
1	A	359	GLU
1	A	365	GLU
1	A	371	ILE
1	A	376	LEU
1	A	392	LYS
1	A	410	THR
1	A	419	GLN
1	A	427	ARG
1	A	443	ASN
1	A	470	LEU
1	A	506	ILE
1	A	519	LYS
1	A	537	GLU
1	A	565	LYS
1	A	568	THR
1	C	118	ARG
1	C	137	LEU
1	C	176	LEU
1	C	183	MET
1	C	192	ILE
1	C	243	GLN
1	C	281	LEU
1	C	293	SER
1	C	297	ILE
1	C	300	GLN
1	C	322	LEU
1	C	324	LYS
1	C	325	GLU
1	C	326	LYS

Continued on next page...

For Manuscript Review

Continued from previous page...

Mol	Chain	Res	Type
1	C	330	LYS
1	C	337	LYS
1	C	341	VAL
1	C	342	LYS
1	C	344	ARG
1	C	346	SER
1	C	347	LEU
1	C	348	THR
1	C	351	LYS
1	C	357	GLU
1	C	358	HIS
1	C	359	GLU
1	C	361	MET
1	C	363	ASN
1	C	364	LEU
1	C	369	GLN
1	C	372	LYS
1	C	376	LEU
1	C	402	ILE
1	C	410	THR
1	C	419	GLN
1	C	441	LEU
1	C	445	GLN
1	C	446	THR
1	C	451	GLN
1	C	476	ASN
1	C	487	GLN
1	C	489	VAL
1	B	9	ARG
1	B	13	GLN
1	B	98	MET
1	B	137	LEU
1	B	155	ARG
1	B	173	LYS
1	B	176	LEU
1	B	183	MET
1	B	192	ILE
1	B	193	LEU
1	B	200	GLU
1	B	204	LYS
1	B	220	GLU
1	B	232	VAL

Continued on next page...

For Manuscript Review

Continued from previous page...

Mol	Chain	Res	Type
1	B	243	GLN
1	B	290	ASN
1	B	291	LYS
1	B	293	SER
1	B	297	ILE
1	B	300	GLN
1	B	315	MET
1	B	322	LEU
1	B	340	ILE
1	B	346	SER
1	B	348	THR
1	B	352	GLU
1	B	357	GLU
1	B	362	LYS
1	B	374	THR
1	B	389	GLN
1	B	390	ILE
1	B	397	PHE
1	B	440	THR
1	B	443	ASN
1	B	448	TYR
1	B	451	GLN
1	B	470	LEU
1	B	476	ASN
1	B	506	ILE
1	B	512	LYS
1	B	523	GLN
1	B	525	LYS
1	B	534	GLN
1	B	565	LYS
1	D	8	ARG
1	D	81	ARG
1	D	83	GLU
1	D	98	MET
1	D	119	LYS
1	D	135	SER
1	D	137	LEU
1	D	143	ASP
1	D	176	LEU
1	D	192	ILE
1	D	220	GLU
1	D	230	GLU

Continued on next page...

For Manuscript Review

Continued from previous page...

Mol	Chain	Res	Type
1	D	232	VAL
1	D	253	PHE
1	D	281	LEU
1	D	289	LYS
1	D	300	GLN
1	D	304	GLU
1	D	313	ILE
1	D	315	MET
1	D	322	LEU
1	D	339	LEU
1	D	340	ILE
1	D	341	VAL
1	D	342	LYS
1	D	344	ARG
1	D	347	LEU
1	D	349	GLN
1	D	352	GLU
1	D	353	LYS
1	D	357	GLU
1	D	359	GLU
1	D	360	GLU
1	D	362	LYS
1	D	364	LEU
1	D	374	THR
1	D	387	SER
1	D	392	LYS
1	D	398	ASN
1	D	410	THR
1	D	445	GLN
1	D	451	GLN
1	D	471	ARG
1	D	472	GLN
1	D	477	ILE
1	D	492	LYS
1	D	507	ARG
1	D	508	ASP
1	D	512	LYS
1	D	523	GLN
1	D	532	GLU
1	D	534	GLN
1	D	537	GLU
1	D	541	ARG

Continued on next page...

For Manuscript Review

Continued from previous page...

Mol	Chain	Res	Type
1	D	565	LYS
1	D	566	ILE
1	D	568	THR

Sometimes sidechains can be flipped to improve hydrogen bonding and reduce clashes. All (44) such sidechains are listed below:

Mol	Chain	Res	Type
1	A	10	HIS
1	A	39	ASN
1	A	111	GLN
1	A	186	GLN
1	A	290	ASN
1	A	300	GLN
1	A	389	GLN
1	A	476	ASN
1	A	564	GLN
1	C	12	HIS
1	C	37	GLN
1	C	39	ASN
1	C	61	ASN
1	C	66	ASN
1	C	186	GLN
1	C	262	GLN
1	C	295	GLN
1	C	300	GLN
1	C	363	ASN
1	C	398	ASN
1	C	454	ASN
1	C	564	GLN
1	B	12	HIS
1	B	13	GLN
1	B	23	HIS
1	B	37	GLN
1	B	39	ASN
1	B	356	HIS
1	B	389	GLN
1	B	443	ASN
1	B	476	ASN
1	B	487	GLN
1	B	523	GLN
1	D	39	ASN
1	D	66	ASN

Continued on next page...

For Manuscript Review

Continued from previous page...

Mol	Chain	Res	Type
1	D	111	GLN
1	D	112	GLN
1	D	186	GLN
1	D	213	GLN
1	D	239	ASN
1	D	295	GLN
1	D	300	GLN
1	D	487	GLN
1	D	564	GLN

5.3.3 RNA (i)

There are no RNA molecules in this entry.

5.4 Non-standard residues in protein, DNA, RNA chains (i)

There are no non-standard protein/DNA/RNA residues in this entry.

5.5 Carbohydrates (i)

There are no oligosaccharides in this entry.

5.6 Ligand geometry (i)

Of 8 ligands modelled in this entry, 4 are monoatomic - leaving 4 for Mogul analysis.

In the following table, the Counts columns list the number of bonds (or angles) for which Mogul statistics could be retrieved, the number of bonds (or angles) that are observed in the model and the number of bonds (or angles) that are defined in the Chemical Component Dictionary. The Link column lists molecule types, if any, to which the group is linked. The Z score for a bond length (or angle) is the number of standard deviations the observed value is removed from the expected value. A bond length (or angle) with $|Z| > 2$ is considered an outlier worth inspection. RMSZ is the root-mean-square of all Z scores of the bond lengths (or angles).

Mol	Type	Chain	Res	Link	Bond lengths			Bond angles		
					Counts	RMSZ	$\#\|Z\| > 2$	Counts	RMSZ	$\#\|Z\| > 2$
2	NAP	C	601	-	45,52,52	0.71	0	56,80,80	0.81	2 (3%)
2	NAP	D	601	-	45,52,52	0.78	0	56,80,80	0.89	3 (5%)
2	NAP	A	601	-	45,52,52	0.86	1 (2%)	56,80,80	0.77	3 (5%)
2	NAP	B	601	-	45,52,52	0.75	0	56,80,80	0.87	3 (5%)

For Manuscript Review

In the following table, the Chirals column lists the number of chiral outliers, the number of chiral centers analysed, the number of these observed in the model and the number defined in the Chemical Component Dictionary. Similar counts are reported in the Torsion and Rings columns. '-' means no outliers of that kind were identified.

Mol	Type	Chain	Res	Link	Chirals	Torsions	Rings
2	NAP	C	601	-	-	18/31/67/67	0/5/5/5
2	NAP	D	601	-	-	8/31/67/67	0/5/5/5
2	NAP	A	601	-	-	8/31/67/67	0/5/5/5
2	NAP	B	601	-	-	5/31/67/67	0/5/5/5

All (1) bond length outliers are listed below:

Mol	Chain	Res	Type	Atoms	Z	Observed(Å)	Ideal(Å)
2	A	601	NAP	P2B-O3X	-2.13	1.46	1.54

All (11) bond angle outliers are listed below:

Mol	Chain	Res	Type	Atoms	Z	Observed(^{o})	Ideal(^{o})
2	D	601	NAP	O4D-C1D-C2D	-2.64	103.07	106.93
2	C	601	NAP	C3N-C2N-N1N	-2.57	117.92	120.43
2	B	601	NAP	O4D-C1D-C2D	-2.47	103.31	106.93
2	B	601	NAP	C6N-N1N-C2N	-2.38	119.80	121.97
2	C	601	NAP	C5A-C6A-N6A	2.33	123.89	120.35
2	B	601	NAP	C5A-C6A-N6A	2.33	123.89	120.35
2	A	601	NAP	C5A-C6A-N6A	2.32	123.89	120.35
2	A	601	NAP	C3B-C2B-C1B	-2.32	98.52	102.89
2	D	601	NAP	C5A-C6A-N6A	2.30	123.85	120.35
2	D	601	NAP	C6N-N1N-C2N	-2.15	120.01	121.97
2	A	601	NAP	C6N-N1N-C2N	-2.06	120.10	121.97

There are no chirality outliers.

All (39) torsion outliers are listed below:

Mol	Chain	Res	Type	Atoms
2	A	601	NAP	C5B-O5B-PA-O1A
2	A	601	NAP	C2B-O2B-P2B-O3X
2	A	601	NAP	O4D-C1D-N1N-C6N
2	C	601	NAP	C5B-O5B-PA-O1A
2	C	601	NAP	C5B-O5B-PA-O2A
2	C	601	NAP	PN-O3-PA-O5B
2	C	601	NAP	C5D-O5D-PN-O3
2	C	601	NAP	O4D-C1D-N1N-C2N

Continued on next page...

For Manuscript Review

Continued from previous page...

Mol	Chain	Res	Type	Atoms
2	C	601	NAP	O4D-C1D-N1N-C6N
2	C	601	NAP	C2D-C1D-N1N-C2N
2	C	601	NAP	C2D-C1D-N1N-C6N
2	B	601	NAP	C2B-O2B-P2B-O1X
2	D	601	NAP	C2B-O2B-P2B-O1X
2	D	601	NAP	C5D-O5D-PN-O3
2	D	601	NAP	O4D-C1D-N1N-C6N
2	A	601	NAP	O4B-C4B-C5B-O5B
2	C	601	NAP	O4B-C4B-C5B-O5B
2	C	601	NAP	O4D-C4D-C5D-O5D
2	C	601	NAP	C3B-C4B-C5B-O5B
2	C	601	NAP	C1B-C2B-O2B-P2B
2	C	601	NAP	C3B-C2B-O2B-P2B
2	A	601	NAP	C1B-C2B-O2B-P2B
2	A	601	NAP	C3B-C4B-C5B-O5B
2	C	601	NAP	C3D-C4D-C5D-O5D
2	D	601	NAP	O4B-C4B-C5B-O5B
2	C	601	NAP	C2B-O2B-P2B-O3X
2	B	601	NAP	C5D-O5D-PN-O3
2	C	601	NAP	PA-O3-PN-O1N
2	C	601	NAP	C5D-O5D-PN-O2N
2	D	601	NAP	C5D-O5D-PN-O1N
2	A	601	NAP	C3B-C2B-O2B-P2B
2	B	601	NAP	O4B-C4B-C5B-O5B
2	D	601	NAP	C1B-C2B-O2B-P2B
2	D	601	NAP	C3B-C4B-C5B-O5B
2	A	601	NAP	C5B-O5B-PA-O3
2	C	601	NAP	C5B-O5B-PA-O3
2	B	601	NAP	C2B-O2B-P2B-O2X
2	B	601	NAP	C5D-O5D-PN-O1N
2	D	601	NAP	C5D-O5D-PN-O2N

There are no ring outliers.

4 monomers are involved in 16 short contacts:

Mol	Chain	Res	Type	Clashes	Symm-Clashes
2	C	601	NAP	3	0
2	D	601	NAP	5	0
2	A	601	NAP	6	0
2	B	601	NAP	2	0

The following is a two-dimensional graphical depiction of Mogul quality analysis of bond lengths,

For Manuscript Review

bond angles, torsion angles, and ring geometry for all instances of the Ligand of Interest. In addition, ligands with molecular weight > 250 and outliers as shown on the validation Tables will also be included. For torsion angles, if less then 5% of the Mogul distribution of torsion angles is within 10 degrees of the torsion angle in question, then that torsion angle is considered an outlier. Any bond that is central to one or more torsion angles identified as an outlier by Mogul will be highlighted in the graph. For rings, the root-mean-square deviation (RMSD) between the ring in question and similar rings identified by Mogul is calculated over all ring torsion angles. If the average RMSD is greater than 60 degrees and the minimal RMSD between the ring in question and any Mogul-identified rings is also greater than 60 degrees, then that ring is considered an outlier. The outliers are highlighted in purple. The color gray indicates Mogul did not find sufficient equivalents in the CSD to analyse the geometry.

Ligand NAP C 601

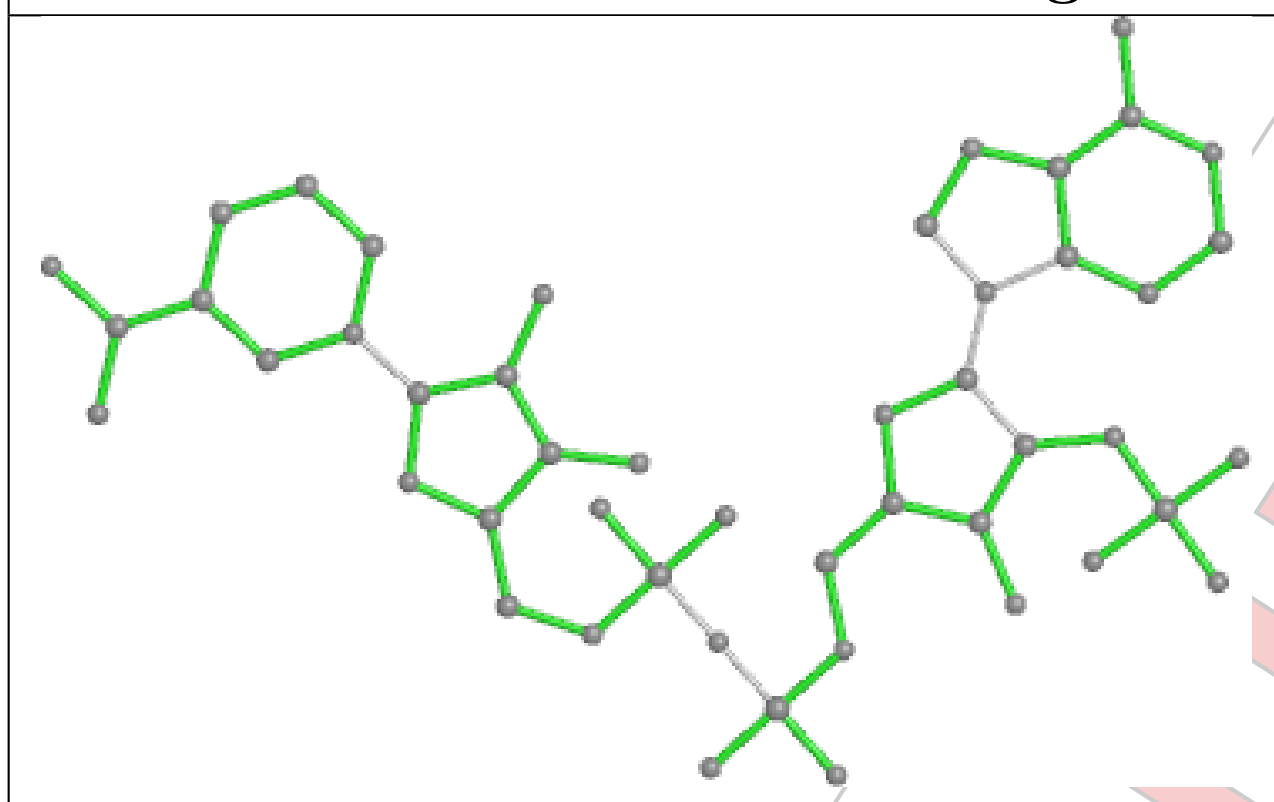

Bond lengths

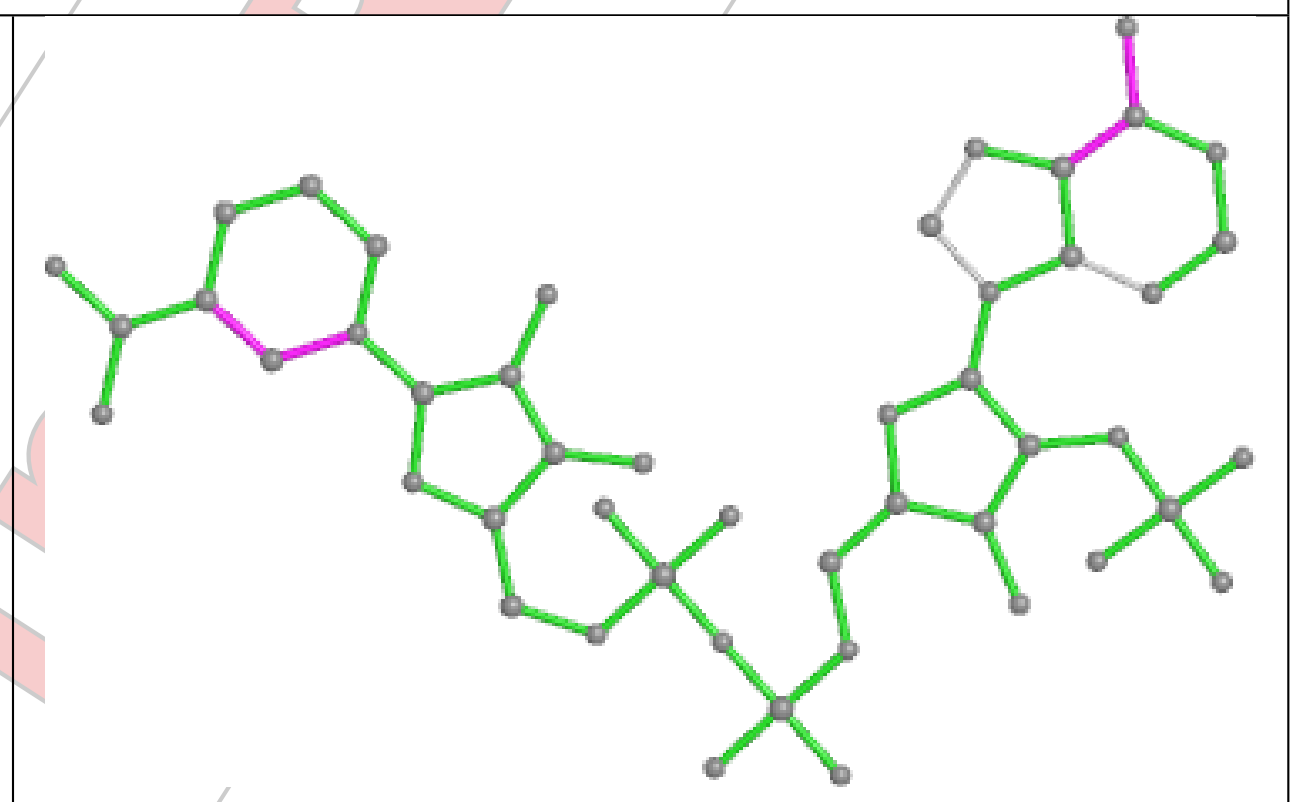

Bond angles

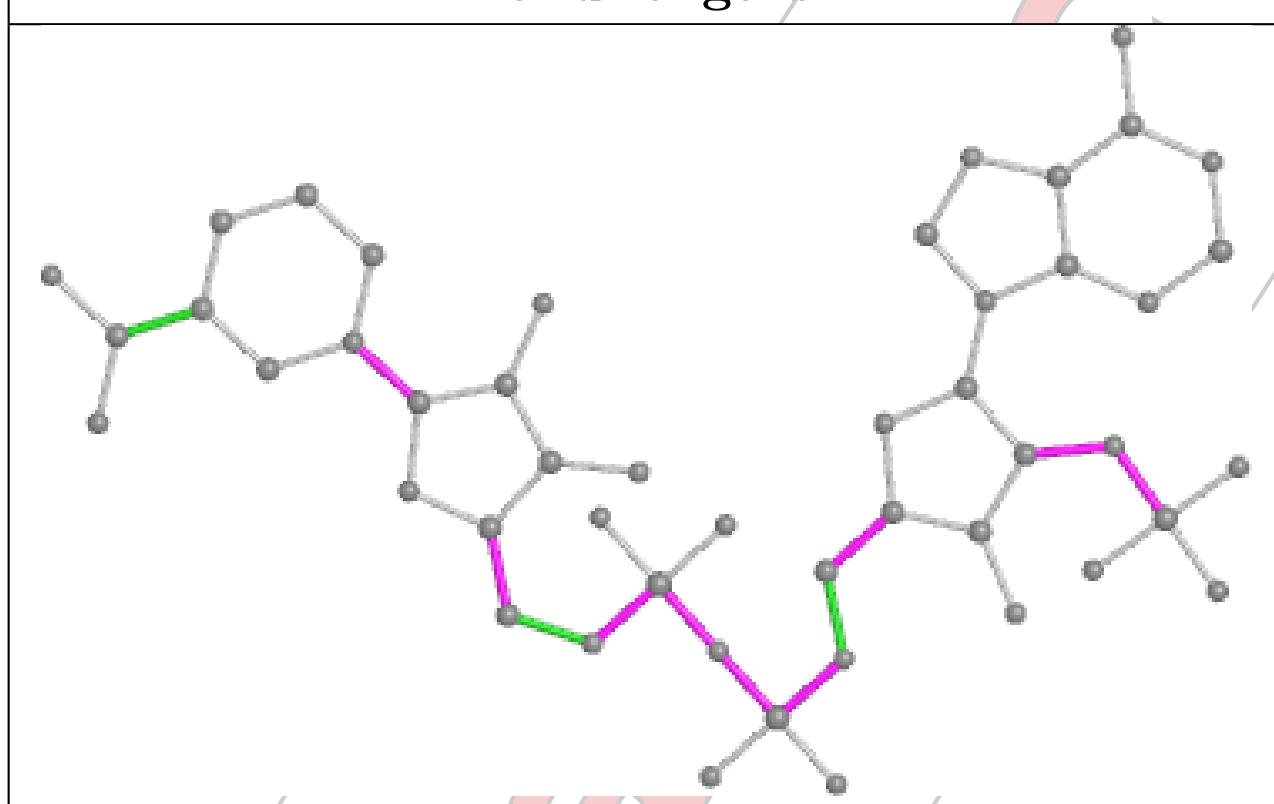

Torsions

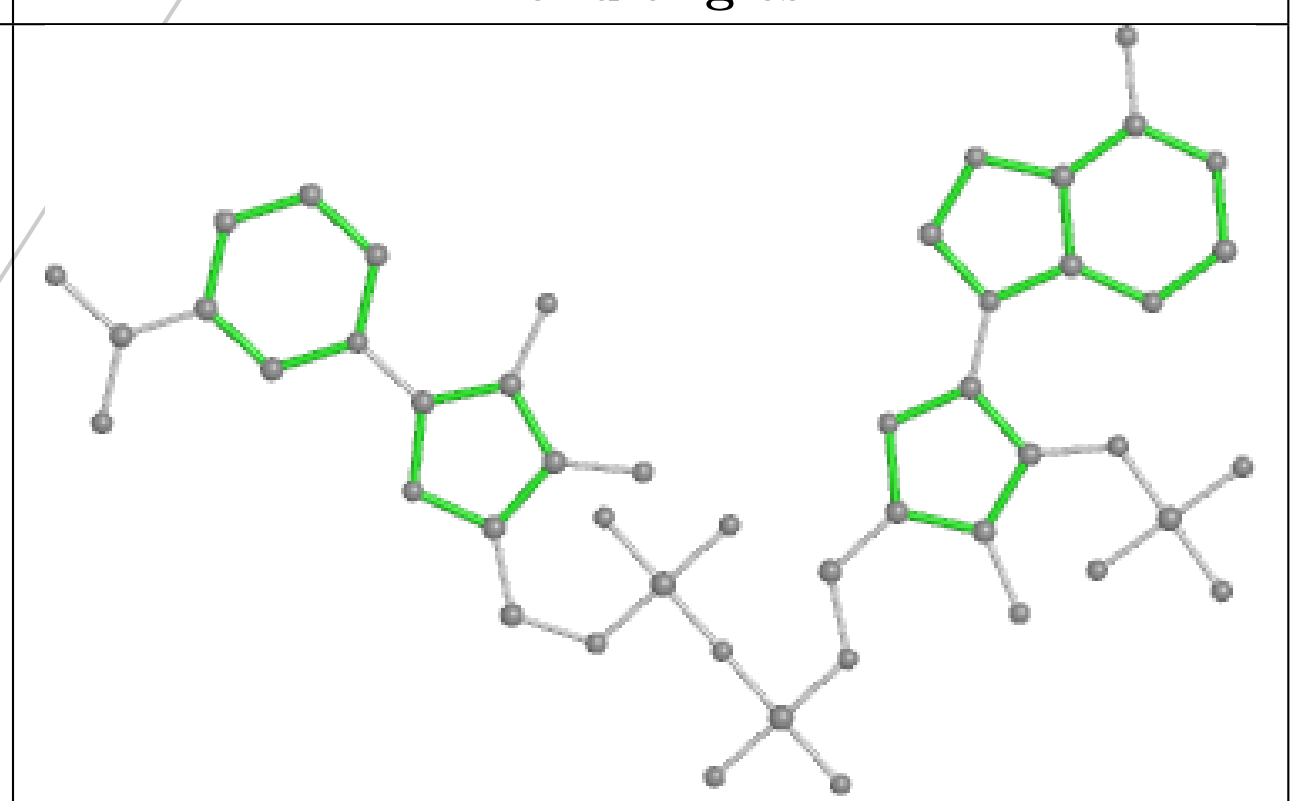

Rings

For Manuscript Review

Ligand NAP D 601

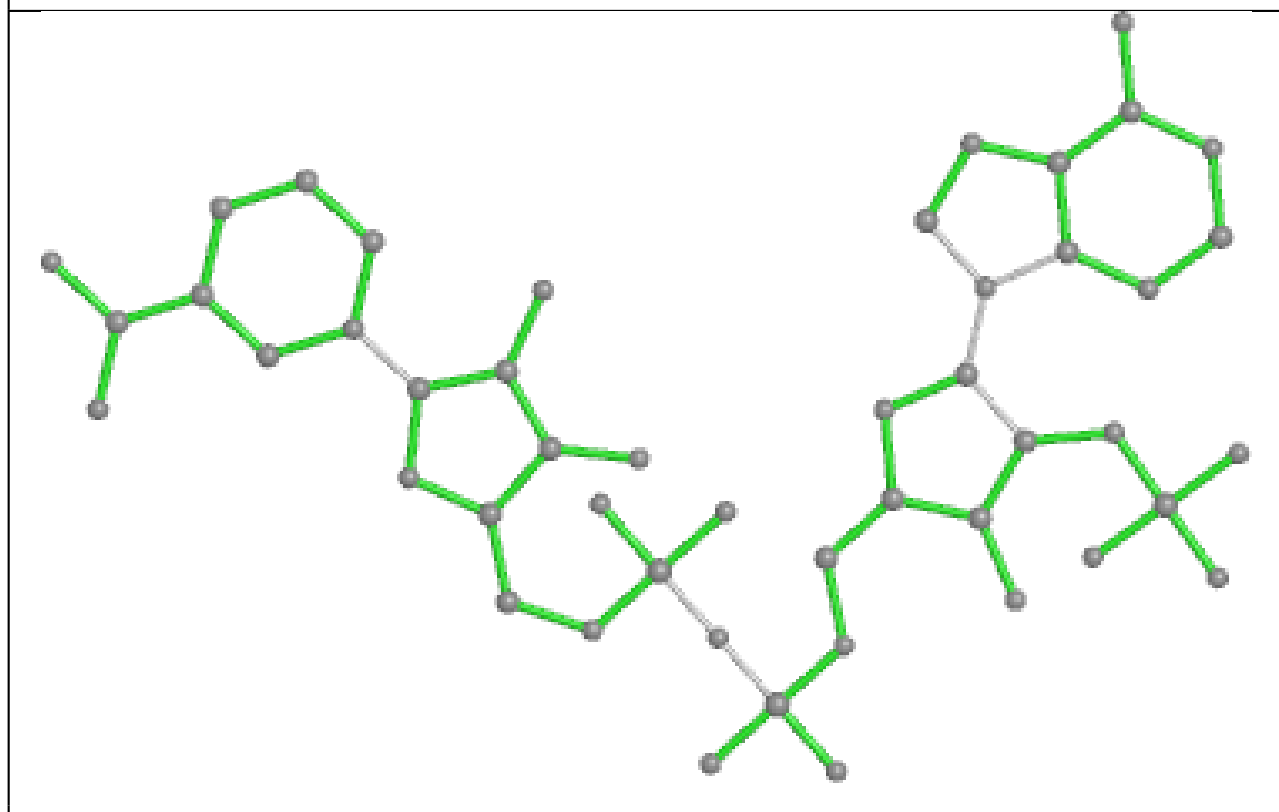

Bond lengths

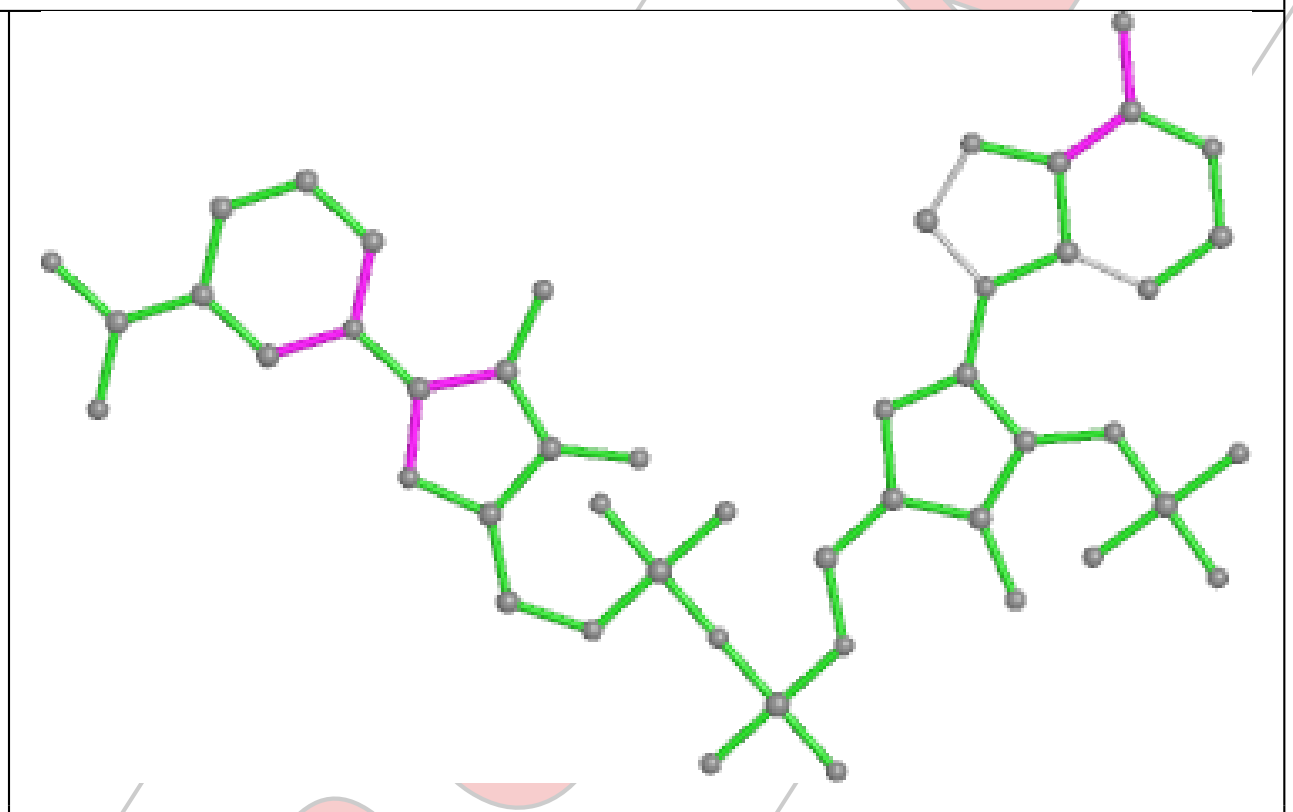

Bond angles

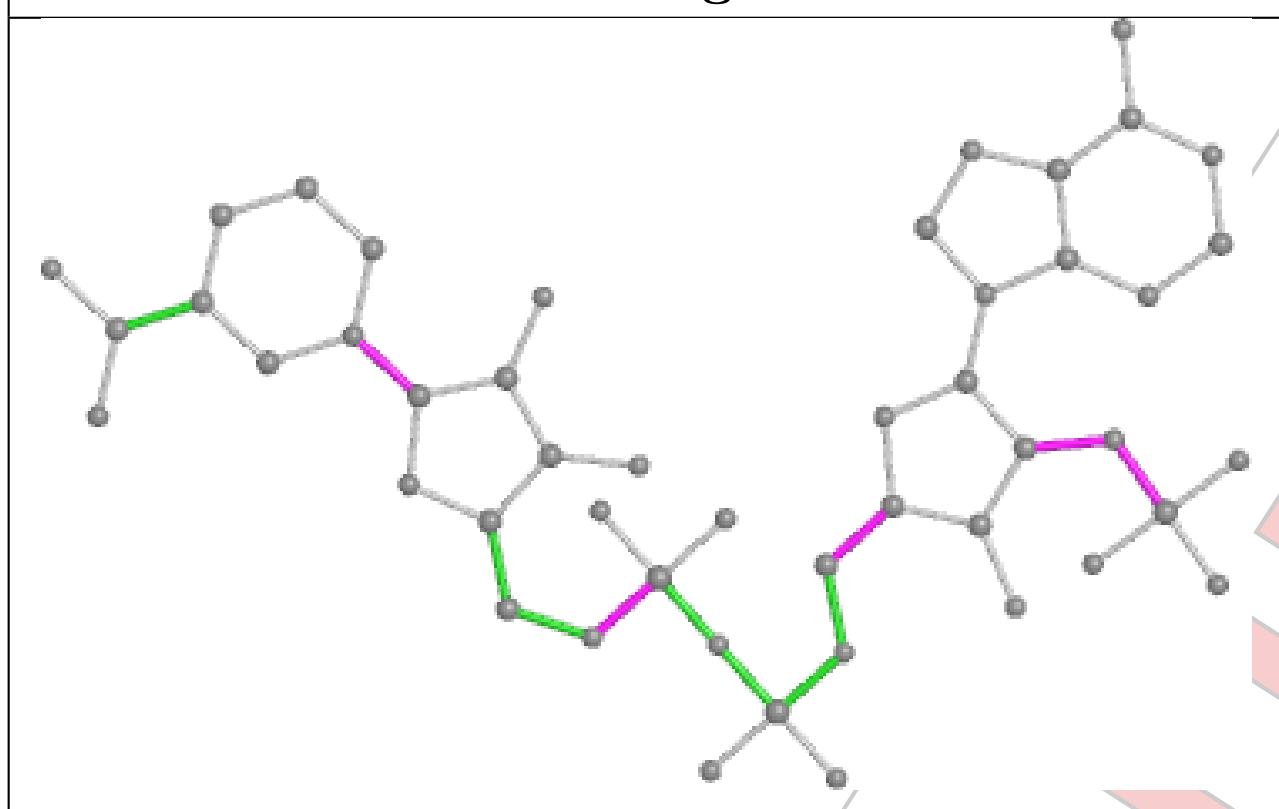

Torsions

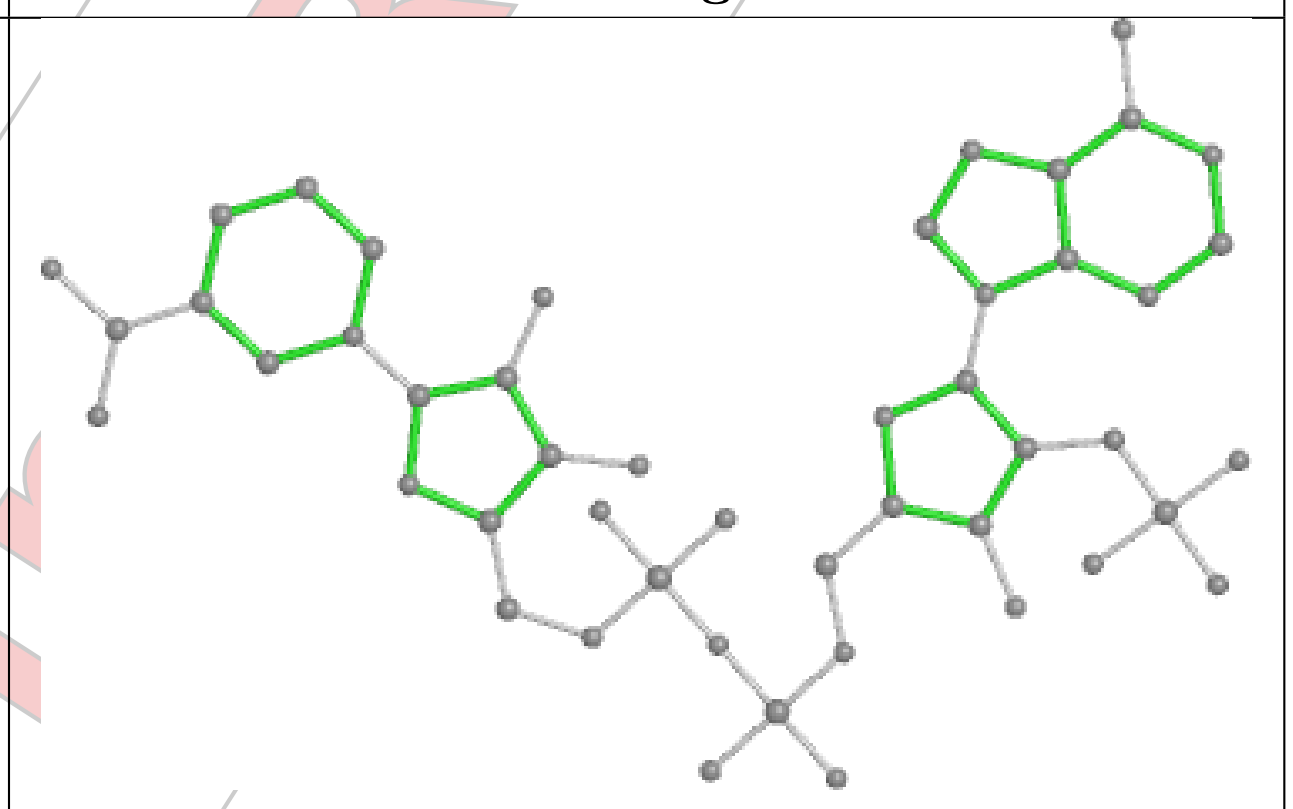

Rings

For Manuscript Review

Ligand NAP A 601

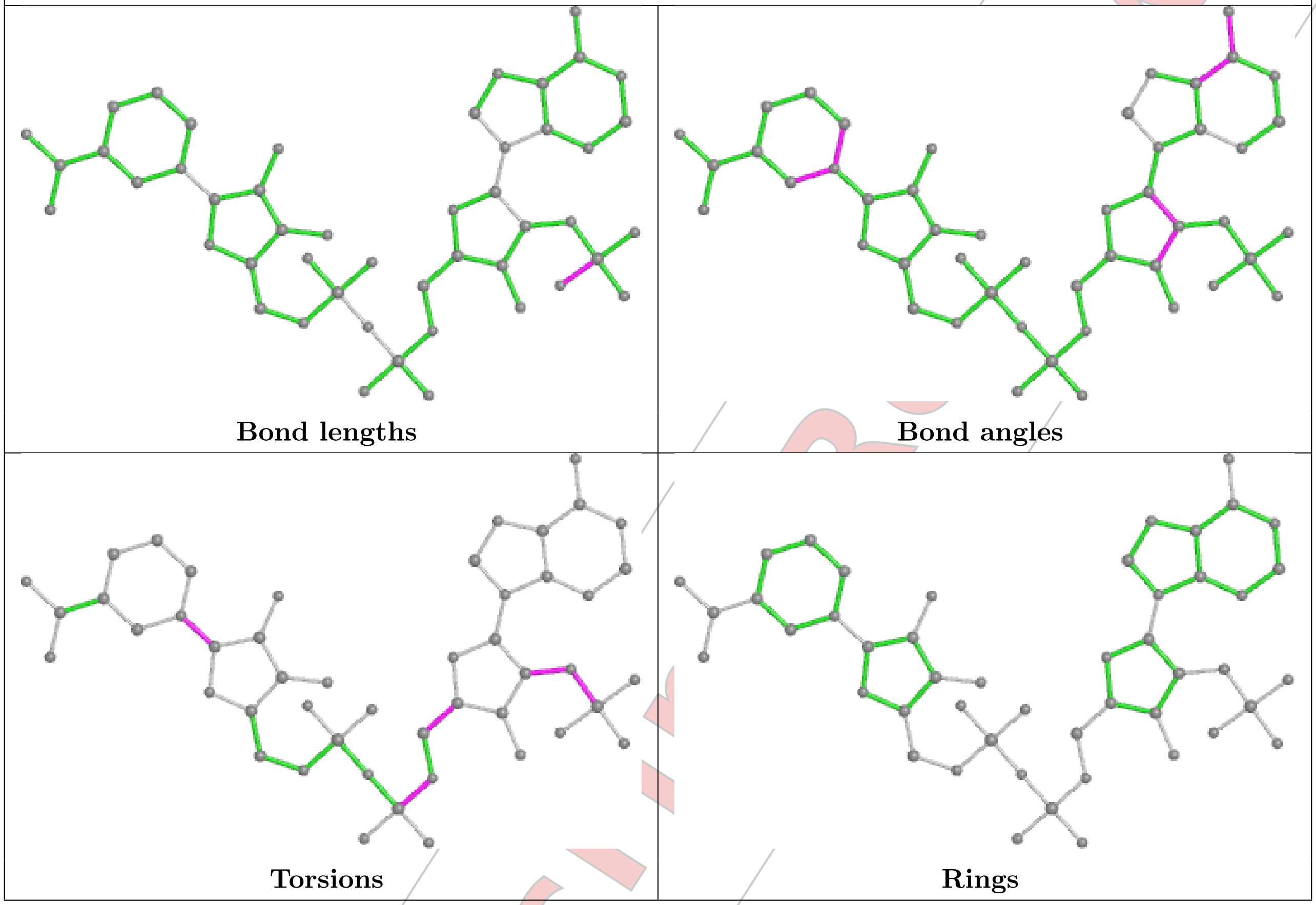

For Manuscript Review

Ligand NAP B 601

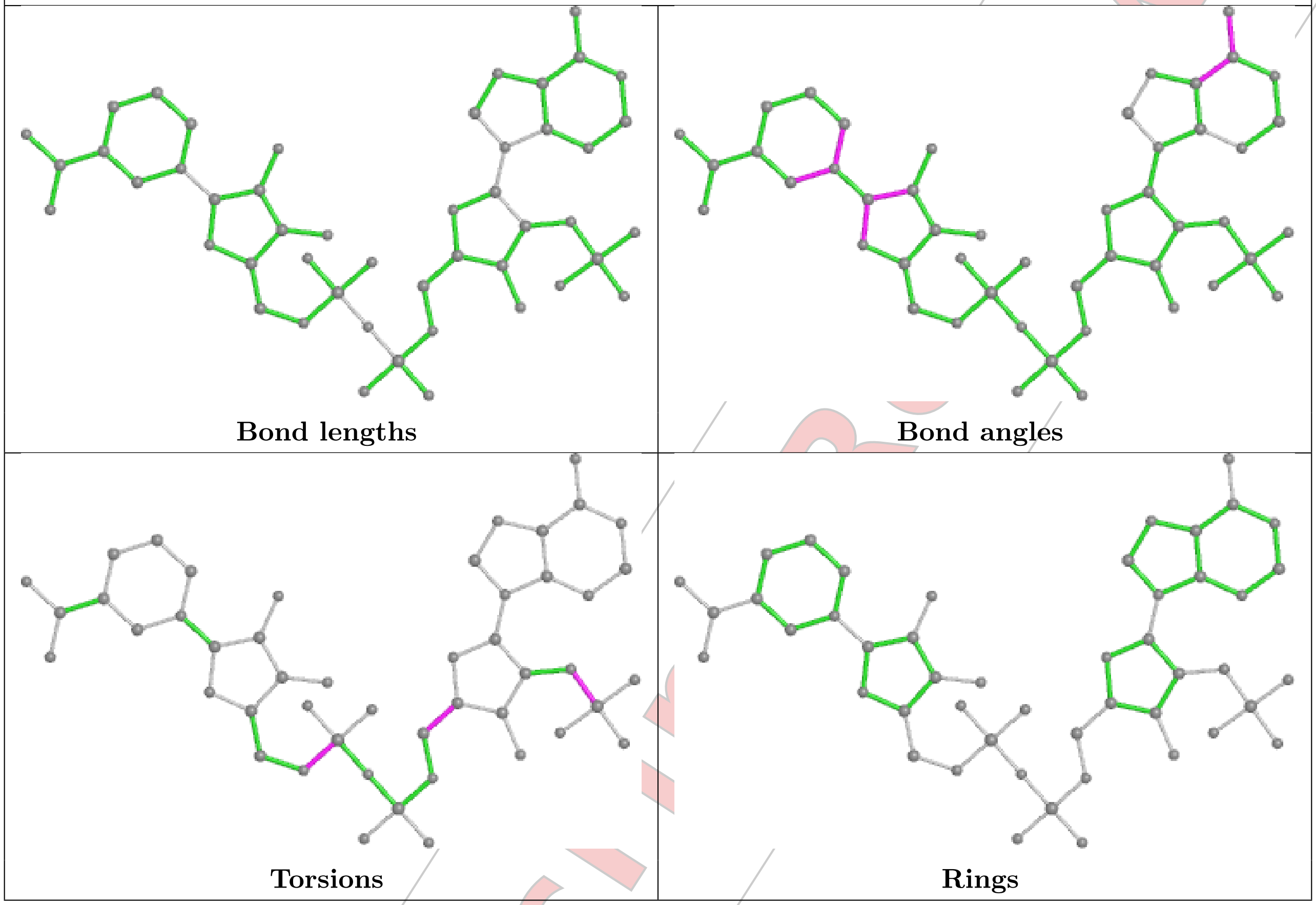

5.7 Other polymers (i)

There are no such residues in this entry.

5.8 Polymer linkage issues (i)

There are no chain breaks in this entry.

For Manuscript Review

6 Fit of model and data (i)

6.1 Protein, DNA and RNA chains (i)

In the following table, the column labelled '#RSRZ> 2' contains the number (and percentage) of RSRZ outliers, followed by percent RSRZ outliers for the chain as percentile scores relative to all X-ray entries and entries of similar resolution. The OWAB column contains the minimum, median, 95th percentile and maximum values of the occupancy-weighted average B-factor per residue. The column labelled 'Q< 0.9' lists the number of (and percentage) of residues with an average occupancy less than 0.9.

Mol	Chain	Analysed	<RSRZ>	#RSRZ>2			OWAB(Å^2)	Q<0.9
1	A	566/572 (98%)	-0.39	0	100	100	47, 67, 98, 123	0
1	B	562/572 (98%)	-0.18	0	100	100	46, 80, 118, 139	0
1	C	562/572 (98%)	-0.12	5 (0%)	81	82	50, 78, 126, 163	0
1	D	561/572 (98%)	-0.15	3 (0%)	87	88	48, 79, 125, 160	0
All	All	2251/2288 (98%)	-0.21	8 (0%)	89	89	46, 75, 121, 163	0

All (8) RSRZ outliers are listed below:

Mol	Chain	Res	Type	RSRZ
1	D	348	THR	3.8
1	C	343	GLY	2.4
1	C	444	GLY	2.3
1	D	383	GLY	2.2
1	C	341	VAL	2.1
1	D	382	ILE	2.1
1	C	442	PRO	2.1
1	C	439	VAL	2.0

6.2 Non-standard residues in protein, DNA, RNA chains (i)

There are no non-standard protein/DNA/RNA residues in this entry.

6.3 Carbohydrates (i)

There are no oligosaccharides in this entry.

For Manuscript Review

6.4 Ligands (i)

In the following table, the Atoms column lists the number of modelled atoms in the group and the number defined in the chemical component dictionary. The B-factors column lists the minimum, median, 95th percentile and maximum values of B factors of atoms in the group. The column labelled 'Q< 0.9' lists the number of atoms with occupancy less than 0.9.

Mol	Type	Chain	Res	Atoms	RSCC	RSR	B-factors(Å^2)	Q<0.9
2	NAP	C	601	48/48	0.93	0.08	78,94,121,122	0
2	NAP	B	601	48/48	0.94	0.07	60,88,104,109	0
2	NAP	D	601	48/48	0.95	0.07	67,82,93,117	0
2	NAP	A	601	48/48	0.97	0.06	55,67,74,79	0
3	MN	D	602	1/1	0.99	0.02	76,76,76,76	0
3	MN	C	602	1/1	1.00	0.01	74,74,74,74	0
3	MN	B	602	1/1	1.00	0.03	81,81,81,81	0
3	MN	A	602	1/1	1.00	0.03	65,65,65,65	0

The following is a graphical depiction of the model fit to experimental electron density of all instances of the Ligand of Interest. In addition, ligands with molecular weight > 250 and outliers as shown on the geometry validation Tables will also be included. Each fit is shown from different orientation to approximate a three-dimensional view.

Electron density around NAP C 601:

2mF$_o$-DF$_c$ (at 0.7 rmsd) in gray
mF$_o$-DF$_c$ (at 3 rmsd) in purple (negative)
and green (positive)

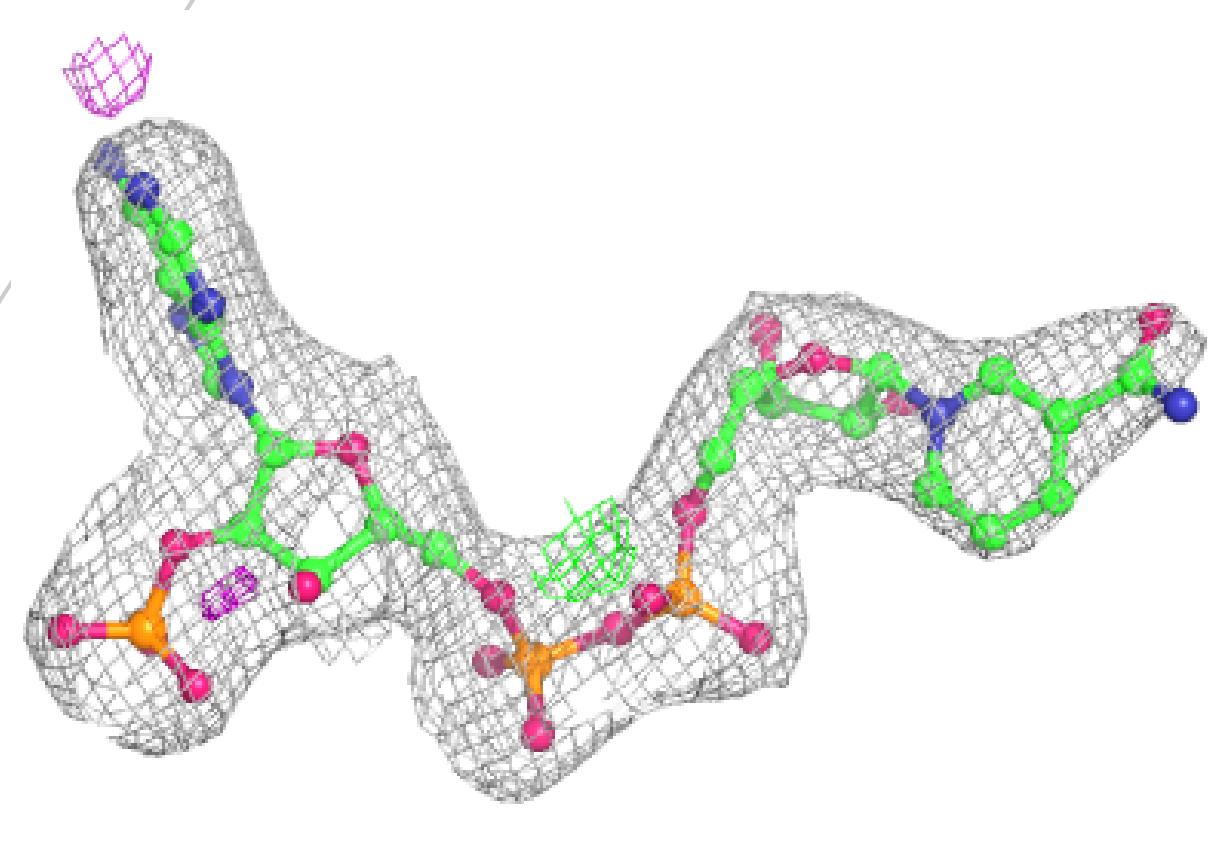

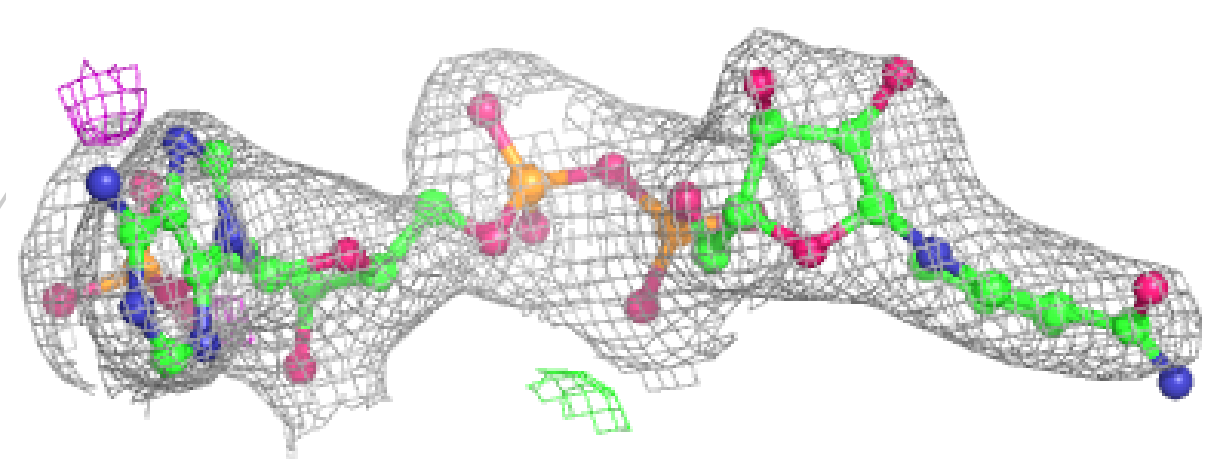

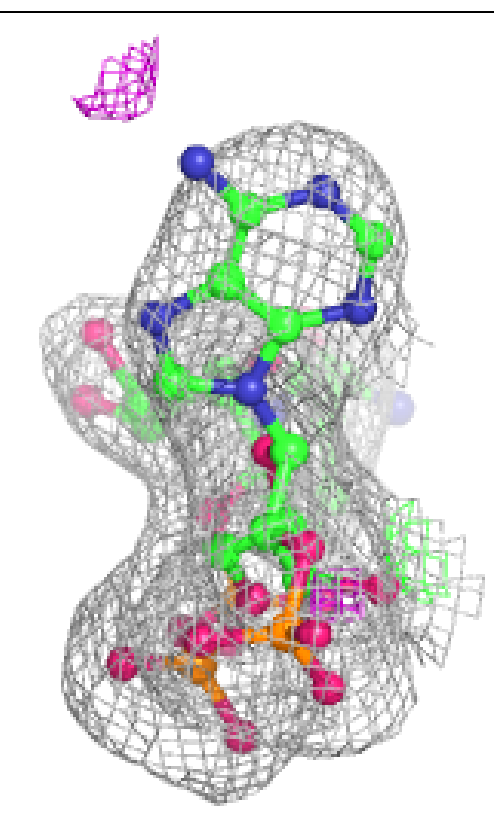

For Manuscript Review

Electron density around NAP B 601:

$2mF_o$-DF_c (at 0.7 rmsd) in gray
mF_o-DF_c (at 3 rmsd) in purple (negative) and green (positive)

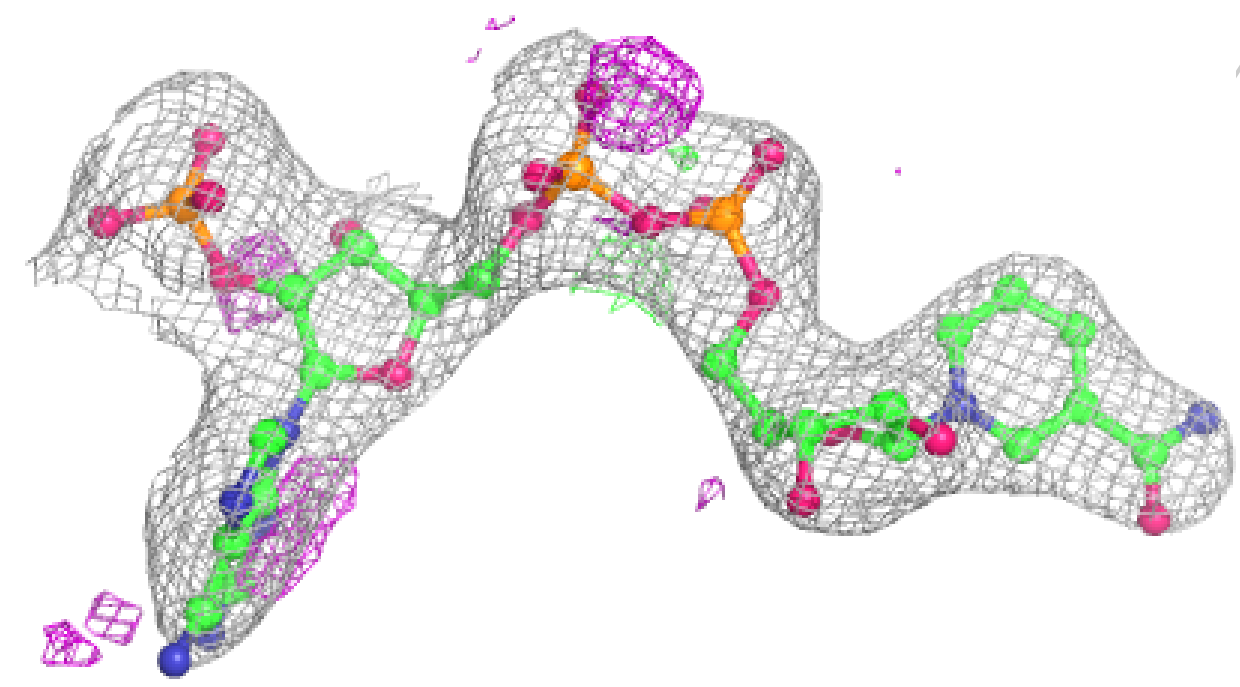

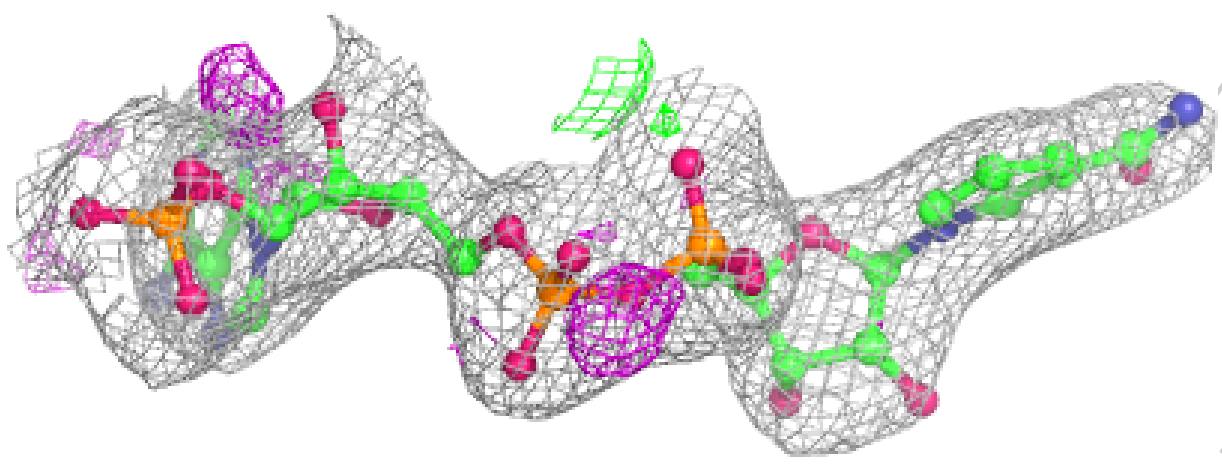

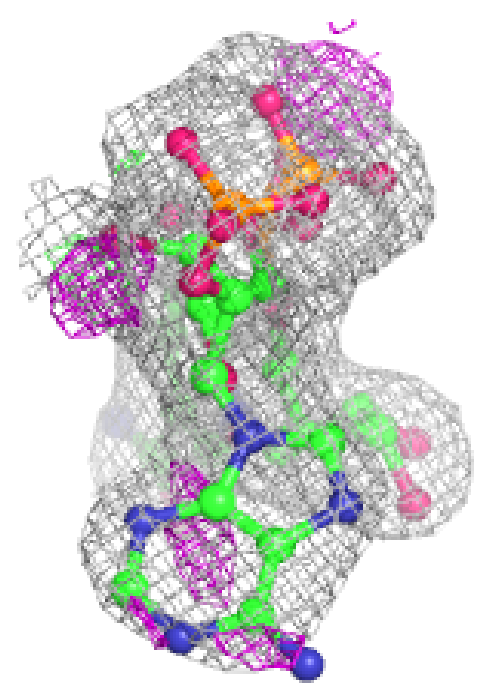

Electron density around NAP D 601:

$2mF_o$-DF_c (at 0.7 rmsd) in gray
mF_o-DF_c (at 3 rmsd) in purple (negative) and green (positive)

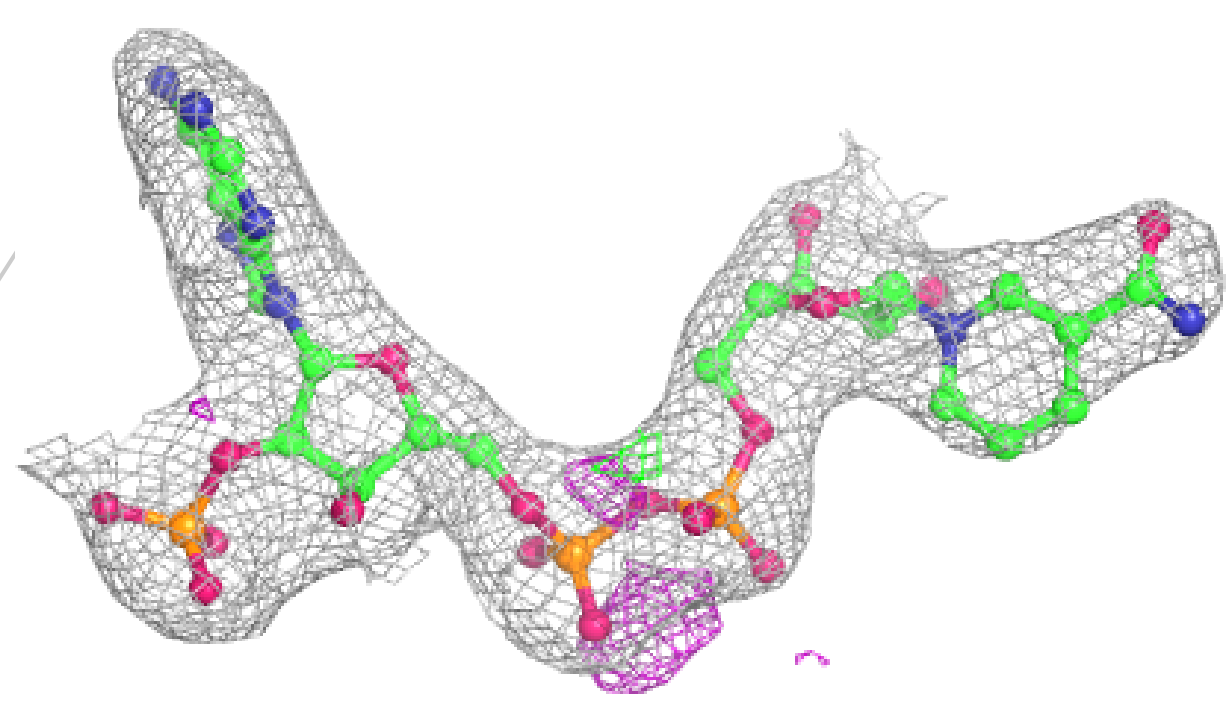

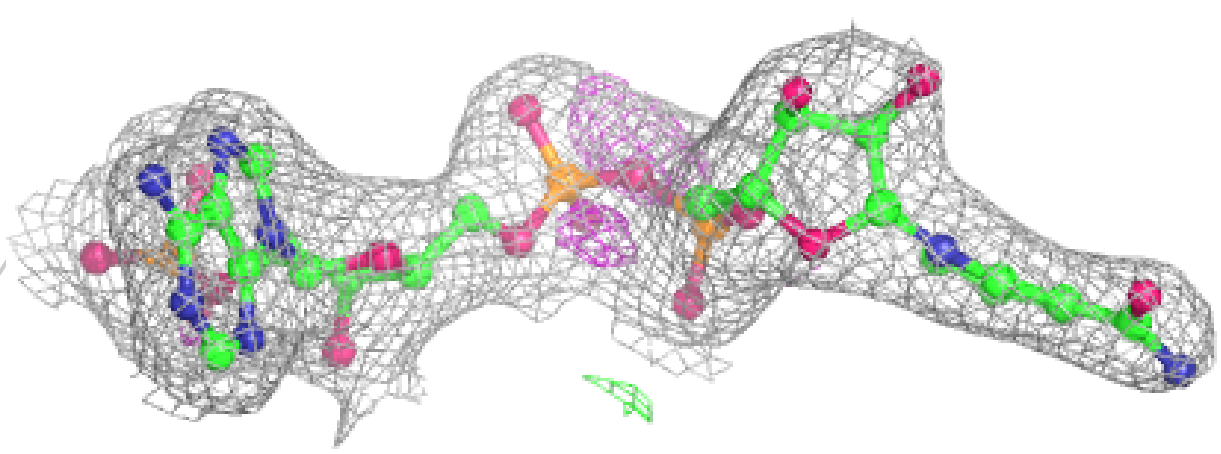

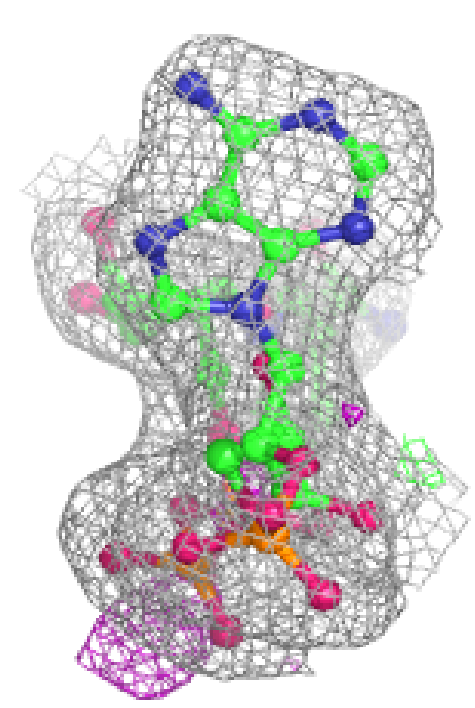

For Manuscript Review

Electron density around NAP A 601:

$2mF_o$-DF_c (at 0.7 rmsd) in gray
mF_o-DF_c (at 3 rmsd) in purple (negative) and green (positive)

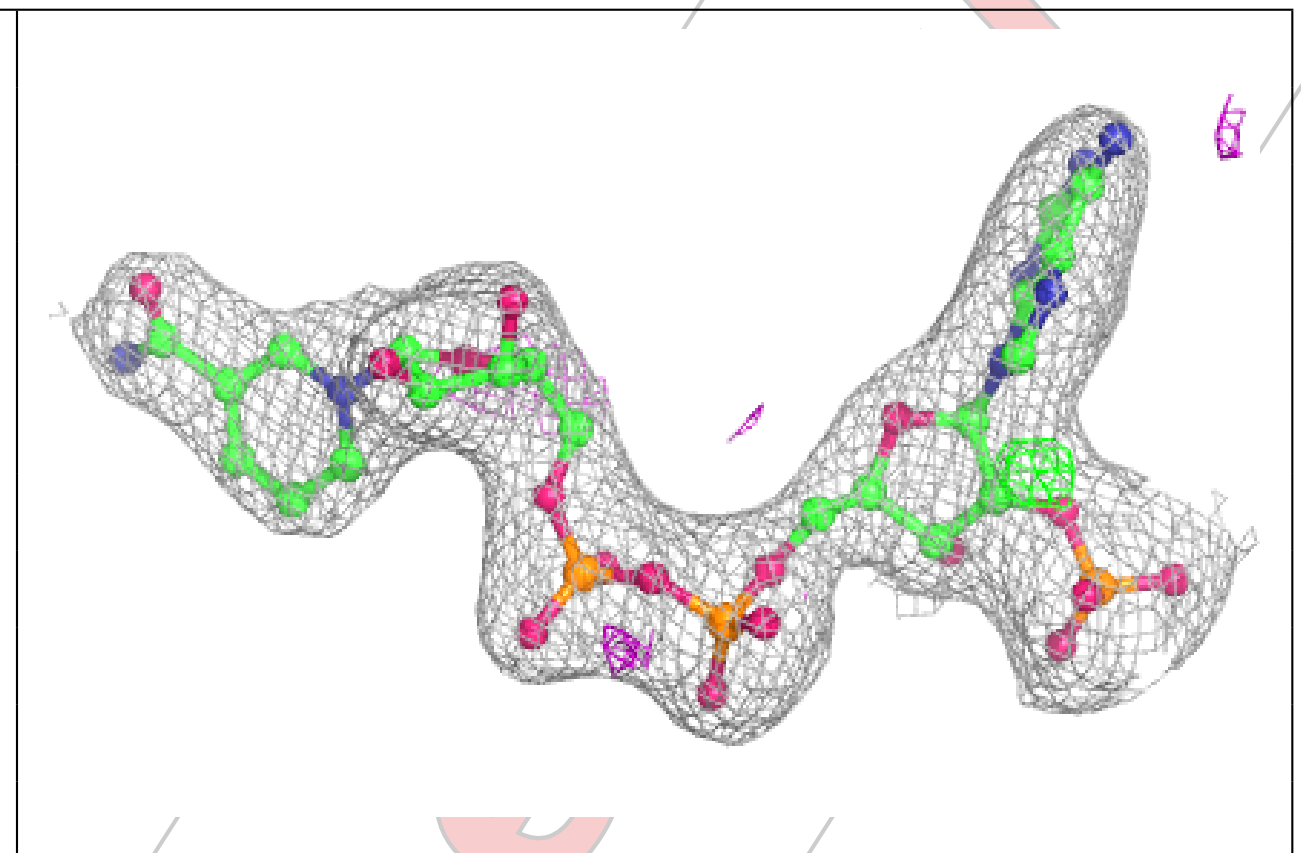

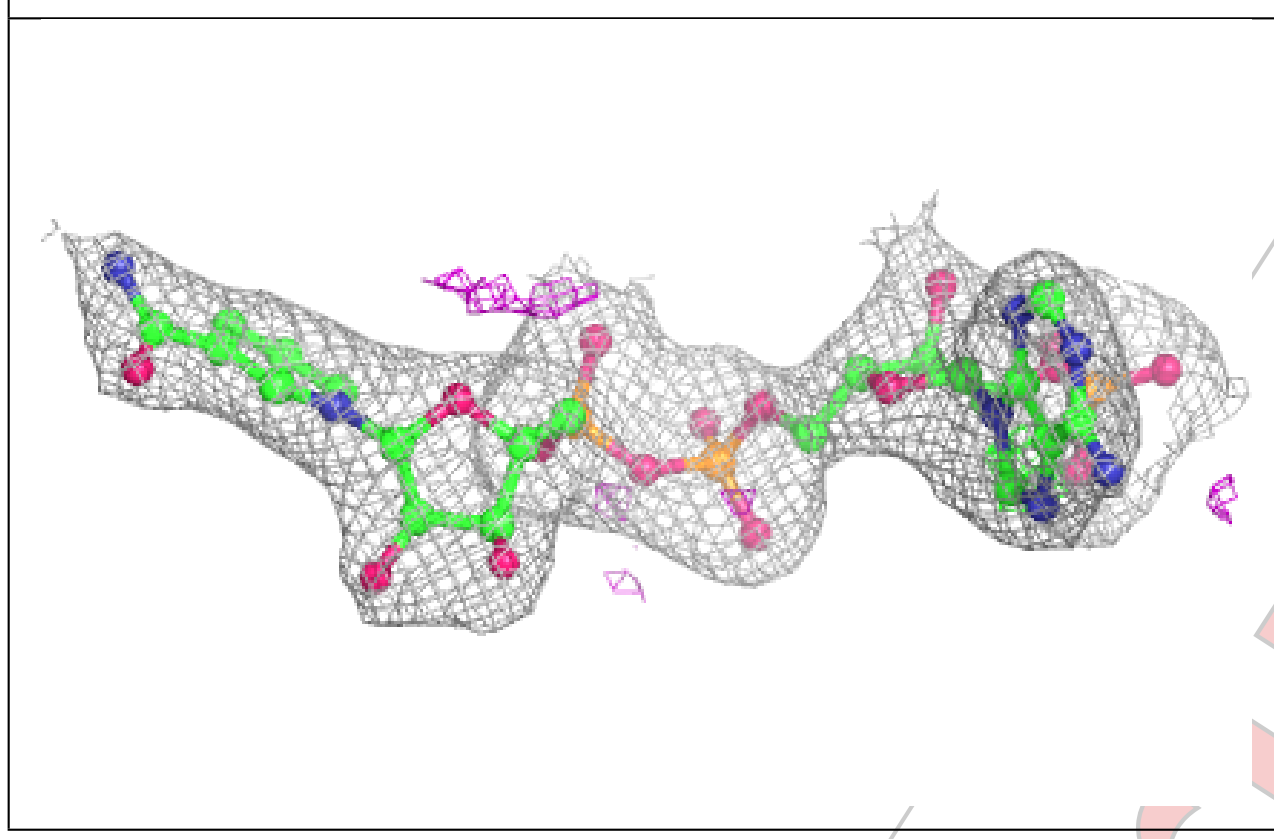

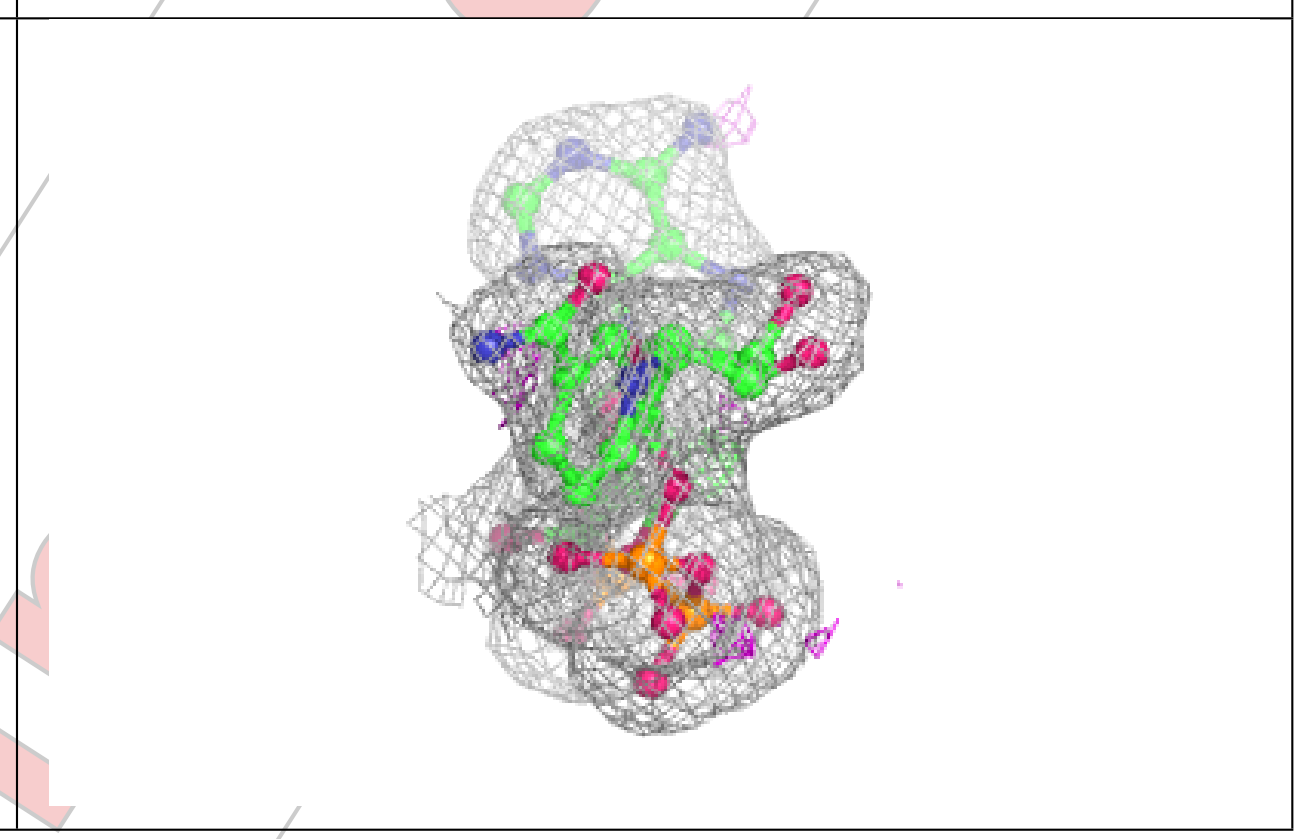

For Manuscript Review

Electron density around MN D 602:

2mF$_o$-DF$_c$ (at 0.7 rmsd) in gray
mF$_o$-DF$_c$ (at 3 rmsd) in purple (negative)
and green (positive)

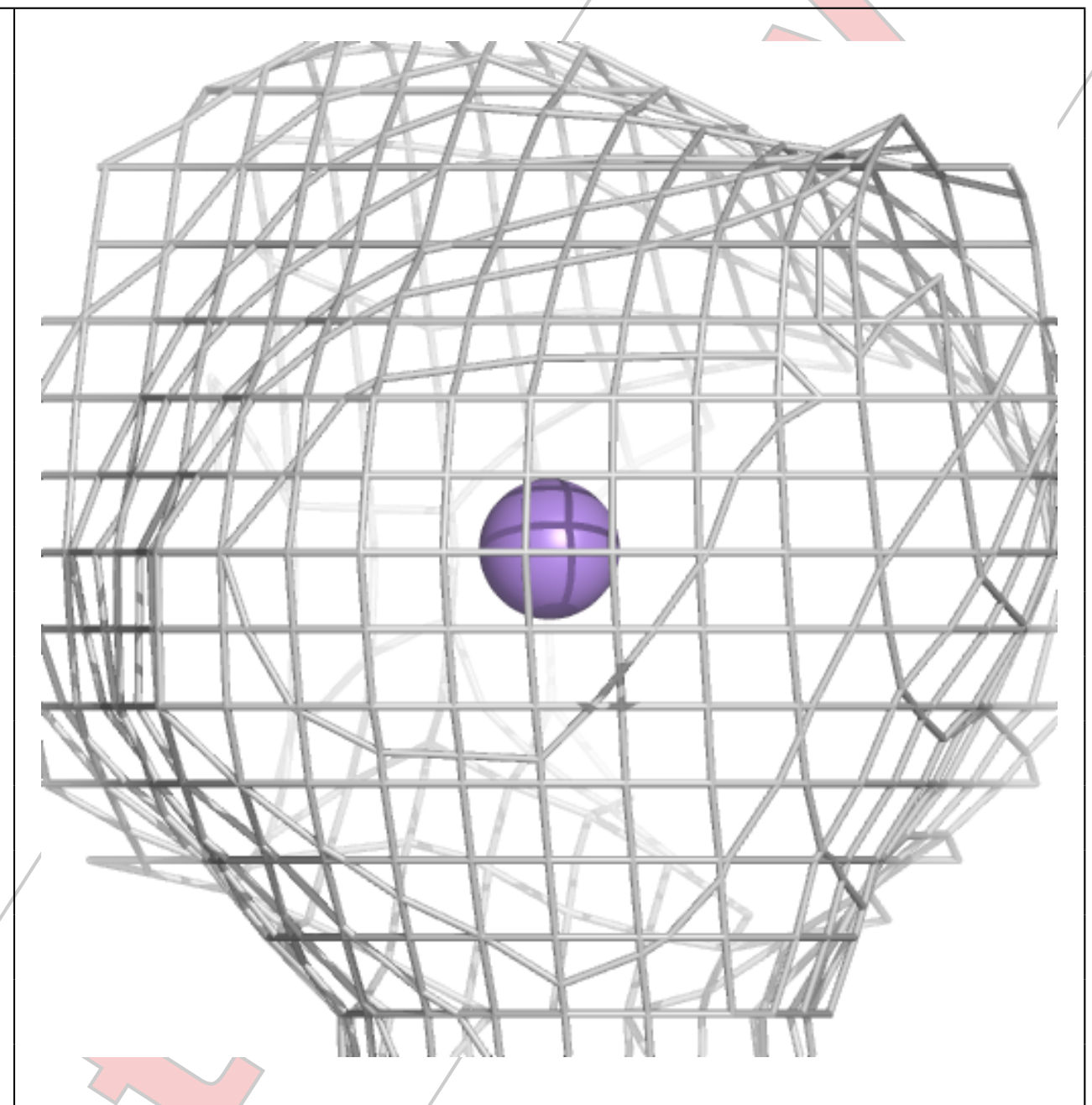

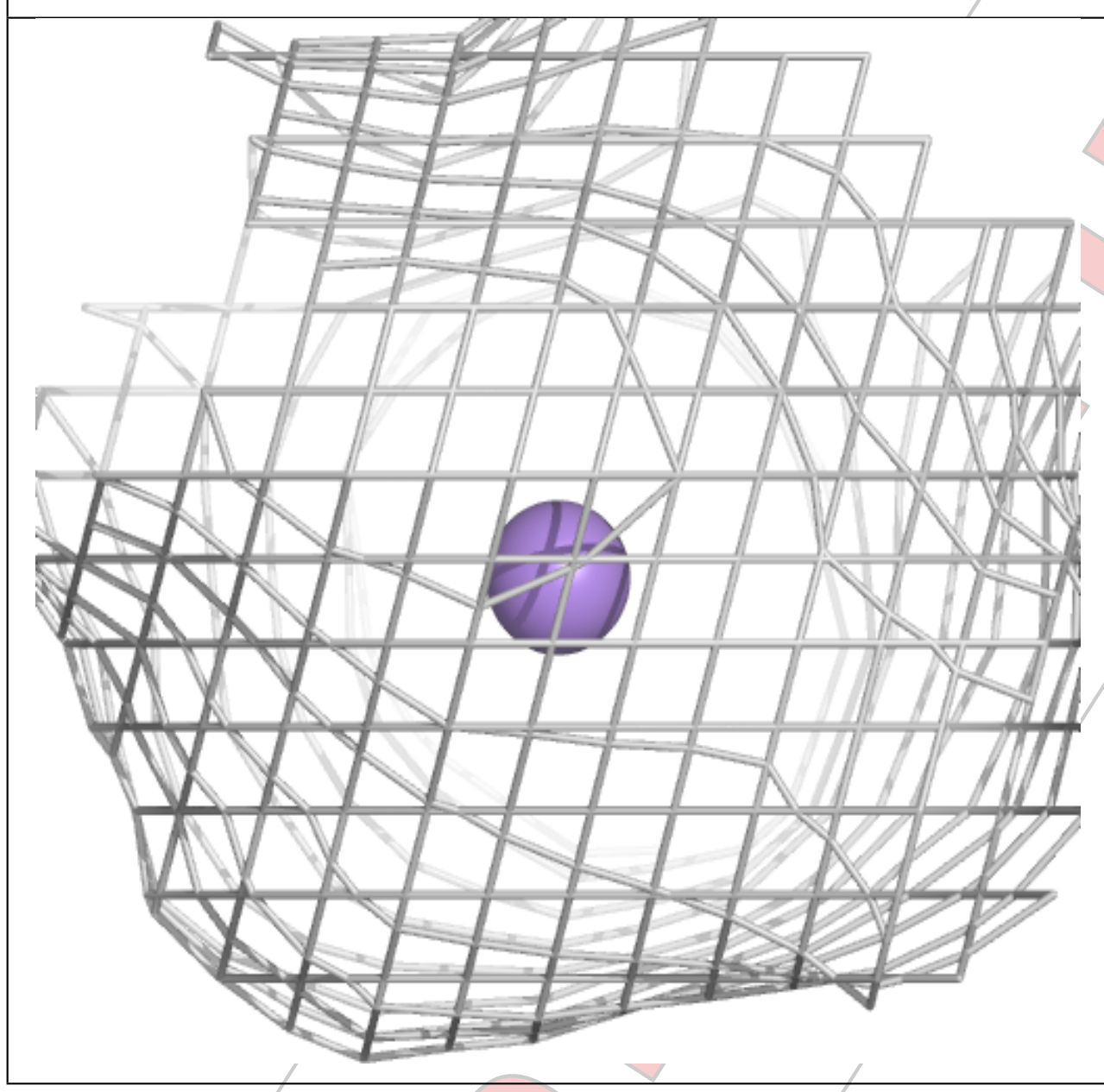

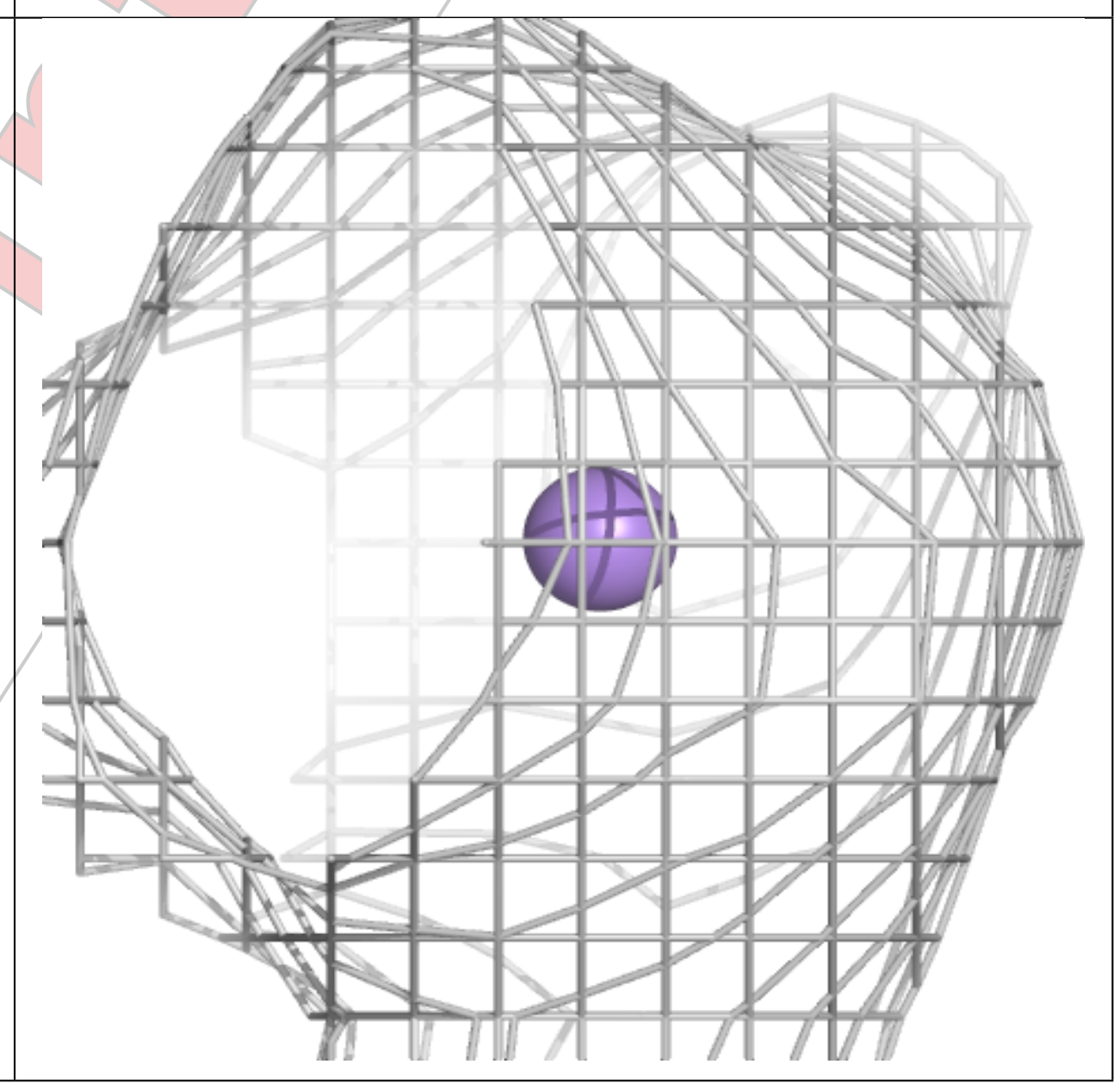

For Manuscript Review

Electron density around MN C 602:

$2mF_o$-DF_c (at 0.7 rmsd) in gray
mF_o-DF_c (at 3 rmsd) in purple (negative)
and green (positive)

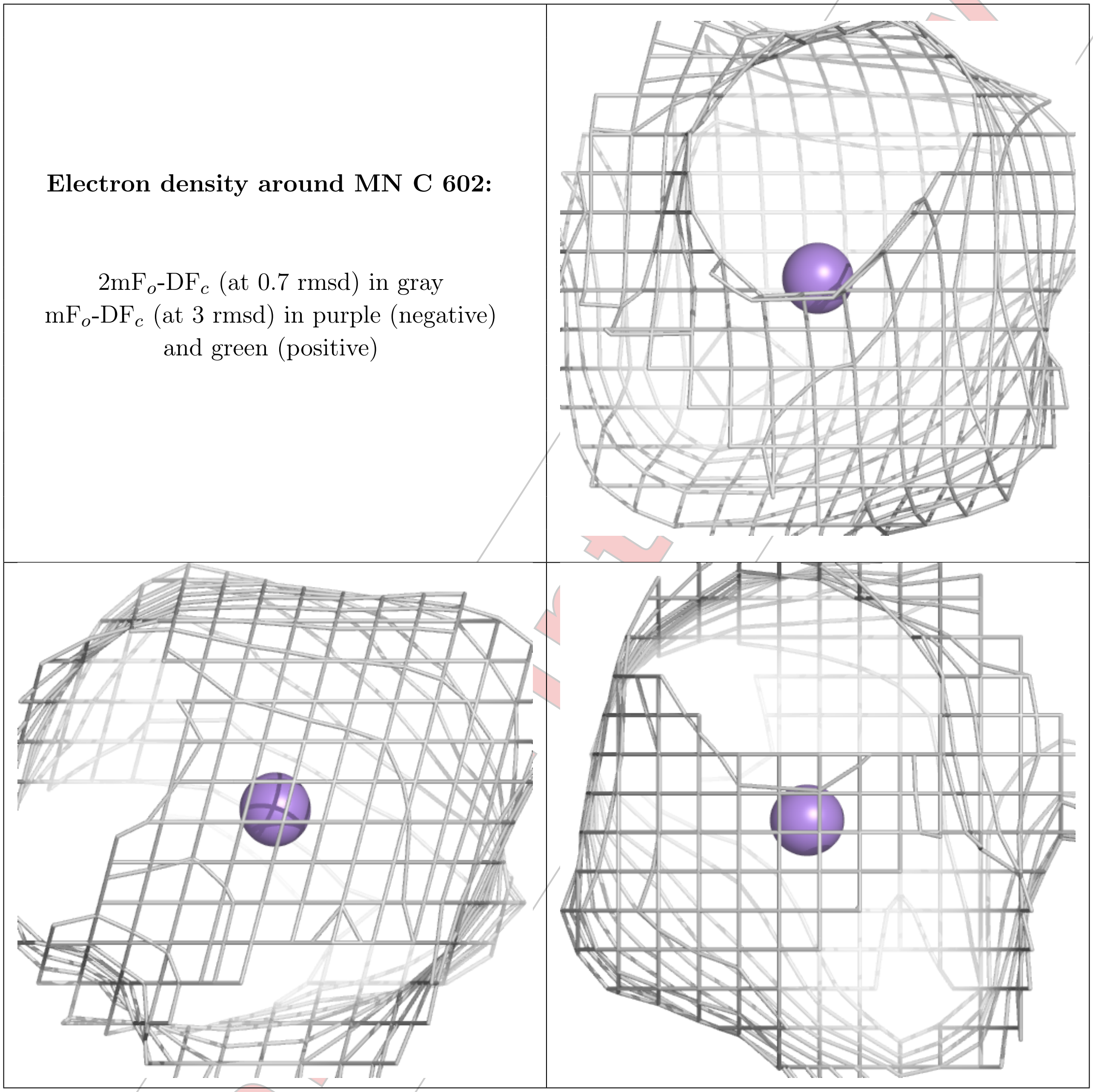

For Manuscript Review

Electron density around MN B 602:

$2mF_o$-DF_c (at 0.7 rmsd) in gray
mF_o-DF_c (at 3 rmsd) in purple (negative)
and green (positive)

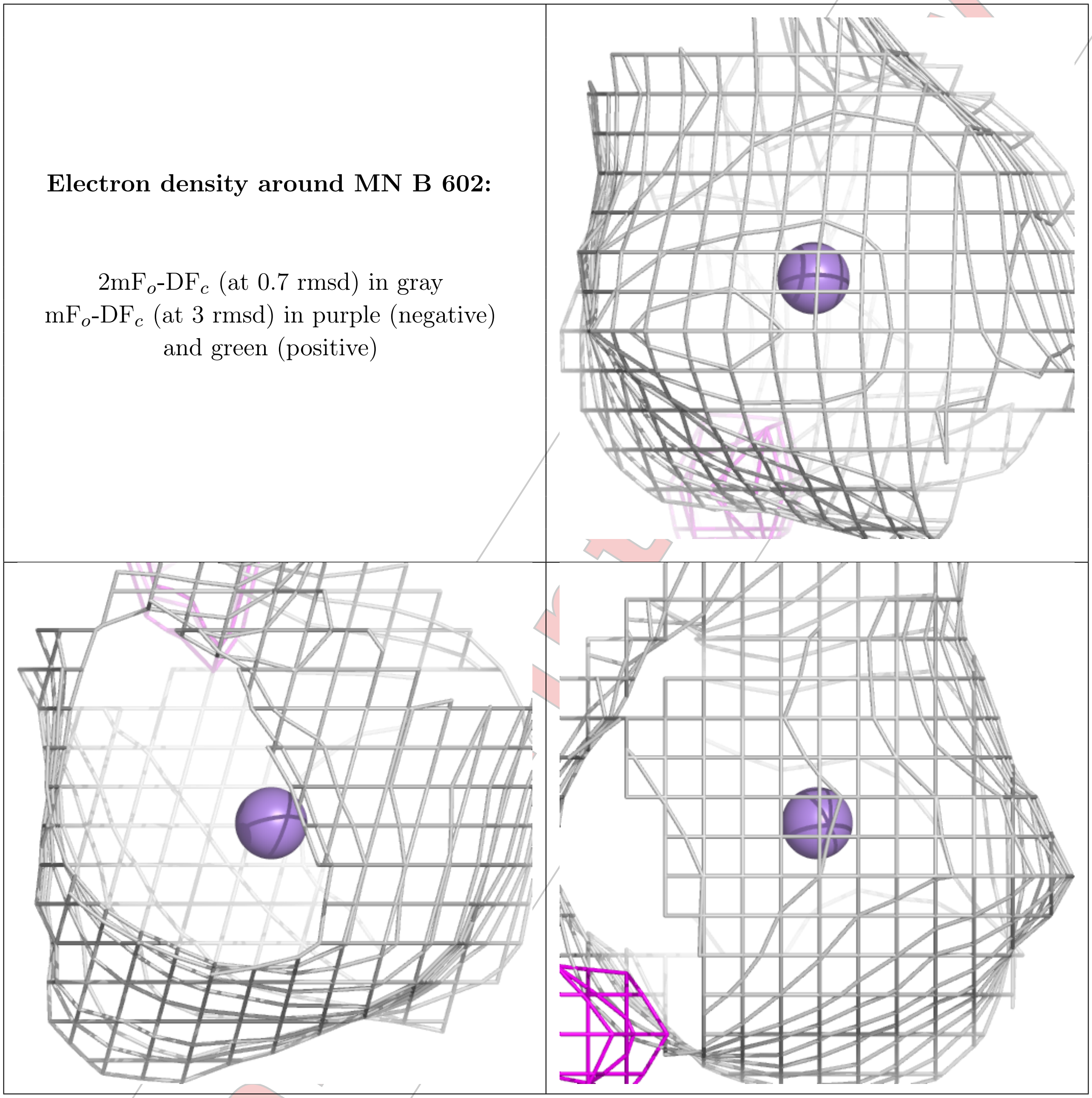

For Manuscript Review

Electron density around MN A 602:

$2mF_o$-DF_c (at 0.7 rmsd) in gray
mF_o-DF_c (at 3 rmsd) in purple (negative)
and green (positive)

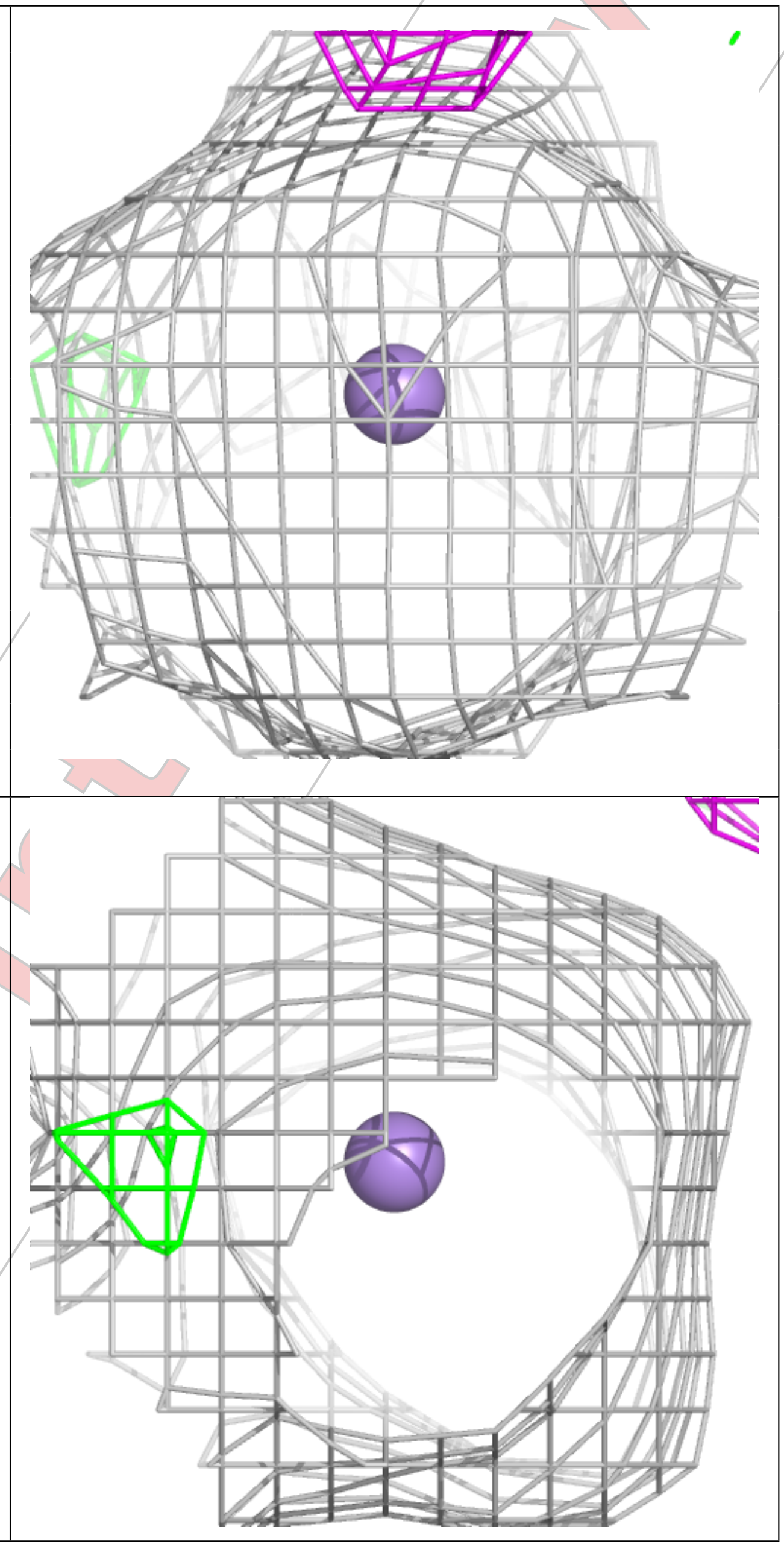

6.5 Other polymers

There are no such residues in this entry.

For Manuscript Review
