# Supplementary material for: Malic enzyme 1 senses L-lactate to determine tumor heterogeneity
Source: Signal Transduct Target Ther. 2026 Sep 11;11:386. doi: 10.1038/s41392-026-02839-6 (PMC13569829; doi:10.1038/s41392-026-02839-6)
Supplement: Supplementary file 3 [file 41392_2026_2839_MOESM5_ESM.pdf]

Full wwPDB X-ray Structure Validation Report (i)

Feb 19, 2026 – 12:48 AM JST

PDB ID	:	23JV / pdb_000023jv
Title	:	Crystal structure of human cytosolic NADP(+)-dependent malic enzyme in complex with small molecules
Deposited on	:	2026-02-09
Resolution	:	2.03 Å(reported)

This wwPDB validation report is for manuscript review

This is a Full wwPDB X-ray Structure Validation Report.

This report is produced by the wwPDB biocuration pipeline after annotation of the structure.

We welcome your comments at *validation@mail.wwpdb.org*
A user guide is available at
https://www.wwpdb.org/validation/2017/XrayValidationReportHelp
with specific help available everywhere you see the (i) symbol.

The types of validation reports are described at
http://www.wwpdb.org/validation/2017/FAQs#types.

For Manuscript Review

The following versions of software and data (see references (i)) were used in the production of this report:

MolProbity	:	4-5-2 with Phenix2.0
Mogul	:	1.8.5 (274361), CSD as541be (2020)
Xtriage (Phenix)	:	2.0
EDS	:	3.0
buster-report	:	1.1.7 (2018)
Percentile statistics	:	20231227.v01 (using entries in the PDB archive December 27th 2023)
CCP4	:	9.0.010 (Gargrove)
Density-Fitness	:	1.0.12
Ideal geometry (proteins)	:	Engh & Huber (2001)

1 Overall quality at a glance (i)

The following experimental techniques were used to determine the structure:
X-RAY DIFFRACTION

The reported resolution of this entry is 2.03 Å.

Percentile scores (ranging between 0-100) for global validation metrics of the entry are shown in the following graphic. The table shows the number of entries on which the scores are based.

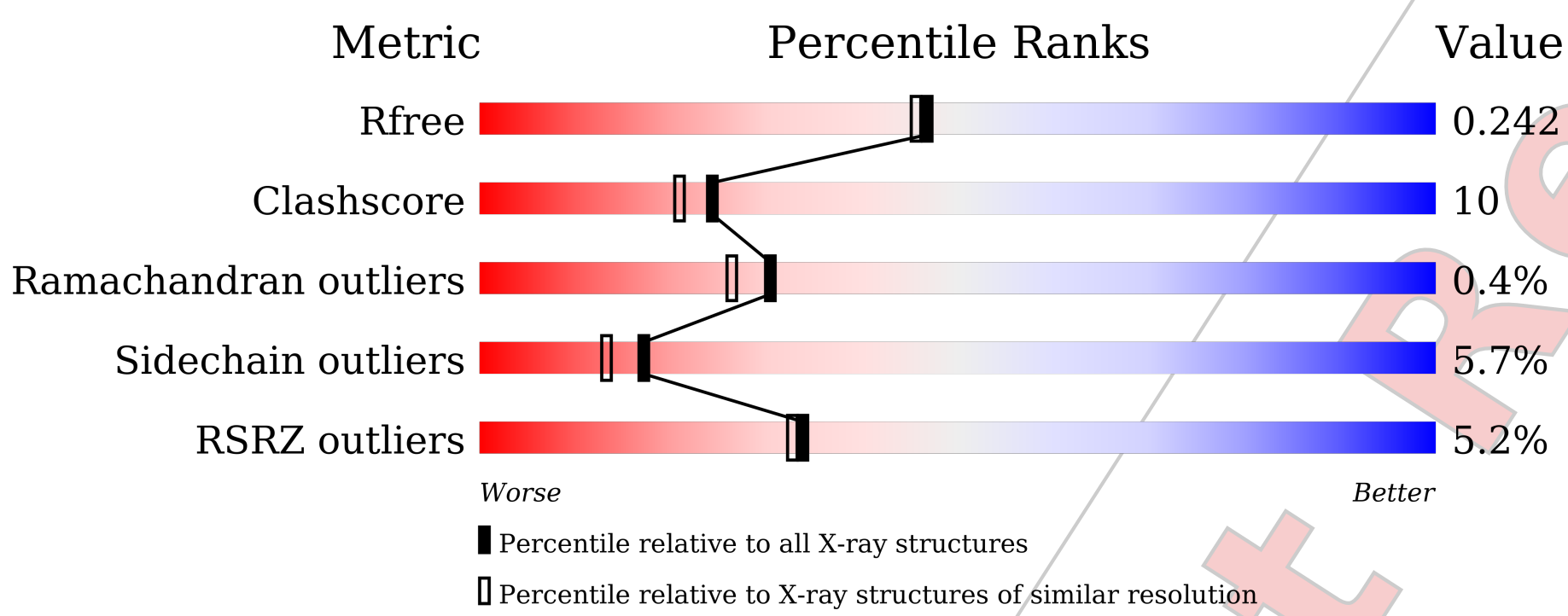

Metric	Whole archive (#Entries)	Similar resolution (#Entries, resolution range(Å))
R_{free}	164625	12358 (2.04-2.00)
Clashscore	180529	13897 (2.04-2.00)
Ramachandran outliers	177936	13770 (2.04-2.00)
Sidechain outliers	177891	13769 (2.04-2.00)
RSRZ outliers	164620	12358 (2.04-2.00)

The table below summarises the geometric issues observed across the polymeric chains and their fit to the electron density. The red, orange, yellow and green segments of the lower bar indicate the fraction of residues that contain outliers for >=3, 2, 1 and 0 types of geometric quality criteria respectively. A grey segment represents the fraction of residues that are not modelled. The numeric value for each fraction is indicated below the corresponding segment, with a dot representing fractions <=5% The upper red bar (where present) indicates the fraction of residues that have poor fit to the electron density. The numeric value is given above the bar.

Mol	Chain	Length	Quality of chain
1	A	572	% 84% 14% ••
1	B	572	3% 81% 16% ••
1	C	572	11% 70% 24% • ••
1	D	572	5% 80% 17% •••

Ideal geometry (DNA, RNA) : Parkinson et al. (1996)
Validation Pipeline (wwPDB-VP) : 2.48

For Manuscript Review

2 Entry composition (i)

There are 5 unique types of molecules in this entry. The entry contains 19256 atoms, of which 0 are hydrogens and 0 are deuteriums.

In the tables below, the ZeroOcc column contains the number of atoms modelled with zero occupancy, the AltConf column contains the number of residues with at least one atom in alternate conformation and the Trace column contains the number of residues modelled with at most 2 atoms.

- Molecule 1 is a protein called NADP-dependent malic enzyme.

Mol	Chain	Residues	Atoms					ZeroOcc	AltConf	Trace
1	A	564	Total 4447	C 2831	N 764	O 832	S 20	0	0	0
1	C	563	Total 4440	C 2827	N 763	O 830	S 20	0	0	0
1	B	563	Total 4442	C 2828	N 763	O 831	S 20	0	0	0
1	D	564	Total 4451	C 2834	N 765	O 832	S 20	0	0	0

- Molecule 2 is NADP NICOTINAMIDE-ADENINE-DINUCLEOTIDE PHOSPHATE (CCD ID: NAP) (formula: $C_{21}H_{28}N_7O_{17}P_3$).

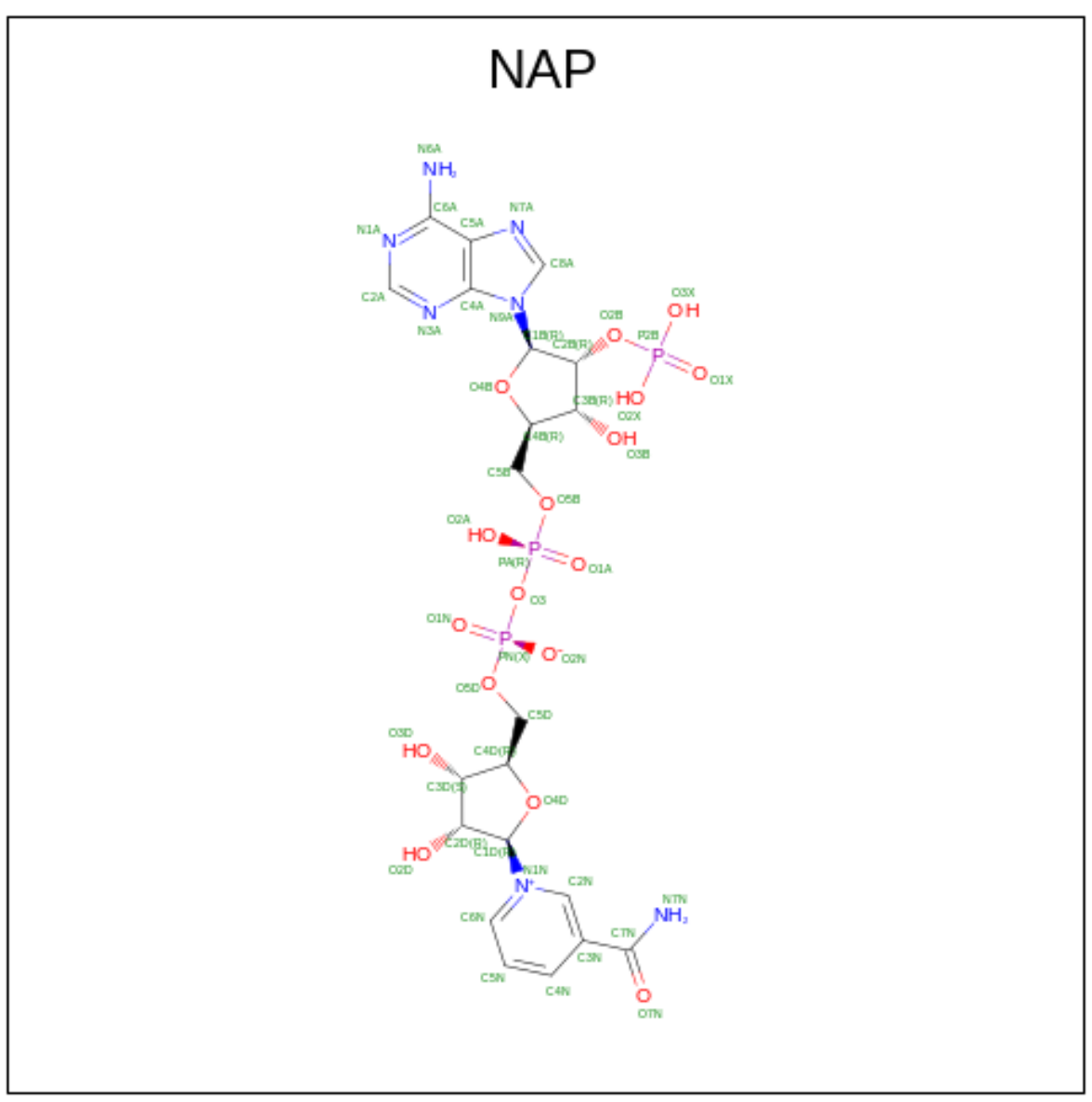

Mol	Chain	Residues	Atoms					ZeroOcc	AltConf
2	A	1	Total 48	C 21	N 7	O 17	P 3	0	0

Continued on next page...

For Manuscript Review

Continued from previous page...

Mol	Chain	Residues	Atoms	ZeroOcc	AltConf
2	C	1	Total C N O P<br>48 21 7 17 3	0	0
2	B	1	Total C N O P<br>48 21 7 17 3	0	0
2	D	1	Total C N O P<br>48 21 7 17 3	0	0

- Molecule 3 is MANGANESE (II) ION (CCD ID: MN) (formula: Mn).

Mol	Chain	Residues	Atoms	ZeroOcc	AltConf
3	A	1	Total Mn<br>1 1	0	0
3	C	1	Total Mn<br>1 1	0	0
3	B	1	Total Mn<br>1 1	0	0
3	D	1	Total Mn<br>1 1	0	0

- Molecule 4 is (2S)-2-HYDROXYPROPANOIC ACID (CCD ID: 2OP) (formula: $C_3H_6O_3$) (labeled as "Ligand of Interest" by depositor).

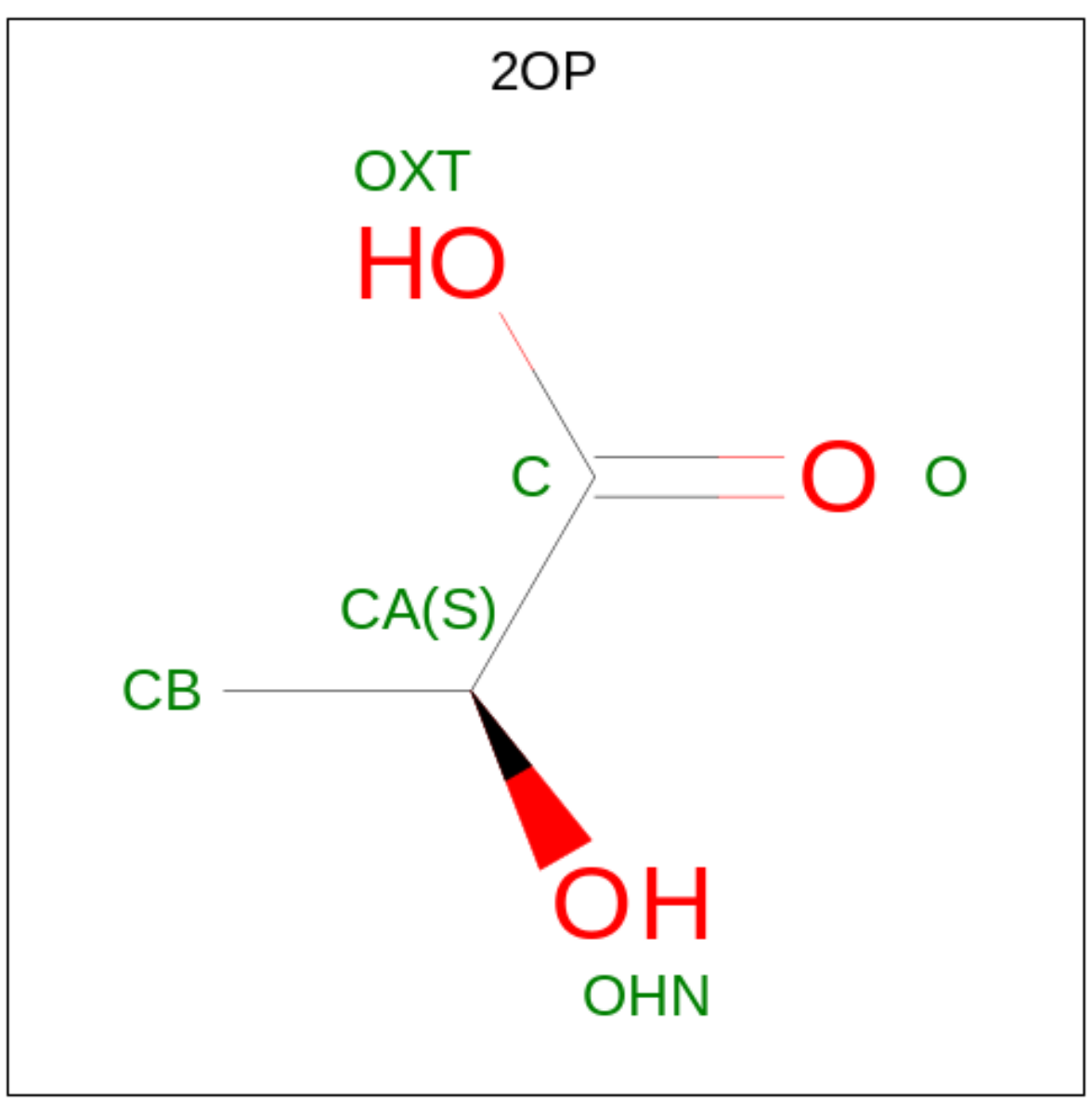

Mol	Chain	Residues	Atoms	ZeroOcc	AltConf
4	C	1	Total C O<br>6 3 3	0	0
4	D	1	Total C O<br>6 3 3	0	0

For Manuscript Review

- Molecule 5 is water.

Mol	Chain	Residues	Atoms	ZeroOcc	AltConf
5	A	404	Total O<br>404 404	0	0
5	C	310	Total O<br>310 310	0	0
5	B	283	Total O<br>283 283	0	0
5	D	271	Total O<br>271 271	0	0

For Manuscript Review

3 Residue-property plots (i)

These plots are drawn for all protein, RNA, DNA and oligosaccharide chains in the entry. The first graphic for a chain summarises the proportions of the various outlier classes displayed in the second graphic. The second graphic shows the sequence view annotated by issues in geometry and electron density. Residues are color-coded according to the number of geometric quality criteria for which they contain at least one outlier: green = 0, yellow = 1, orange = 2 and red = 3 or more. A red dot above a residue indicates a poor fit to the electron density (RSRZ > 2). Stretches of 2 or more consecutive residues without any outlier are shown as a green connector. Residues present in the sample, but not in the model, are shown in grey.

- Molecule 1: NADP-dependent malic enzyme

Chain A:

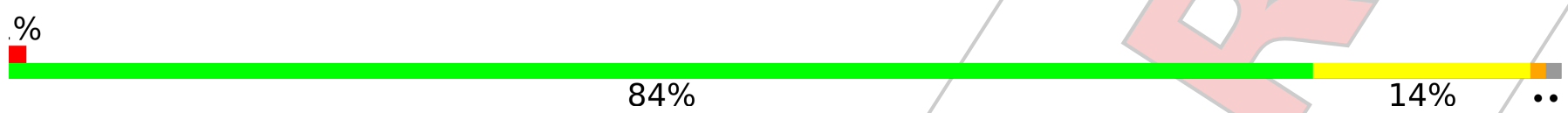

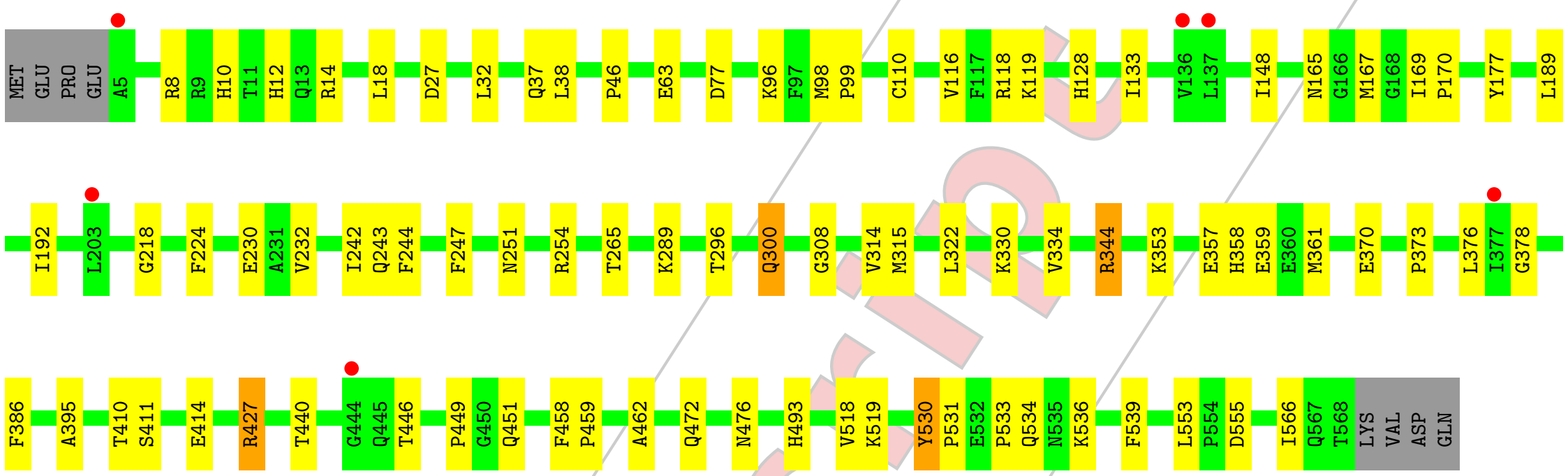

- Molecule 1: NADP-dependent malic enzyme

Chain C:

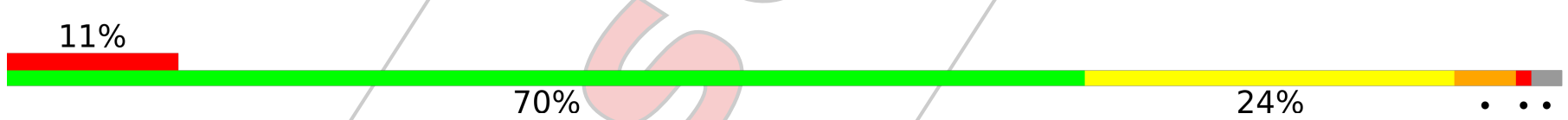

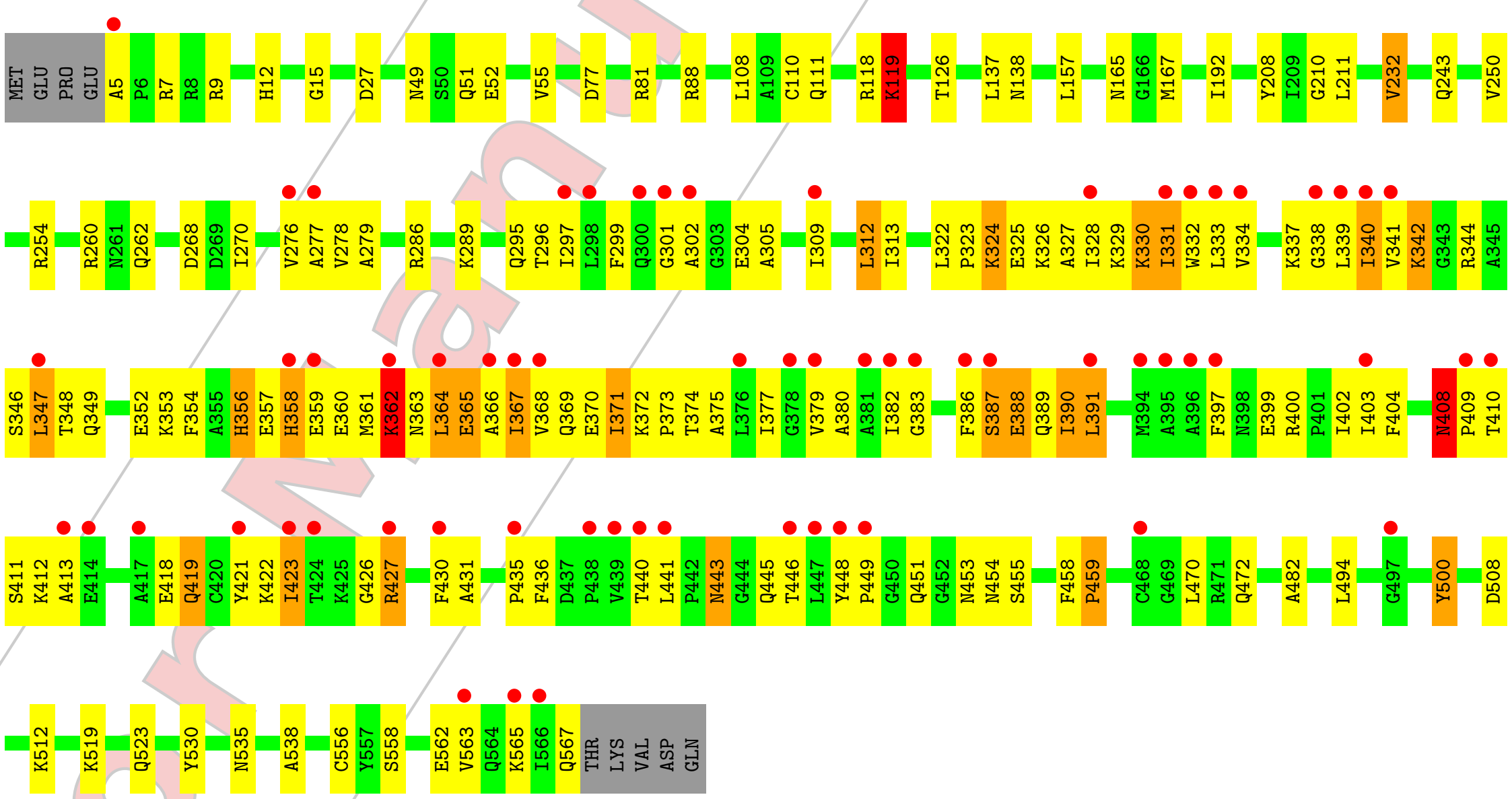

- Molecule 1: NADP-dependent malic enzyme

For Manuscript Review

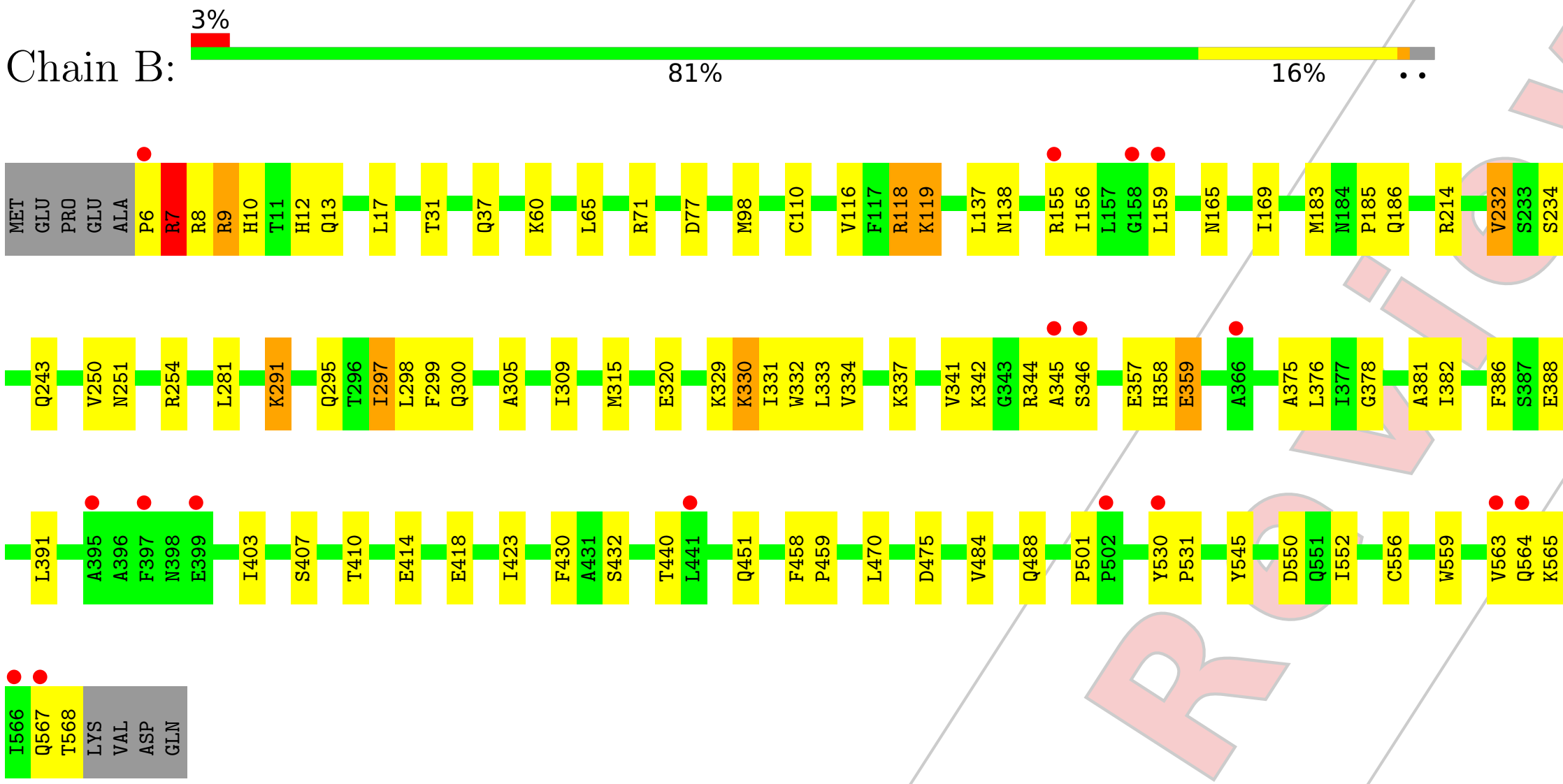

• Molecule 1: NADP-dependent malic enzyme

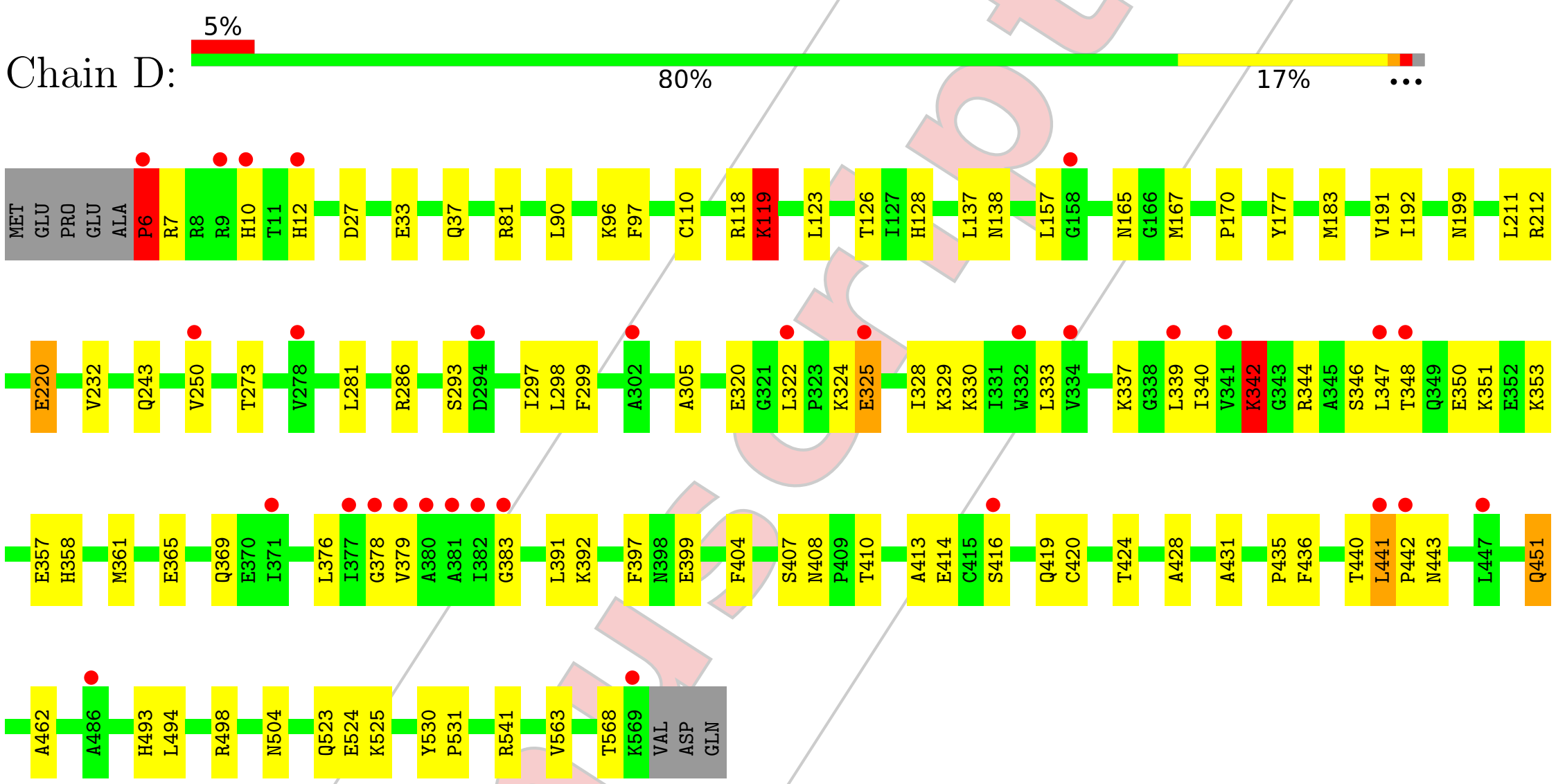

For Manuscript Review

4 Data and refinement statistics (i)

Property	Value	Source
Space group	P 1 21 1	Depositor
Cell constants<br>a, b, c, α, β, γ	70.55Å 180.59Å 108.04Å<br>90.00° 106.09° 90.00°	Depositor
Resolution (Å)	32.96 – 2.03<br>32.96 – 2.03	Depositor<br>EDS
% Data completeness<br>(in resolution range)	90.6 (32.96-2.03)<br>91.1 (32.96-2.03)	Depositor<br>EDS
R_{merge}	0.10	Depositor
R_{sym}	(Not available)	Depositor
$< I/\sigma(I) >$ [1]	2.04 (at 2.03Å)	Xtriage
Refinement program	REFMAC 5.8.0258	Depositor
R, R_{free}	0.177 , 0.238<br>0.188 , 0.242	Depositor<br>DCC
R_{free} test set	7504 reflections (4.46%)	wwPDB-VP
Wilson B-factor (Å^2)	34.7	Xtriage
Anisotropy	0.050	Xtriage
Bulk solvent k_{sol}(e/Å^3), B_{sol}(Å^2)	0.34 , 34.5	EDS
L-test for twinning[2]	$< \|L\| > = 0.49$, $< L^2 > = 0.32$	Xtriage
Estimated twinning fraction	0.022 for h,-k,-h-l	Xtriage
F_o,F_c correlation	0.96	EDS
Total number of atoms	19256	wwPDB-VP
Average B, all atoms (Å^2)	45.0	wwPDB-VP

Xtriage's analysis on translational NCS is as follows: *The largest off-origin peak in the Patterson function is 3.42% of the height of the origin peak. No significant pseudotranslation is detected.*

[1] Intensities estimated from amplitudes.

[2] Theoretical values of $< |L| >$, $< L^2 >$ for acentric reflections are 0.5, 0.333 respectively for untwinned datasets, and 0.375, 0.2 for perfectly twinned datasets.

For Manuscript Review

5 Model quality (i)

5.1 Standard geometry (i)

Bond lengths and bond angles in the following residue types are not validated in this section: 2OP, MN, NAP

The Z score for a bond length (or angle) is the number of standard deviations the observed value is removed from the expected value. A bond length (or angle) with $|Z| > 5$ is considered an outlier worth inspection. RMSZ is the root-mean-square of all Z scores of the bond lengths (or angles).

Mol	Chain	Bond lengths		Bond angles	
		RMSZ	#|Z| >5	RMSZ	#|Z| >5
1	A	1.08	2/4534 (0.0%)	1.37	5/6139 (0.1%)
1	B	1.10	1/4529 (0.0%)	1.39	1/6131 (0.0%)
1	C	1.11	4/4527 (0.1%)	1.43	18/6129 (0.3%)
1	D	1.12	3/4538 (0.1%)	1.42	15/6142 (0.2%)
All	All	1.10	10/18128 (0.1%)	1.40	39/24541 (0.2%)

Chiral center outliers are detected by calculating the chiral volume of a chiral center and verifying if the center is modelled as a planar moiety or with the opposite hand.A planarity outlier is detected by checking planarity of atoms in a peptide group, atoms in a mainchain group or atoms of a sidechain that are expected to be planar.

Mol	Chain	#Chirality outliers	#Planarity outliers
1	C	0	1

All (10) bond length outliers are listed below:

Mol	Chain	Res	Type	Atoms	Z	Observed(Å)	Ideal(Å)
1	C	500	TYR	C-N	14.38	1.50	1.33
1	C	119	LYS	C-N	12.65	1.49	1.33
1	D	119	LYS	C-N	11.50	1.48	1.33
1	D	408	ASN	C-N	8.32	1.50	1.34
1	C	455	SER	C-O	-7.75	1.14	1.24
1	B	501	PRO	C-O	-7.53	1.18	1.24
1	C	459	PRO	C-O	-6.20	1.15	1.24
1	D	191	VAL	C-O	5.80	1.29	1.24
1	A	308	GLY	C-O	5.52	1.30	1.23
1	A	449	PRO	C-O	-5.21	1.17	1.23

All (39) bond angle outliers are listed below:

For Manuscript Review

Mol	Chain	Res	Type	Atoms	Z	Observed(°)	Ideal(°)
1	C	500	TYR	O-C-N	-12.73	113.56	121.71
1	C	119	LYS	CA-C-N	10.70	130.82	119.90
1	C	119	LYS	C-N-CA	10.70	130.82	119.90
1	D	119	LYS	CA-C-N	10.62	130.70	119.76
1	D	119	LYS	C-N-CA	10.62	130.70	119.76
1	C	500	TYR	CA-C-N	10.02	130.70	120.38
1	C	500	TYR	C-N-CA	10.02	130.70	120.38
1	D	119	LYS	CB-CA-C	-9.37	102.00	110.44
1	D	27	ASP	CB-CA-C	-8.05	105.06	116.34
1	A	27	ASP	CB-CA-C	-6.76	108.76	116.54
1	C	27	ASP	CB-CA-C	6.33	123.03	110.42
1	A	344	ARG	CG-CD-NE	-6.31	98.12	112.00
1	D	407	SER	O-C-N	6.20	130.17	122.86
1	B	6	PRO	CA-N-CD	-6.19	103.33	112.00
1	D	451	GLN	CA-C-N	5.69	125.74	120.34
1	D	451	GLN	C-N-CA	5.69	125.74	120.34
1	D	524	GLU	CA-C-O	-5.57	112.54	119.28
1	C	455	SER	CA-C-O	-5.51	112.15	119.10
1	A	530	TYR	CB-CA-C	5.43	117.28	108.87
1	C	119	LYS	CB-CA-C	-5.39	104.83	110.33
1	D	119	LYS	O-C-N	-5.36	115.43	120.85
1	C	448	TYR	CB-CA-C	5.34	115.78	110.33
1	C	232	VAL	CB-CA-C	5.29	118.95	112.02
1	C	387	SER	N-CA-C	5.25	121.98	110.80
1	A	218	GLY	CA-C-N	5.22	127.28	120.28
1	A	218	GLY	C-N-CA	5.22	127.28	120.28
1	D	408	ASN	O-C-N	5.17	126.52	121.66
1	C	49	ASN	CA-CB-CG	5.13	117.73	112.60
1	C	386	PHE	CA-C-N	5.11	131.31	121.54
1	C	386	PHE	C-N-CA	5.11	131.31	121.54
1	C	530	TYR	CB-CA-C	5.09	116.76	108.87
1	C	119	LYS	O-C-N	-5.08	115.45	120.83
1	D	410	THR	CA-C-N	5.06	127.57	120.28
1	D	410	THR	C-N-CA	5.06	127.57	120.28
1	D	6	PRO	CA-N-CD	-5.06	104.92	112.00
1	C	363	ASN	CA-C-N	5.05	131.19	121.54
1	C	363	ASN	C-N-CA	5.05	131.19	121.54
1	D	199	ASN	CA-C-N	5.01	126.96	120.44
1	D	199	ASN	C-N-CA	5.01	126.96	120.44

There are no chirality outliers.

All (1) planarity outliers are listed below:

For Manuscript Review

Mol	Chain	Res	Type	Group
1	C	408	ASN	Peptide

5.2 Too-close contacts (i)

In the following table, the Non-H and H(model) columns list the number of non-hydrogen atoms and hydrogen atoms in the chain respectively. The H(added) column lists the number of hydrogen atoms added and optimized by MolProbity. The Clashes column lists the number of clashes within the asymmetric unit, whereas Symm-Clashes lists symmetry-related clashes.

Mol	Chain	Non-H	H(model)	H(added)	Clashes	Symm-Clashes
1	A	4447	0	4474	53	0
1	B	4442	0	4470	73	0
1	C	4440	0	4467	164	0
1	D	4451	0	4483	74	0
2	A	48	0	25	0	0
2	B	48	0	25	0	0
2	C	48	0	25	4	0
2	D	48	0	25	6	0
3	A	1	0	0	0	0
3	B	1	0	0	0	0
3	C	1	0	0	0	0
3	D	1	0	0	0	0
4	C	6	0	4	2	0
4	D	6	0	4	2	0
5	A	404	0	0	13	0
5	B	283	0	0	20	0
5	C	310	0	0	17	0
5	D	271	0	0	22	0
All	All	19256	0	18002	354	0

The all-atom clashscore is defined as the number of clashes found per 1000 atoms (including hydrogen atoms). The all-atom clashscore for this structure is 10.

All (354) close contacts within the same asymmetric unit are listed below, sorted by their clash magnitude.

Atom-1	Atom-2	Interatomic distance (Å)	Clash overlap (Å)
1:C:337:LYS:O	1:C:362:LYS:HG3	1.35	1.23
1:B:331:ILE:HA	5:B:716:HOH:O	1.49	1.12
1:A:37:GLN:HG3	5:A:1048:HOH:O	1.51	1.07
1:C:296:THR:H	1:C:374:THR:HG22	1.02	1.06
1:C:361:MET:HE1	1:C:370:GLU:HG2	1.34	1.06

Continued on next page...

For Manuscript Review

Continued from previous page...

Atom-1	Atom-2	Interatomic distance (Å)	Clash overlap (Å)
1:C:296:THR:H	1:C:374:THR:CG2	1.69	1.05
1:D:416:SER:HB3	5:D:1218:HOH:O	1.59	1.01
1:C:250:VAL:HG13	5:C:888:HOH:O	1.66	0.94
1:B:138:ASN:HB3	1:D:10:HIS:CD2	2.03	0.93
1:D:305:ALA:HB2	2:D:601:NAP:H52N	1.49	0.91
1:C:119:LYS:HB2	5:C:718:HOH:O	1.72	0.89
1:C:296:THR:N	1:C:374:THR:HG22	1.86	0.89
1:C:556:CYS:SG	5:D:1011:HOH:O	2.30	0.88
1:A:361:MET:HE1	1:A:370:GLU:HG2	1.57	0.87
5:A:863:HOH:O	1:B:568:THR:HA	1.73	0.86
1:B:358:HIS:CE1	5:B:726:HOH:O	2.28	0.86
1:B:358:HIS:HE1	5:B:726:HOH:O	1.56	0.86
1:C:332:TRP:CG	1:C:371:ILE:HD13	2.10	0.85
1:C:435:PRO:HA	1:C:451:GLN:HE22	1.39	0.85
1:A:358:HIS:NE2	1:A:361:MET:HE2	1.90	0.85
1:C:500:TYR:HE1	5:C:706:HOH:O	1.59	0.85
1:C:329:LYS:HA	1:C:356:HIS:NE2	1.92	0.83
1:C:361:MET:CE	1:C:370:GLU:HG2	2.08	0.83
1:D:305:ALA:CB	2:D:601:NAP:H52N	2.07	0.83
1:C:361:MET:HE1	1:C:370:GLU:CG	2.08	0.82
1:A:358:HIS:NE2	1:A:361:MET:CE	2.42	0.82
1:D:123:LEU:HD21	1:D:137:LEU:HD12	1.59	0.82
1:C:348:THR:O	1:C:352:GLU:HG2	1.78	0.82
1:C:295:GLN:HA	1:C:374:THR:HG21	1.60	0.81
1:B:330:LYS:HD2	5:B:798:HOH:O	1.78	0.81
5:A:970:HOH:O	1:B:556:CYS:SG	2.37	0.80
1:C:297:ILE:CG2	1:C:377:ILE:HD12	2.12	0.78
1:C:391:LEU:HD22	1:C:391:LEU:H	1.48	0.78
1:A:119:LYS:HB3	5:A:753:HOH:O	1.84	0.77
1:A:118:ARG:HD3	5:A:715:HOH:O	1.84	0.77
1:C:295:GLN:CA	1:C:374:THR:HG21	2.14	0.77
1:C:332:TRP:CG	1:C:371:ILE:CD1	2.67	0.76
1:B:342:LYS:HE3	1:B:357:GLU:HG2	1.67	0.76
1:D:157:LEU:HD23	4:D:602:2OP:HA	1.67	0.76
1:C:409:PRO:HD2	1:C:412:LYS:HG3	1.68	0.75
1:D:138:ASN:HB2	5:D:1222:HOH:O	1.87	0.75
1:B:12:HIS:CD2	5:B:929:HOH:O	2.41	0.73
1:C:348:THR:HG21	5:C:983:HOH:O	1.88	0.73
1:C:440:THR:HG22	1:C:446:THR:HG22	1.71	0.72
2:D:601:NAP:H6N	2:D:601:NAP:H3D	1.71	0.72
1:C:15:GLY:HA3	1:D:12:HIS:CE1	2.25	0.71

Continued on next page...

For Manuscript Review

Continued from previous page...

Atom-1	Atom-2	Interatomic distance (Å)	Clash overlap (Å)
1:B:388:GLU:HA	1:B:423:ILE:HD13	1.73	0.71
1:C:366:ALA:HB2	1:C:390:ILE:HG13	1.71	0.71
1:C:567:GLN:C	5:C:827:HOH:O	2.34	0.70
1:C:331:ILE:HD13	1:C:354:PHE:HE1	1.57	0.70
1:B:382:ILE:HB	5:B:816:HOH:O	1.91	0.70
1:C:364:LEU:HD12	1:C:364:LEU:O	1.92	0.69
1:B:155:ARG:HD2	5:B:743:HOH:O	1.93	0.69
1:C:404:PHE:CG	1:C:431:ALA:HB3	2.27	0.69
1:B:118:ARG:CG	1:B:118:ARG:HH11	2.05	0.69
1:D:220:GLU:HG3	5:D:1224:HOH:O	1.91	0.69
1:C:361:MET:O	1:C:361:MET:HG3	1.92	0.69
1:C:391:LEU:HD22	1:C:391:LEU:N	2.08	0.69
1:C:277:ALA:HB3	1:C:309:ILE:HG12	1.74	0.68
1:C:296:THR:N	1:C:374:THR:CG2	2.50	0.68
1:C:332:TRP:CD1	1:C:371:ILE:HD13	2.26	0.68
1:B:118:ARG:HH11	1:B:118:ARG:HG2	1.59	0.68
1:D:320:GLU:HG3	1:D:320:GLU:O	1.92	0.68
1:C:118:ARG:HD2	5:B:812:HOH:O	1.94	0.68
1:C:391:LEU:H	1:C:391:LEU:CD2	2.05	0.68
1:D:305:ALA:HB2	2:D:601:NAP:C5D	2.23	0.68
1:D:358:HIS:HE1	5:D:1210:HOH:O	1.76	0.67
1:B:65:LEU:O	1:B:71:ARG:NH2	2.28	0.67
1:D:416:SER:HA	1:D:436:PHE:CZ	2.30	0.67
1:B:567:GLN:HE22	1:D:212:ARG:HH21	1.41	0.66
1:D:118:ARG:HD2	5:D:1160:HOH:O	1.94	0.66
1:C:305:ALA:HB3	1:C:379:VAL:HG21	1.77	0.66
1:B:138:ASN:HB3	1:D:10:HIS:CG	2.31	0.66
1:C:375:ALA:CB	1:C:402:ILE:HB	2.26	0.66
1:A:128:HIS:CD2	1:C:563:VAL:HG21	2.32	0.65
1:D:435:PRO:HA	1:D:451:GLN:HE22	1.62	0.65
1:B:300:GLN:HE21	1:B:300:GLN:HA	1.63	0.64
1:C:443:ASN:HD22	1:C:443:ASN:H	1.45	0.64
1:B:98:MET:HE3	5:B:789:HOH:O	1.96	0.63
1:C:443:ASN:HD22	1:C:443:ASN:N	1.96	0.63
1:C:366:ALA:CB	1:C:390:ILE:HG13	2.27	0.63
1:C:332:TRP:CD2	1:C:371:ILE:HD11	2.34	0.63
1:C:337:LYS:C	1:C:362:LYS:HG3	2.21	0.63
1:C:262:GLN:HB2	5:C:967:HOH:O	2.00	0.61
1:C:408:ASN:HD22	1:C:409:PRO:HA	1.65	0.61
1:C:270:ILE:HA	2:C:601:NAP:H71N	1.65	0.61
1:C:369:GLN:HA	1:C:397:PHE:CZ	2.35	0.61

Continued on next page...

For Manuscript Review

Continued from previous page...

Atom-1	Atom-2	Interatomic distance (Å)	Clash overlap (Å)
1:C:427:ARG:CG	1:C:427:ARG:HH11	2.13	0.61
1:C:297:ILE:CG2	1:C:377:ILE:CD1	2.79	0.61
1:C:427:ARG:HH11	1:C:427:ARG:HG3	1.66	0.61
5:B:820:HOH:O	1:D:10:HIS:CD2	2.52	0.61
1:D:339:LEU:HD22	1:D:361:MET:HE3	1.83	0.61
1:C:305:ALA:CB	1:C:379:VAL:HG21	2.30	0.61
1:D:123:LEU:CD2	1:D:137:LEU:HD12	2.31	0.61
1:B:9:ARG:O	1:B:9:ARG:HG2	1.99	0.61
1:B:391:LEU:HD12	1:B:423:ILE:HD12	1.83	0.60
1:C:361:MET:HE1	1:C:370:GLU:CB	2.31	0.60
1:D:157:LEU:CD2	4:D:602:2OP:HA	2.31	0.60
1:C:403:ILE:O	1:C:430:PHE:HA	2.02	0.60
1:B:346:SER:N	5:B:702:HOH:O	2.34	0.60
5:A:872:HOH:O	1:B:37:GLN:HG3	2.01	0.59
1:D:167:MET:HE2	1:D:192:ILE:HG22	1.85	0.59
1:D:391:LEU:HD13	1:D:420:CYS:HA	1.85	0.59
1:B:9:ARG:O	1:B:552:ILE:HA	2.02	0.59
1:B:484:VAL:O	1:B:488:GLN:HG2	2.03	0.59
1:A:395:ALA:O	1:A:427:ARG:NH1	2.34	0.59
1:C:254:ARG:NH2	5:C:703:HOH:O	2.34	0.59
1:B:559:TRP:H	1:B:564:GLN:NE2	2.01	0.59
1:B:110:CYS:O	1:B:165:ASN:HB3	2.02	0.58
1:A:493:HIS:CD2	5:A:873:HOH:O	2.55	0.58
1:D:299:PHE:CD2	5:D:1036:HOH:O	2.52	0.58
1:A:110:CYS:O	1:A:165:ASN:HB3	2.03	0.58
1:C:362:LYS:HB3	1:C:362:LYS:HZ3	1.68	0.58
1:D:392:LYS:NZ	5:D:1005:HOH:O	2.37	0.58
1:C:338:GLY:HA3	1:C:362:LYS:CG	2.33	0.57
1:B:295:GLN:O	1:B:330:LYS:HG3	2.04	0.57
1:C:322:LEU:HD12	1:C:323:PRO:HD2	1.86	0.57
1:C:427:ARG:HB2	1:C:427:ARG:NH1	2.19	0.57
1:D:177:TYR:HE2	1:D:462:ALA:HB2	1.69	0.57
1:C:404:PHE:CD1	1:C:431:ALA:HB3	2.40	0.57
1:A:119:LYS:HE2	1:D:81:ARG:HB2	1.87	0.57
1:C:286:ARG:CZ	1:C:494:LEU:HD11	2.35	0.57
1:C:419:GLN:HE21	1:C:419:GLN:HA	1.70	0.56
1:D:297:ILE:C	5:D:1036:HOH:O	2.48	0.56
1:D:297:ILE:HG22	5:D:1036:HOH:O	2.04	0.56
1:C:330:LYS:HZ3	1:C:330:LYS:HB2	1.70	0.56
1:A:12:HIS:HE1	1:C:138:ASN:OD1	1.89	0.56
1:C:535:ASN:ND2	1:C:538:ALA:H	2.04	0.56

Continued on next page...

For Manuscript Review

Continued from previous page...

Atom-1	Atom-2	Interatomic distance (Å)	Clash overlap (Å)
1:C:375:ALA:HB2	1:C:402:ILE:HD12	1.87	0.56
1:C:435:PRO:HG2	5:C:730:HOH:O	2.05	0.56
1:A:361:MET:CE	1:A:370:GLU:HG2	2.34	0.56
1:B:116:VAL:O	1:B:118:ARG:HG3	2.06	0.55
1:D:383:GLY:O	1:D:413:ALA:O	2.24	0.55
1:C:167:MET:HE2	1:C:192:ILE:HG22	1.87	0.55
1:C:295:GLN:CB	1:C:374:THR:HG21	2.36	0.55
1:D:337:LYS:HD2	1:D:346:SER:HB2	1.86	0.55
1:C:421:TYR:OH	1:C:449:PRO:HA	2.07	0.55
1:C:423:ILE:N	1:C:423:ILE:HD13	2.20	0.55
1:A:32:LEU:HD23	1:B:564:GLN:HE21	1.72	0.55
1:C:371:ILE:HG22	1:C:373:PRO:HD3	1.89	0.54
1:D:441:LEU:HB2	1:D:442:PRO:HD2	1.90	0.54
1:C:383:GLY:HA2	1:C:413:ALA:O	2.07	0.54
1:B:299:PHE:HB2	1:B:333:LEU:HD23	1.88	0.54
1:D:286:ARG:CZ	1:D:494:LEU:HD11	2.38	0.54
1:D:504:ASN:HB2	5:D:1244:HOH:O	2.07	0.54
1:C:391:LEU:HB2	1:C:423:ILE:HG21	1.89	0.54
1:A:376:LEU:HG	1:A:386:PHE:CZ	2.43	0.54
5:C:917:HOH:O	1:D:37:GLN:HG3	2.06	0.53
1:C:427:ARG:CB	1:C:427:ARG:CZ	2.85	0.53
1:C:250:VAL:CG1	5:C:888:HOH:O	2.40	0.53
1:C:342:LYS:O	1:C:342:LYS:HD3	2.09	0.53
1:C:367:ILE:HG23	1:C:367:ILE:O	2.08	0.53
1:B:9:ARG:HH22	1:B:186:GLN:HE22	1.57	0.53
1:C:332:TRP:CD2	1:C:371:ILE:CD1	2.92	0.53
1:B:251:ASN:OD1	1:B:254:ARG:NH2	2.42	0.53
1:C:339:LEU:CD2	1:C:361:MET:HB3	2.39	0.53
1:C:338:GLY:HA3	1:C:362:LYS:CB	2.39	0.52
1:C:365:GLU:OE2	1:C:365:GLU:HA	2.09	0.52
2:D:601:NAP:H6N	2:D:601:NAP:C3D	2.40	0.52
1:C:77:ASP:OD1	1:C:119:LYS:HE2	2.10	0.52
1:A:77:ASP:OD1	1:D:119:LYS:NZ	2.37	0.52
1:C:362:LYS:CB	1:C:362:LYS:NZ	2.72	0.52
1:B:315:MET:SD	1:B:475:ASP:HB3	2.50	0.52
1:C:372:LYS:HD3	1:C:397:PHE:CD1	2.44	0.52
1:C:399:GLU:HG3	1:C:400:ARG:HG2	1.92	0.52
1:C:324:LYS:HA	1:C:327:ALA:HB3	1.92	0.52
1:C:458:PHE:CD1	1:C:459:PRO:HD3	2.45	0.52
1:C:427:ARG:NH1	1:C:427:ARG:CB	2.73	0.51
1:C:348:THR:HG21	5:C:991:HOH:O	2.10	0.51

Continued on next page...

For Manuscript Review

Continued from previous page...

Atom-1	Atom-2	Interatomic distance (Å)	Clash overlap (Å)
1:C:567:GLN:HB2	5:C:768:HOH:O	2.10	0.51
1:A:566:ILE:HD11	1:B:31:THR:HG22	1.90	0.51
1:C:157:LEU:HD23	4:C:602:2OP:HA	1.92	0.51
1:C:369:GLN:HA	1:C:397:PHE:HZ	1.76	0.51
1:D:37:GLN:NE2	5:D:1011:HOH:O	2.44	0.51
1:A:46:PRO:HB3	1:D:126:THR:HG21	1.92	0.51
1:A:119:LYS:HB2	5:A:1034:HOH:O	2.10	0.51
1:A:169:ILE:HB	1:A:170:PRO:HD3	1.91	0.51
1:C:349:GLN:O	1:C:353:LYS:HD3	2.09	0.51
1:D:348:THR:HG22	1:D:351:LYS:HB2	1.92	0.51
1:C:339:LEU:HD22	1:C:361:MET:HG2	1.92	0.51
1:A:472:GLN:HG2	5:A:1019:HOH:O	2.10	0.51
1:C:334:VAL:HG22	1:C:339:LEU:HD12	1.93	0.51
1:A:177:TYR:HE1	1:A:462:ALA:HB2	1.76	0.51
1:B:530:TYR:HA	1:B:531:PRO:C	2.36	0.51
1:C:341:VAL:HG11	1:C:360:GLU:H	1.77	0.50
1:B:298:LEU:HB3	1:B:376:LEU:HD12	1.92	0.50
1:C:15:GLY:HA3	1:D:12:HIS:HE1	1.76	0.50
1:C:329:LYS:HG2	1:C:356:HIS:HD2	1.77	0.50
1:C:427:ARG:CG	1:C:427:ARG:NH1	2.72	0.50
1:D:340:ILE:HD11	1:D:351:LYS:HD3	1.93	0.50
1:B:403:ILE:O	1:B:430:PHE:HA	2.12	0.50
1:C:302:ALA:HB2	1:C:333:LEU:HB3	1.93	0.50
1:A:167:MET:HE2	1:A:192:ILE:HG22	1.94	0.49
1:C:349:GLN:HA	1:C:352:GLU:HG3	1.93	0.49
1:D:365:GLU:HG3	5:D:1239:HOH:O	2.11	0.49
1:D:138:ASN:ND2	5:D:1010:HOH:O	2.44	0.49
1:C:443:ASN:H	1:C:443:ASN:ND2	2.10	0.49
1:A:10:HIS:HD2	1:A:553:LEU:O	1.96	0.49
5:B:820:HOH:O	1:D:10:HIS:HD2	1.93	0.49
1:B:118:ARG:HB2	1:B:119:LYS:HD3	1.95	0.49
1:C:379:VAL:HG13	1:C:379:VAL:O	2.11	0.48
1:A:118:ARG:NH1	5:A:715:HOH:O	2.45	0.48
1:C:208:TYR:CZ	1:C:210:GLY:HA3	2.49	0.48
1:A:98:MET:N	1:A:99:PRO:HD2	2.29	0.48
1:C:297:ILE:HD12	1:C:375:ALA:HB3	1.96	0.48
1:D:110:CYS:O	1:D:165:ASN:HB3	2.13	0.48
1:C:297:ILE:HG21	1:C:377:ILE:CD1	2.43	0.48
1:B:331:ILE:CA	5:B:716:HOH:O	2.26	0.48
1:A:230:GLU:HB3	5:A:1041:HOH:O	2.14	0.48
1:C:329:LYS:HG2	1:C:356:HIS:CD2	2.49	0.48

Continued on next page...

For Manuscript Review

Continued from previous page...

Atom-1	Atom-2	Interatomic distance (Å)	Clash overlap (Å)
1:C:426:GLY:HA3	1:C:441:LEU:CD1	2.43	0.48
1:B:298:LEU:HB3	1:B:376:LEU:CD1	2.43	0.47
1:D:404:PHE:CD1	1:D:431:ALA:HB3	2.49	0.47
1:D:344:ARG:O	1:D:347:LEU:HG	2.14	0.47
1:A:322:LEU:HD21	1:A:330:LYS:CE	2.45	0.47
1:C:12:HIS:HD2	5:D:1131:HOH:O	1.96	0.47
1:A:116:VAL:O	1:A:118:ARG:HG3	2.14	0.47
1:C:375:ALA:HB2	1:C:402:ILE:HB	1.97	0.47
1:D:325:GLU:O	1:D:328:ILE:HG22	2.15	0.46
1:B:332:TRP:N	5:B:716:HOH:O	2.47	0.46
1:C:110:CYS:O	1:C:165:ASN:HB3	2.16	0.46
1:A:358:HIS:HD2	1:A:359:GLU:O	1.98	0.46
1:B:381:ALA:HA	1:B:407:SER:HB3	1.98	0.46
1:C:309:ILE:HG22	1:C:313:ILE:HD11	1.97	0.46
1:B:8:ARG:HH21	1:B:10:HIS:HE1	1.63	0.46
1:D:96:LYS:HD3	1:D:97:PHE:CZ	2.50	0.46
1:D:293:SER:O	1:D:330:LYS:NZ	2.49	0.46
1:D:441:LEU:HB2	1:D:442:PRO:CD	2.45	0.46
1:B:77:ASP:OD1	1:B:119:LYS:NZ	2.23	0.46
1:A:96:LYS:HE2	5:A:709:HOH:O	2.17	0.46
1:A:242:ILE:O	1:A:265:THR:HA	2.15	0.46
1:C:567:GLN:CB	5:C:768:HOH:O	2.63	0.45
1:D:419:GLN:HB2	5:D:1218:HOH:O	2.16	0.45
1:C:388:GLU:OE2	1:C:419:GLN:OE1	2.35	0.45
1:C:436:PHE:H	1:C:451:GLN:NE2	2.14	0.45
1:C:369:GLN:HA	1:C:397:PHE:CE2	2.52	0.45
1:C:126:THR:HB	1:C:211:LEU:HD11	1.97	0.45
1:C:299:PHE:CE1	1:C:377:ILE:HG21	2.52	0.45
1:B:13:GLN:NE2	5:B:703:HOH:O	2.36	0.45
1:C:304:GLU:HG3	2:C:601:NAP:H72N	1.81	0.45
1:C:358:HIS:HB3	1:C:359:GLU:H	1.65	0.45
1:B:156:ILE:HG23	1:B:169:ILE:HG13	1.98	0.45
1:B:432:SER:O	1:B:451:GLN:HA	2.16	0.45
1:C:325:GLU:HG2	1:C:329:LYS:HE3	1.99	0.45
1:C:372:LYS:HG2	1:C:397:PHE:CZ	2.52	0.45
1:C:277:ALA:CB	1:C:309:ILE:CD1	2.94	0.45
1:C:339:LEU:HD23	1:C:361:MET:HB3	1.97	0.45
1:C:342:LYS:HD3	1:C:342:LYS:C	2.42	0.45
1:B:119:LYS:HB2	5:B:788:HOH:O	2.15	0.45
1:D:126:THR:HB	1:D:211:LEU:HD11	1.99	0.45
1:D:541:ARG:HG3	5:D:1074:HOH:O	2.17	0.45

Continued on next page...

For Manuscript Review

Continued from previous page...

Atom-1	Atom-2	Interatomic distance (Å)	Clash overlap (Å)
1:A:12:HIS:HD2	5:B:959:HOH:O	2.00	0.45
1:C:372:LYS:HG2	1:C:397:PHE:CE2	2.52	0.45
1:D:530:TYR:HA	1:D:531:PRO:C	2.41	0.45
1:C:295:GLN:HB3	1:C:297:ILE:HD11	1.99	0.44
1:D:342:LYS:HA	1:D:342:LYS:HD3	1.63	0.44
1:B:9:ARG:HG2	1:B:552:ILE:HG22	2.00	0.44
1:C:51:GLN:O	1:C:55:VAL:HG23	2.17	0.44
1:C:367:ILE:HA	1:C:367:ILE:HD12	1.42	0.44
1:A:458:PHE:N	1:A:459:PRO:CD	2.80	0.44
1:B:291:LYS:HB2	1:B:320:GLU:OE2	2.17	0.44
1:C:388:GLU:N	1:C:391:LEU:HD23	2.33	0.44
1:A:378:GLY:HA3	1:A:414:GLU:HG2	1.99	0.44
1:C:331:ILE:HD13	1:C:354:PHE:CE1	2.44	0.44
1:C:333:LEU:CB	1:C:340:ILE:HG13	2.48	0.44
1:C:443:ASN:N	1:C:443:ASN:ND2	2.66	0.44
1:A:18:LEU:C	1:A:18:LEU:HD23	2.43	0.44
1:A:296:THR:HB	1:A:373:PRO:HA	1.99	0.44
1:A:322:LEU:HD21	1:A:330:LYS:HE3	2.00	0.44
1:C:388:GLU:CD	1:C:419:GLN:OE1	2.61	0.44
1:D:399:GLU:HG3	5:D:1268:HOH:O	2.17	0.43
1:A:8:ARG:NH1	1:A:555:ASP:OD1	2.52	0.43
1:C:51:GLN:NE2	1:C:88:ARG:HE	2.16	0.43
2:C:601:NAP:H5N	5:C:942:HOH:O	2.17	0.43
1:C:52:GLU:OE1	1:C:88:ARG:NH2	2.49	0.43
1:C:278:VAL:HG21	1:C:312:LEU:HB3	2.00	0.43
1:A:518:VAL:HG12	1:A:536:LYS:HG3	2.00	0.43
1:C:295:GLN:HB3	1:C:374:THR:HG21	1.99	0.43
1:A:410:THR:HG23	1:A:411:SER:N	2.33	0.43
1:C:458:PHE:CG	1:C:459:PRO:HD3	2.54	0.43
1:D:33:GLU:O	1:D:37:GLN:HG2	2.18	0.43
1:A:244:PHE:HD2	1:A:247:PHE:CE1	2.37	0.42
1:D:493:HIS:CD2	1:D:498:ARG:HD2	2.54	0.42
1:C:404:PHE:CE1	1:C:431:ALA:HB2	2.54	0.42
1:C:435:PRO:HA	1:C:451:GLN:NE2	2.21	0.42
1:D:167:MET:O	1:D:170:PRO:HD2	2.20	0.42
1:D:378:GLY:C	1:D:414:GLU:OE2	2.63	0.42
1:C:118:ARG:CD	5:B:812:HOH:O	2.58	0.42
1:C:268:ASP:CG	1:C:454:ASN:HD22	2.28	0.42
1:C:278:VAL:HG21	1:C:312:LEU:CB	2.50	0.42
1:B:185:PRO:HD2	1:B:545:TYR:CD1	2.54	0.42
1:C:108:LEU:HA	1:C:111:GLN:HE21	1.84	0.42

Continued on next page...

For Manuscript Review

Continued from previous page...

Atom-1	Atom-2	Interatomic distance (Å)	Clash overlap (Å)
1:C:279:ALA:HB2	1:C:482:ALA:HA	2.02	0.42
1:C:332:TRP:CG	1:C:371:ILE:HD11	2.49	0.42
1:C:391:LEU:HB2	1:C:423:ILE:CG2	2.50	0.42
1:B:7:ARG:NH1	5:B:711:HOH:O	2.41	0.42
1:B:137:LEU:HD21	1:B:232:VAL:HG23	2.00	0.42
1:C:365:GLU:HB3	1:C:366:ALA:H	1.69	0.42
1:B:563:VAL:HG21	1:D:128:HIS:CD2	2.55	0.42
1:C:260:ARG:O	1:C:472:GLN:HG3	2.20	0.42
1:B:214:ARG:HG3	5:B:944:HOH:O	2.20	0.42
1:B:341:VAL:O	1:B:344:ARG:HB2	2.19	0.42
1:B:359:GLU:H	1:B:359:GLU:HG3	1.52	0.42
1:A:148:ILE:HG12	1:A:189:LEU:HB3	2.01	0.42
1:A:530:TYR:HA	1:A:531:PRO:C	2.45	0.42
1:C:494:LEU:HD23	1:C:494:LEU:HA	1.86	0.42
1:B:315:MET:HE2	1:B:315:MET:HB3	1.88	0.42
1:B:386:PHE:CG	1:B:414:GLU:HB3	2.55	0.42
1:B:297:ILE:HA	1:B:375:ALA:O	2.20	0.41
1:B:559:TRP:H	1:B:564:GLN:HE21	1.66	0.41
1:B:17:LEU:HD23	1:B:17:LEU:HA	1.89	0.41
1:A:133:ILE:HD11	1:A:224:PHE:CE1	2.54	0.41
1:C:344:ARG:HB3	1:C:347:LEU:HD22	2.03	0.41
1:D:369:GLN:HA	1:D:397:PHE:CZ	2.56	0.41
1:A:251:ASN:OD1	1:A:254:ARG:NH2	2.38	0.41
1:C:380:ALA:HB1	2:C:601:NAP:C4A	2.51	0.41
1:D:273:THR:HG21	2:D:601:NAP:C5N	2.50	0.41
1:A:315:MET:HE2	1:A:315:MET:HB3	1.89	0.41
1:D:333:LEU:HB2	1:D:340:ILE:HD12	2.03	0.41
1:B:337:LYS:HE3	1:B:346:SER:OG	2.20	0.41
1:B:391:LEU:HD12	1:B:423:ILE:CD1	2.48	0.41
1:D:298:LEU:HD23	1:D:376:LEU:HD13	2.03	0.41
1:B:305:ALA:O	1:B:309:ILE:HG13	2.21	0.41
1:B:458:PHE:N	1:B:459:PRO:CD	2.83	0.41
1:D:329:LYS:HE3	5:D:1250:HOH:O	2.19	0.41
1:C:5:ALA:N	5:C:733:HOH:O	2.54	0.41
1:A:378:GLY:C	1:A:414:GLU:OE2	2.64	0.41
1:A:440:THR:HG22	1:A:446:THR:OG1	2.20	0.41
5:A:863:HOH:O	1:B:568:THR:CA	2.51	0.41
1:C:508:ASP:HB2	5:C:797:HOH:O	2.21	0.41
1:B:9:ARG:NH1	1:B:550:ASP:OD2	2.54	0.41
1:D:119:LYS:H	1:D:119:LYS:HG2	1.76	0.41
1:C:299:PHE:CZ	1:C:377:ILE:HD13	2.55	0.41

Continued on next page...

For Manuscript Review

Continued from previous page...

Atom-1	Atom-2	Interatomic distance (Å)	Clash overlap (Å)
1:B:300:GLN:HB3	1:B:378:GLY:HA2	2.03	0.41
1:D:322:LEU:HD12	1:D:322:LEU:HA	1.95	0.41
1:D:424:THR:HG21	1:D:428:ALA:HB2	2.01	0.41
1:C:279:ALA:HB2	1:C:482:ALA:O	2.20	0.40
1:C:297:ILE:HG23	1:C:377:ILE:HD12	1.96	0.40
1:C:347:LEU:HA	1:C:347:LEU:HD12	1.85	0.40
1:C:367:ILE:O	1:C:367:ILE:CG2	2.68	0.40
1:D:12:HIS:CG	5:D:1113:HOH:O	2.74	0.40
1:D:348:THR:HG23	1:D:351:LYS:H	1.86	0.40
1:A:14:ARG:HD2	1:A:38:LEU:HA	2.04	0.40
1:A:300:GLN:HE21	1:A:300:GLN:HA	1.86	0.40
1:A:533:PRO:HG3	1:A:539:PHE:CD2	2.56	0.40
1:C:362:LYS:CG	1:C:362:LYS:O	2.69	0.40
1:D:6:PRO:N	5:D:1034:HOH:O	2.55	0.40
1:A:300:GLN:HB3	1:A:378:GLY:HA2	2.02	0.40
1:C:119:LYS:HE3	1:B:77:ASP:OD1	2.22	0.40
4:C:602:2OP:HB3	5:C:942:HOH:O	2.22	0.40
1:B:341:VAL:CG1	1:B:358:HIS:HB3	2.51	0.40
1:B:376:LEU:HG	1:B:386:PHE:CZ	2.55	0.40
1:C:342:LYS:C	1:C:342:LYS:CD	2.93	0.40
1:D:541:ARG:CG	5:D:1074:HOH:O	2.69	0.40

There are no symmetry-related clashes.

5.3 Torsion angles (i)

5.3.1 Protein backbone (i)

In the following table, the Percentiles column shows the percent Ramachandran outliers of the chain as a percentile score with respect to all X-ray entries followed by that with respect to entries of similar resolution.

The Analysed column shows the number of residues for which the backbone conformation was analysed, and the total number of residues.

Mol	Chain	Analysed	Favoured	Allowed	Outliers	Percentiles	
1	A	562/572 (98%)	548 (98%)	14 (2%)	0	100	100
1	B	561/572 (98%)	545 (97%)	14 (2%)	2 (0%)	30	26
1	C	561/572 (98%)	524 (93%)	31 (6%)	6 (1%)	12	6
1	D	562/572 (98%)	544 (97%)	16 (3%)	2 (0%)	30	26

Continued on next page...

For Manuscript Review

Continued from previous page...

Mol	Chain	Analysed	Favoured	Allowed	Outliers	Percentiles	
All	All	2246/2288 (98%)	2161 (96%)	75 (3%)	10 (0%)	30	26

All (10) Ramachandran outliers are listed below:

Mol	Chain	Res	Type
1	C	362	LYS
1	C	364	LEU
1	C	387	SER
1	C	356	HIS
1	C	410	THR
1	B	7	ARG
1	B	345	ALA
1	D	342	LYS
1	D	7	ARG
1	C	301	GLY

5.3.2 Protein sidechains (i)

In the following table, the Percentiles column shows the percent sidechain outliers of the chain as a percentile score with respect to all X-ray entries followed by that with respect to entries of similar resolution.

The Analysed column shows the number of residues for which the sidechain conformation was analysed, and the total number of residues.

Mol	Chain	Analysed	Rotameric	Outliers	Percentiles	
1	A	482/490 (98%)	467 (97%)	15 (3%)	35	34
1	B	482/490 (98%)	459 (95%)	23 (5%)	21	18
1	C	481/490 (98%)	433 (90%)	48 (10%)	6	3
1	D	483/490 (99%)	460 (95%)	23 (5%)	21	18
All	All	1928/1960 (98%)	1819 (94%)	109 (6%)	17	13

All (109) residues with a non-rotameric sidechain are listed below:

Mol	Chain	Res	Type
1	A	63	GLU
1	A	232	VAL
1	A	243	GLN
1	A	289	LYS
1	A	300	GLN

Continued on next page...

For Manuscript Review

Continued from previous page...

Mol	Chain	Res	Type
1	A	314	VAL
1	A	334	VAL
1	A	344	ARG
1	A	353	LYS
1	A	357	GLU
1	A	427	ARG
1	A	451	GLN
1	A	476	ASN
1	A	519	LYS
1	A	534	GLN
1	C	7	ARG
1	C	9	ARG
1	C	81	ARG
1	C	119	LYS
1	C	137	LEU
1	C	232	VAL
1	C	243	GLN
1	C	276	VAL
1	C	289	LYS
1	C	312	LEU
1	C	324	LYS
1	C	326	LYS
1	C	328	ILE
1	C	330	LYS
1	C	331	ILE
1	C	340	ILE
1	C	342	LYS
1	C	346	SER
1	C	347	LEU
1	C	357	GLU
1	C	358	HIS
1	C	362	LYS
1	C	365	GLU
1	C	367	ILE
1	C	368	VAL
1	C	371	ILE
1	C	382	ILE
1	C	388	GLU
1	C	389	GLN
1	C	390	ILE
1	C	391	LEU
1	C	408	ASN

Continued on next page...

For Manuscript Review

Continued from previous page...

Mol	Chain	Res	Type
1	C	411	SER
1	C	418	GLU
1	C	419	GLN
1	C	422	LYS
1	C	423	ILE
1	C	427	ARG
1	C	443	ASN
1	C	445	GLN
1	C	453	ASN
1	C	470	LEU
1	C	512	LYS
1	C	519	LYS
1	C	523	GLN
1	C	558	SER
1	C	562	GLU
1	C	565	LYS
1	B	7	ARG
1	B	9	ARG
1	B	60	LYS
1	B	118	ARG
1	B	119	LYS
1	B	159	LEU
1	B	183	MET
1	B	232	VAL
1	B	234	SER
1	B	243	GLN
1	B	250	VAL
1	B	281	LEU
1	B	291	LYS
1	B	297	ILE
1	B	329	LYS
1	B	330	LYS
1	B	334	VAL
1	B	359	GLU
1	B	410	THR
1	B	418	GLU
1	B	440	THR
1	B	470	LEU
1	B	565	LYS
1	D	6	PRO
1	D	90	LEU
1	D	119	LYS

Continued on next page...

For Manuscript Review

Continued from previous page...

Mol	Chain	Res	Type
1	D	183	MET
1	D	220	GLU
1	D	232	VAL
1	D	243	GLN
1	D	250	VAL
1	D	281	LEU
1	D	324	LYS
1	D	325	GLU
1	D	342	LYS
1	D	350	GLU
1	D	353	LYS
1	D	357	GLU
1	D	379	VAL
1	D	440	THR
1	D	441	LEU
1	D	443	ASN
1	D	523	GLN
1	D	525	LYS
1	D	563	VAL
1	D	568	THR

Sometimes sidechains can be flipped to improve hydrogen bonding and reduce clashes. All (56) such sidechains are listed below:

Mol	Chain	Res	Type
1	A	10	HIS
1	A	12	HIS
1	A	61	ASN
1	A	66	ASN
1	A	186	GLN
1	A	262	GLN
1	A	300	GLN
1	A	389	GLN
1	A	445	GLN
1	A	487	GLN
1	C	12	HIS
1	C	39	ASN
1	C	51	GLN
1	C	111	GLN
1	C	186	GLN
1	C	249	ASN
1	C	251	ASN
1	C	262	GLN

Continued on next page...

For Manuscript Review

Continued from previous page...

Mol	Chain	Res	Type
1	C	290	ASN
1	C	389	GLN
1	C	408	ASN
1	C	419	GLN
1	C	443	ASN
1	C	451	GLN
1	C	454	ASN
1	C	523	GLN
1	C	535	ASN
1	C	567	GLN
1	B	12	HIS
1	B	49	ASN
1	B	66	ASN
1	B	186	GLN
1	B	213	GLN
1	B	239	ASN
1	B	262	GLN
1	B	300	GLN
1	B	358	HIS
1	B	369	GLN
1	B	389	GLN
1	B	419	GLN
1	B	443	ASN
1	B	488	GLN
1	B	564	GLN
1	D	12	HIS
1	D	39	ASN
1	D	61	ASN
1	D	66	ASN
1	D	79	GLN
1	D	111	GLN
1	D	186	GLN
1	D	249	ASN
1	D	363	ASN
1	D	389	GLN
1	D	451	GLN
1	D	472	GLN
1	D	488	GLN

5.3.3 RNA ⓘ

There are no RNA molecules in this entry.

For Manuscript Review

5.4 Non-standard residues in protein, DNA, RNA chains (i)

There are no non-standard protein/DNA/RNA residues in this entry.

5.5 Carbohydrates (i)

There are no oligosaccharides in this entry.

5.6 Ligand geometry (i)

Of 10 ligands modelled in this entry, 4 are monoatomic - leaving 6 for Mogul analysis.

In the following table, the Counts columns list the number of bonds (or angles) for which Mogul statistics could be retrieved, the number of bonds (or angles) that are observed in the model and the number of bonds (or angles) that are defined in the Chemical Component Dictionary. The Link column lists molecule types, if any, to which the group is linked. The Z score for a bond length (or angle) is the number of standard deviations the observed value is removed from the expected value. A bond length (or angle) with $|Z| > 2$ is considered an outlier worth inspection. RMSZ is the root-mean-square of all Z scores of the bond lengths (or angles).

Mol	Type	Chain	Res	Link	Bond lengths			Bond angles		
					Counts	RMSZ	$\#\|Z\| > 2$	Counts	RMSZ	$\#\|Z\| > 2$
4	2OP	D	602	3	5,5,5	1.23	1 (20%)	4,6,6	1.30	1 (25%)
2	NAP	B	601	-	45,52,52	0.95	3 (6%)	56,80,80	0.87	3 (5%)
2	NAP	A	601	-	45,52,52	0.97	3 (6%)	56,80,80	0.92	4 (7%)
2	NAP	D	601	-	45,52,52	1.05	2 (4%)	56,80,80	1.01	3 (5%)
2	NAP	C	601	-	45,52,52	0.71	0	56,80,80	1.28	6 (10%)
4	2OP	C	602	3	5,5,5	0.95	0	4,6,6	0.45	0

In the following table, the Chirals column lists the number of chiral outliers, the number of chiral centers analysed, the number of these observed in the model and the number defined in the Chemical Component Dictionary. Similar counts are reported in the Torsion and Rings columns. '-' means no outliers of that kind were identified.

Mol	Type	Chain	Res	Link	Chirals	Torsions	Rings
4	2OP	D	602	3	-	2/4/4/4	-
2	NAP	B	601	-	-	5/31/67/67	0/5/5/5
2	NAP	A	601	-	-	4/31/67/67	0/5/5/5
2	NAP	D	601	-	-	10/31/67/67	0/5/5/5
2	NAP	C	601	-	-	13/31/67/67	0/5/5/5
4	2OP	C	602	3	-	0/4/4/4	-

For Manuscript Review

All (9) bond length outliers are listed below:

Mol	Chain	Res	Type	Atoms	Z	Observed(Å)	Ideal(Å)
2	D	601	NAP	O4D-C1D	3.83	1.46	1.41
2	D	601	NAP	C2N-N1N	3.32	1.39	1.35
4	D	602	2OP	OXT-C	-2.60	1.22	1.30
2	A	601	NAP	P2B-O2X	-2.35	1.45	1.54
2	B	601	NAP	P2B-O2X	-2.28	1.46	1.54
2	B	601	NAP	P2B-O3X	-2.11	1.46	1.54
2	B	601	NAP	C8A-N7A	-2.05	1.31	1.34
2	A	601	NAP	P2B-O3X	-2.03	1.47	1.54
2	A	601	NAP	C8A-N7A	-2.02	1.31	1.34

All (17) bond angle outliers are listed below:

Mol	Chain	Res	Type	Atoms	Z	Observed(^{o})	Ideal(^{o})
2	C	601	NAP	C3N-C2N-N1N	-4.21	116.31	120.43
2	C	601	NAP	C6N-N1N-C2N	-3.90	118.42	121.97
2	D	601	NAP	C6N-N1N-C2N	-3.29	118.97	121.97
2	C	601	NAP	O4D-C1D-C2D	-3.11	102.38	106.93
2	C	601	NAP	C5A-C6A-N6A	2.75	124.53	120.35
2	C	601	NAP	C3B-C2B-C1B	-2.61	97.98	102.89
2	B	601	NAP	O2B-P2B-O1X	-2.53	99.62	109.39
2	D	601	NAP	C5A-C6A-N6A	2.52	124.18	120.35
2	A	601	NAP	C3B-C2B-C1B	-2.47	98.24	102.89
2	B	601	NAP	C5A-C6A-N6A	2.44	124.06	120.35
2	D	601	NAP	O3D-C3D-C2D	2.38	119.52	111.82
2	A	601	NAP	C6N-N1N-C2N	-2.21	119.96	121.97
2	B	601	NAP	C6N-N1N-C2N	-2.19	119.97	121.97
4	D	602	2OP	CB-CA-C	2.19	115.59	110.63
2	A	601	NAP	C5A-C6A-N6A	2.18	123.66	120.35
2	A	601	NAP	O2B-C2B-C3B	2.14	119.42	111.68
2	C	601	NAP	C2D-C3D-C4D	-2.06	98.64	102.64

There are no chirality outliers.

All (34) torsion outliers are listed below:

Mol	Chain	Res	Type	Atoms
2	A	601	NAP	O4D-C1D-N1N-C6N
2	C	601	NAP	C5B-O5B-PA-O2A
2	C	601	NAP	C2D-C1D-N1N-C2N
2	C	601	NAP	C2D-C1D-N1N-C6N
2	C	601	NAP	C2N-C3N-C7N-O7N
2	C	601	NAP	C2N-C3N-C7N-N7N

Continued on next page...

For Manuscript Review

Continued from previous page...

Mol	Chain	Res	Type	Atoms
2	B	601	NAP	C2B-O2B-P2B-O1X
2	D	601	NAP	C2B-O2B-P2B-O1X
2	D	601	NAP	O4D-C4D-C5D-O5D
2	D	601	NAP	O4D-C1D-N1N-C6N
2	C	601	NAP	C4N-C3N-C7N-N7N
2	C	601	NAP	C4N-C3N-C7N-O7N
2	A	601	NAP	C3B-C2B-O2B-P2B
2	C	601	NAP	C3B-C2B-O2B-P2B
2	D	601	NAP	C3D-C4D-C5D-O5D
2	B	601	NAP	C1B-C2B-O2B-P2B
2	D	601	NAP	C1B-C2B-O2B-P2B
2	B	601	NAP	C3B-C2B-O2B-P2B
2	D	601	NAP	C3B-C2B-O2B-P2B
2	A	601	NAP	C1B-C2B-O2B-P2B
2	C	601	NAP	C3D-C4D-C5D-O5D
2	C	601	NAP	O4D-C4D-C5D-O5D
2	B	601	NAP	C2B-O2B-P2B-O3X
2	D	601	NAP	PN-O3-PA-O1A
4	D	602	2OP	OXT-C-CA-CB
2	A	601	NAP	O4B-C4B-C5B-O5B
2	C	601	NAP	O4B-C4B-C5B-O5B
4	D	602	2OP	O-C-CA-CB
2	C	601	NAP	C5B-O5B-PA-O3
2	D	601	NAP	C2D-C1D-N1N-C2N
2	D	601	NAP	O4B-C4B-C5B-O5B
2	D	601	NAP	PN-O3-PA-O2A
2	C	601	NAP	C5B-O5B-PA-O1A
2	B	601	NAP	O4B-C4B-C5B-O5B

There are no ring outliers.

4 monomers are involved in 14 short contacts:

Mol	Chain	Res	Type	Clashes	Symm-Clashes
4	D	602	2OP	2	0
2	D	601	NAP	6	0
2	C	601	NAP	4	0
4	C	602	2OP	2	0

The following is a two-dimensional graphical depiction of Mogul quality analysis of bond lengths, bond angles, torsion angles, and ring geometry for all instances of the Ligand of Interest. In addition, ligands with molecular weight > 250 and outliers as shown on the validation Tables will also be included. For torsion angles, if less then 5% of the Mogul distribution of torsion angles is

For Manuscript Review

within 10 degrees of the torsion angle in question, then that torsion angle is considered an outlier. Any bond that is central to one or more torsion angles identified as an outlier by Mogul will be highlighted in the graph. For rings, the root-mean-square deviation (RMSD) between the ring in question and similar rings identified by Mogul is calculated over all ring torsion angles. If the average RMSD is greater than 60 degrees and the minimal RMSD between the ring in question and any Mogul-identified rings is also greater than 60 degrees, then that ring is considered an outlier. The outliers are highlighted in purple. The color gray indicates Mogul did not find sufficient equivalents in the CSD to analyse the geometry.

Ligand 2OP D 602

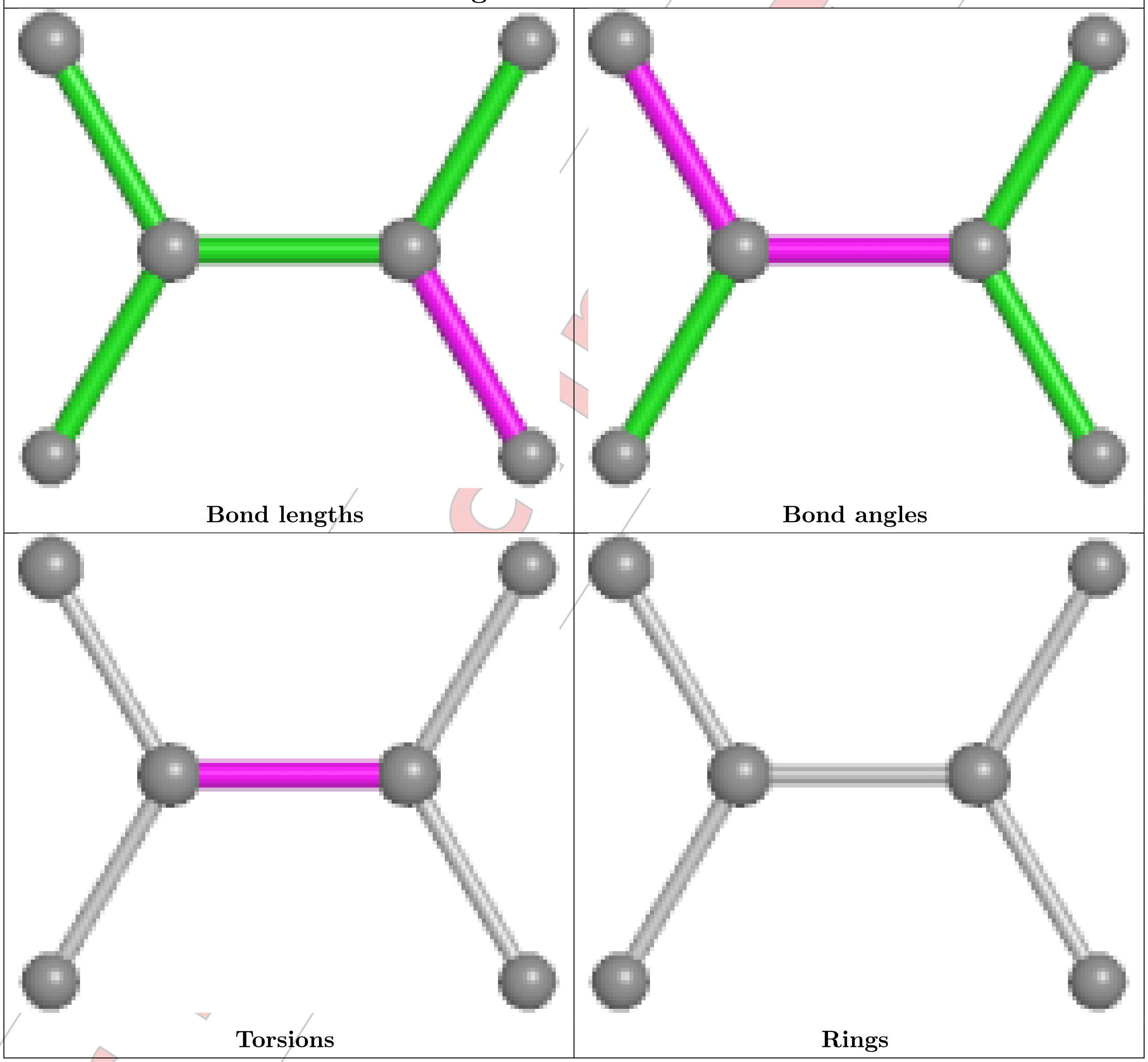

Ligand NAP B 601

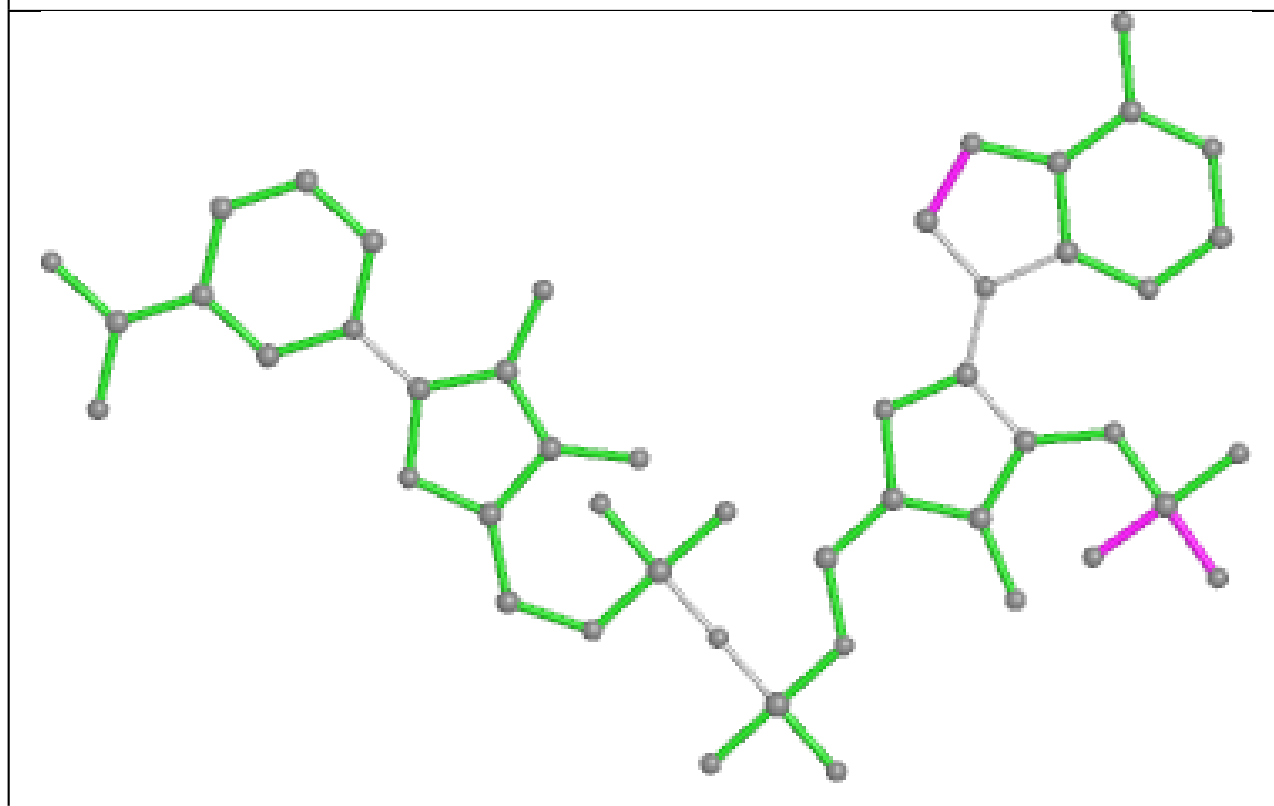

Bond lengths

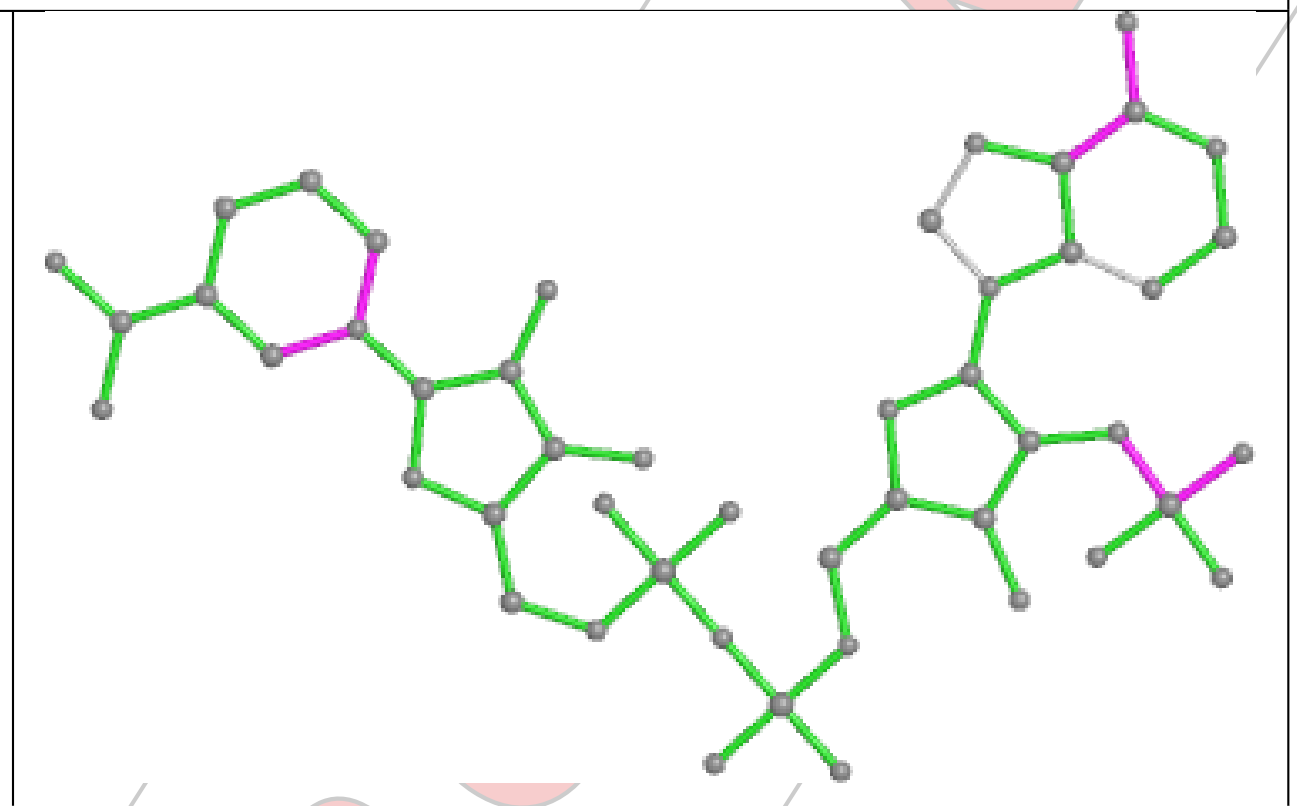

Bond angles

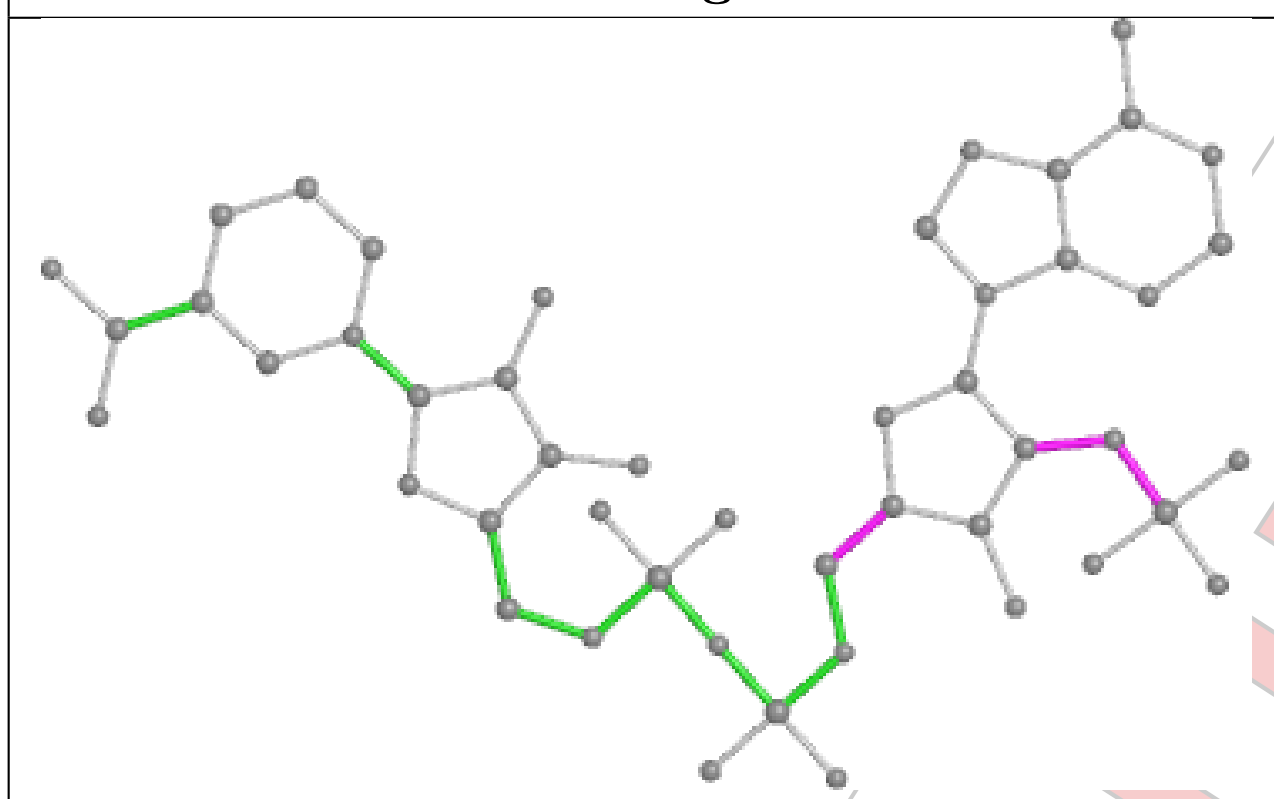

Torsions

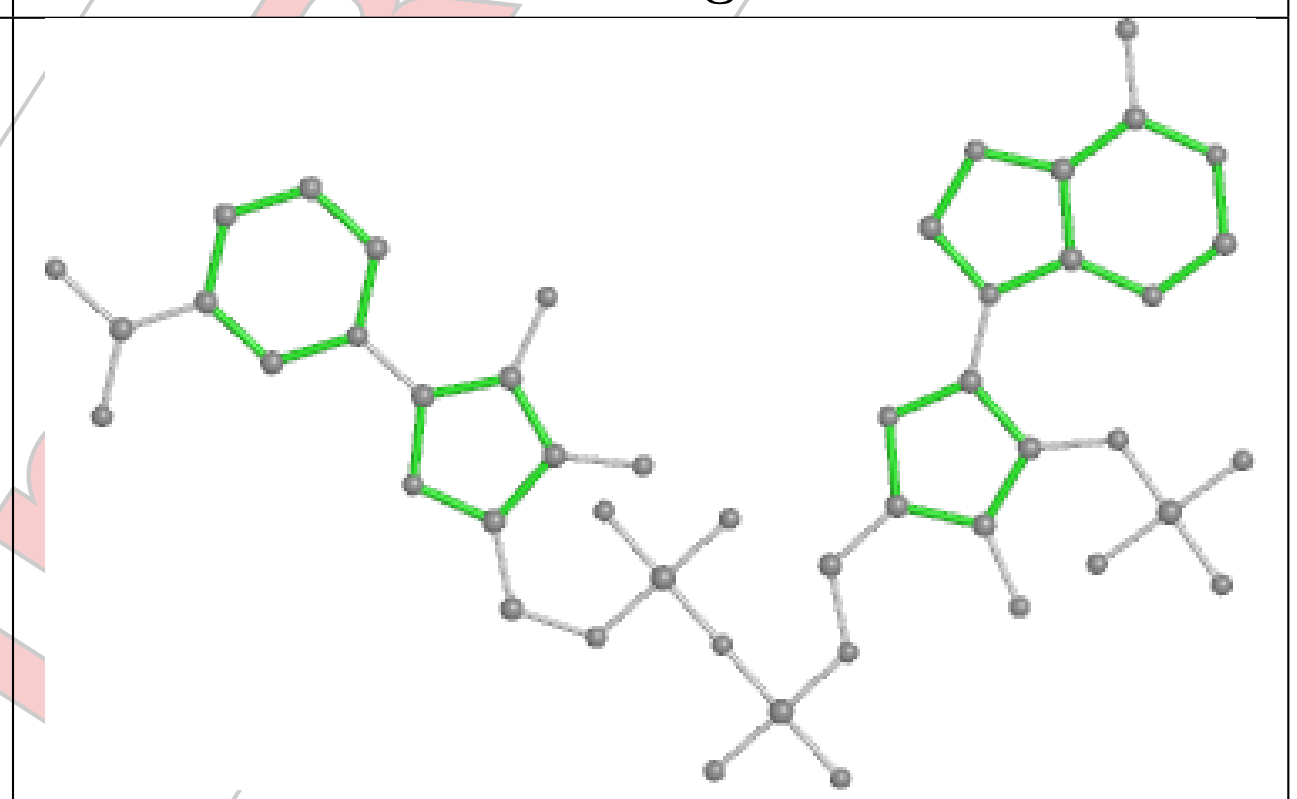

Rings

For Manuscript Review

Ligand NAP A 601

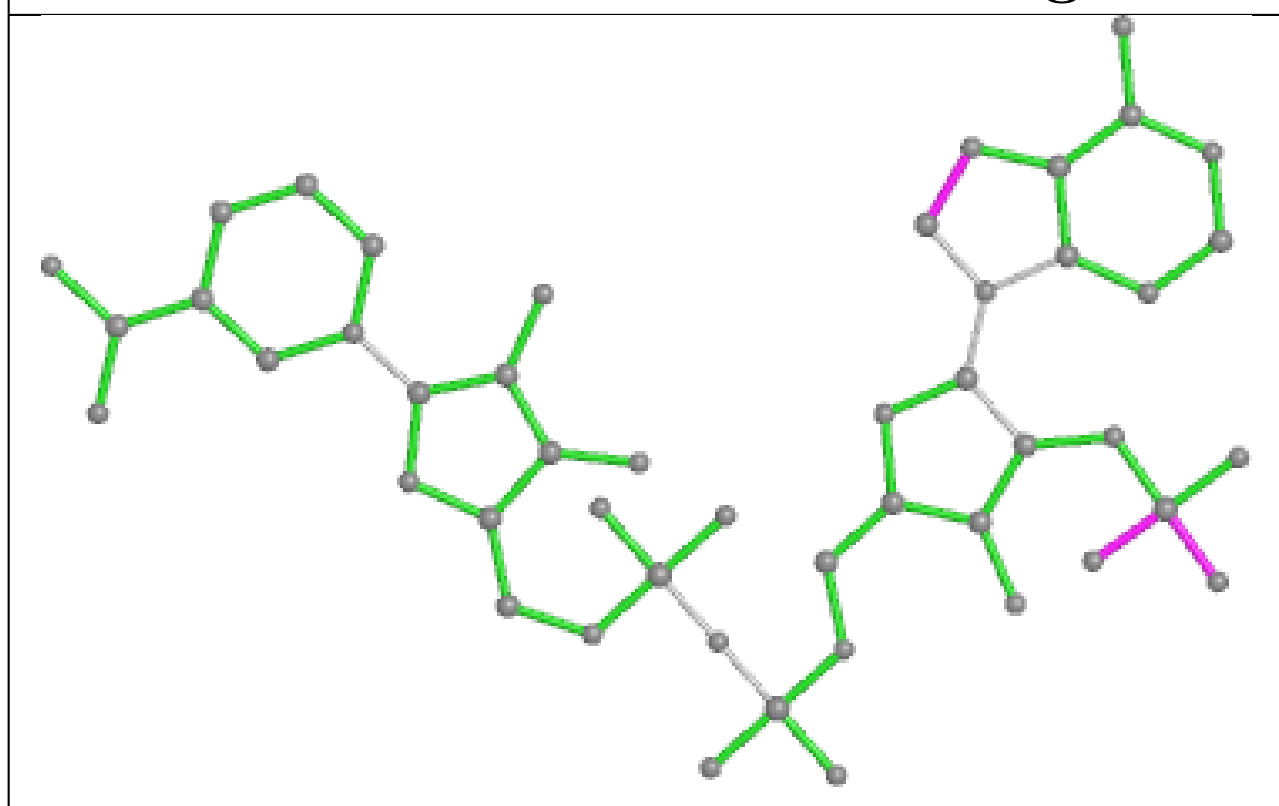

Bond lengths

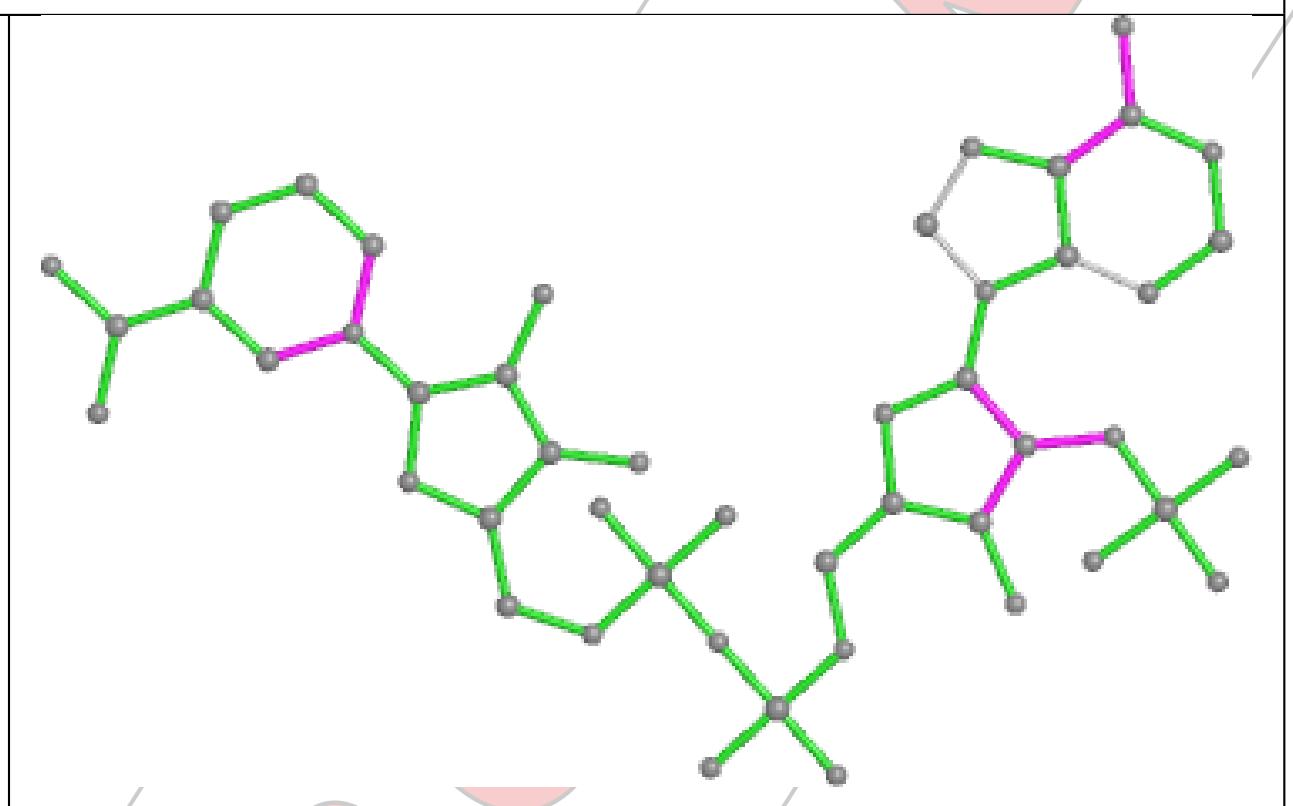

Bond angles

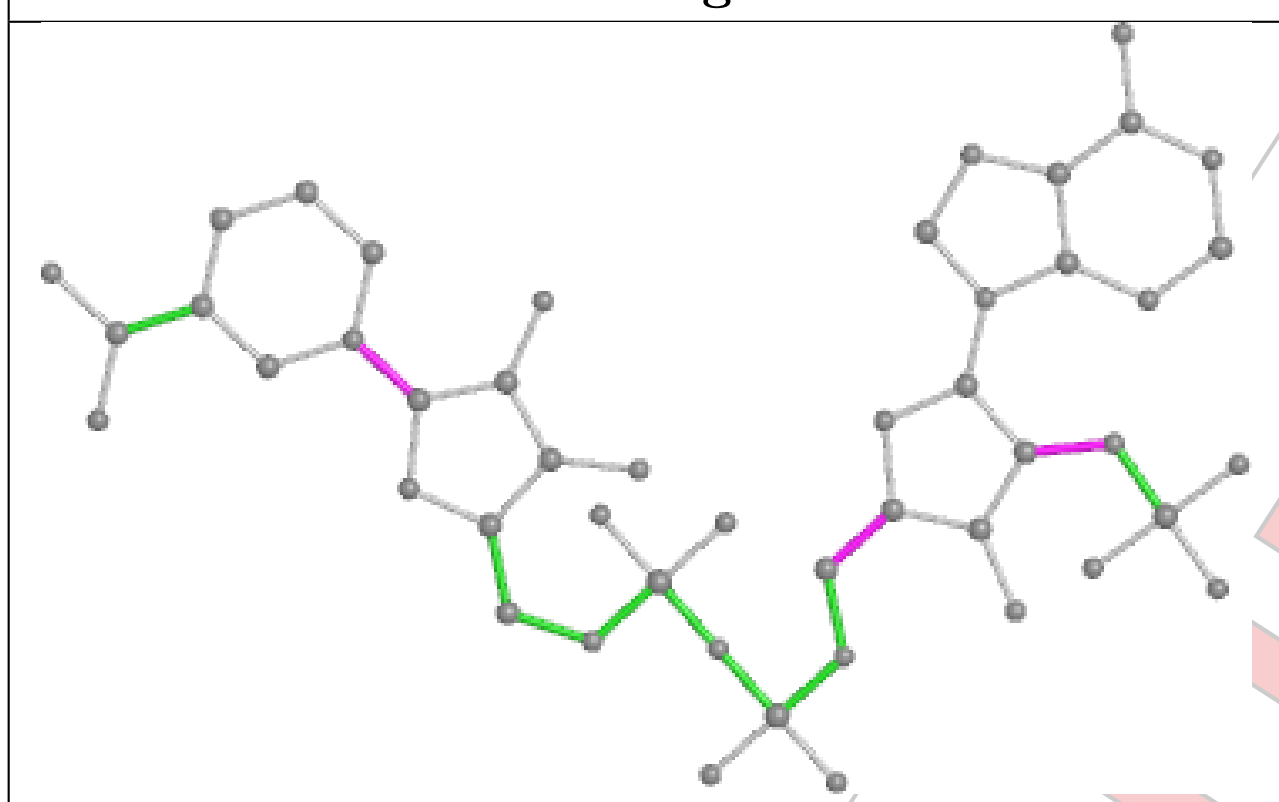

Torsions

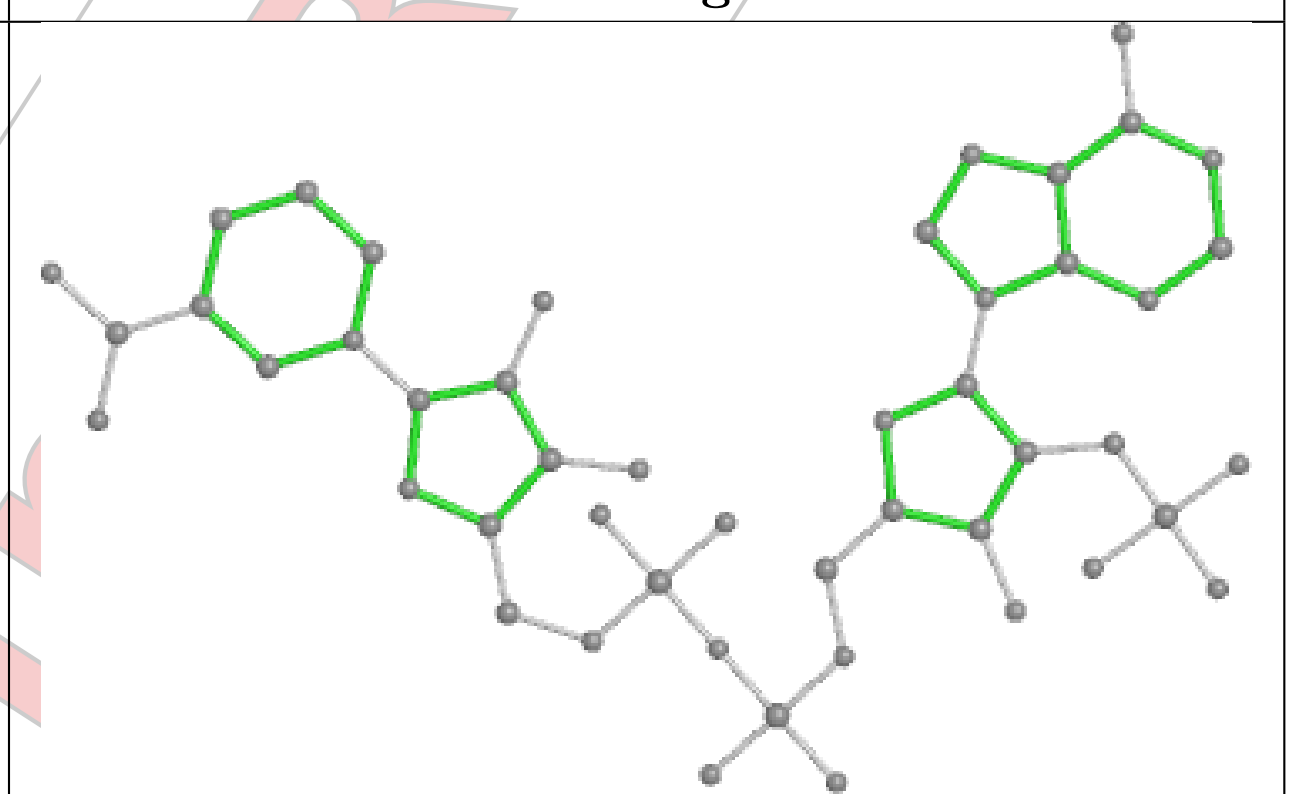

Rings

For Manuscript Review

Ligand NAP D 601

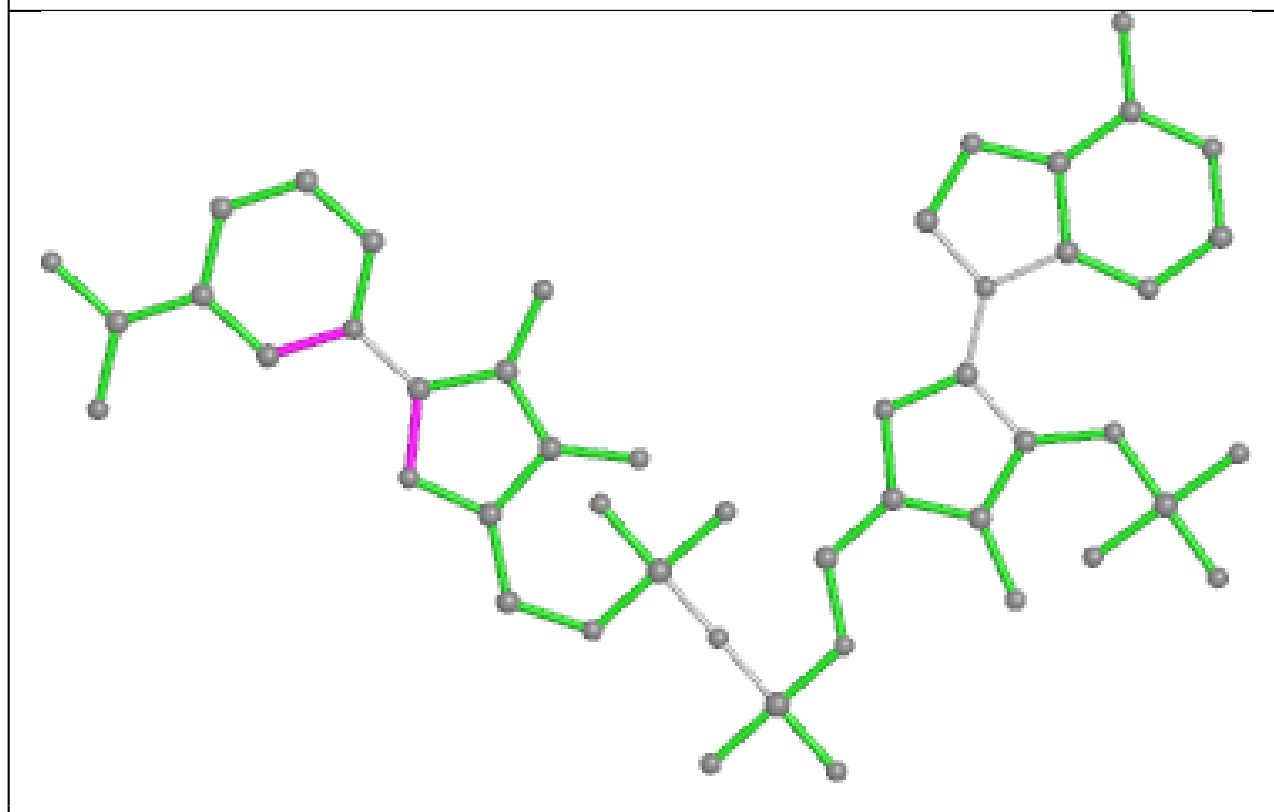

Bond lengths

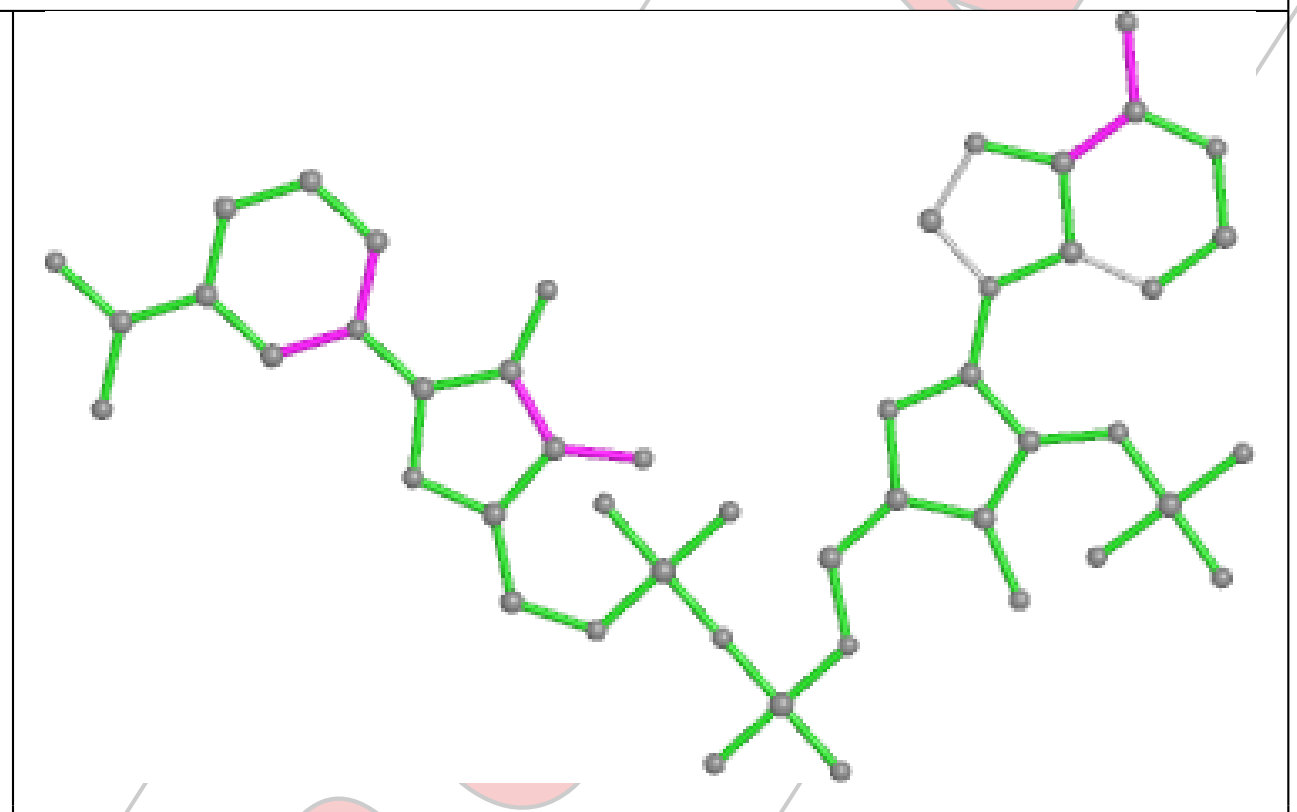

Bond angles

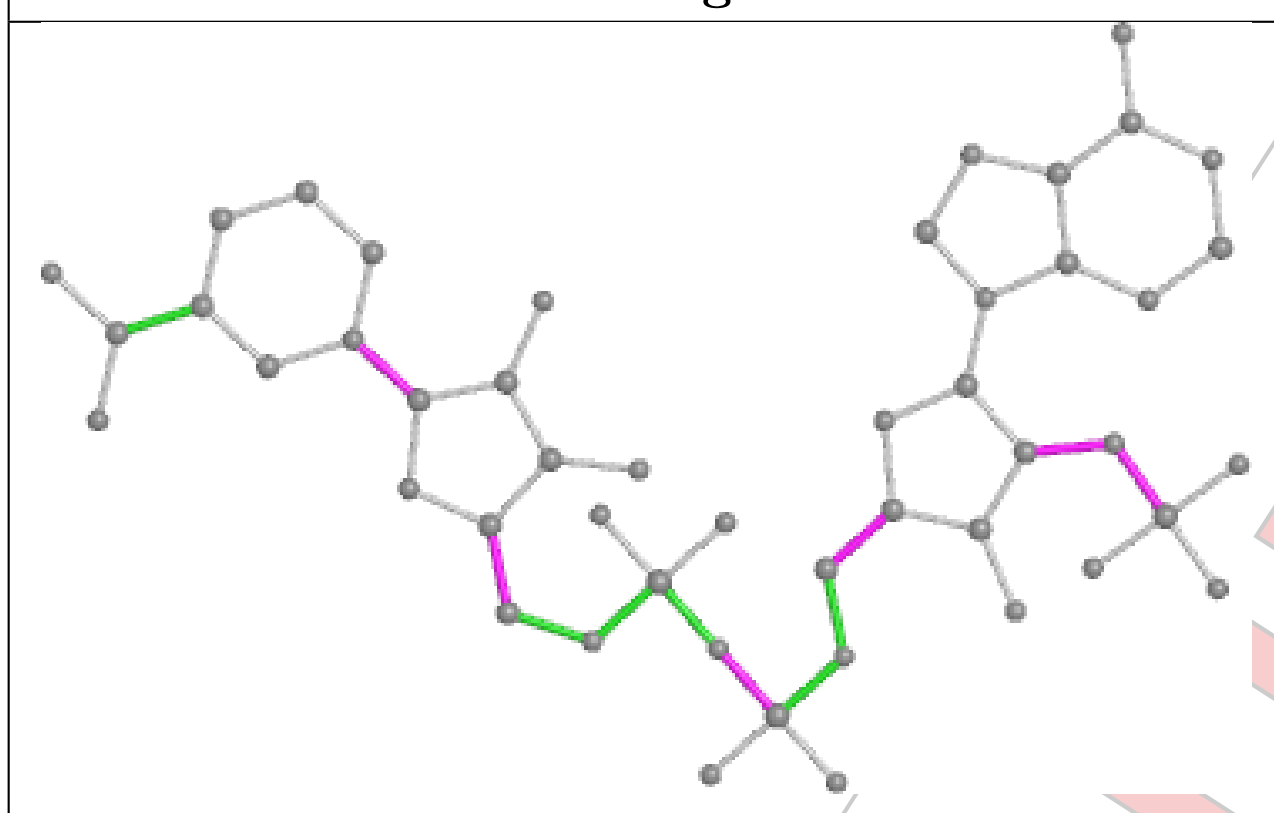

Torsions

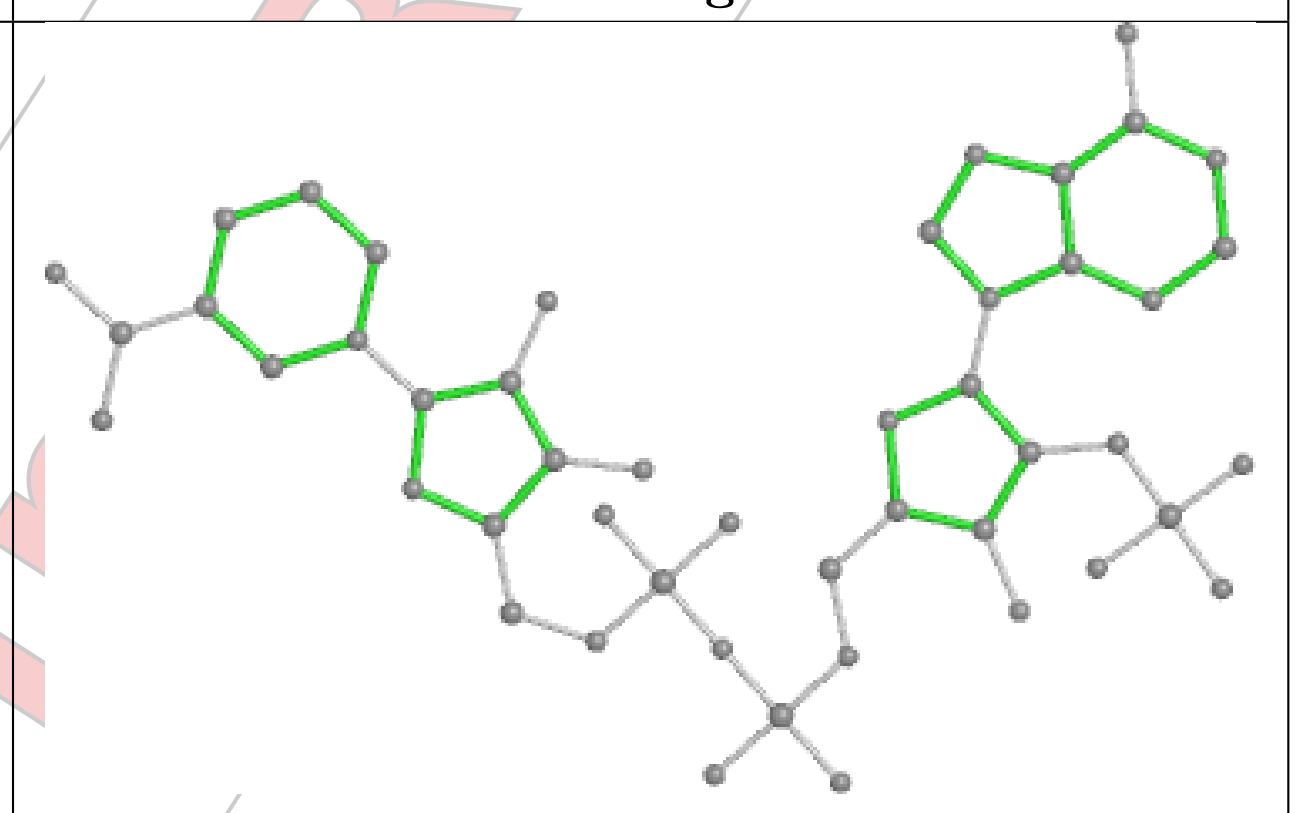

Rings

For Manuscript Review

Ligand NAP C 601

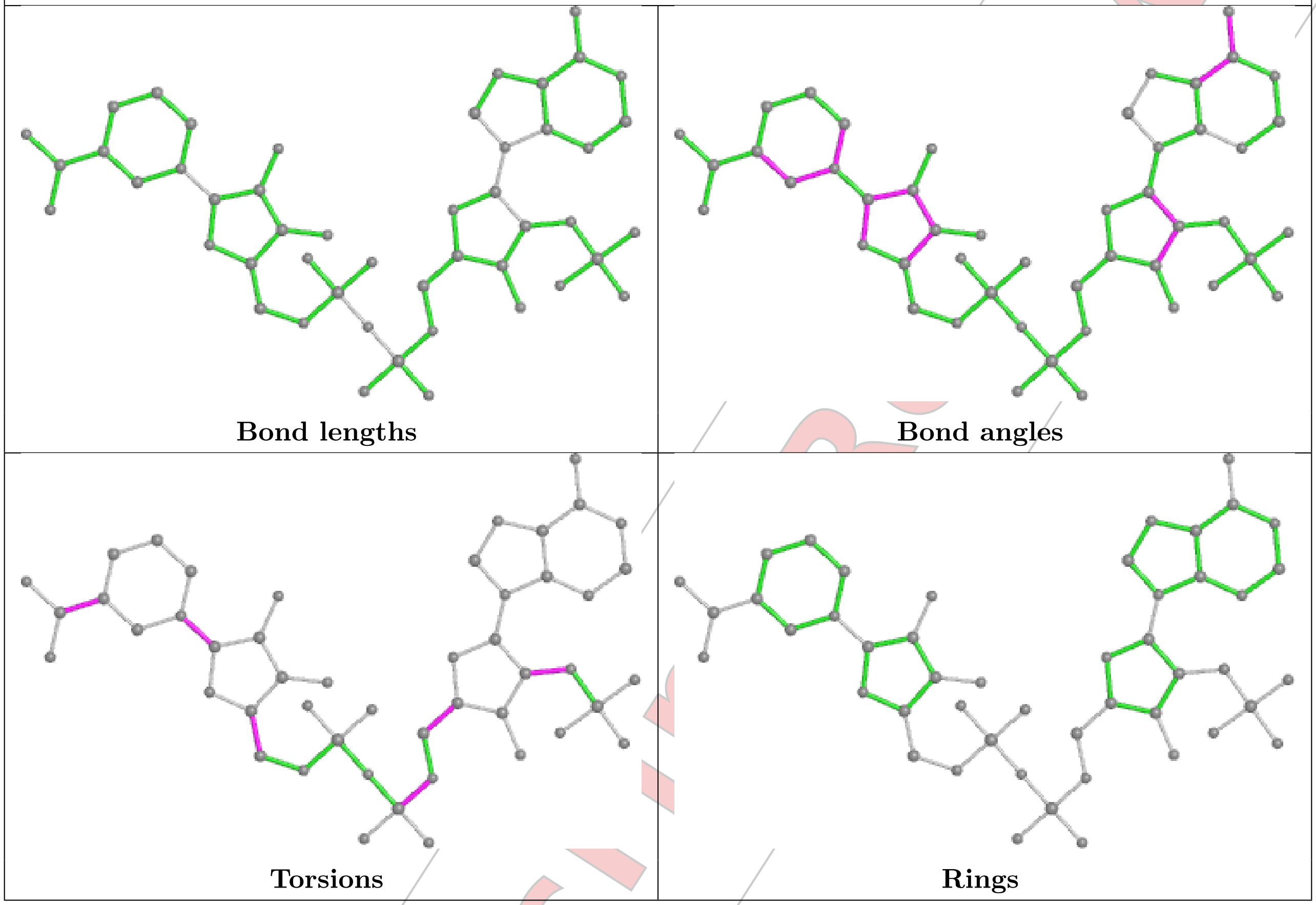

For Manuscript Review

Ligand 2OP C 602	
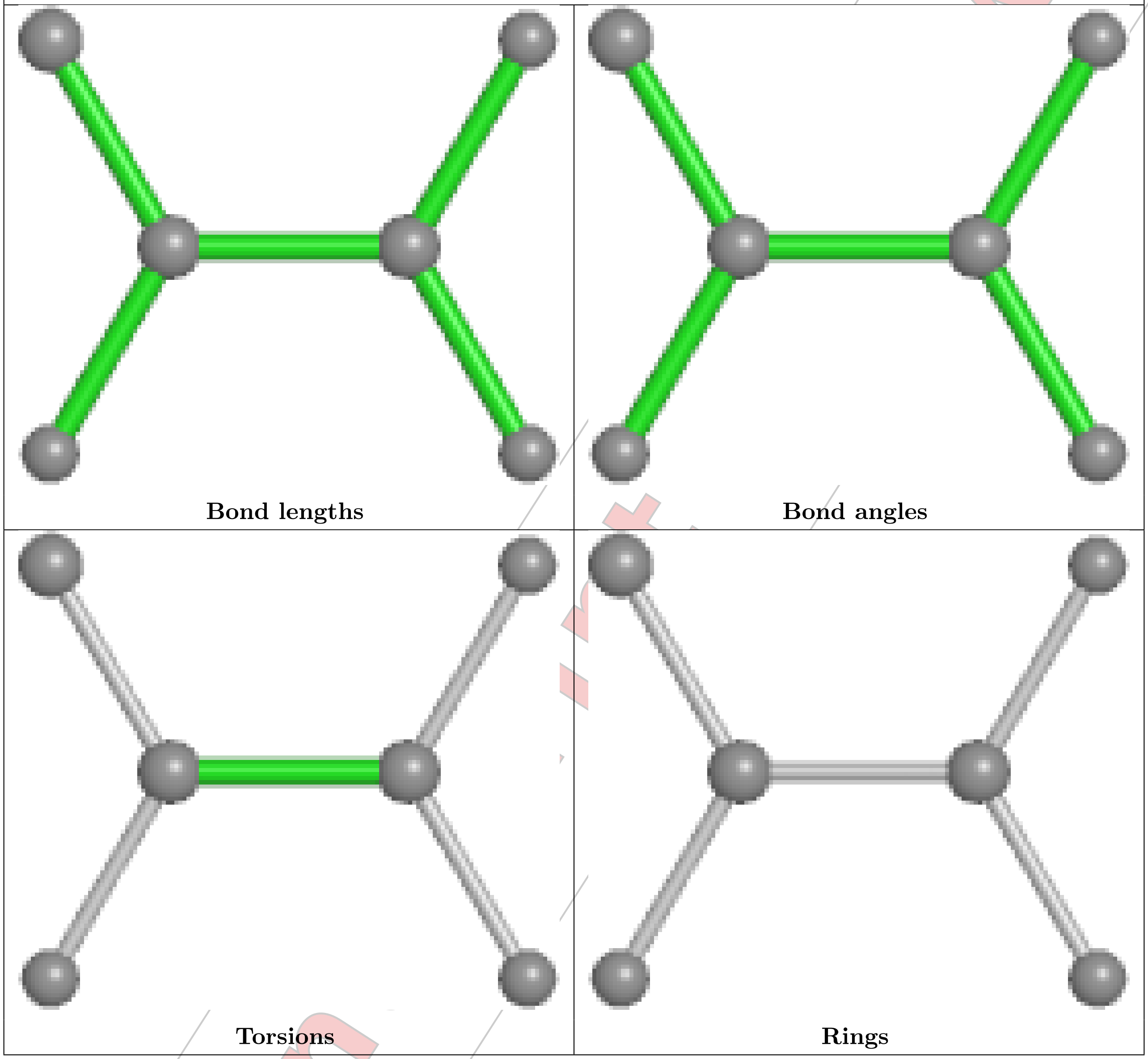	

5.7 Other polymers (i)

There are no such residues in this entry.

5.8 Polymer linkage issues (i)

There are no chain breaks in this entry.

For Manuscript Review

6 Fit of model and data (i)

6.1 Protein, DNA and RNA chains (i)

In the following table, the column labelled '#RSRZ> 2' contains the number (and percentage) of RSRZ outliers, followed by percent RSRZ outliers for the chain as percentile scores relative to all X-ray entries and entries of similar resolution. The OWAB column contains the minimum, median, 95th percentile and maximum values of the occupancy-weighted average B-factor per residue. The column labelled 'Q< 0.9' lists the number of (and percentage) of residues with an average occupancy less than 0.9.

Mol	Chain	Analysed	<RSRZ>	#RSRZ>2			OWAB(Å^2)	Q<0.9
1	A	564/572 (98%)	-0.11	6 (1%)	77	77	24, 35, 56, 70	0
1	B	563/572 (98%)	0.18	17 (3%)	52	51	24, 43, 66, 89	0
1	C	563/572 (98%)	0.46	64 (11%)	11	10	24, 41, 93, 116	0
1	D	564/572 (98%)	0.36	31 (5%)	32	31	24, 43, 73, 93	0
All	All	2254/2288 (98%)	0.22	118 (5%)	34	33	24, 40, 75, 116	0

All (118) RSRZ outliers are listed below:

Mol	Chain	Res	Type	RSRZ
1	B	345	ALA	7.3
1	C	563	VAL	5.1
1	C	364	LEU	4.7
1	C	368	VAL	4.2
1	B	159	LEU	4.2
1	C	332	TRP	4.2
1	B	366	ALA	3.9
1	C	367	ILE	3.8
1	C	449	PRO	3.8
1	D	382	ILE	3.7
1	D	441	LEU	3.7
1	A	136	VAL	3.6
1	C	331	ILE	3.6
1	B	530	TYR	3.6
1	C	430	PHE	3.6
1	D	377	ILE	3.5
1	C	383	GLY	3.5
1	C	339	LEU	3.5
1	C	358	HIS	3.5
1	C	386	PHE	3.4
1	C	338	GLY	3.4

Continued on next page...

For Manuscript Review

Continued from previous page...

Mol	Chain	Res	Type	RSRZ
1	C	366	ALA	3.4
1	D	334	VAL	3.4
1	C	382	ILE	3.4
1	B	6	PRO	3.4
1	C	441	LEU	3.3
1	C	566	ILE	3.2
1	C	302	ALA	3.2
1	D	332	TRP	3.2
1	C	446	THR	3.2
1	C	447	LEU	3.2
1	D	302	ALA	3.2
1	C	394	MET	3.2
1	D	379	VAL	3.1
1	B	566	ILE	3.1
1	D	6	PRO	3.0
1	B	567	GLN	3.0
1	C	421	TYR	3.0
1	C	362	LYS	3.0
1	C	417	ALA	2.9
1	C	340	ILE	2.9
1	C	497	GLY	2.9
1	C	440	THR	2.9
1	C	409	PRO	2.9
1	C	468	CYS	2.9
1	C	276	VAL	2.8
1	D	278	VAL	2.8
1	C	379	VAL	2.8
1	B	155	ARG	2.8
1	C	410	THR	2.7
1	B	564	GLN	2.7
1	D	569	LYS	2.7
1	C	309	ILE	2.7
1	C	438	PRO	2.7
1	C	381	ALA	2.7
1	C	423	ILE	2.7
1	A	137	LEU	2.6
1	C	395	ALA	2.6
1	D	339	LEU	2.6
1	B	158	GLY	2.6
1	C	397	PHE	2.6
1	D	381	ALA	2.5
1	C	387	SER	2.5

Continued on next page...

For Manuscript Review

Continued from previous page...

Mol	Chain	Res	Type	RSRZ
1	C	378	GLY	2.5
1	C	413	ALA	2.5
1	C	435	PRO	2.5
1	C	297	ILE	2.5
1	D	486	ALA	2.4
1	C	359	GLU	2.4
1	C	333	LEU	2.4
1	C	414	GLU	2.3
1	C	298	LEU	2.3
1	C	301	GLY	2.3
1	C	376	LEU	2.3
1	D	10	HIS	2.3
1	D	12	HIS	2.3
1	C	391	LEU	2.3
1	B	395	ALA	2.3
1	C	300	GLN	2.3
1	D	341	VAL	2.3
1	D	383	GLY	2.3
1	C	5	ALA	2.3
1	B	346	SER	2.3
1	A	203	LEU	2.2
1	D	347	LEU	2.2
1	C	565	LYS	2.2
1	A	377	ILE	2.2
1	C	396	ALA	2.2
1	C	424	THR	2.2
1	D	348	THR	2.2
1	D	380	ALA	2.2
1	D	442	PRO	2.2
1	C	341	VAL	2.2
1	D	322	LEU	2.1
1	D	447	LEU	2.1
1	D	416	SER	2.1
1	D	294	ASP	2.1
1	C	439	VAL	2.1
1	B	563	VAL	2.1
1	C	334	VAL	2.1
1	C	427	ARG	2.1
1	C	347	LEU	2.1
1	D	378	GLY	2.1
1	B	399	GLU	2.1
1	B	441	LEU	2.1

Continued on next page...

For Manuscript Review

Continued from previous page...

Mol	Chain	Res	Type	RSRZ
1	B	397	PHE	2.1
1	D	371	ILE	2.1
1	A	5	ALA	2.0
1	A	444	GLY	2.0
1	B	502	PRO	2.0
1	D	9	ARG	2.0
1	D	250	VAL	2.0
1	C	328	ILE	2.0
1	C	403	ILE	2.0
1	D	158	GLY	2.0
1	D	325	GLU	2.0
1	C	277	ALA	2.0
1	C	448	TYR	2.0

6.2 Non-standard residues in protein, DNA, RNA chains (i)

There are no non-standard protein/DNA/RNA residues in this entry.

6.3 Carbohydrates (i)

There are no oligosaccharides in this entry.

6.4 Ligands (i)

In the following table, the Atoms column lists the number of modelled atoms in the group and the number defined in the chemical component dictionary. The B-factors column lists the minimum, median, 95th percentile and maximum values of B factors of atoms in the group. The column labelled 'Q< 0.9' lists the number of atoms with occupancy less than 0.9.

Mol	Type	Chain	Res	Atoms	RSCC	RSR	B-factors(Å^2)	Q<0.9
2	NAP	C	601	48/48	0.71	0.16	50,86,121,128	0
2	NAP	D	601	48/48	0.81	0.15	57,80,142,151	0
4	2OP	C	602	6/6	0.90	0.13	37,52,54,58	0
4	2OP	D	602	6/6	0.90	0.12	41,47,49,54	0
2	NAP	B	601	48/48	0.94	0.08	34,43,47,56	0
2	NAP	A	601	48/48	0.97	0.06	29,35,40,42	0
3	MN	C	603	1/1	0.98	0.03	34,34,34,34	0
3	MN	D	603	1/1	0.99	0.04	37,37,37,37	0
3	MN	B	602	1/1	1.00	0.04	40,40,40,40	0
3	MN	A	602	1/1	1.00	0.02	31,31,31,31	0

For Manuscript Review

The following is a graphical depiction of the model fit to experimental electron density of all instances of the Ligand of Interest. In addition, ligands with molecular weight > 250 and outliers as shown on the geometry validation Tables will also be included. Each fit is shown from different orientation to approximate a three-dimensional view.

Electron density around NAP C 601:

$2mF_o$-DF_c (at 0.7 rmsd) in gray
mF_o-DF_c (at 3 rmsd) in purple (negative)
and green (positive)

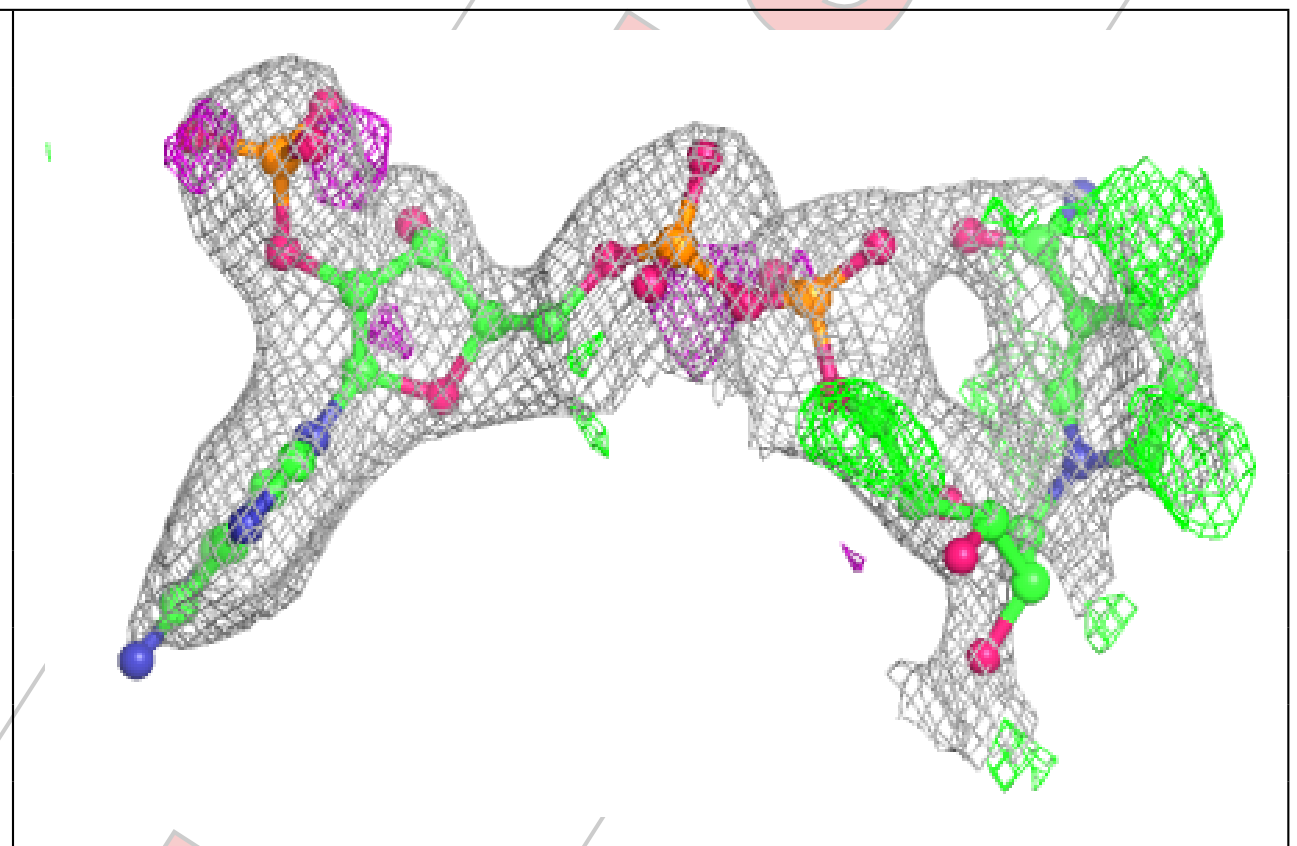

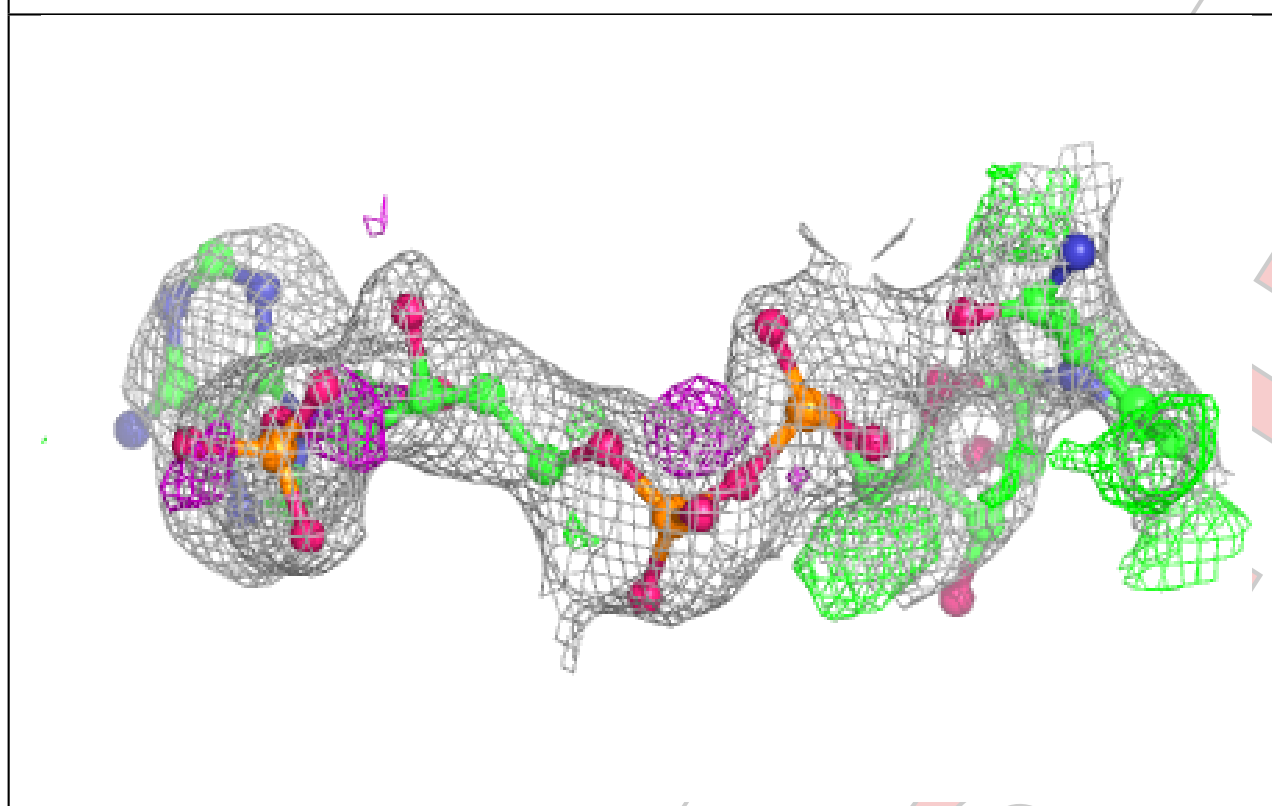

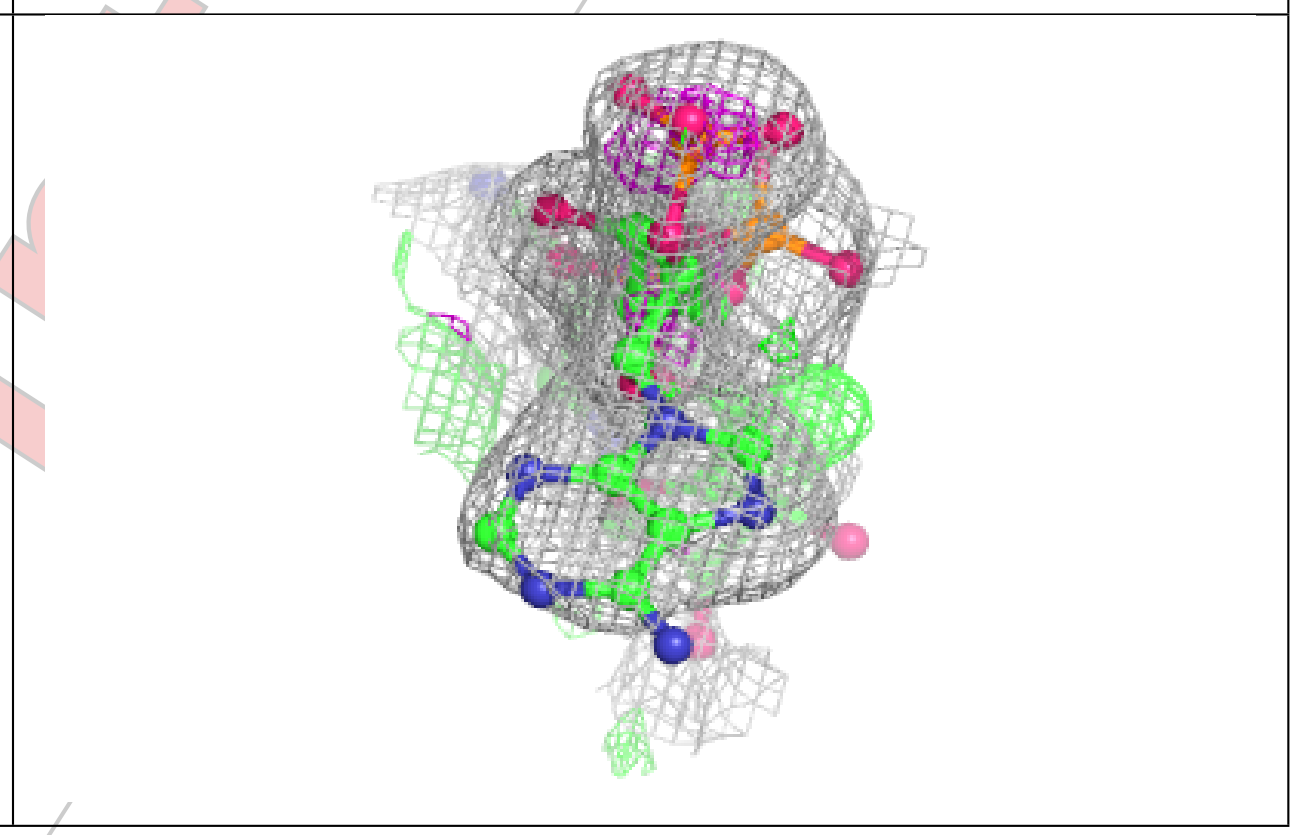

For Manuscript Review

Electron density around NAP D 601:

2mF$_o$-DF$_c$ (at 0.7 rmsd) in gray
mF$_o$-DF$_c$ (at 3 rmsd) in purple (negative)
and green (positive)

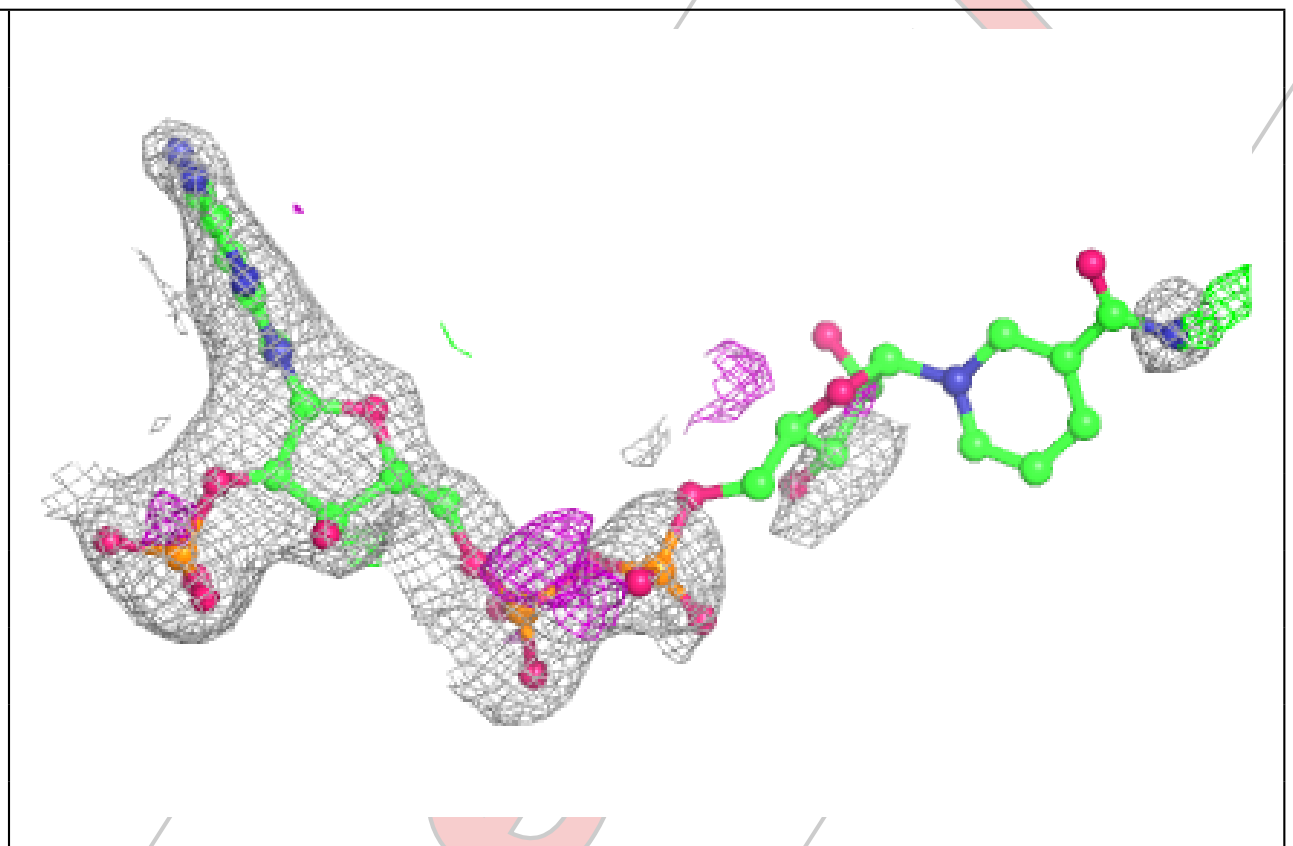

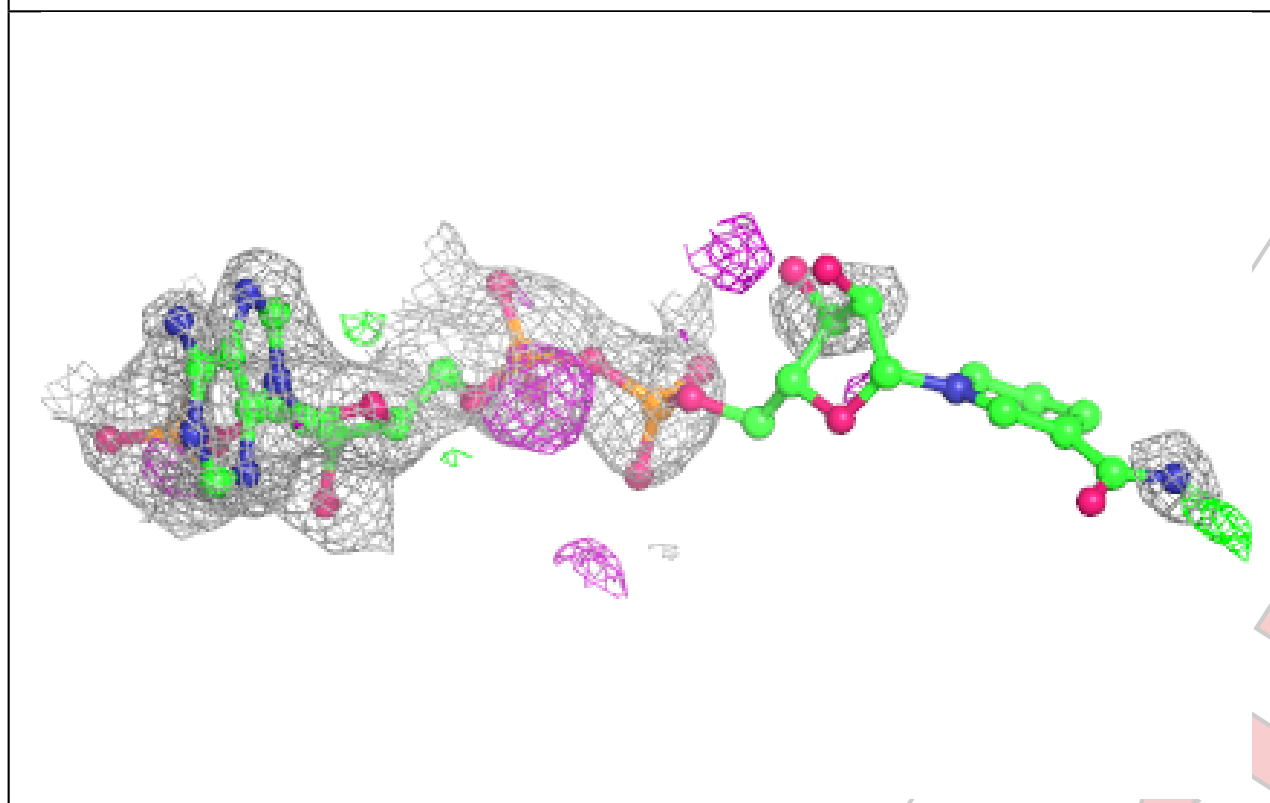

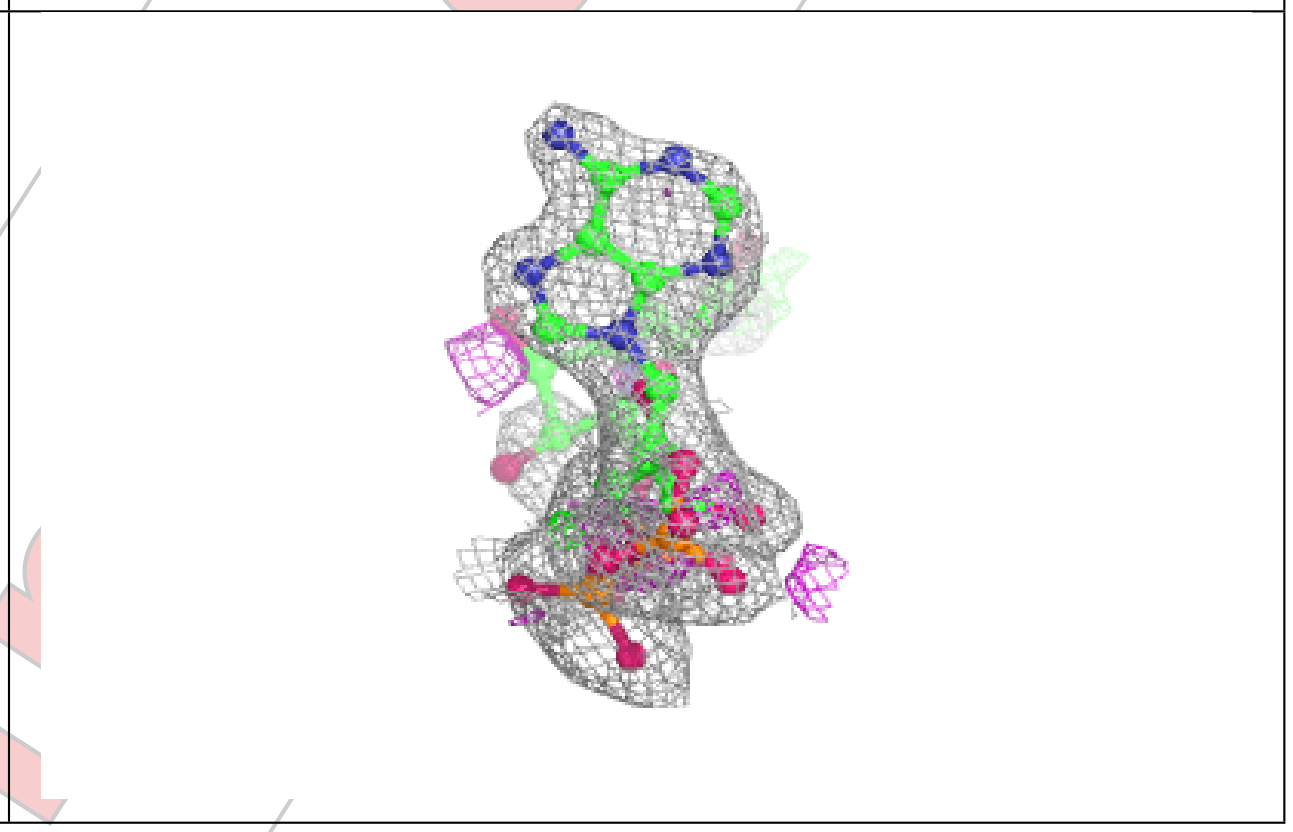

For Manuscript Review

Electron density around 2OP C 602:

2mF$_o$-DF$_c$ (at 0.7 rmsd) in gray
mF$_o$-DF$_c$ (at 3 rmsd) in purple (negative)
and green (positive)

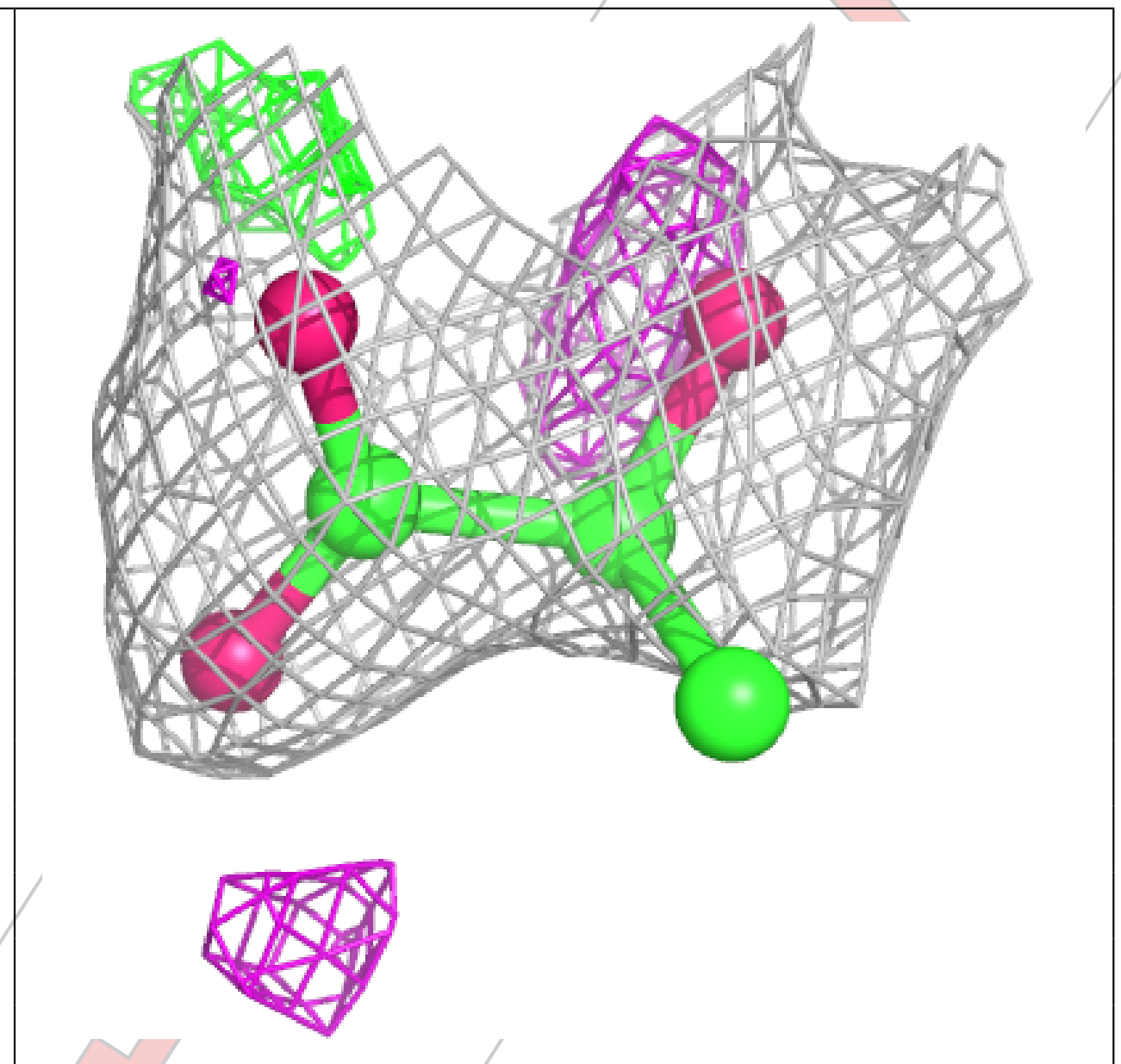

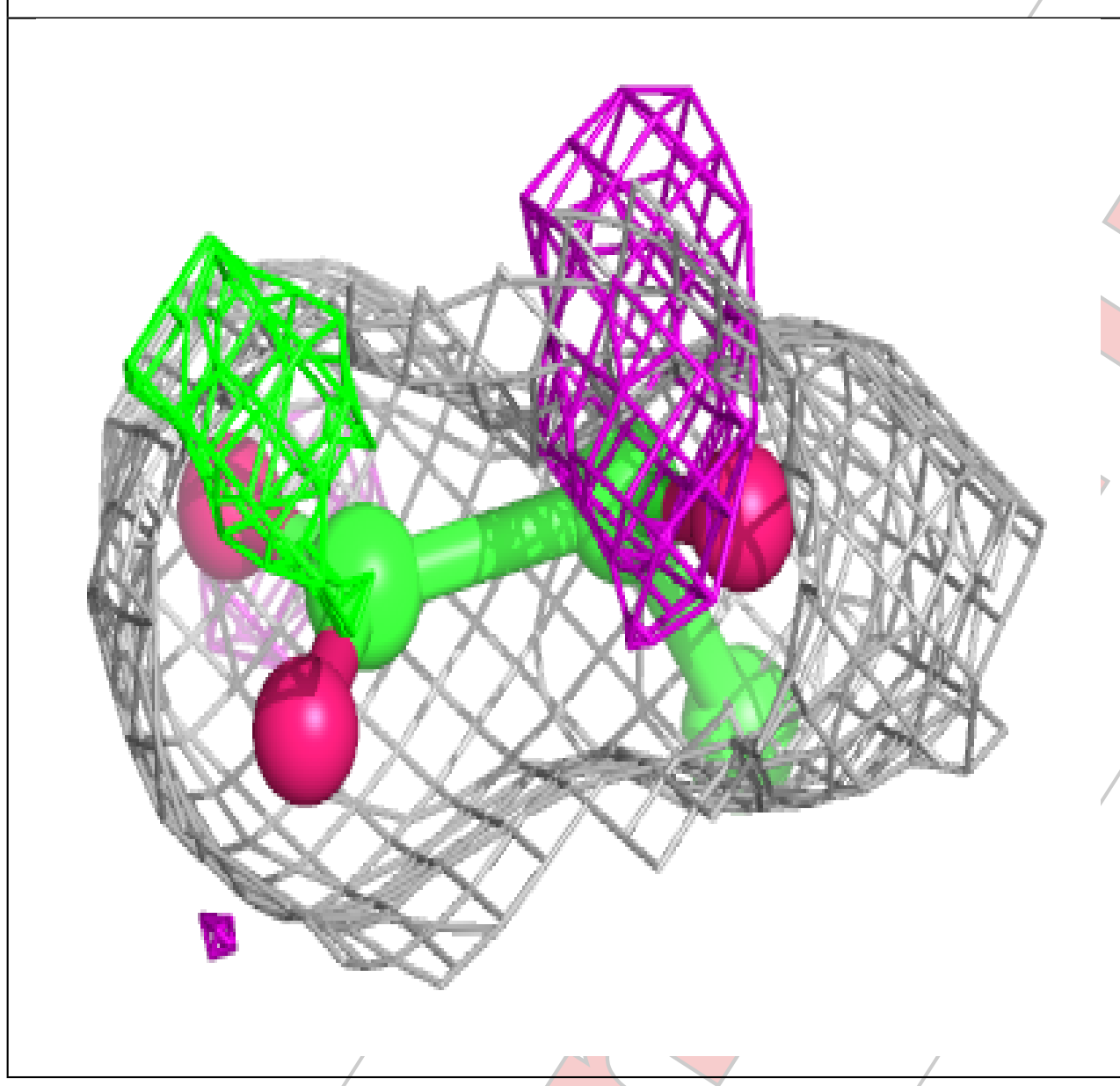

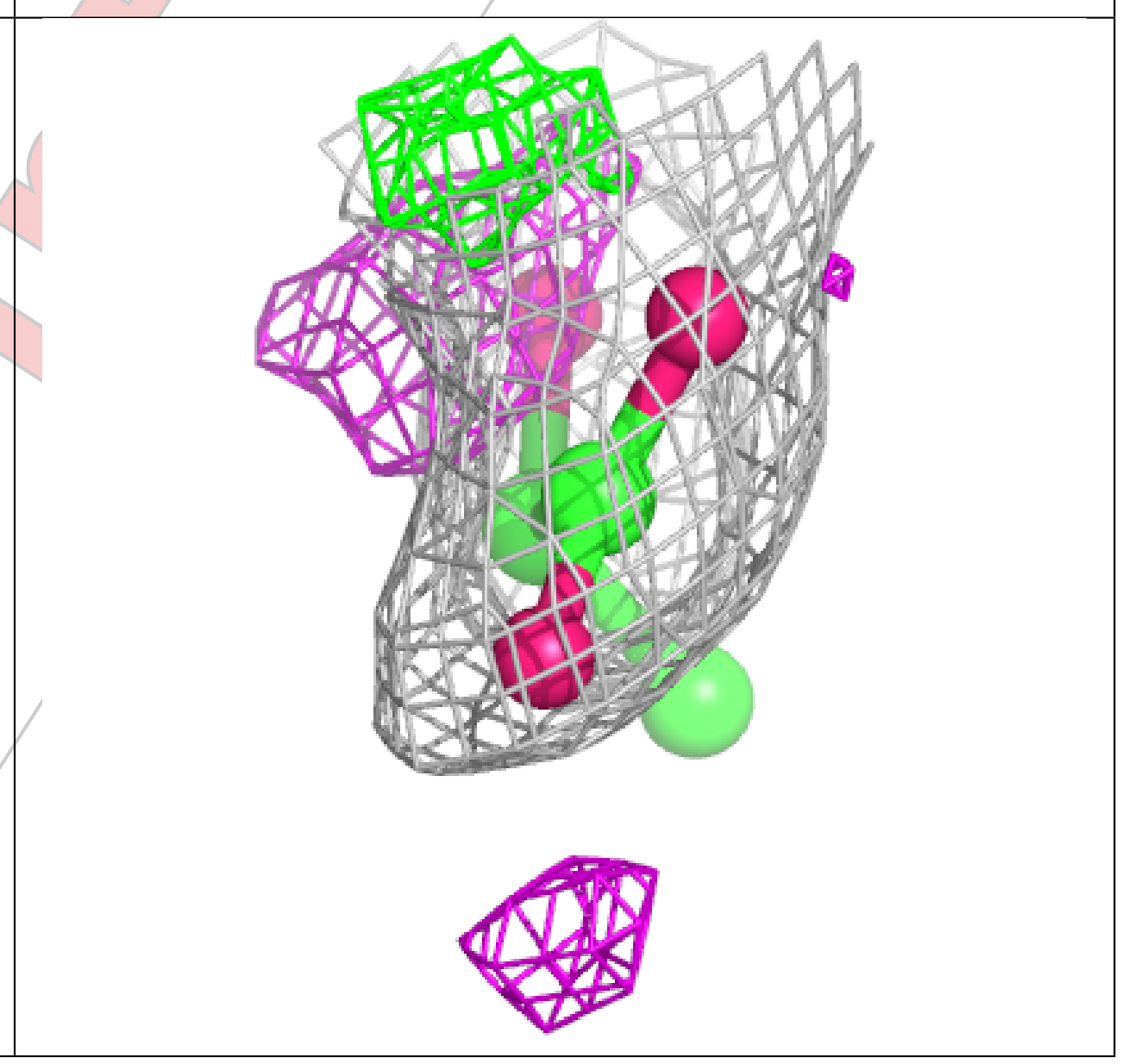

For Manuscript Review

Electron density around 2OP D 602:

$2mF_o$-DF_c (at 0.7 rmsd) in gray
mF_o-DF_c (at 3 rmsd) in purple (negative)
and green (positive)

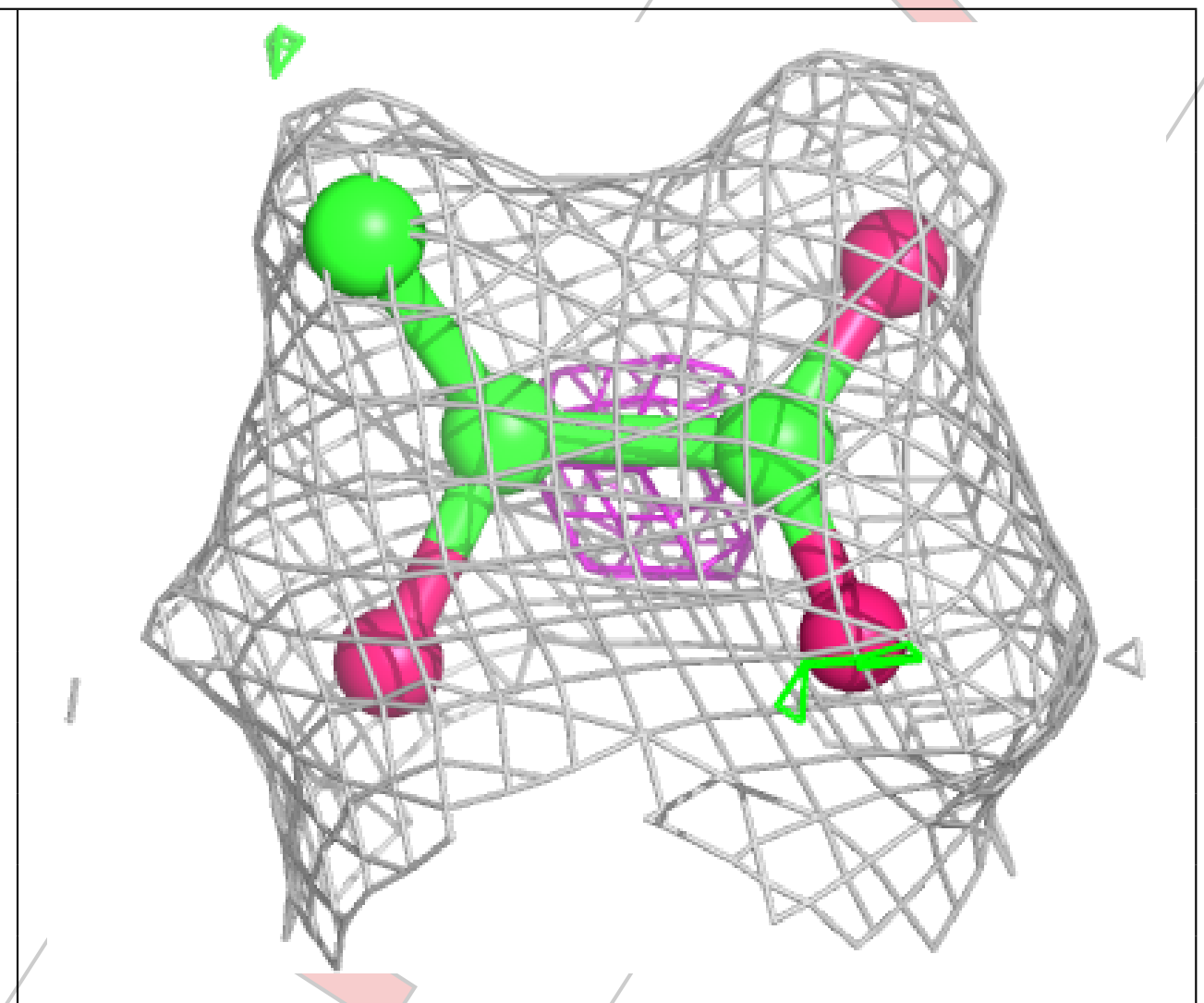

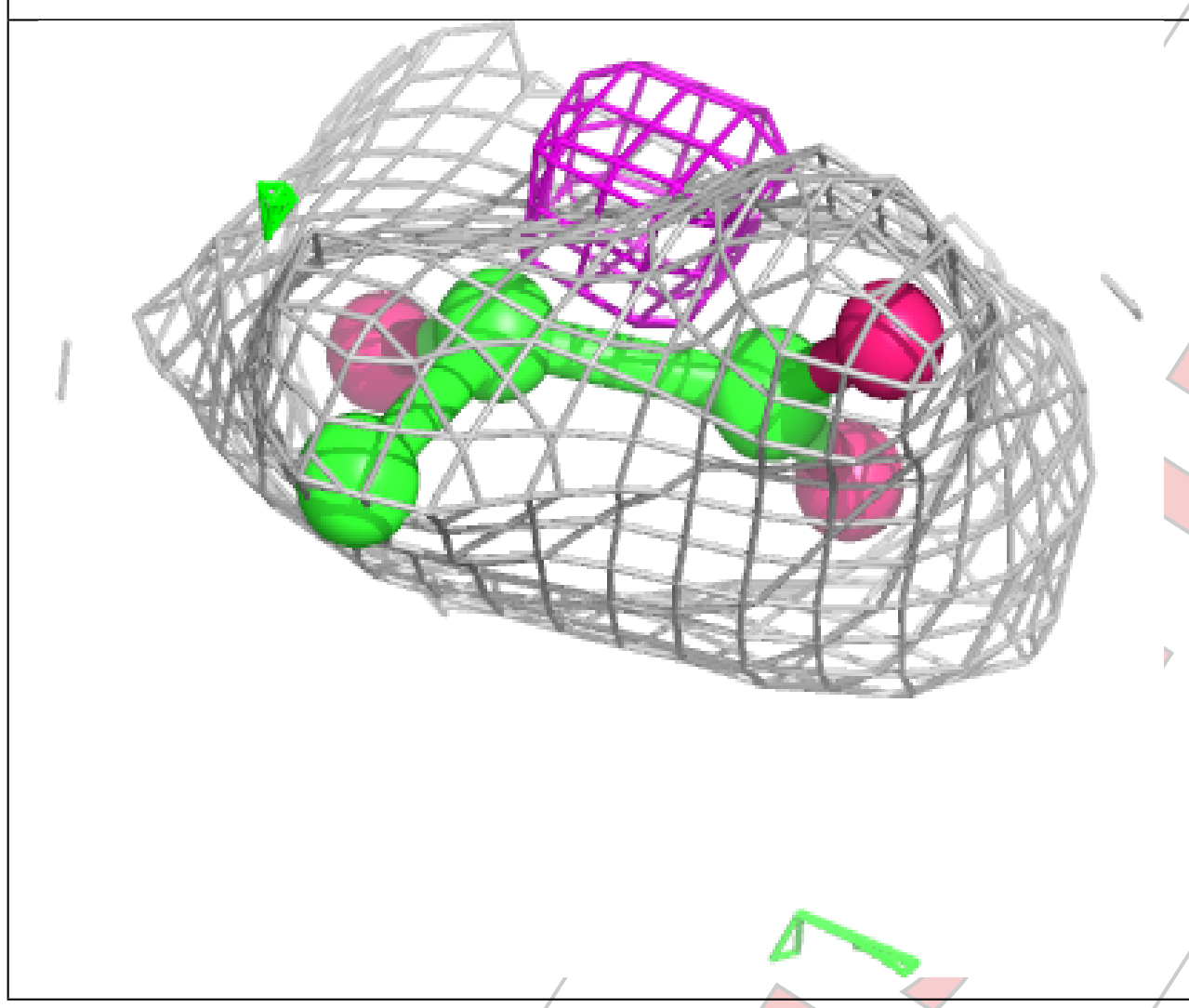

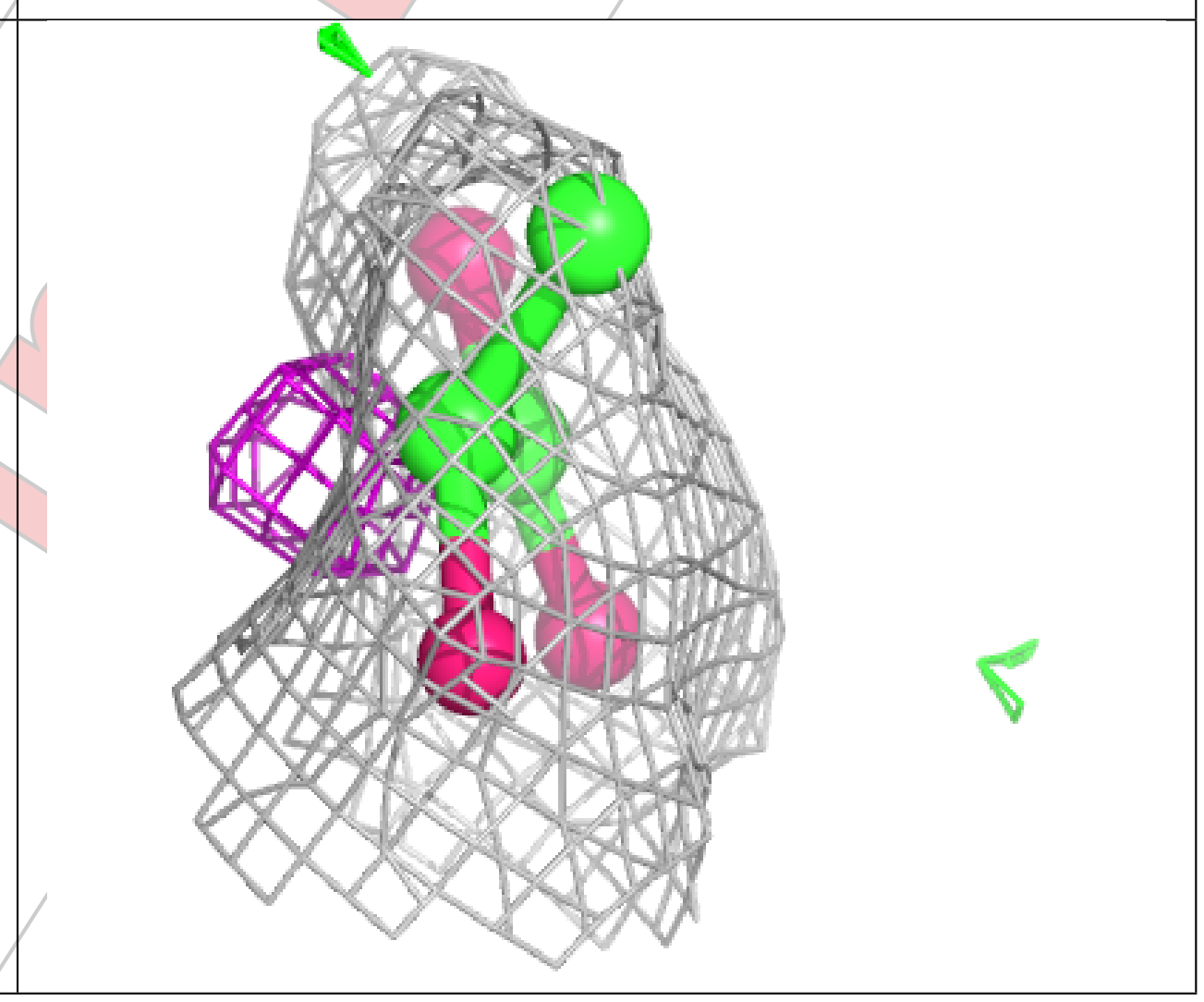

For Manuscript Review

Electron density around NAP B 601:

$2mF_o$-DF_c (at 0.7 rmsd) in gray
mF_o-DF_c (at 3 rmsd) in purple (negative)
and green (positive)

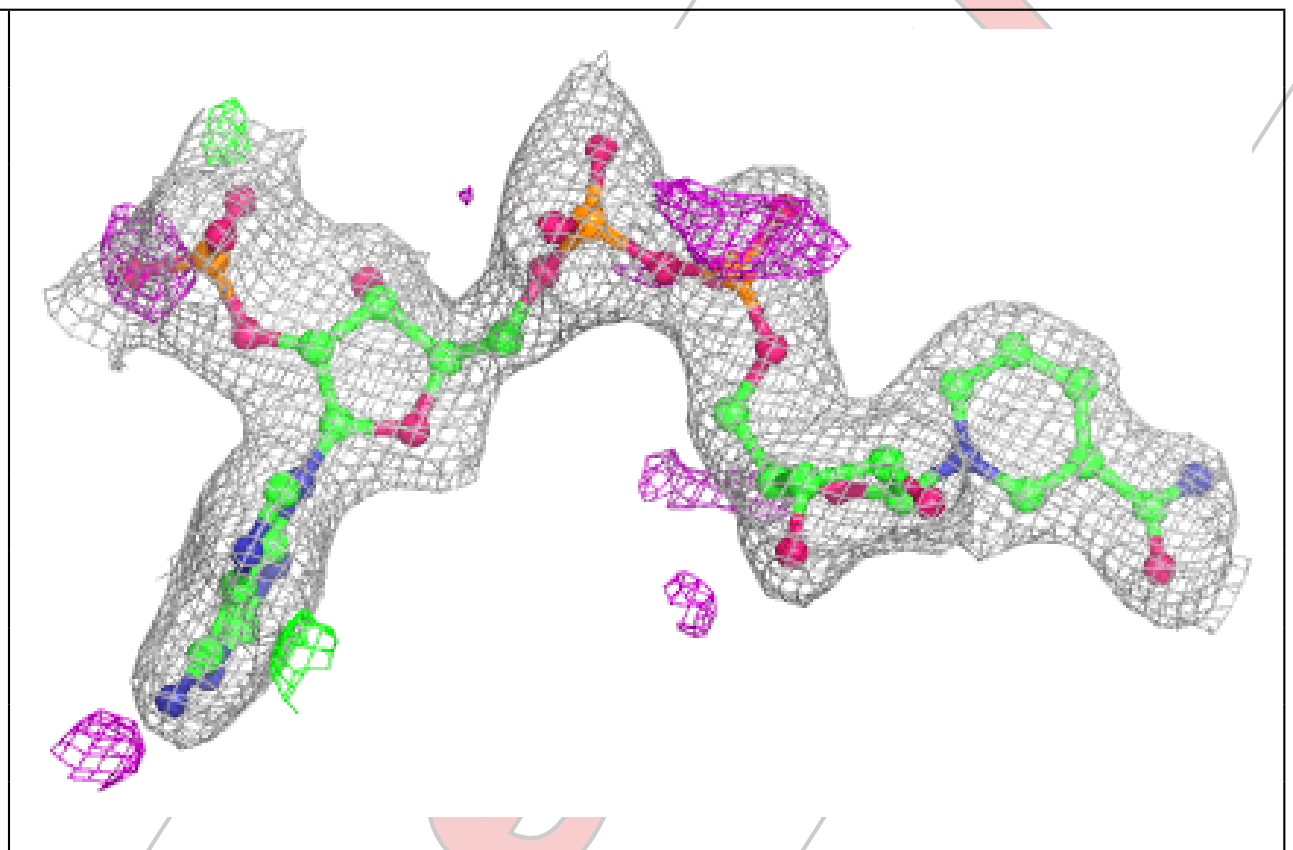

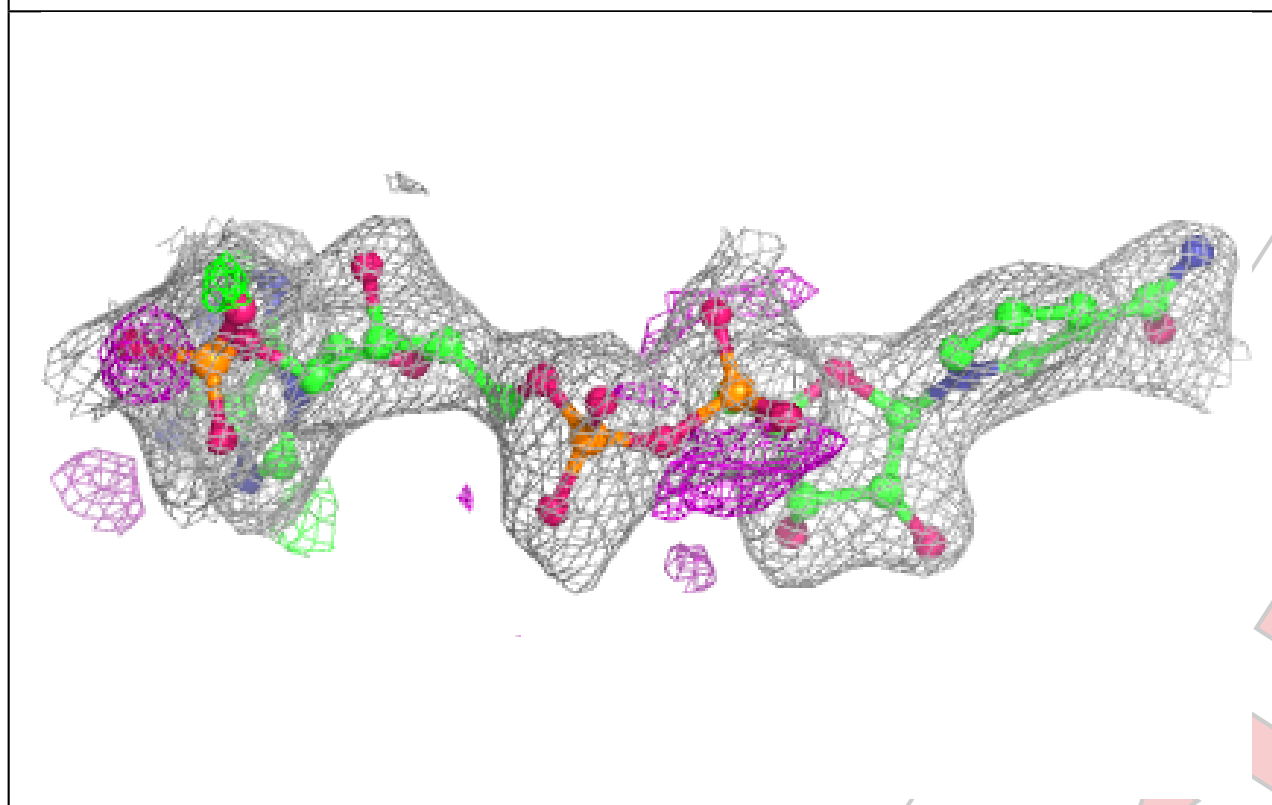

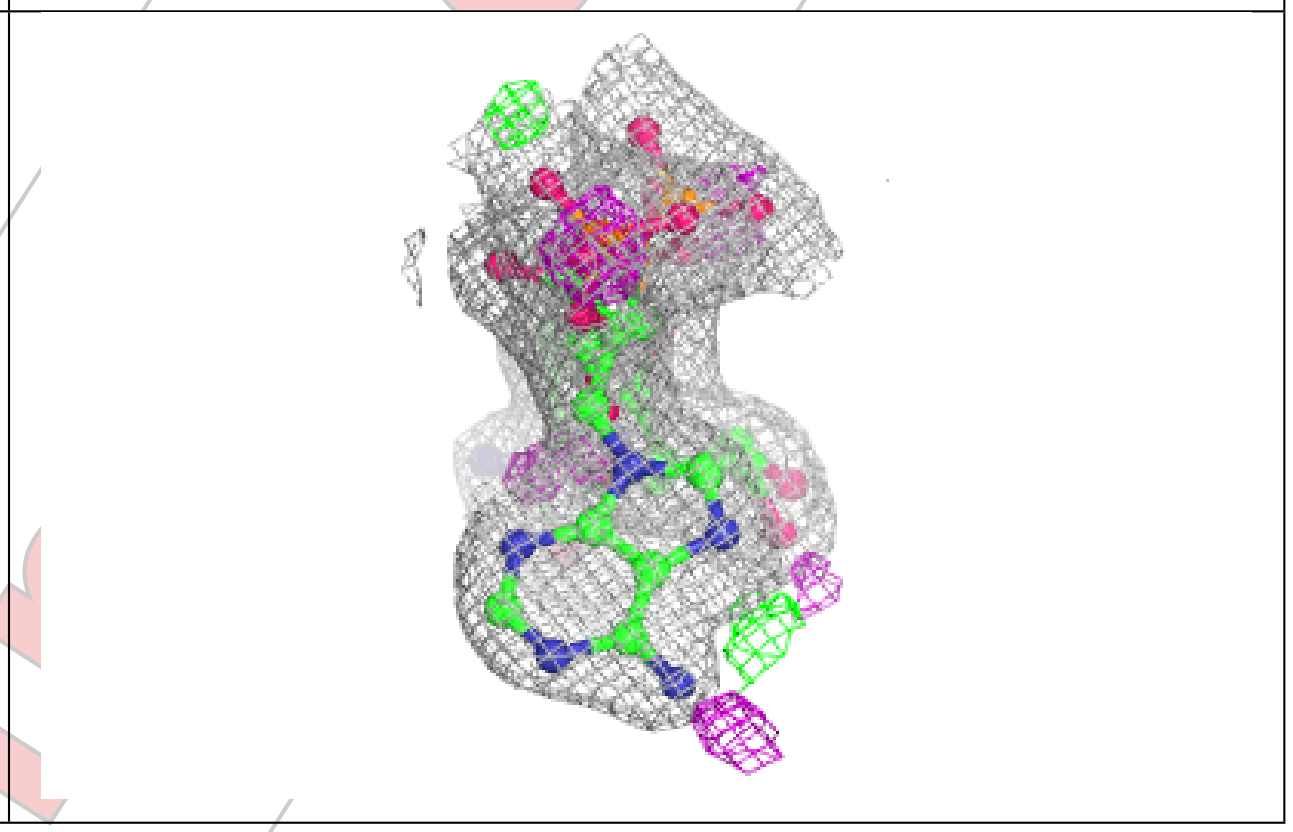

Electron density around NAP A 601:

$2mF_o$-DF_c (at 0.7 rmsd) in gray
mF_o-DF_c (at 3 rmsd) in purple (negative)
and green (positive)

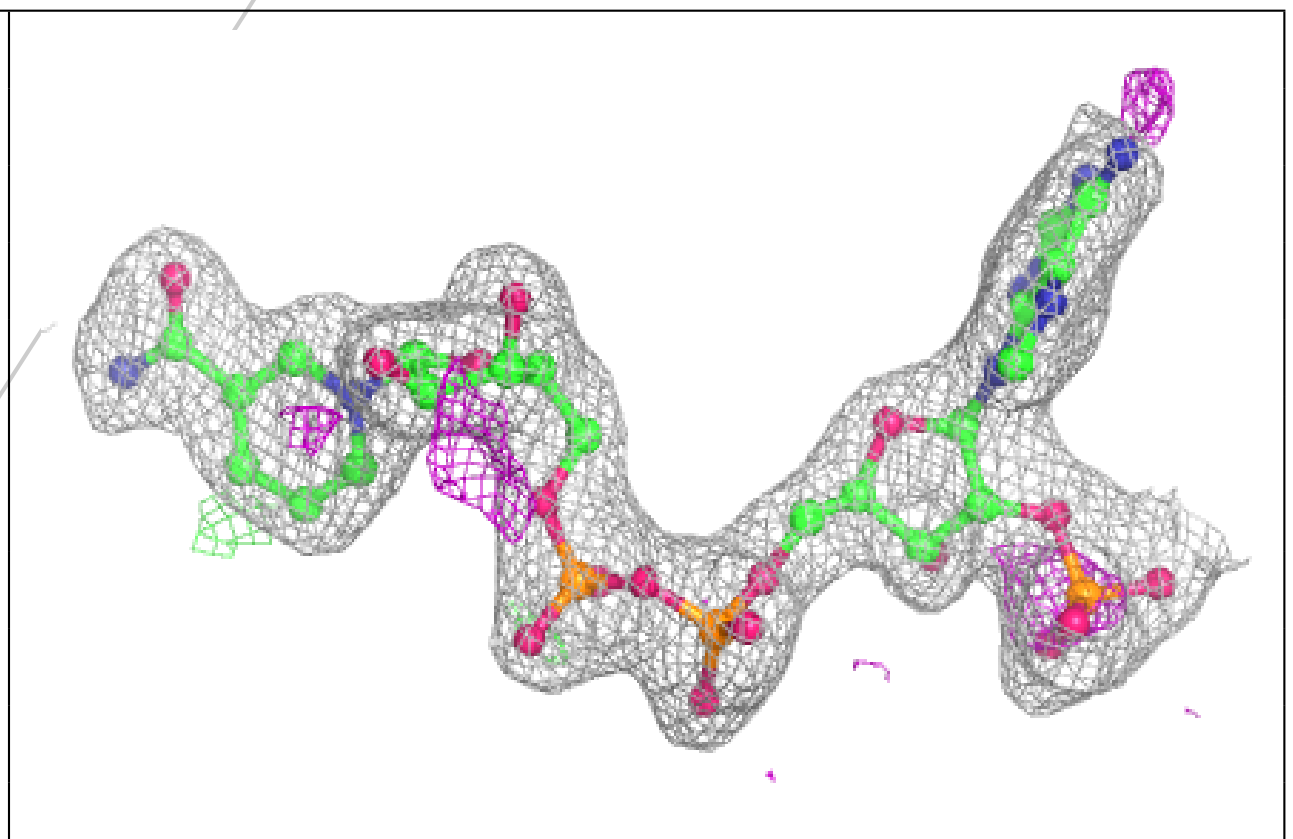

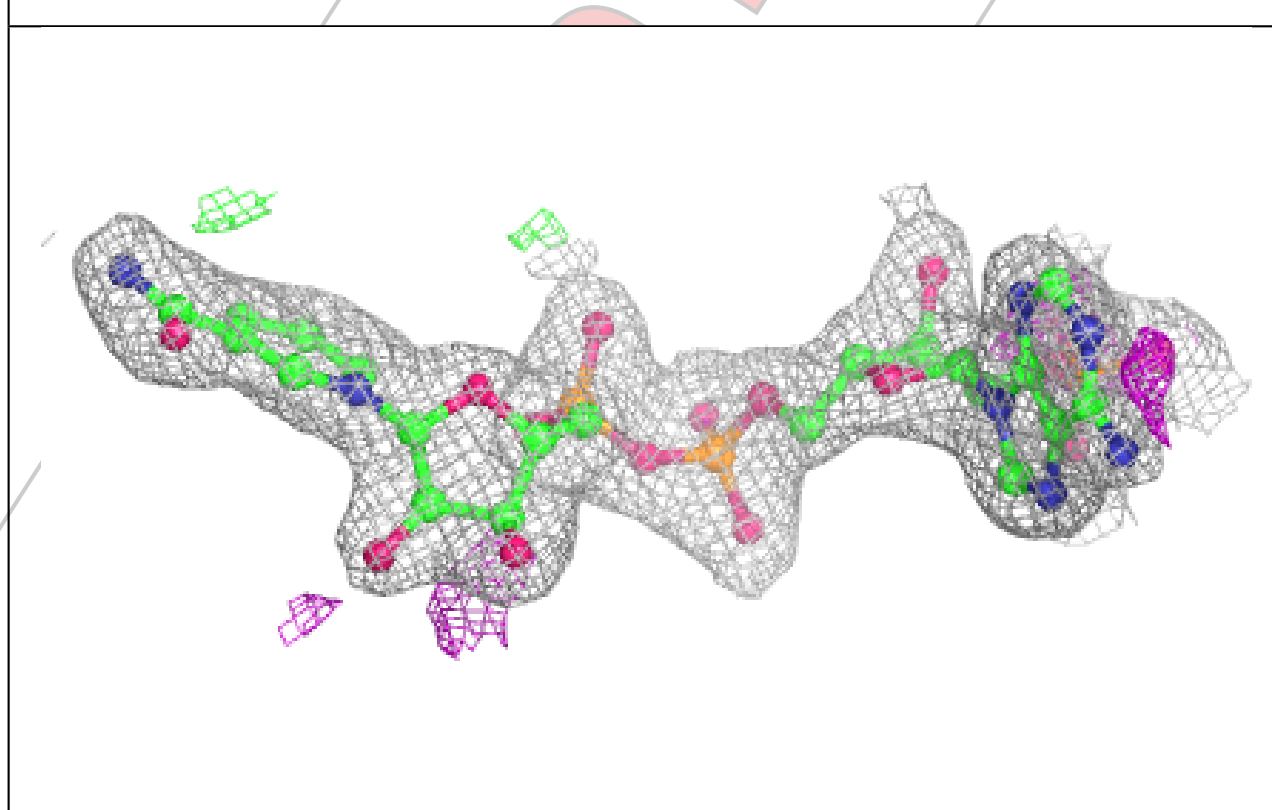

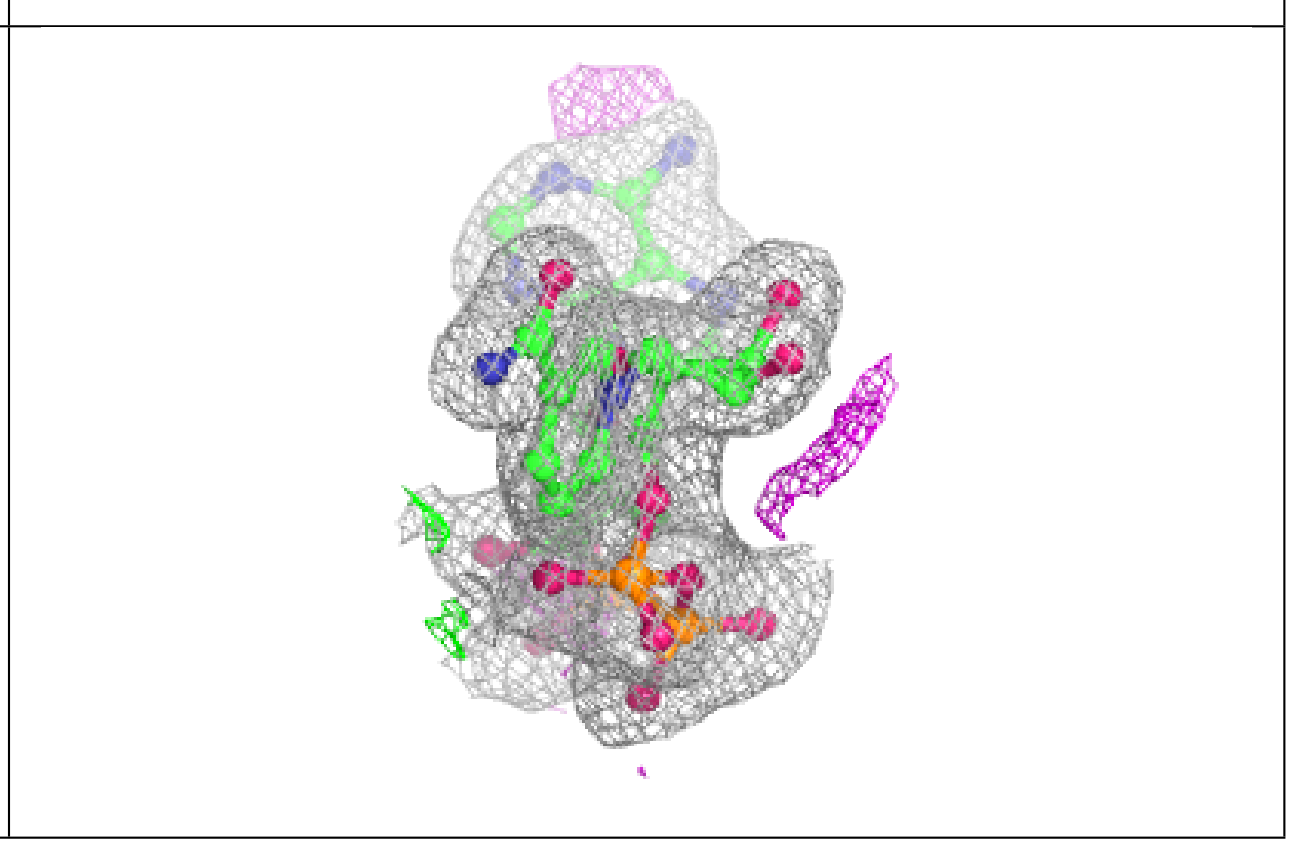

6.5 Other polymers (i)

There are no such residues in this entry.

For Manuscript Review
