# Supplementary material for: Malic enzyme 1 senses L-lactate to determine tumor heterogeneity
Source: Signal Transduct Target Ther. 2026 Sep 11;11:386. doi: 10.1038/s41392-026-02839-6 (PMC13569829; doi:10.1038/s41392-026-02839-6)
Supplement: Supplementary file 4 [file 41392_2026_2839_MOESM1_ESM.docx]

Supplementary Materials for

**Malic Enzyme 1 Senses L-lactate to Determine Tumor Heterogeneity**

Si-Yi Cao^1,2,7^, Kewen Hu^1,2,7^, Shijing Huang^1,7^, Xiao-Lin Guan^1,2,7^, Jian Wang^1,2^, Yuan Shen^1,2^, Hui Ming^1,2^, Aihong Gu^1^, Chao Wang^1,2^, Xiao Shen ^1,2^, Qing Wu^1,2^, Zhengjun Chen^6^, Miao Yin^1,2*^, Wenyu Wen^1,3,4*^, Qun-Ying Lei^1,2,4, 5*^

Correspondence to: [qlei@fudan.edu.cn](mailto:qlei@fudan.edu.cn), wywen@fudan.edu.cn or [miaoyin@fudan.edu.cn](mailto:miaoyin@fudan.edu.cn).

**This PDF file includes:**

Figures. S1 to S9

Tables S1 to S2

**Other Supplementary Materials for this manuscript include the following:**

Data S1 to S3

Supplementary Figures

Figure.S1

**Supplementary Fig. 1 ME1 expression is induced by L-lactate and elevated in prostate cancer.** **a,** The indicated concentrations of sodium L-lactate have no effect on pH value of culture media. **b,** Sodium L-lactate increases cellular lactate level in PC3 cell under normal medium (mean ± s.e.m., unpaired t test). **c**, Sodium L-lactate suppresses cell growth in prostate cell lines PC3 and DU145 cultured with normal medium. Quantifications of the cell numbers are shown (mean ± s.e.m., One-way ANOVA). **d**, Sodium L-lactate increases cellular lactate level in PC3 cell under nutrient-depleted medium (2.5 mM glucose and 0.5 mM glutamine) (mean ± s.e.m., unpaired t test). **e**, Quantification of PC3 cell numbers in the normal (12.5 mM glucose and 2 mM glutamine) and nutrient-depleted medium with 30 mM sodium L-lactate or NaCl as the control group are shown (mean ± s.e.m., Two-way ANOVA). **f**, **g**, The protein levels of the indicated metabolic enzymes in DU145 cells cultured in low pyruvate, glutamine, and glucose nutrient-depleted medium with different concentrations of sodium L-lactate for 48 h by WB analysis. 1 mM pyruvate, 25 mM glucose, and 4 mM glutamine in normal medium. 0.1 mM pyruvate, 2.5 mM glucose, and 0.4 mM glutamine in nutrient-depleted medium. Representative WB images (**f**) and quantification (**g**) are shown (mean ± s.e.m., One-way ANOVA for ME1, ME2, ME3, LDHA, LDHB, HK2, PKM2, and GLS, Welch ANOVA for CS). **h**, The mRNA expression level of metabolic enzymes in DU145 cells under different concentrations of sodium L-lactate in the nutrient-depleted medium for 48 hours (mean ± s.e.m., One-way ANOVA). **i,** L-lactate increases ME1 expression level in A549 and HCT116 cells. **j**, ME1 is highly expressed in the tumor tissue of human prostate cancer compared to adjacent normal tissue. Representative IHC images for ME1 expression in human prostate cancer samples (left panel) and quantification (Adjacent normal tissue n = 50, Tumor tissue n = 100 biologically independent patients, Mann-Whitney test) (right panel) are shown. Scale bar, 300 μm. **k**, Survival of patients with low (4744 cases) versus high (4744 cases) ME1 levels (left), low (4744 cases) versus high (4744 cases) are compared by TCGA database analysis.

**Figure. S2**

**Supplementary Fig. 2 Characterization of *Me1^pc-/-^* and *Pten^pc-/-^; Me1^pc-/-^* mice. a,** Generation of prostate tissue-specific *Me1*-knockout mice. **b,** Prostate weight of *Me1^pc-/-^* and *Me1^pc+/+^* mice (mean ± s.e.m., unpaired t test). **c,** Representative images of H&E-stained prostates from *Me1^pc-/-^* and *Me1^pc+/+^* mice. Scale bar, 40 μm. **d,** Verification for efficiency of *Me1* knockout. **e,** Representative IHC staining images of αSMA of *Pten^pc+/+^*, *Pten^pc-/-^,* and *Pten^pc-/-^; Me1^pc-/-^* mice. Scale bar, 40 μm. **f**, Representative IHC staining images and quantification of Ki-67 of *Pten^pc+/+^*, *Pten^pc-/-^,* and *Pten^pc-/-^; Me1^pc-/-^* mice. Scale bar, 40 μm.

**Figure. S3**

**Supplementary Fig. 3 Crystal structures of hME1 complexes and lactate- ME1 binding *in vivo*. a**, Ribbon representation of the tetrameric hME1-NADP^+^-Mn^2+^ complex. Monomers A-D are colored in magenta, orange, blue and green, respectively. NADP^+^ (in cyan for carbon atoms) is drawn in the ball-and-stick model, and Mn^2+^ is shown as a purple sphere. **b,** Ribbon representation of the monomer of hME1-lactate-NADP^+^-Mn^2+^ complex. The four domains are colored in pink (A), wheat (B), green (C), and cyan (D). The active site is indicated with a red rectangle. **c,** Images of the 2Fo-Fc map for the NADP^+^ and lactate molecules in the hME1-lactate-NADP^+^-Mn^2+^ complex structure at 2.03 Å contoured at 1.0 σ. **d,** Superimposition (with domains A, B, and D) of the monomer D (green) with monomer A (pink) or C (blue). NADP^+^ and lactate are drawn in the ball-and-stick model. For clarify, ME1 and Mn^2+^ are omitted in the figure. **e,** Schematic drawing showing the hydrogen-bonding interactions between hME1 and NADP^+^ in the active site. **f**, Schematic drawing showing the hydrogen-bonding interactions between hME1 and lactate in the active site. (**e**) and (**f**) are drawn by the LIGPLOT program. **g,** The binding affinities of L-lactate and the ME1^WT^ or ME1^R155A^, malate and ME1^WT^ were measured by isothermal titration calorimetry (ITC)- based analysis. **h,** Biotin-lactate pull down assay shows ME1 binding to lactate in PC3, A549, and HCT116 cells. **i,** Biotin-lactate pull down assay shows ME1 binding to lactate in PC3 cells with different concentration of malate treatment. **j,** L-lactate stabilizes ME1 protein in *ME1sg* PC3 cell expressing ME1^WT^ not ME1^R155A^ cells by cellular thermal shift assay (CETSA) analysis.

**Figure. S4**

**Supplementary Fig. 4 The conformational changes of ME1 upon binding of cofactor and L-lactate.** **a**, Human ME1 in the apo form (PDB: 3WJA). **b,** Human ME1 complexed with NADP^+^ and Mn^2+^ (this study, PDB: 23JW). **c,** Human ME1 complexed with NADP^+^, Mn^2+^ and Lactate (monomer D, this study, PDB: 23JV). **d**, Sodium L-lactate inhibits enzymatic activity of recombinant ME1 produced by prokaryotic expression system in a dose-dependent manner according to the analysis of *in vitro* enzymatic reaction assay. **e,** A diagram for incorporation of ^13^C_4_ from malate to pyruvate catalyzed by ME1. **f,** Abundance of M+3 pyruvate level in PC3 cells with or without L-lactate treatment under normal or nutrient-depleted medium (mean ± s.e.m., unpaired t test). **g,** Total cellular Malate and pyruvate level are increased in PC3 cells cultured with L-lactate (for normal medium, 10 mM, for nutrient-depleted medium, 30 mM) (mean ± s.e.m., unpaired t test). **h**, The inhibited enzymatic activity of recombinant ME1^R155A^ compared to ME1^WT^ according to the analysis of *in vitro* enzymatic reaction assay. **i,** Abundance of M+3 pyruvate level in ME1^WT^ and ME1^R155A^ cells under normal or nutrient-depleted medium through ^13^C_4_ malate tracing (mean ± s.e.m., unpaired t test). **j,** Total cellular Malate and pyruvate level in *ME1sg* PC3 cells expressing ME1^WT^ or ME1^R155A^ (mean ± s.e.m., unpaired t test). **k,** Seahorse assay shows glycolysis rate in *ME1sg* PC3 cells expressing ME1^WT^ or ME1^R155A^ under normal medium with or without L-lactate (mean ± s.e.m., unpaired t test).

**Figure. S5**

**Supplementary Fig. 5 L-lactate-induced ME1 K362 acetylation increases ME1 nuclear translocation. a**, Representative immunofluorescence (IF) staining images for nuclear location of Me1 in the areas with relatively low or high expression level of Ki-67 in the prostate tissue from *Pten^pc-/-^* mice are shown (left panel). The dotted box represents the area with high Ki-67 expression level (arrowhead) and the area with low Ki-67 expression level is denoted by the solid box. The right panel shows the quantification for percentage of nuclear green fluorescence-positive cell numbers (asterisk) in the area with low or high expression level of Ki-67, respectively (two hundred cells counted for each area, n = 3 biologically independent animals). Scale bar, 10 μm. **b,** Sodium L-lactate promotes ME1 translocation to the nucleus in DU145 and PC3 cells cultured with normal medium. The ‘Ratio’ represents ME1/H3. **c**, Identification of ME1 binding to karyopherin-α 4 (KPNA4) but not other subtypes in the KPNA family in HEK293T cells by IP. **d**, *KPNA4* knockout inhibits ME1 nuclear translocation in PC3 cells cultured with normal medium (mean ± s.e.m., One-way ANOVA). **e,** L-lactate decreases acetylation level of ME1. PC3 cells were cultured in the normal medium. The ‘Ratio’ represents Pan-Ac/Flag. **f**, K362R mutation decreases ME1 acetylation level in HEK293T cells cultured in the normal medium. The ‘Ratio’ represents Pan-Ac/Flag. **g**, Specificity of the antibody recognizing acetylated K362 site (K362Ac antibody) is determined by dot blotting assay. **h**, L-lactate decreases K362 acetylation level of ME1. The ‘Ratio’ represents K362Ac/Flag. **i,** K362 acetylation level of ME1 with treatment of HDAC inhibitor TSA. **j,** Overexpression of SIRT5 decreases K362 acetylation level of ME1 in HEK293T cells cultured with normal medium. Overexpression of HA-tagged subtypes (1-7) of SIRT in HEK293T cells, respectively, followed by detection with anti-K362Ac. **k**, *SIRT5* knockout abrogates L-lactate-increased ME1 nuclear translocation in PC3 cells cultured in the normal medium with 10 mM sodium L-lactate (+) or NaCl for the control group (-). The ‘Ratio’ represents ME1/H3. **l, m,** L-lactate promotes ME1^WT^ binding to SIRT5 to decrease K362 acetylation and interaction with KPNA4 (**l**) to increase nuclear translocation (**m**) in A549 cells and HCT116 cells. R155A mutant blocks such effect. **n, o,** AZD3965 decreases K362 acetylation level through binding to SIRT5 (**n**) and to strengthen the interaction with KPNA4 (**n**) to increase nuclear translocation (**o**) in A549 cells and HCT116 cells.

**Figure. S6**

**Supplementary Fig. 6 L-lactate-increased ME1 nuclear translocation promotes epithelial-mesenchymal transition. a**, *ME1* knockout out decreases *ZEB2* mRNA level and increases *E-cadherin* mRNA level (mean ± s.e.m., One-way ANOVA). **b**, Identification of the binding between ME1 and HDGF. Flag-ME1^WT^, Flag-ME1^NES^, or Flag-ME1^NLS^ was transiently transfected in HEK293T cells for IP-MS analysis. The lists show the top 10 transcription factors according to fold change (WT/NES or NLS/NES). **c**, Sodium L-lactate (10 mM) enhances the interaction of ME1 and HDGF in PC3 cells cultured with normal medium. **d**, ME1 is indispensable to HDGF and/or L-lactate-activated pSmad signaling. Transient transfection of Myc-HDGF in *ME1sg* and *CTRL* PC3 cells cultured with normal medium, followed by treatment of 10 mM sodium L-lactate or NaCl. **e,** Sodium L-lactate promotes the migration and invasion ability of PC3 cells expressing ME1^WT^ but not ME1^NES^ (mean ± s.e.m., Two-way ANOVA). Scale bar, 100 μm. **f,** L-lactate binds to ME1^WT^ to increase cell migration and invasion while ME1^R155A^ mutant blocking such effect. NaCl treatment serves as control group (mean ± s.e.m., Two-way ANOVA). Scale bar, 100 μm.

**Figure.S7**

**Supplementary Fig. 7 L-lactate promotes the interaction between ME1 and LDHB to strengthen mitochondrial function. a,** L-lactate supports A549 and HCT116 cell growth in the nutrient-depleted medium (5 mM glucose and 1 mM glutamine) dependent on binding to ME1-R155 site. Quantification for the cell numbers with 30 mM sodium L-lactate or NaCl as the control group are shown (mean ± s.e.m., Two-way ANOVA). **b,** Quantification of the cell numbers of PC3, A549, and HCT116 with AZD3965 (1 μM) or DMSO treatment (mean ± s.e.m., Two-way ANOVA). **c,** Increased LDHB binding to ME1 with L-lactate treatment under nutrient-deficient medium in PC3, A549, and HCT116 cells. **d,** Sodium L-lactate (30 mM) (+) has no effect on the binding of ME1 to LDHA in PC3 cells cultured with nutrient-depleted medium (2.5 mM glucose and 0.5 mM glutamine) as NaCl treatment (30 mM) for control group (-). **e,** Sodium L-lactate promotes ME1 binding to LDHB in *ME1sg* A549 and HCT116 cells expressing ME1^WT^ but not ME1^R155A^ in the nutrient-depleted medium compared to the control group with NaCl (30 mM). **f,** AZD3965 strengthens ME1 binding to LDHB in *ME1sg* A549 and HCT116 cells expressing ME1^WT^ but not ME1^R155A^. DMSO treatment for the control group (-). **g,** Prokaryotic His-ME1 promotes LDHB enzymatic activity. **h, i,** Sodium L-lactate increases mitochondrial function in PC3 cells under nutrient-deficient medium (2.5 mM glucose and 0.5 mM glutamine) dependent on ME1-LDHB complex by Seahorse analysis as NaCl treatment for control group (mean ± s.e.m., Two-way ANOVA).

**Figure.S8**

**Supplementary Fig. 8 AZD3965 promotes PC3 cell-xenografted tumor metastasis and colocalization of ME1 with LDHB. a,** Oral administration of AZD3965 (100 mg/kg in 0.5% hydroxypropyl methyl cellulose, 0.1% tween 80 per day) suppresses tumor growth in the *ME1sg* PC3 cells expressing ME1^WT^ or ME1^R155A^ xenograft NSG mouse model compared to the control group with vehicle (n = 6 mice for each group). **b,** Relative lactate level in ME1^WT^ or ME1^R155A^ xenograft tumor tissue (mean ± s.e.m., Two-way ANOVA). **c, d,** Xenograft tumor growth and tumor weight with or without AZD3965 treatment (mean ± s.e.m., Two-way ANOVA). **e**, AZD3965 decreases Ki-67 expression level in the ME1^WT^ but not ME1^R155A^ group according to IHC staining analysis. Representative images (left panel) and statistical analysis (right panel) are shown (n = 6, mean ± s.e.m., Two-way ANOVA). Scale bar, 40 μm. **f**, AZD3965 increases area of lung metastases in the ME1^WT^ group compared to ME1^R155A^ group. Representative images of H&E staining for lung metastases are shown (left). Statistical analyses for area of metastatic foci are shown (mean ± s.e.m., Two-way ANOVA). Scale bar, 40 μm. **g,** Colocalization of ME1 with LDHB in ME1^WT^ or ME1^R155A^ xenograft tumor with or without AZD3965 treatment. Representative images for ME1 and LDHB staining are shown (left). Quantification for Pearson’s correlation of ME1 with LDHB are shown (n = 6, mean ± s.e.m., Two-way ANOVA). Scale bar, 10 μm.

**Figure. S9**

**Supplementary Fig. 9 AZD3965 increases A549 cell-xenografted tumor metastasis and colocalization of ME1 with LDHB. a,** Oral administration of AZD3965 (100 mg/kg in 0.5% hydroxypropyl methyl cellulose, 0.1% tween 80 per day) suppresses tumor growth in the *ME1sg* A549 cells expressing ME1^WT^ or ME1^R155A^ xenograft NSG mouse model compared to the control group with vehicle (n = 6 mice for each group). **b, c,** Xenograft tumor growth and tumor weight with AZD3965 treatment or PBS (mean ± s.e.m., Two-way ANOVA). **d,** Relative lactate level in xenograft tumor tissue with AZD3965 treatment or PBS (mean ± s.e.m., Two-way ANOVA). **e**, Ki-67 expression level according to IHC staining analysis. Representative images (left panel) and statistical analysis (right panel) are shown (n = 6, mean ± s.e.m., Two-way ANOVA). Scale bar, 40 μm. **f**, Area of lung metastases by H&E staining. Statistical analyses for area of metastatic foci are shown (mean ± s.e.m., Two-way ANOVA). Scale bar, 40 μm. **g,** Colocalization of ME1 with LDHB in xenograft tumor with or without AZD3965 treatment. Representative images for ME1 and LDHB staining are shown (left). Quantification for Pearson’s correlation of ME1 with LDHB are shown (n = 6, mean ± s.e.m., Two-way ANOVA). Scale bar, 10 μm.

Table S1. Data collection and refinement statistics

|  | | **ME1-lactate -NADP^+^-Mn^2+^** | **ME1-NADP^+^-Mn^2+^** | |
| --- | --- | --- | --- | --- |
| **Data collection** |  | | |  |
| Space group | P 1 21 1 | | | P 1 21 1 |
| Cell dimensions |  | | |  |
| *a,b,c* (Å) | 70.552, 180.591, 108.038 | | | 70.710, 181.880, 108.800 |
| *α,β,γ* (°) | 90.000, 106.094, 90.000 | | | 90.000, 105.330, 90.000 |
| Wavelength (Å) | 0.9792 | | | 0.9792 |
| Resolution (Å) | 50.00-2.02 (2.05-2.02)* | | | 27.01-2.31 (2.37-2.31)* |
| R_merge_ (%) | 10.3 (82.3) | | | 6.1 (159.1) |
| I / σI | 19.0 (2.0) | | | 20.3 (1.8) |
| Completeness (%) | 91.2 (85.2) | | | 99.9 (100) |
| Redundancy | 6.3 (6.4) | | | 10.4 (10.7) |
| **Refinement** |  | | |  |
| Resolution (Å) | 32.96-2.03 | | | 27.02-2.31 |
| No. reflections | 144,865 | | | 109,754 |
| *R*_work_ / *R*_free_(%) | 17.7/23.8 | | | 17.5/23.9 |
| No. atoms |  | | |  |
| Protein/ligand | 17,984 | | | 17,947 |
| Ion | 4 | | | 4 |
| Water | 1,268 | | | 265 |
| B-factors (Å^2^) | 43.49 | | | 77.36 |
| Protein/ligand | 45.07 | | | 80.36 |
| Ion | 36.04 | | | 74.57 |
| Water | 44.66 | | | 68.16 |
| Ramachandran statistics (%) | | | |  |
| Most favored | 94.57 | | | 92.96 |
| Allowed | 4.50 | | | 5.93 |
| Outliers | 0.93 | | | 1.11 |
| R.m.s. deviations |  | | |  |
| Bond lengths (Å) | 0.008 | | | 0.009 |
| Bond angles (°) | 1.543 | | | 1.679 |
| ***Values in parenthese indicate the highest-resolution shell.** | | | | |

Table S2. Patients information for tissue microarray

| **The clinical information of samples in tissue microarray** | | | | | | | | | | | | |
| --- | --- | --- | --- | --- | --- | --- | --- | --- | --- | --- | --- | --- |
| **Catalog No.** | | | | | | HProA150CS01 | | | | | | |
| **No.** | **Tissue type** | **Tissue ID** | **Gender** | **Age** | **Organ** | **Primary organ (Yes/No)** | **Pathological classification** | **Gleason score** | **T** | **N** | **M** | **8th clinical stage** |
| 1 | Ca/ para-Ca | 1815659 | Man | 74 | Prostate | Yes | Adenocarcinoma | 3+4=7 | T2 | N0 | M0 | 2B |
| 2 | Ca/ para-Ca | 1701493 | Man | 70 | Prostate | Yes | Adenocarcinoma | 3+4=7 | T2 | N0 | M0 | 2B |
| 3 | Ca/ para-Ca | 1705679 | Man | 65 | Prostate | Yes | Adenocarcinoma | 3+4=7 | T2 | N0 | M0 | 2B |
| 4 | Ca/ para-Ca | 1711389 | Man | 76 | Prostate | Yes | Adenocarcinoma | 3+4=7 | T2 | N0 | M0 | 2B |
| 5 | Ca/ para-Ca | 1712283 | Man | 65 | Prostate | Yes | Adenocarcinoma | 3+4=7 | T2 | N0 | M0 | 2B |
| 6 | Ca/ para-Ca | 1729272 | Man | 73 | Prostate | Yes | Adenocarcinoma | 3+4=7 | T2 | N0 | M0 | 2B |
| 7 | Ca/ para-Ca | 1615945 | Man | 57 | Prostate | Yes | Adenocarcinoma | 3+4=7 | T2 | N0 | M0 | 2B |
| 8 | Ca/ para-Ca | 1617235 | Man | 73 | Prostate | Yes | Diffuse infiltrating adenocarcinoma | 4+3=7 | T2 | N0 | M0 | 2C |
| 9 | Ca/ para-Ca | 1645382 | Man | 77 | Prostate | Yes | Adenocarcinoma | 4+3=7 | T2 | N0 | M0 | 2C |
| 10 | Ca/ para-Ca | 1921049 | Man | 68 | Prostate | Yes | Adenocarcinoma | 3+3=6 | T2 | N0 | M0 | 3A |
| 11 | Ca/ para-Ca | 1647448 | Man | 76 | Prostate | Yes | Adenocarcinoma | 4+3=7 | T2 | N0 | M0 | 3A |
| 12 | Ca/ para-Ca | 1810237 | Man | 79 | Prostate | Yes | Adenocarcinoma | 3+3=6 | T4 | N0 | M0 | 3B |
| 13 | Ca/ para-Ca | 1436845 | Man | 78 | Prostate | Yes | Diffuse infiltrating adenocarcinoma | 3+3=6 | T4 | N0 | M0 | 3B |
| 14 | Ca/ para-Ca | 1901662 | Man | 83 | Prostate | Yes | Adenocarcinoma | 3+4=7 | T3 | N0 | M0 | 3B |
| 15 | Ca/ para-Ca | 1802181 | Man | 81 | Prostate | Yes | Adenocarcinoma | 3+4=7 | T3 | N0 | M0 | 3B |
| 16 | Ca/ para-Ca | 1812607 | Man | 62 | Prostate | Yes | Adenocarcinoma | 3+4=7 | T3 | N0 | M0 | 3B |
| 17 | Ca/ para-Ca | 1830109 | Man | 76 | Prostate | Yes | Adenocarcinoma | 3+4=7 | T4 | N0 | M0 | 3B |
| 18 | Ca/ para-Ca | 1834984 | Man | 70 | Prostate | Yes | Adenocarcinoma | 3+4=7 | T3 | N0 | M0 | 3B |
| 19 | Ca/ para-Ca | 1732754 | Man | 63 | Prostate | Yes | Adenocarcinoma | 3+4=7 | T3 | N0 | M0 | 3B |
| 20 | Ca/ para-Ca | 1746937 | Man | 72 | Prostate | Yes | Adenocarcinoma | 3+4=7 | T3 | N0 | M0 | 3B |
| 21 | Ca/ para-Ca | 1626787 | Man | 68 | Prostate | Yes | Adenocarcinoma | 3+4=7 | T4 | N0 | M0 | 3B |
| 22 | Ca/ para-Ca | 1531534 | Man | 68 | Prostate | Yes | Adenocarcinoma | 3+4=7 | T3 | N0 | M0 | 3B |
| 23 | Ca/ para-Ca | 1915589 | Man | 69 | Prostate | Yes | Adenocarcinoma | 4+3=7 | T3 | N0 | M0 | 3B |
| 24 | Ca/ para-Ca | 1812902 | Man | 71 | Prostate | Yes | Adenocarcinoma | 4+3=7 | T4 | N0 | M0 | 3B |
| 25 | Ca/ para-Ca | 1813094 | Man | 72 | Prostate | Yes | Adenocarcinoma | 4+3=7 | T4 | N0 | M0 | 3B |
| 26 | Ca/ para-Ca | 1831974 | Man | 71 | Prostate | Yes | Adenocarcinoma | 4+3=7 | T3 | N0 | M0 | 3B |
| 27 | Ca/ para-Ca | 1735558 | Man | 71 | Prostate | Yes | Adenocarcinoma | 4+3=7 | T4 | N0 | M0 | 3B |
| 28 | Ca/ para-Ca | 1745899 | Man | 74 | Prostate | Yes | Adenocarcinoma | 4+3=7 | T3 | N0 | M0 | 3B |
| 29 | Ca/ para-Ca | 1748012 | Man | 64 | Prostate | Yes | Adenocarcinoma | 4+3=7 | T3 | N0 | M0 | 3B |
| 30 | Ca/ para-Ca | 1442076 | Man | 70 | Prostate | Yes | Adenocarcinoma | 4+3=7 | T3 | N0 | M0 | 3B |
| 31 | Ca/ para-Ca | 1737812 | Man | 72 | Prostate | Yes | Adenocarcinoma | 3+5=8 | T3 | N0 | M0 | 3B |
| 32 | Ca/ para-Ca | 1823731 | Man | 72 | Prostate | Yes | Acinar adenocarcinoma with ductal adenocarcinoma | 4+4=8 | T4 | N0 | M0 | 3B |
| 33 | Ca/ para-Ca | 1824209 | Man | 75 | Prostate | Yes | Adenocarcinoma | 4+4=8 | T3 | N0 | M0 | 3B |
| 34 | Ca/ para-Ca | 1630336 | Man | 70 | Prostate | Yes | Adenocarcinoma | 5+3=8 | T3 | N0 | M0 | 3B |
| 35 | Ca/ para-Ca | 1708153 | Man | 72 | Prostate | Yes | Adenocarcinoma | 4+5=9 | T3 | N0 | M0 | 3C |
| 36 | Ca/ para-Ca | 1716752 | Man | 69 | Prostate | Yes | Adenocarcinoma | 4+5=9 | —— | N0 | M0 | 3C |
| 37 | Ca/ para-Ca | 1732705 | Man | 60 | Prostate | Yes | Adenocarcinoma | 4+5=9 | —— | N0 | M0 | 3C |
| 38 | Ca/ para-Ca | 1504663 | Man | 73 | Prostate | Yes | Adenocarcinoma | 4+5=9 | —— | N0 | M0 | 3C |
| 39 | Ca/ para-Ca | 1725134 | Man | 70 | Prostate | Yes | Adenocarcinoma | 5+4=9 | T3 | N0 | M0 | 3C |
| 40 | Ca/ para-Ca | 1726869 | Man | 61 | Prostate | Yes | Adenocarcinoma | 5+4=9 | T3 | N0 | M0 | 3C |
| 41 | Ca/ para-Ca | 1441266 | Man | 60 | Prostate | Yes | Adenocarcinoma | 5+4=9 | T3 | N0 | M0 | 3C |
| 42 | Ca/ para-Ca | 1726174 | Man | 67 | Prostate | Yes | Adenocarcinoma | 5+5=10 | T3 | N0 | M0 | 3C |
| 43 | Ca/ para-Ca | 1735625 | Man | 69 | Prostate | Yes | Adenocarcinoma | 5+5=10 | T4 | N0 | M0 | 3C |
| 44 | Ca/ para-Ca | 1644793 | Man | 62 | Prostate | Yes | Adenocarcinoma | 3+3=6 | —— | N1 | M0 | 4A |
| 45 | Ca/ para-Ca | 1833726 | Man | 78 | Prostate | Yes | Adenocarcinoma | 3+4=7 | —— | N1 | M0 | 4A |
| 46 | Ca/ para-Ca | 1851780 | Man | 73 | Prostate | Yes | Adenocarcinoma | 3+4=7 | T3 | N1 | M0 | 4A |
| 47 | Ca/ para-Ca | 1912914 | Man | 72 | Prostate | Yes | Adenocarcinoma | 4+3=7 | T3 | N1 | M0 | 4A |
| 48 | Ca/ para-Ca | 1817685 | Man | 78 | Prostate | Yes | Acinar adenocarcinoma and ductal adenocarcinoma with neuroendocrine differentiation | 4+3=7 | T3 | N1 | M0 | 4A |
| 49 | Ca/ para-Ca | 1437757 | Man | 74 | Prostate | Yes | Diffuse infiltrating adenocarcinoma | 3+5=8 | T4 | N1 | M0 | 4A |
| 50 | Ca/ para-Ca | 1836631 | Man | 65 | Prostate | Yes | Adenocarcinoma | 5+4=9 | T3 | N1 | M0 | 4A |
| 51 | Ca | 1626161 | Man | 62 | Prostate | Yes | Adenocarcinoma | 3+3=6 | T2 | N0 | M0 | 2A |
| 52 | Ca | 1831747 | Man | 72 | Prostate | Yes | Adenocarcinoma | 3+4=7 | T2 | N0 | M0 | 2B |
| 53 | Ca | 1645979 | Man | 62 | Prostate | Yes | Adenocarcinoma | 3+4=7 | T2 | N0 | M0 | 2B |
| 54 | Ca | 1829094 | Man | 83 | Prostate | Yes | Adenocarcinoma | 3+3=6 | T3 | N0 | M0 | 3B |
| 55 | Ca | 1901688 | Man | 76 | Prostate | Yes | Adenocarcinoma | 3+4=7 | T3 | N0 | M0 | 3B |
| 56 | Ca | 1907856 | Man | 72 | Prostate | Yes | Adenocarcinoma | 3+4=7 | T3 | N0 | M0 | 3B |
| 57 | Ca | 1841003 | Man | 63 | Prostate | Yes | Acinar adenocarcinoma, partly ductal adenocarcinoma | 3+4=7 | T3 | N0 | M0 | 3B |
| 58 | Ca | 1731274 | Man | 70 | Prostate | Yes | Adenocarcinoma | 3+4=7 | T3 | N0 | M0 | 3B |
| 59 | Ca | 1634261 | Man | 69 | Prostate | Yes | Adenocarcinoma | 3+4=7 | T3 | N0 | M0 | 3B |
| 60 | Ca | 1643530 | Man | 75 | Prostate | Yes | Adenocarcinoma | 3+4=7 | T3 | N0 | M0 | 3B |
| 61 | Ca | 1853827 | Man | 76 | Prostate | Yes | Adenocarcinoma | 4+3=7 | T3 | N0 | M0 | 3B |
| 62 | Ca | 1707338 | Man | 71 | Prostate | Yes | Adenocarcinoma | 4+3=7 | T3 | N0 | M0 | 3B |
| 63 | Ca | 1743928 | Man | 71 | Prostate | Yes | Adenocarcinoma | 4+3=7 | T3 | N0 | M0 | 3B |
| 64 | Ca | 1745534 | Man | 76 | Prostate | Yes | Adenocarcinoma | 4+3=7 | T3 | N0 | M0 | 3B |
| 65 | Ca | 1717393 | Man | 76 | Prostate | Yes | Adenocarcinoma | 3+5=8 | T3 | N0 | M0 | 3B |
| 66 | Ca | 1917991 | Man | 77 | Prostate | Yes | Adenocarcinoma | 4+4=8 | T4 | N0 | M0 | 3B |
| 67 | Ca | 1822734 | Man | 65 | Prostate | Yes | Adenocarcinoma | 4+4=8 | T4 | N0 | M0 | 3B |
| 68 | Ca | 1826787 | Man | 64 | Prostate | Yes | Adenocarcinoma | 4+4=8 | T3 | N0 | M0 | 3B |
| 69 | Ca | 1750116 | Man | 69 | Prostate | Yes | Adenocarcinoma | 4+4=8 | T3 | N0 | M0 | 3B |
| 70 | Ca | 1809044 | Man | 71 | Prostate | Yes | Adenocarcinoma | 5+3=8 | T3 | N0 | M0 | 3B |
| 71 | Ca | 1809099 | Man | 70 | Prostate | Yes | Adenocarcinoma | 5+3=8 | T4 | N0 | M0 | 3B |
| 72 | Ca | 1816666 | Man | 83 | Prostate | Yes | Adenocarcinoma | 5+3=8 | T3 | N0 | M0 | 3B |
| 73 | Ca | 1728815 | Man | 68 | Prostate | Yes | Adenocarcinoma | 5+3=8 | T3 | N0 | M0 | 3B |
| 74 | Ca | 1802887 | Man | 75 | Prostate | Yes | Adenocarcinoma | 4+5=9 | T3 | N0 | M0 | 3C |
| 75 | Ca | 1708329 | Man | 74 | Prostate | Yes | Adenocarcinoma | 4+5=9 | T3 | N0 | M0 | 3C |
| 76 | Ca | 1734545 | Man | 63 | Prostate | Yes | Adenocarcinoma | 4+5=9 | T3 | N0 | M0 | 3C |
| 77 | Ca | 1745275 | Man | 81 | Prostate | Yes | Adenocarcinoma | 4+5=9 | T3 | N0 | M0 | 3C |
| 78 | Ca | 1535289 | Man | 62 | Prostate | Yes | Adenocarcinoma | 4+5=9 | T3 | N0 | M0 | 3C |
| 79 | Ca | 1840203 | Man | 75 | Prostate | Yes | Adenocarcinoma, partly micropapillary | 5+4=9 | T3 | N0 | M0 | 3C |
| 80 | Ca | 1631153 | Man | 79 | Prostate | Yes | Adenocarcinoma | 5+4=9 | T4 | N0 | M0 | 3C |
| 81 | Ca | 1502298 | Man | 59 | Prostate | Yes | Diffuse infiltrating adenocarcinoma | 5+4=9 | T3 | N0 | M0 | 3C |
| 82 | Ca | 1503783 | Man | 57 | Prostate | Yes | Diffuse infiltrating adenocarcinoma | 5+4=9 | T2 | N0 | M0 | 3C |
| 83 | Ca | 1542888 | Man | 67 | Prostate | Yes | Adenocarcinoma | 5+4=9 | T4 | N0 | M0 | 3C |
| 84 | Ca | 1545441 | Man | 70 | Prostate | Yes | Adenocarcinoma | 5+4=9 | T3 | N0 | M0 | 3C |
| 85 | Ca | 1901853 | Man | 74 | Prostate | Yes | Adenocarcinoma | 4+3=7 | T3 | N1 | M0 | 4A |
| 86 | Ca | 1819155 | Man | 76 | Prostate | Yes | Adenocarcinoma | 4+3=7 | T4 | N1 | M0 | 4A |
| 87 | Ca | 1834254 | Man | 69 | Prostate | Yes | Adenocarcinoma | 4+3=7 | —— | N1 | M0 | 4A |
| 88 | Ca | 1852914 | Man | 73 | Prostate | Yes | Acinar adenocarcinoma | 4+3=7 | T3 | N1 | M0 | 4A |
| 89 | Ca | 1633940 | Man | 62 | Prostate | Yes | Adenocarcinoma | 4+3=7 | T3 | N1 | M0 | 4A |
| 90 | Ca | 1834429 | Man | 76 | Prostate | Yes | Adenocarcinoma | 3+5=8 | T4 | N1 | M0 | 4A |
| 91 | Ca | 1814639 | Man | 83 | Prostate | Yes | Adenocarcinoma | 4+4=8 | T3 | N1 | M0 | 4A |
| 92 | Ca | 1821857 | Man | 83 | Prostate | Yes | Adenocarcinoma | 4+4=8 | T4 | N1 | M0 | 4A |
| 93 | Ca | 1850060 | Man | 72 | Prostate | Yes | Adenocarcinoma | 4+4=8 | T3 | N1 | M0 | 4A |
| 94 | Ca | 1744584 | Man | 67 | Prostate | Yes | Adenocarcinoma | 4+4=8 | T3 | N1 | M0 | 4A |
| 95 | Ca | 1714453 | Man | 71 | Prostate | Yes | Adenocarcinoma | 4+5=9 | T3 | N1 | M0 | 4A |
| 96 | Ca | 1747577 | Man | 78 | Prostate | Yes | Adenocarcinoma | 4+5=9 | T3 | N1 | M0 | 4A |
| 97 | Ca | 1410041 | Man | 70 | Prostate | Yes | Adenocarcinoma | 4+5=9 | T3 | N1 | M0 | 4A |
| 98 | Ca | 1429514 | Man | 63 | Prostate | Yes | Adenocarcinoma | 4+5=9 | T3 | N1 | M0 | 4A |
| 99 | Ca | 1919120 | Man | 76 | Prostate | Yes | Adenocarcinoma | 5+4=9 | T3 | N1 | M0 | 4A |
| 100 | Ca | 1627033 | Man | 62 | Prostate | Yes | Adenocarcinoma | 5+4=9 | T3 | N1 | M0 | 4A |

**Data S1. (separate file)**

Data of 2-DG treatment

**Data S2. (separate file)**

PDB reports for 23JW and 23JV

**Data S3.** **(separate file)**

Original films of western blots
