# Supplementary material for: Malic enzyme 1 senses L-lactate to determine tumor heterogeneity
Source: Signal Transduct Target Ther. 2026 Sep 11;11:386. doi: 10.1038/s41392-026-02839-6 (PMC13569829; doi:10.1038/s41392-026-02839-6)
Supplement: Supplementary file 5 [file 41392_2026_2839_MOESM3_ESM.docx]

**Data S1**

**2-DG treatment increases ME1 K362 acetylation and inhibits ME1 nuclear translocation to inhibit migration.** (a) Schematic model of 2-DG. (b) 2-DG treatment (20 mM) decrease intercellular L-lactate level in PC3 cells. (c) ME1 expression level with 2-DG treatment under normal medium by western blot. (d) 2-DG increases ME1 K362 acetylation level and decreases binding to SIRT5 and KPNA4 in ME1^WT^ cells, but not ME1^R155A^ cells. (e) 2-DG inhibits ME1 nuclear translocation in ME1^WT^ cells, but not ME1^R155A^ cells. (f) Representative images of cell migration and quantification with 2-DG treatment or vehicle in ME1^WT^ and ME1^R155A^ cells.
